# Divergent Total Synthesis of Denudatine Alkaloids Cochlearenine, Macrocentrine, Dictizine, 15‐Veratroyl‐17‐Acetyl‐19‐Oxodictizine, and the Proposed Structure of Acochlearine

**DOI:** 10.1002/anie.202521481

**Published:** 2025-12-04

**Authors:** Shun Kawano, Naoya Miyamoto, Kosuke Fujioka, Hiroki Toya, Juri Sakata, Hidetoshi Tokuyama

**Affiliations:** ^1^ Graduate School of Pharmaceutical Sciences Tohoku University Aoba 6‐3 Aramaki, Aoba‐ku 980–8578 Sendai

**Keywords:** Diels–Alder reaction, Diterpenoid alkaloids, Divergent synthesis, Mannich reaction, Total synthesis

## Abstract

The first asymmetric total syntheses of four denudatine alkaloids, cochlearenine, macrocentrine, dictizine, and 15‐veratroyl‐17‐acetyl‐19‐oxodictizine, along with the asymmetric synthesis of the proposed structure of acochlearine, were accomplished in a divergent manner. Highly fused tetracyclic skeletons (A/B/E/F rings) with different *N*‐alkyl groups (Me and Et) were constructed by Brønsted acid promoted intramolecular Mannich reactions. The bicyclo[2.2.2]octane C/D rings were constructed via two‐fold intermolecular Diels–Alder reactions. By utilizing these key processes, the common intermediates, possessing the complex cage‐like hexacyclic skeleton, were constructed. From these common intermediates, the four denudatine alkaloids were synthesized through late‐stage, chemo‐ and stereoselective modifications around the A/C rings. The spectroscopic data of the proposed structure of acochlearine were inconsistent with those of natural acochlearine.

## Graphical abstract

An intramolecular Mannich reaction (A/B/E/F rings) and intermolecular Diels–Alder reactions (C/D rings) afforded a cage‐like hexacyclic core skeleton of denudatine alkaloids. Derivatization of the hexacyclic intermediate enabled the first asymmetric total syntheses of four denudatine alkaloids: (–)‐cochlearenine, (–)‐macrocentrine, (–)‐dictizine, and (–)‐15‐veratroyl‐17‐acetyl‐19‐oxodictizine (see scheme).

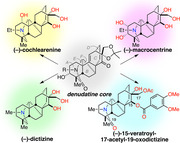

## Introduction

**Figure 1:**
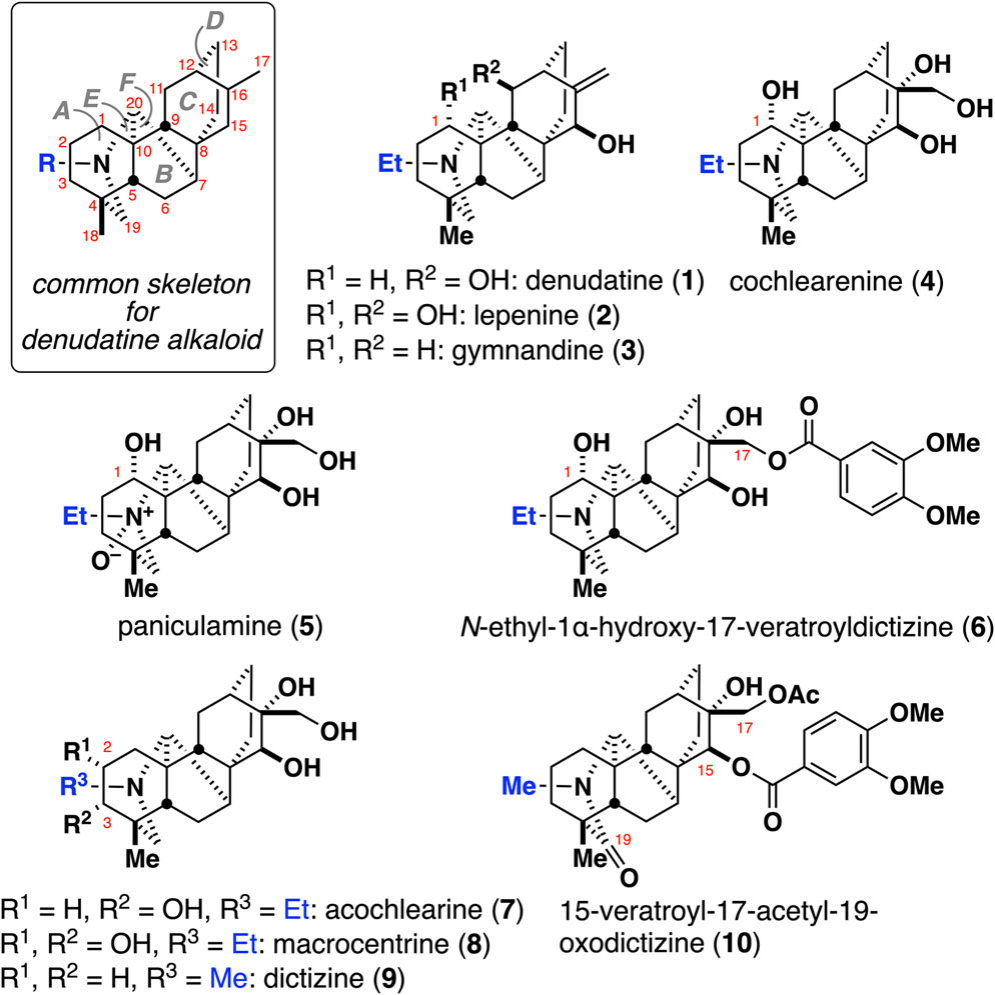
Structures of denudatine alkaloids.

Diterpenoid alkaloids, isolated from the plant genera *Aconitium* and *Delphinium*, constitute a vast array of over 1,200 congeners,^[^ ^1^, ^2^, ^3^, ^4^ ^]^ which are categorized into C_20_, C_19_, and C_18_ subgroups based on the number of carbons forming their main frameworks. The denudatine family comprises 60 compounds in the C_20_ subgroup. The denudatines possess a densely fused hexacyclic ring system formed by fusion of *aza*‐bicyclo[3.3.1]nonane (A/E rings), bicyclo[2.2.1]heptane (B/F rings), and bicyclo[2.2.2]octane skeletons (C/D rings). The A and C rings are highly decorated with oxygen functionalities (Figure 1). Several congeners have demonstrated promising biological activity; for instance, cochlearenine (**4**) behaves as an antioxidant,^[^ ^5^ ^]^ dictizine (**9**) exerts antiarrhythmic activity,^[^ ^6^ ^]^ aconicarnine A and D display analgesic effects,^[^ ^7^ ^]^ and kirisine G inhibits acetylcholinesterase.^[^ ^8^ ^]^ The denudatine alkaloids have also attracted interest as biosynthetic^[^ ^9^ ^]^ precursors of aconitine alkaloids with extremely potent biological activities, such as inhibition of voltage‐dependent sodium ion channels.

Given their structural and biological importance, the denudatine alkaloids have attracted considerable interest from the synthetic community.^[^ ^10^, ^11^, ^12^, ^13^, ^14^, ^15^, ^16^, ^17^ ^]^ In the late 1970s, Wiesner and co‐workers reported the total synthesis of the aconitine‐type alkaloid, (±)‐chasmanine via a bioinspired rearrangement of denudatine framework.^[^ ^10^, ^11^, ^18^ ^]^ Several decades later in 2014, Fukuyama and Yokoshima accomplished the total synthesis of (–)‐lepenine (**2**).^[^ ^19^ ^]^ Subsequently, Sarpong and co‐workers established the unified total syntheses of (±)‐cochlearenine (**4**), (±)‐paniculamine (**5**), and (±)‐*N*‐ethyl‐1α‐hydroxy‐17‐veratroyldictizine (**6**), along with other C_18_ and C_19_ diterpenoid alkaloids.^[^ ^20^, ^21^ ^]^ Adopting a bioinspired approach, Qin and co‐workers reported the unified total synthesis of (±)‐gymnandine (**3**) and three other types of C_20_ diterpenoid alkaloids.^[^ ^22^ ^]^ Despite these elegant approaches, no divergent routes to denudatine alkaloids had been reported.

**Figure 2:**
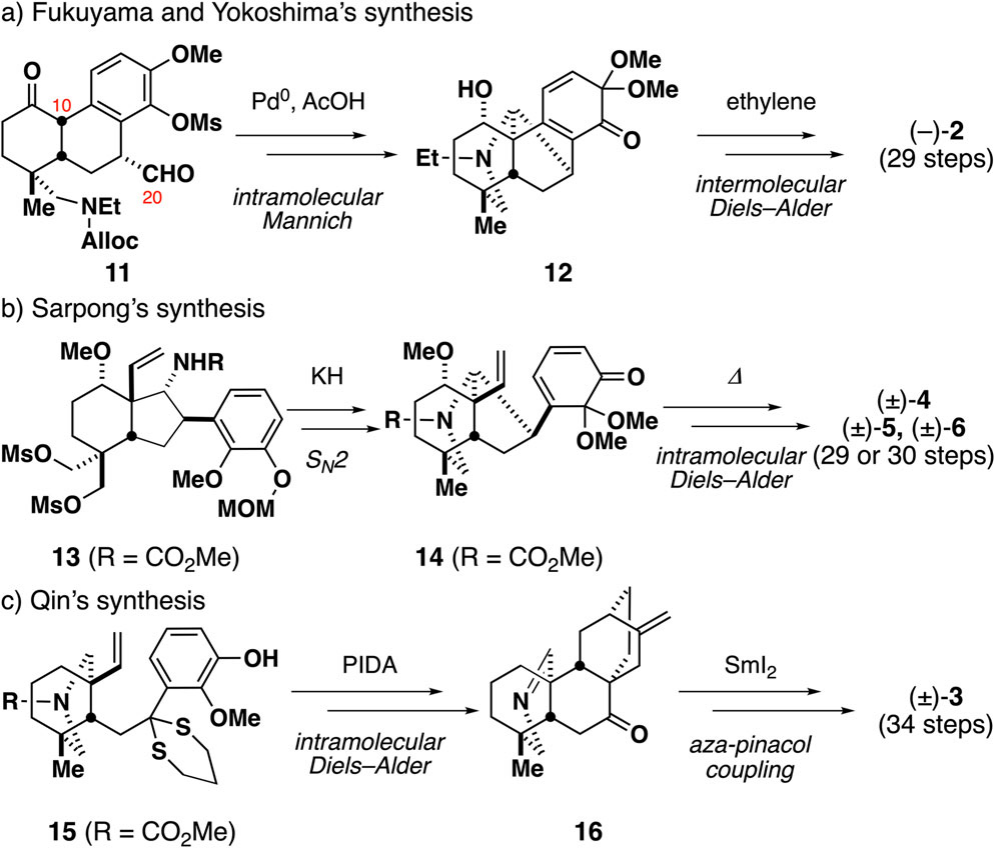
a) The key process in Fukuyama and Yokoshima's synthesis of (–)‐lepenine. b) The key process in Sarpong's syntheses of denudatine alkaloids. c) The key process in Qin's synthesis of (±)‐gymnandine.

To develop an efficient and divergent route to denudatine alkaloids, three major synthetic challenges needed to be addressed. The first task should be the construction of nitrogen‐containing tetracyclic A/B/E/F rings containing *aza*‐bicyclo[3.3.1]nonane and bicyclo[2.2.1]heptane rings. The second would be formation of bicyclo[2.2.2]octane C/D rings. The final challenge should be the installation of four to five oxygen functionalities at the requisite positions with the correct stereochemistries. To construct nitrogen‐containing tetracyclic A/B/E/F rings containing *aza*‐bicyclo[3.3.1]nonane and bicyclo[2.2.1]heptane rings, Fukuyama and Yokoshima performed an intramolecular Mannich reaction of tricyclic ketone **11**, forming the crucial C10–C20 bond (Figure 2a). Sarpong formed the E ring through an intramolecular S*_N_* 2 reaction of tricyclic carbamate **13** (Figure 2b). Qin formed the F ring via intramolecular *aza*‐pinacol coupling of imine **16** (Figure 2c). The bicyclo[2.2.2]octane C/D rings were intermolecularly (Fukuyama and Yokoshima, Figure 2a) or intramolecularly (Sarpong and Qin, Figure 2b,c) formed through Diels–Alder reactions. The third challenge is the key to realizing divergent synthesis of a broad range of denudatine alkaloids. However, as a divergent strategy enabling diverse oxidation states of the A and C rings is lacking, most of the denudatine alkaloids have not yet been synthesized. Herein, we report the asymmetric synthesis of the proposed structure of acochlearine (**7**)^[^ ^23^, ^24^ ^]^ and the first asymmetric total syntheses of four denudatine‐type diterpenoid alkaloids: cochlearenine (**4**),^[^ ^25^, ^26^ ^]^ macrocentrine (**8**),^[^ ^27^ ^]^ dictizine (**9**),^[^ ^28^, ^29^, ^30^ ^]^ and 15‐veratroyl‐17‐acetyl‐19‐oxodictizine (**10**)^[^ ^31^ ^]^ via chemo‐ and stereoselective late‐stage modifications on the A ring.

## Results and Discussion

**Scheme 1:**
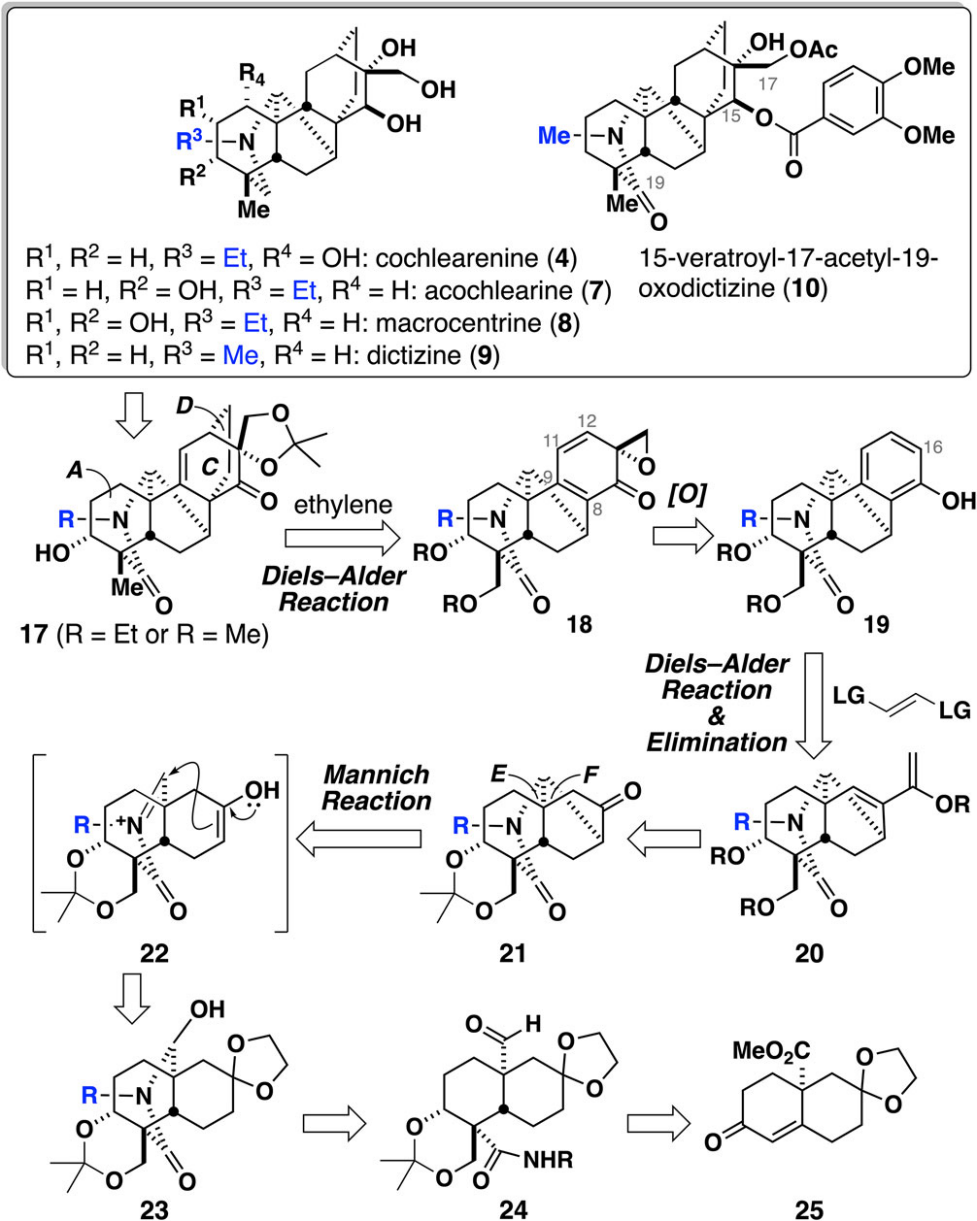
Retrosynthetic analysis.

Retrosynthetic analysis is depicted in Scheme 1. We expected that late‐stage chemo‐ and stereoselective transformations around the A and C rings would lead to five denudatine alkaloids: acochlearine (**7**), cochlearenine (**4**), macrocentrine (**8**), dictizine (**9**), and 15‐veratroyl‐17‐acetyl‐19‐oxodictizine (**10**). Accordingly, we selected the keto‐diol acetonide **17** (R = Et or Me) as a common precursor. The bicyclo[2.2.2]octane C/D rings should be constructed following Fukuyama and Yokoshima's intermolecular Diels–Alder strategy using diene **18** and ethylene.^[^ ^19^ ^]^ The keto‐diol acetonide moiety can be formed from phenol **19** by electrophilic hydroxymethylation, followed by oxidative keto‐epoxide formation and ring‐opening hydration. To construct the phenol ring of **19**, we planned the conversion of the key tetracyclic intermediate **21** to diene **20** and the Diels–Alder reaction with the dienophile bearing two leaving groups,^[^ ^32^ ^]^ followed by eliminations. During our synthetic campaign toward denudatine alkaloids, we previously synthesized the key tetracyclic ketone **21** via a cascade reaction including radical‐mediated bromination of the lactam ring and the Mannich reaction, and then constructed the C ring via electrocyclic reaction of a triene intermediate.^[^ ^33^ ^]^ However, this approach was discontinued because the sequential Mannich reaction was poorly reproducible and the D ring was difficult to construct.^[^ ^33^ ^]^ In this study, we revise the strategy with hemiaminal **23** as the precursor of the acyliminium ion **22**. Hemiaminal **23** can be prepared by converting the known optically active enone **25** ^[^ ^34^ ^]^ to **24** followed by intramolecular condensation of amide and aldehyde.

**Scheme 2:**
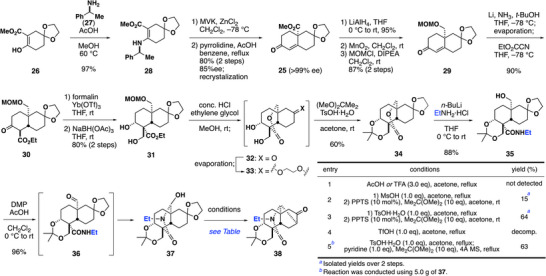
Synthesis of the tetracyclic ketone **38** including optimization of the key intramolecular Mannich reaction (Table).

Our synthesis commenced with the preparation of enone **25** via a modified Costa's Robinson annulation including an asymmetric Michael reaction (Scheme 2).^[^ ^34^ ^]^ A chiral enamine **28** was prepared via condensation of the commercially available ester **26** and (*S*)‐(–)‐1‐phenylethylamine (**27**),^[^ ^35^ ^]^ and then subjected to the asymmetric Michael reaction with methyl vinyl ketone and subsequent the intramolecular aldol condensation obtaining enone **25** in 80% yield (85% ee). The enone **25** was then recrystallized to obtain **25** in optically pure form (>99% ee). Reduction of both ketone and ester, chemoselective oxidation of the resulting allylic alcohol, and protection of the primary alcohol with the methoxymethyl (MOM) group afforded enone **29**, which was subjected to the Birch reduction condition followed by *C*‐acylation of the generated lithium enolate with Mander's reagent, furnishing ketoester **30** as a single isomer.^[^ ^36^ ^]^ The Yb(OTf)_3_‐catalyzed diastereoselective aldol reaction^[^ ^37^ ^]^ of **30** with formalin and stereoselective reduction of a ketone with NaBH(OAc)_3_ afforded 1,3‐diol **31** as a single isomer.^[^ ^38^ ^]^ Conversion of the ester to *N*‐ethylamide is rendered difficult by steric hindrance, but was realized via ring opening of a lactone derivative with lithium ethylamide. The MOM group was removed by treating **31** with conc. HCl in a mixture of MeOH and ethylene glycol. Lactonization and removal of the C8 ketal yielded a mixture of keto‐lactone **32** with a trace amount of ketal **33**. Removal of MeOH and H_2_O by evaporation promoted reprotection of the C8 ketone, providing **33**. One‐pot protection of the 1,3‐diol with acetonide then afforded lactone **34**. Lactone opening of **34** by lithium ethylamide afforded the desired *N*‐ethylamide **35**. Oxidation of **35** with Dess–Martin periodinane (DMP) in the presence of acetic acid generated the hemiaminal **37**.

Having prepared the acyliminium ion precursor, we investigated the key intramolecular Mannich reaction (Scheme 2, table). The initial trial using weak Brønsted acids such as acetic acid or trifluoroacetic acid (TFA) yielded no observable Mannich products and only 1,3‐diol was formed via removal of acetonide (entry 1). The reaction using MsOH in acetone provided a mixture of Mannich product **38** and its 1,3‐diol derivative generated by removal of the acetonide. This mixture was subjected to acetonide formation conditions to obtain Mannich product **38** in 15% yield over two steps (entry 2). After extensive investigations, *p*‐toluenesulfonic acid monohydrate (TsOH·H_2_O) was identified as the optimal Brønsted acid, affording **38** in 64% yield after reprotection of the partially generated 1,3‐diol (entry 3). A stronger acid, TfOH, only decomposed the starting material (entry 4). Finally, we established a reproducible Gram‐scale protocol with improved one‐pot reprotection of 1,3‐diol using 2,2‐dimethoxypropane, molecular sieves 4A (4Å MS), and pyridine under reflux (entry 5).

**Scheme 3:**
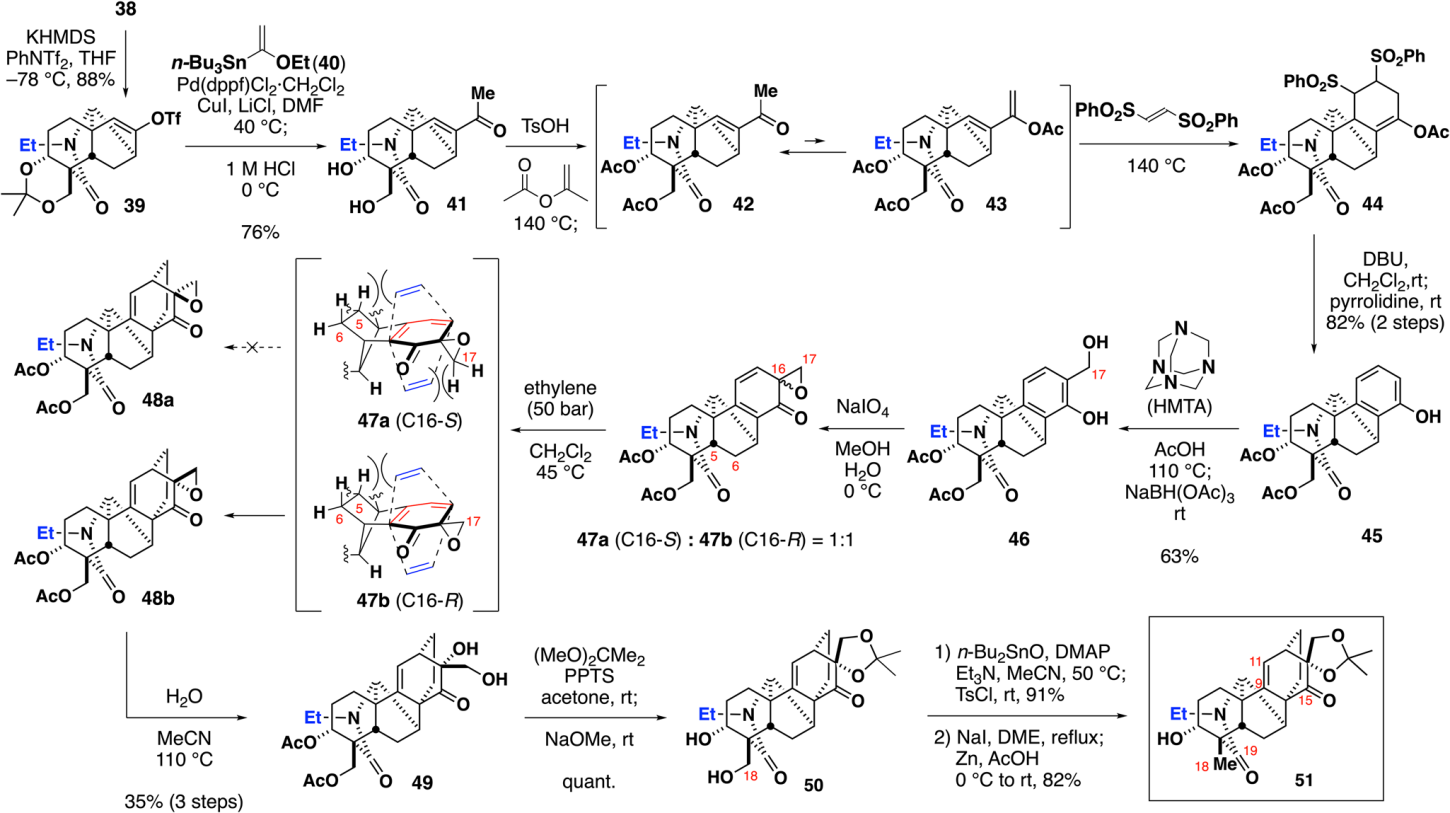
Synthesis of the common hexacyclic precursor of denudatine alkaloids.

We then constructed the C ring (Scheme 3). Ketone **38** was converted to enol triflate **39**,^[^ ^33^ ^]^ which was subjected to Stille coupling with tributyl(1‐ethoxyvinyl)stannane (**40**) and one‐pot acidic hydrolysis of the resultant vinyl ether and acetonide, furnishing keto‐diol **41**. The C ring was successfully constructed through a modified Alvarez–Manzaneda's Diels–Alder protocol.^[^ ^39^ ^]^ Upon heating keto‐diol **41** with isopropenyl acetate in the presence of a catalytic amount of TsOH, enone **42** established equilibrium with acetoxydiene **43**. One‐pot addition of *trans*‐1,2‐bis(phenylsulfonyl)ethylene then facilitated the Diels–Alder reaction and shifted the equilibrium toward the formation of **43**. Cycloadduct **44**, obtained as a diastereomeric mixture, underwent two‐fold elimination of its sulfonyl groups with 1,8‐diazabicyclo[5.4.0]undec‐7‐ene (DBU). After subsequent selective aminolysis of acetylphenol, phenol **45** was obtained in 82% yield over two steps from **41**.^[^ ^40^ ^]^

The D ring was then formed via the second Diels–Alder reaction. Prior to the Diels–Alder reaction, we introduced a C17 carbon and oxygen functionalities on the C ring. Regioselective formylation of phenol **45** by hexamethylenetetramine (HMTA),^[^ ^41^, ^42^, ^43^ ^]^ reduction of the formyl group, and NaIO_4_‐mediated oxidative dearomatization of **46** provided a diastereomeric mixture of dienes **47a** and **47b** in a 1:1 ratio.^[^ ^44^, ^45^ ^]^ As all attempts to isolate^[^ ^46^ ^]^ or selectively synthesize^[^ ^47^ ^]^ the desired **47b** were unsuccessful, the **47a**/**47b** mixture was subjected to the Diels–Alder reaction under pressurized ethylene (50 bar).^[^ ^19^ ^]^ Interestingly, only diene **47b** underwent the expected Diels–Alder reaction, affording the cycloadduct **48b** with perfect facial selectivity while leaving **47a** intact. The low reactivity of **47a** was attributed to steric hindrances of the α‐ and β‐faces caused by the two hydrogen atoms at C5 and C6 and by the methylene carbon of epoxide, respectively. In contrast, the sterically less‐hindered α‐face of **47b** could be approached by the dienophile while its β‐face was shielded by the C5 and C6 hydrogen atoms.^[^ ^48^, ^49^ ^]^ The inseparable mixture of **48b** and **47a** was then hydrated in refluxing acetonitrile, providing the 1,2‐diol **49** in 35% yield over three steps from **46**. Protection of 1,2‐diol **49** as acetonide, followed by removal of the two acetyl groups, afforded the 1,3‐diol **50**. Finally, the hydroxyl group at C18 was selectively removed via Hanessian's protocol,^[^ ^50^ ^]^ which converted **50** to monotosylate. Replacement of the TsO‐ group with an iodine atom and reductive deiodination then furnished **51**, the key precursor of the planned divergent synthesis of denudatine alkaloids.

**Scheme 4:**
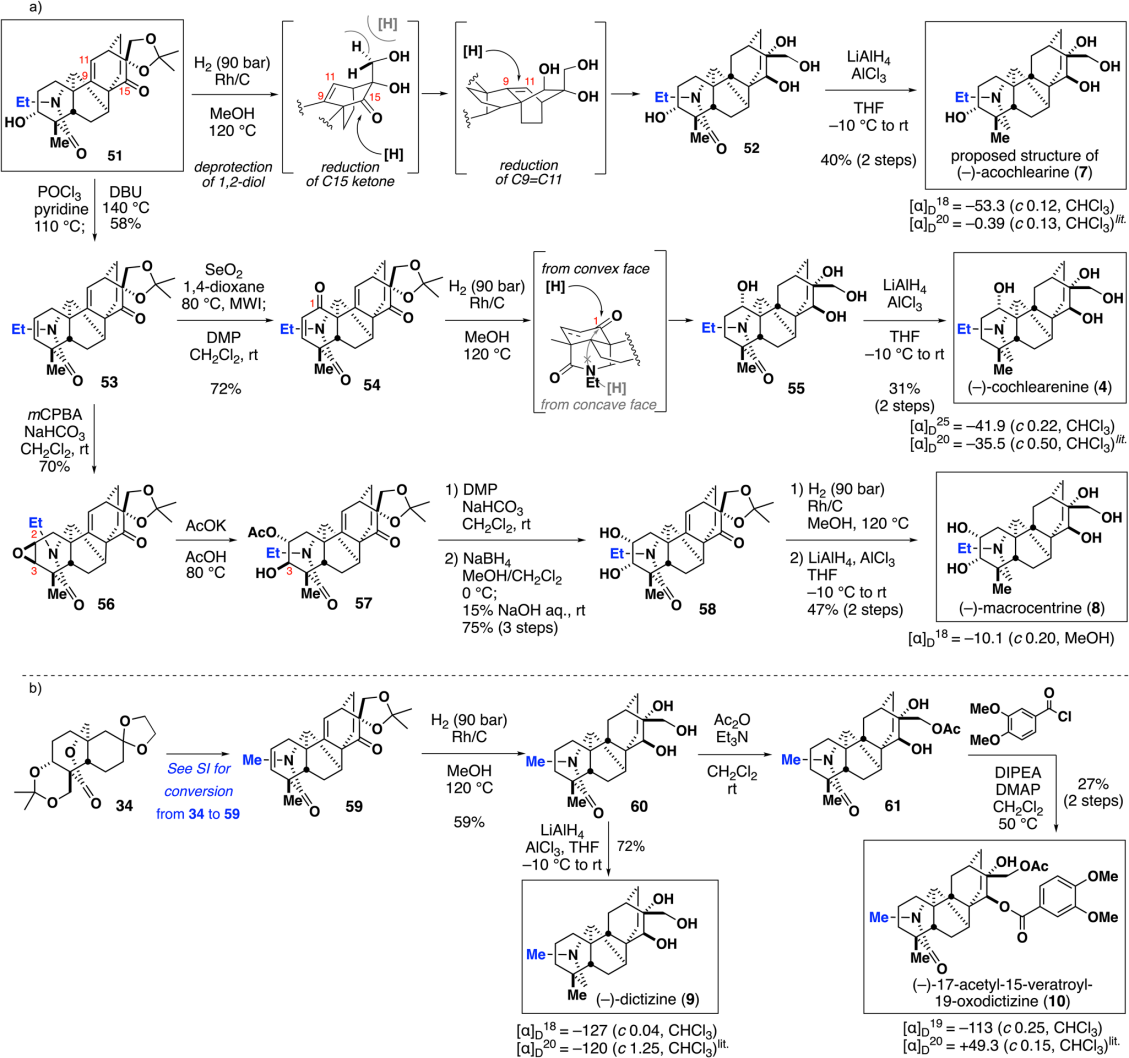
a) Synthesis of the proposed structure of (–)‐acochlearine (**7**), and total syntheses of (–)‐cochlearenine (**4**) and (–)‐macrocentrin (**8**). b) Total syntheses of (–)‐dictizine (**9**) and (–)‐17‐acetyl‐15‐veratroyl‐19‐oxodictizine (**10**)

The key precursor **51** was then diversified to synthesize various denudatine alkaloids. First, we targeted acochlearine (**7**) (Scheme 4a).^[^ ^23^, ^24^ ^]^ To achieve total synthesis of acochlearine (**7**), we needed to reduce the C9–C11 double bond, C15 ketone, and C19 lactam. Initially, we explored the hydrogenation conditions for reducing the C9–C11 double bond. Surprisingly, the reaction condition with rhodium/carbon (Rh/C) in methanol under a pressurized hydrogen atmosphere (90 bar) promoted not only the hydrogenation of the C9–C11 double bond, but also 1,2‐reduction of the C15 ketone^[^ ^51^, ^52^, ^53^ ^]^ and removal of acetonide affording lactam **52** as a single isomer.^[^ ^54^, ^55^ ^]^ Finally, lactam **52** was reduced by alane generated from LiAlH_4_ and AlCl_3_, furnishing the reported structure of acochlearine (**7**). Structure of synthetic **7** was unambiguously proved by 2D NMR, including COSY, HMQC, HMBC, and NOESY. Stereoselectivities of the 1,2‐reduction of ketone and the hydrogenation of the C9–C11 double bond were confirmed by extensive NMR analyses after conversion to **7**. Mass spectrometry analysis revealed that the reaction was initiated by deprotection of the acetonide, followed by 1,2‐reduction of ketone and hydrogenation of the C9–C11 double bond. During the 1,2‐reduction of ketone, hydride was delivered from the sterically less‐hindered α‐face, possibly avoiding the C16 hydroxylmethyl group. The selective hydrogenation of the C9–C11 double bond^[^ ^56^ ^]^ could be explained by the directing effects of the hydroxyl groups at C17 and C15.^[^ ^57^ ^]^

Surprisingly, the ^1^H‐NMR and ^13^C‐NMR spectral data and specific rotation of **7** disagreed with the reported data of natural (–)‐acochlearine (see Supporting Information for the details).^[^ ^23^, ^24^ ^]^ Sarpong and co‐workers similarly reported discrepancies in the NMR data of synthetic *N*‐ethyl‐1α‐hydroxy‐17‐veratroyldictizine (**6**) and the reported data of natural **6**.^[^ ^20^, ^21^ ^]^ Eventually, they found that the ^1^H‐ and ^13^C‐NMR data of the TFA salt of synthetic **6** fully match those of natural **6**. Motivated by Sarpong's report, we investigated the effect of acids on the ^1^H‐ and ^13^C‐NMR chemical shifts of **7** through titration experiments. However, under all conditions, the ^1^H‐NMR data of synthetic **7** remained inconsistent with the reported data (see Supporting Information for the details).^[^ ^58^ ^]^ These results indicate that the correct structure of acochlearine is not that the reported structure, and remains unclear.

We then targeted (–)‐cochlearenine (**4**), bearing an oxygen functionality at C1. Dehydration of alcohol **51**, followed by allylic oxidation of the resulting **53** by SeO_2_, provided a mixture of enone **54** and an allylic alcohol intermediate. One‐pot treatment of this nonseparated mixture with Dess–Martin periodinane afforded enone **54**, which underwent the Rh/C‐mediated global reductions of C2–C3 and C9–C11 double bonds and C15 ketone, along with 1,2‐reduction of C1 ketone and removal of acetonide, affording lactam **55** as a single isomer. Finally, reduction of lactam **55** with alane provided (–)‐cochlearenine (**4**). The ^1^H‐NMR, ^13^C‐NMR, and other spectroscopic data of **4** perfectly matched the reported data.^[^ ^20^, ^21^, ^25^, ^26^ ^]^

Next, we targeted (–)‐macrocentrine (**8**). The synthetic challenge was the construction of 1,2‐*cis*‐diol at the sterically hindered α‐face. Reaction of **53** with *m*‐chloroperoxybenzoic acid (*m*CPBA) provided β‐epoxide **56** as a single isomer, which was selectively opened at the C2 position by potassium acetate, yielding **57**. The undesired stereochemistry at C3 was inverted, obtaining 1,2‐*cis*‐diol **58** via the oxidation/reduction protocol followed by hydrolysis of the acetyl group. Then, **58** was subjected to Rh/C‐mediated global reductions, followed by reduction of lactam to complete the first total synthesis of (–)‐macrocentrine (**8**). The^1^H‐ and ^13^C‐NMR spectra of **8** perfectly agreed with the reported data.^[^ ^27^ ^]^

Using lactone **34** as a pivotal intermediate, the divergent strategy was further expanded to congeners with an *N*‐methy group. As the target compounds, we selected (–)‐dictizine (**9**) and (–)‐15‐veratroyl‐17‐acetyl‐19‐oxodictizine (**10**) (Scheme 4b). The *N*‐Me group was introduced by opening lactone **34** using lithium methylamide to give the corresponding *N*‐ methylamide, which was converted into **59** in the same manner as the synthesis of *N*‐ethyl derivative **53** (see Supporting Information for the details). The Rh/C‐mediated global reduction protocol, followed by the reduction of lactam **60**, accomplished the first total synthesis of (–)‐dictizine (**9**).^[^ ^28^, ^29^, ^30^ ^]^ In addition, sequential introductions of acetyl and veratroyl groups on the primary and secondary alcohols of **60**, respectively, achieved the first total synthesis of (–)‐15‐veratroyl‐17‐acetyl‐19‐oxodictizine (**10**).^[^ ^31^ ^]^ The ^1^H‐NMR, ^13^C‐NMR, and other spectroscopic data of **9** and **10** perfectly matched those of the natural products; the exception was the specific rotation of **10**. As no denudatine alkaloids having a main framework with opposite absolute stereochemistry have been reported thus far, we suspect that the reported specific rotation of the natural sample was affected by impurities (see Supporting Information for the details).

## Conclusion

In summary, we have developed a divergent synthetic route to denudatine alkaloids, which allowed us to synthesize the proposed structure of (–)‐acochlearine (**7**) and achieve the first asymmetric total syntheses of four denudatine alkaloids, (–)‐cochlearenine (**4**), (–)‐macrocentrine (**8**), (–)‐dictizine (**9**), and (–)‐15‐veratroyl‐17‐acetyl‐19‐oxodictizine (**10**). Our strategy relies on the syntheses of highly congested hexacyclic core frameworks with carefully controlled oxidation states by utilizing three key reactions: the acid promoted intramolecular Mannich reaction and two‐fold Diels–Alder reactions. The late‐stage chemo‐ and stereo‐controlled functional group manipulations diversify the hexacyclic core frameworks into the four denudatine alkaloids. Structural reassignment of (–)‐acochlearine (**7**) and further syntheses of the denudatine alkaloids based on our strategy are ongoing in our laboratory.

## Conflict of Interests

The authors declare no conflict of interest.

## Supporting information



Supporting Information

## Data Availability

The data supporting the findings of this study are available in the Supporting Information of this article.
