# Supplementary material for: Divergent Total Synthesis of Denudatine Alkaloids Cochlearenine, Macrocentrine, Dictizine, 15‐Veratroyl‐17‐Acetyl‐19‐Oxodictizine, and the Proposed Structure of Acochlearine
Source: Angew Chem Int Ed Engl. 2025 Dec 4;65(4):e21481. doi: 10.1002/anie.202521481 (PMC12828479; doi:10.1002/anie.202521481)

Supporting Information for

Divergent Total Synthesis of Denudatine Alkaloids Cochlearenine, Macrocentrine, Dictizine, 15-Veratroyl-17-Acetyl-19-Oxodictizine, and the Proposed Structure of Acochlearine

Shun Kawano, Naoya Miyamoto, Kosuke Fujioka, Juri Sakata, and Hidetoshi Tokuyama*

Graduate School of Pharmaceutical Sciences, Tohoku University, Aoba 6-3, Aramaki, Aoba-ku, Sendai

980-8578, Japan

Contents

General Remarks S1

Experimental Procedures S2

Computational Experiments S48

References S58

^{1}H- and ^{13}C-NMR Spectra S59

General Remarks

All moisture or air sensitive reactions were carried out under a positive atmosphere of argon in dried glassware. Materials were obtained from commercial suppliers and used without further purification unless otherwise mentioned. MeOH, EtOH, DMF, DME, Et_3N, toluene, and pyridine were dried and distilled according to the standard protocols. Anhydrous THF, MeCN, and CH_2Cl_2 were purchased from commercial suppliers. Flash column chromatography was performed on Silica Gel 60N (spherical neutral, 40–50 μm) using the indicated solvent. Preparative TLC and analytical TLC were performed on Merck 60 F254 glass plates precoated with a 0.25 mm thickness of silica gel. Automated MPLC purification (column chromatography) was conducted by Biotage Isolera One ACITM Spektra. NMR spectra were recorded on a 400 MHz and 600 MHz spectrometer. Chemical shifts for ^{1}H-NMR are reported in parts per million (ppm) downfield from tetramethylsilane (0 ppm) as the internal standard and coupling constants are in Hertz (Hz). The following abbreviations are used for spin multiplicity: s = singlet, d = doublet, t = triplet, m = multiplet, and br = broad. Chemical shifts for ^{13}C-NMR are reported in ppm, relative to the central line of a triplet at 77.0 ppm for $CDCl_3$, a quintet at 49.0 ppm for CD_3OD and a triplet at 123.5 ppm for C_5D_5N. NMR spectra were recorded on JNM-AL400 spectrometer or a JEOL ECA600 spectrometer. IR spectra were measured on a FT-IR-4100 spectrometer. Mass spectra were recorded on a BRUKER micrOTOF II spectrometer and a JEOL JMS-700 spectrometer. Optical rotation was measured on a JASCO P-2200 polarimeter. All melting points were determined on a Yanoco micromelting point apparatus MP-500P.

Experimental Procedure

Enamine 28

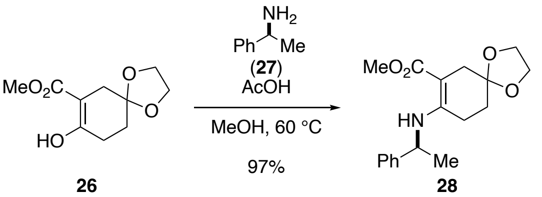

To a solution of ester **26** (72.6 g, 339 mmol) and (*S*)-(–)-1-phenylethylamine (**27**) (49.6 mL, 356 mmol) in MeOH (170 mL) was added AcOH (2.91 mL, 50.9 mmol) at room temperature. After stirring for 2 h at 60 °C, the solvent was removed under reduced pressure. The residue was passed through a short column of silica gel using 9% EtOAc in hexane as an eluent and the eluate was concentrated under reduced pressure to give a crude material. The crude material was purified by the recrystallization from EtOAc to afford chiral enamine **28** (104 g, 328 mmol, 97%). A white solid; R_f = 0.30 (hexane-EtOAc = 3:1); mp: 71–73 °C (EtOAc); $[\alpha]_D^{18}$ = +308 (*c* 1.00, $CHCl_3$); IR (film): 3269, 2936, 2872, 1647, 1596, 1447, 1367, 1235, 1206, 1054 cm^{-1}; ^{1}H-NMR (400 MHz, $CDCl_3$): δ 9.42 (1H, d, *J* = 6.8 Hz), 7.33 (2H, dd, *J* = 7.6, 7.6 Hz), 7.27–7.22 (3H, m), 4.62 (1H, dq, *J* = 6.8, 6.8 Hz), 4.00–3.88 (4H, m), 3.69 (3H, s), 2.59–2.46 (3H, m), 2.24 (1H, ddd, *J* = 17.6, 6.0, 6.0 Hz), 1.73–1.59 (2H, m), 1.49 (3H, d, *J* = 6.8 Hz); ^{13}C-NMR (100 MHz, $CDCl_3$): δ 169.8, 157.3, 144.8, 128.1 (2C), 126.3, 124.7 (2C), 106.6, 86.9, 63.62, 63.58, 51.6, 49.6, 33.2, 29.5, 24.8, 24.6; HRMS (ESI) *m/z*: $[M+H]^+$ Calcd for $C_{18}H_{24}NO_4$ 318.1700, Found 318.1709.

Ester 25

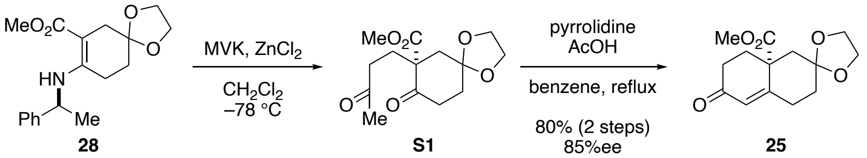

To a solution of chiral enamine **28** (52.0 g, 164 mmol) and $ZnCl_2$ (69.5 g, 510 mmol) in CH_2Cl_2 (340 mL) was added dropwise methyl vinyl ketone (42.6 mL, 510 mmol) at –78 °C over 2.5 h. After stirring for 3.5 h at –78 °C, the reaction was quenched with 10% aq. AcOH at –78 °C, and the mixture was extracted with CH_2Cl_2 three times. The combined organic extracts were washed with brine, dried over anhydrous sodium sulfate, and filtered. The filtrate was concentrated under reduced pressure to remove organic solvents to give a residue, which was passed through a short column of silica gel using 25 to 50% EtOAc in hexane as an eluent. The eluate was concentrated under reduced pressure to give a crude **S1**, which was used for the next reaction without further purification. To a solution of the crude **S1** and pyrrolidine (1.35 mL, 16.4 mmol) in benzene (164 mL) was added AcOH (938 μL, 16.4 mmol) at room temperature. After stirring for 3 h at 120 °C, the solvent was removed under reduced pressure to give a crude material, which was purified by a silica gel column chromatography (hexane-EtOAc = 3:1 to 3:2) to afford ester **25** (36.3 g, 136 mmol, 80%, 2 steps). A white solid; The enantiomeric excess of **25** was determined by HPLC (DAICEL-CHIRALCEL OD-H, *n*-hexane-*i*-PrOH = 50:50, flow rate = 0.600 mL/min, retention times: t_R = 9.66 min, t_S = 11.9 min, 85% ee); R_f = 0.59 (EtOAc only);

mp: 122–124 °C (hexane-EtOAc); $[\alpha]_D^{15}$ = –123 (*c* 2.10, $CHCl_3$); IR (film): 2950, 2892, 1728, 1673, 1453, 1442, 1296, 1226, 1167, 1074, 710 cm^{-1}; ^{1}H-NMR (400 MHz, $CDCl_3$): δ 5.99 (1H, s), 4.05–3.86 (4H, m), 3.74 (3H, s), 2.92 (1H, dddd, *J* = 15.6, 14.4, 6.0, 2.4 Hz), 2.62 (1H, dd, *J* = 14.4, 3.2 Hz), 2.49 (1H, ddd, *J* = 15.6, 4.8, 2.4 Hz), 2.38–2.32 (2H, m), 2.21 (1H, ddd, *J* = 14.0, 14.0, 4.0 Hz), 1.97–1.74 (3H, m), 1.52 (1H, d, *J* = 14.0 Hz); ^{13}C-NMR (150 MHz, $CDCl_3$): δ 197.2, 173.1, 159.6, 126.5, 106.3, 64.0, 63.7, 51.9, 47.7, 43.2, 35.0, 34.2, 33.8, 31.0; HRMS (ESI) *m/z*: $[M+H]^+$ Calcd for $C_{14}H_{19}O_5$ 267.1237, Found 267.1230.

*Enantiomeric excess and reproducibility were improved by the modification of the original protocol established by Costa and co-workers.[1]

Ester 25 (racemic)

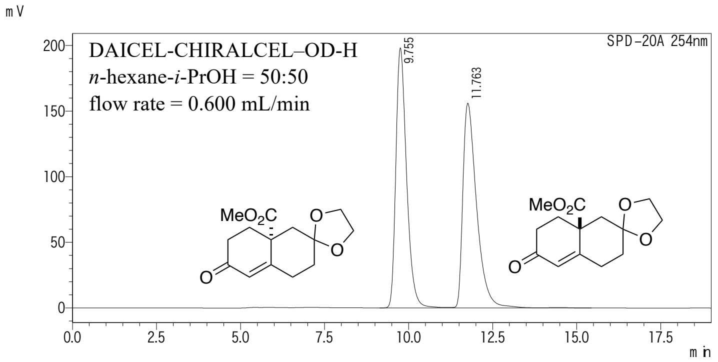

Peak	Time	Area	Height	Area %
1	9.755	4395668	197191	49.688
2	11.763	4450864	155945	50.312
total		8846532	353136	100

Ester 25 (85% ee)

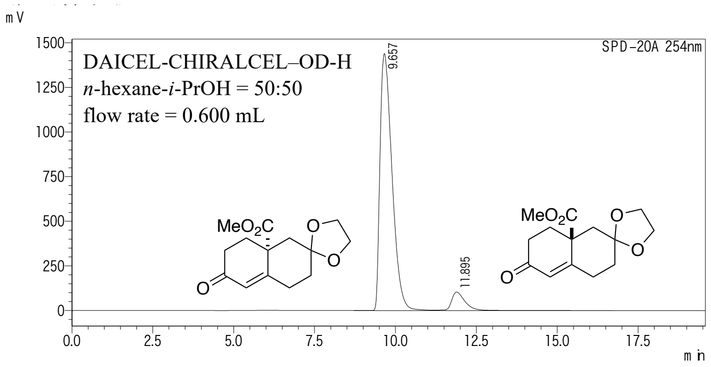

Peak	Time	Area	Height	Area %
1	9.657	37308174	1425468	92.396
2	11.895	3070470	103591	7.604
total		40378644	1529059	100

Ester 25 (>99% ee)

Ester **25** (36.3 g, 136 mmol, 85% ee) was recrystallized from hexane/EtOAc to afford optically pure **25** (27.2 g, 102 mmol, 75%, >99% ee). $[\alpha]_D^{15}$ = –148 (*c* 2.40, $CHCl_3$); The enantiomeric excess of **25** was determined by HPLC (DAICEL-CHIRALCEL OD-H, *n*-hexane-*i*-PrOH = 50:50, flow rate = 0.600 mL/min, retention times: t_R = 9.68 min, t_S = 12.0 min, >99% ee).

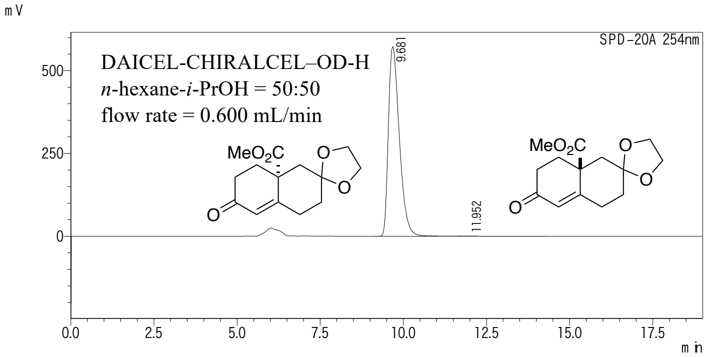

Peak	Time	Area	Height	Area %
1	9.681	13171759	571518	99.940
2	11.952	7856	340	0.060
total		13179615	571858	100

Diol S2

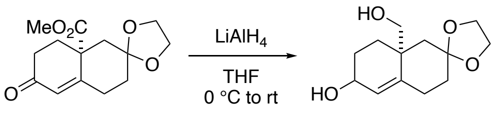

25 95% **S2**

To a suspension of $LiAlH_4$ (18.2 g, 480 mmol) in THF (768 mL) was added ester **25** (51.0 g, 192 mmol) in THF (384 mL) at 0 °C. After stirring for 1.5 h at room temperature, the reaction was quenched by slow addition of H_2O (18.2 mL), 15% aqueous NaOH (18.2 mL), and H_2O (54.6 mL) at 0 °C. The resulting suspension was filtered through a pad of Celite and the filter cake was washed with EtOAc. The combined filtrate and washings were concentrated under reduced pressure to give a crude material, which was purified by silica gel column chromatography (hexane-EtOAc = 1:2 to 100% EtOAc, then EtOAc-MeOH = 10:1) to afford diol **S2** (44.0 g, 183 mmol, 95%). A white solid; R_f = 0.23 (hexane-EtOAc = 1:3); mp: 115–117 °C (hexane–CH_2Cl_2); $[\alpha]_D^{18}$ = –50.4 (*c* 1.00, $CHCl_3$); IR (film): 2930, 2872, 1654, 1447, 1354, 1128, 1032, 931, 849, 713 cm^{-1}; ^1H-NMR (400 MHz, $CDCl_3$): δ 5.59 (1H, s), 4.23–4.21 (1H, m), 4.06–3.89 (4H, m), 3.79 (1H, dd, *J* = 11.6, 6.0 Hz), 3.57 (1H, dd, *J* = 11.6, 7.6 Hz), 2.52–2.44 (1H, m), 2.22–2.19 (1H, m), 2.11 (1H, ddd, *J* = 14.4, 4.8, 2.4 Hz), 2.01 (1H, dd, *J* = 14.0, 2.8 Hz), 1.94–1.83 (3H, m), 1.59–1.46 (3H, m), 1.37 (1H, d, *J* = 14.0 Hz), 1.21–1.14 (1H, m); ^{13}C-NMR (150 MHz, $CDCl_3$): δ 140.3, 127.9, 108.7, 66.7, 65.1, 64.7, 63.6, 43.3, 40.2, 35.5, 31.3, 29.8, 28.1; HRMS (ESI) *m/z*: $[M+Na]^+$ Calcd for $C_{13}H_{20}NaO_4$ 263.1254, Found 263.1263.

Enone 29

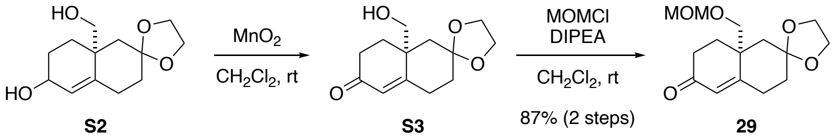

To a solution of diol **S2** (44.0 g, 183 mmol) in CH_2Cl_2 (370 mL) was added MnO_2 (159 g, 1.83 mol) at room temperature. After stirring for 12 h at room temperature, the reaction mixture was filtered through a pad of Celite and the filter cake was washed with CH_2Cl_2 and MeOH. The combined filtrate and washings were concentrated under reduced pressure to give a crude **S3**, which was used for the next reaction without further purification. To a solution of the crude **S3** and DIPEA (98.0 mL, 549 mmol) in CH_2Cl_2 (180 mL) was added MOMCl (27.8 mL, 366 mmol) at room temperature. After stirring for 11 h at room temperature, the reaction was quenched with H_2O at the same temperature, and the mixture was extracted with CH_2Cl_2 three times. The combined organic extracts were washed with brine, dried over anhydrous sodium sulfate, and filtered. The filtrate was concentrated under reduced pressure to give a crude material, which was purified by automated column chromatography (gradient: 10–60% EtOAc in hexane) to afford enone **29** (44.9 g, 159 mmol, 87%, 2 steps). A white solid; R_f = 0.63 (EtOAc); mp: 87–88 °C (hexane-EtOAc); $[\alpha]_D^{18}$ = –14.4 (*c* 1.00, $CHCl_3$); IR (film): 2943, 2885, 1670, 1442, 1332, 1228, 1109, 1074, 1041, 941 cm^{-1}; ^{1}H-NMR (400 MHz, $CDCl_3$): δ 5.91 (1H, d, *J* =2.0 Hz), 4.66 (1H, d, *J* = 6.8 Hz), 4.61 (1H, d, *J* = 6.8 Hz) 4.02–3.91 (4H, m), 3.81 (1H, d, *J* = 9.6 Hz), 3.72 (1H, d, *J* = 9.6 Hz), 3.36 (3H, s), 2.70–2.54 (2H, m), 2.38–2.18 (4H, m), 1.92 (1H, dddd, *J* = 13.2, 5.6, 2.8, 2.8 Hz), 1.76–1.65 (2H, m), 1.42 (1H, d, *J* = 14.0 Hz); ^{13}C-NMR (150 MHz, $CDCl_3$): δ 199.0, 163.5, 126.8, 107.5, 96.5, 68.7, 64.5, 63.6, 55.0, 42.0, 40.5, 34.6, 33.5, 33.3, 30.5; HRMS (ESI) *m/z*: $[M+Na]^+$ Calcd for $C_{15}H_{22}NaO_5$ 305.1359, Found 305.1347.

Ester 30

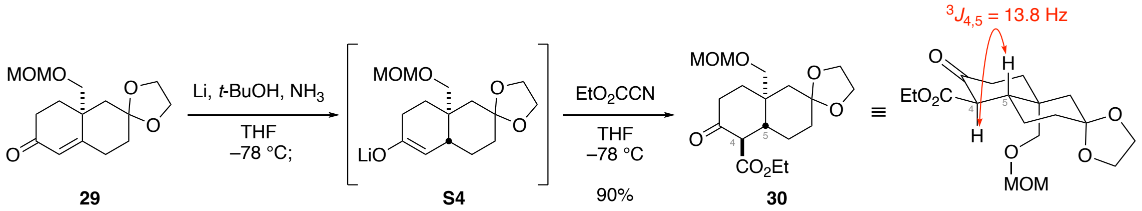

To a solution of lithium (2.77 g, 398 mmol) in liquid ammonia (295 mL) were added enone **29** (22.5 g, 79.7 mmol) and *t*-BuOH (6.82 mL, 71.9 mmol) in THF (100 mL) at –78 °C. After stirring for 1 h at –78 °C, the reaction was quenched with isoprene at –78 °C. The mixture was gently warmed to room temperature to remove ammonia, and then other volatile materials were removed under reduced pressure to give lithium enolate **S4**. To a suspension of the lithium enolate **S4** in THF (295 mL) was added dropwise ethyl cyanoformate (11.8 mL, 120 mmol) at –78 °C over 10 min. After stirring for 1 h at –78 °C, the reaction was quenched with H_2O, and the mixture was extracted with CH_2Cl_2 three times. The combined organic extracts were washed with brine, dried over anhydrous sodium sulfate, and filtered. The filtrate was concentrated under reduced pressure to remove organic solvents to give a crude material, which was purified by flash silica gel column chromatography (hexane-EtOAc = 2:1) to afford ester **30** (25.7 g, 72.1 mmol, 90%). A white solid; R_f = 0.64 (hexane-EtOAc = 1:3); mp: 109–112 °C (Et_2O-CH_2Cl_2-hexane); $[\alpha]_D^{18}$ = +30.8 (*c* 0.880, $CHCl_3$); IR (film): 2946, 2878, 1740,

1712, 1451, 1373, 1254, 1161, 1044, 941 cm^{-1}; ^{1}H-NMR (600 MHz, C_6D_6): δ 4.51 (1H, d, J = 7.2 Hz), 4.41 (1H, d, J = 7.2 Hz), 4.20–4.14 (1H, m), 4.13–4.07 (1H, m), 3.94 (1H, d, J = 9.6 Hz), 3.56 (1H, d, J = 9.6 Hz), 3.49–3.42 (4H, m), 3.28 (1H, d, J = 13.8 Hz), 3.13 (3H, s), 2.27 (1H, ddd, J = 15.0, 13.8, 6.6 Hz), 2.20 (1H, ddd, J = 15.0, 5.4, 2.4 Hz), 2.13–2.08 (2H, m), 1.91 (1H, ddd, J = 13.8, 6.6, 2.4 Hz), 1.73 (1H, dddd, J = 13.8, 3.0, 3.0, 3.0 Hz), 1.53 (1H, ddd, J = 13.8, 13.8, 4.8 Hz), 1.54–1.50 (1H, m), 1.44 (1H, dddd, J = 13.8, 13.8, 13.8, 3.0 Hz), 1.07–1.05 (4H, m), 0.89 (1H, ddd, J = 13.8, 13.8, 5.4 Hz); ^{13}C-NMR (150 MHz, $CDCl_3$): δ 205.2, 169.6, 108.0, 96.7, 65.1, 64.4, 63.4, 60.7, 58.9, 55.0, 45.2, 42.1, 36.9, 34.6, 34.5, 24.1, 13.9 (One signal is missing due to overlap); HRMS (ESI) *m/z*: [M+Na]$^+$ Calcd for $C_{18}H_{28}NaO_7$ 379.1727, Found 379.1718.

*The stereochemistries at C4 and C5 were determined by the coupling constant between H4 and H5.

1,3-Diol 31

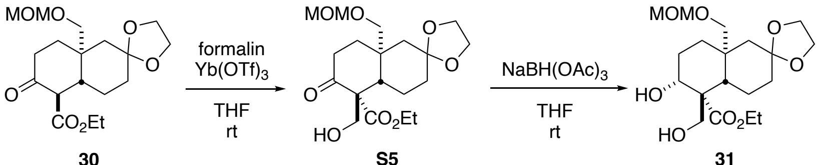

30 **S5** **31**

To a solution of ester **30** (25.7 g, 72.1 mmol) and $Yb(OTf)_3$ (4.47 g, 7.21 mmol) in THF (144 mL) was added formalin (35% in H_2O, 58.5 mL, 721 mmol) at room temperature. After stirring for 24 h at room temperature, the reaction was quenched with H_2O at the same temperature, and the mixture was extracted with CH_2Cl_2 three times. The combined organic extracts were washed with brine, dried over anhydrous sodium sulfate, and filtered. The filtrate was concentrated under reduced pressure to remove organic solvents. The residue was filtered through a short column of silica gel using 25 to 50% EtOAc in hexane as eluents. The eluate was concentrated under reduced pressure to give a crude **S5**, which was used for the next reaction without further purification. To a solution of crude **S5** in THF (130 mL) was added $NaBH(OAc)_3$ (41.3 g, 195 mmol) at room temperature. After stirring for 24 h at room temperature, the reaction was quenched with saturated aqueous $NaHCO_3$ at the same temperature, and the mixture was extracted with CH_2Cl_2 five times. The combined organic extracts were washed with brine, dried over anhydrous sodium sulfate, and filtered. The filtrate was concentrated under reduced pressure to give a crude material, which was purified by flash silica gel column chromatography (hexane-EtOAc = 2:1) to afford 1,3-diol **31** (22.3 g, 57.4 mmol, 80%, 2 steps). A white solid; R_f = 0.40 (100% EtOAc); mp: 108–111 °C (CH_2Cl_2-hexane); $[\alpha]_D^{18}$ = +6.80 (c 3.01, $CHCl_3$); IR (film): 3449, 2952, 2882, 1696, 1451, 1231, 1138, 1109, 1041, 909 cm^{-1}; ^{1}H-NMR (400 MHz, $CDCl_3$): δ 4.60 (1H, d, J = 6.4 Hz), 4.56 (1H, d, J = 6.4 Hz), 4.30–4.18 (3H, m), 3.97–3.69 (8H, m), 3.59 (1H, ddd, J = 14.0, 14.0, 4.8 Hz), 3.39 (1H, dd, J = 9.2, 1.2 Hz), 3.34 (3H, s), 2.21 (1H, dd, J = 14.0, 2.8 Hz), 2.11–1.76 (6H, m), 1.53–1.46 (1H, m), 1.35 (3H, t, J = 7.2 Hz), 1.28 (1H, dd, J = 12.8, 2.8 Hz), 1.00–0.92 (2H, m); ^{13}C-NMR (150 MHz, $CDCl_3$): δ 175.3, 108.1, 96.6, 77.6, 69.0, 64.6, 64.4, 63.5, 61.1, 54.9, 53.2, 46.9, 42.6, 38.6, 36.1, 34.5, 27.8, 21.0, 13.9; HRMS (ESI) *m/z*: [M+Na]$^+$ Calcd for $C_{19}H_{32}NaO_8$ 411.1989, Found 411.2001.

Lactone 34

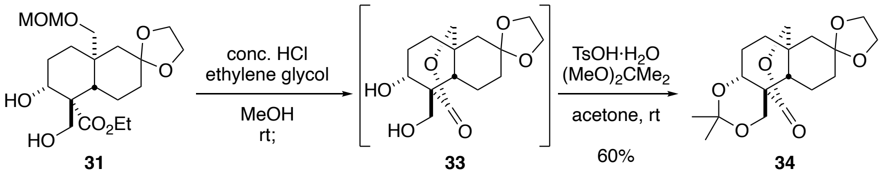

To a solution of 1,3-diol **31** (22.3 g, 57.4 mmol) and ethylene glycol (12.8 mL, 230 mmol) in MeOH (115 mL) was added conc. HCl (12 M in H_2O, 2.39 mL, 28.7 mmol) at room temperature. After stirring for 21 h at room temperature, the solvent was removed under reduced pressure. Trace amounts of H_2O were completely removed by azeotropic co-evaporation using toluene (3 × 10 mL) to give diol **33**. To a solution of **33** in acetone (190 mL) and 2,2-dimethoxypropane (70.3 mL, 574 mmol) was added TsOH·H_2O (1.09 g, 5.74 mmol) at room temperature. After stirring for 1.5 h at room temperature, the reaction was quenched with saturated aqueous $NaHCO_3$ at the same temperature, and the mixture was extracted with EtOAc three times. The combined organic extracts were washed with brine, dried over anhydrous sodium sulfate, and filtered. The filtrate was concentrated under reduced pressure to give a crude material, which was purified by flash silica gel column chromatography (hexane-acetone = 3:1) to afford lactone **34** (11.6 g, 34.3 mmol, 60%). A white solid; R_f = 0.47 (100% EtOAc); mp: 178–180 °C (EtOAc); $[\alpha]_D^{18}$ = +35.1 (*c* 1.30, $CHCl_3$); IR (film): 3005, 2950, 2878, 1721, 1454, 1367, 1260, 1157, 1113, 951, 748 cm^{-1}; ^{1}H-NMR (400 MHz, $CDCl_3$): δ 5.16 (1H, dd, *J* = 8.4, 2.8 Hz), 4.24 (1H, d, *J* = 11.6 Hz), 3.96–3.86 (6H, m), 3.63 (1H, d, *J* = 11.6 Hz), 1.92–1.79 (4H, m), 1.61–1.59 (1H, m), 1.55–1.47 (12H, m); ^{13}C-NMR (150 MHz, $CDCl_3$): δ 169.8, 107.3, 99.1, 74.3, 73.6, 65.0, 64.7, 63.7, 45.1, 44.6, 42.7, 40.0, 34.7, 34.2, 29.1, 26.6, 20.4, 19.2; HRMS (ESI) *m/z*: $[M+Na]^+$ Calcd for $C_{18}H_{26}NaO_6$ 361.1622, Found 361.1619.

Amide 35

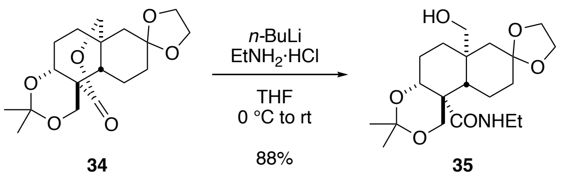

To a suspension of $EtNH_2$·HCl (4.57 g, 56.0 mmol) in THF (37 mL) was added *n*-BuLi (2.40 M in *n*-hexane, 42.0 mL, 101 mmol) at 0 °C. After stirring for 50 min at room temperature, a THF (37 mL) solution of lactone **34** (3.80 g, 11.2 mmol) was added at 0 °C. After stirring for 30 min at room temperature, the reaction was quenched with H_2O at 0 °C, and the mixture was extracted with CH_2Cl_2 five times. The combined organic extracts were washed with brine, dried over anhydrous sodium sulfate, and filtered. The filtrate was concentrated under reduced pressure to give a crude material, which was purified by flash silica gel column chromatography (hexane-acetone = 2:1 to 3:2) to afford amide **35** (3.80 g, 9.91 mmol, 88%). A white foam; R_f = 0.17 (hexane-acetone = 2:1); $[\alpha]_D^{17}$ = –13.4 (*c* 3.00, $CHCl_3$); IR (film): 3384, 2988, 2943, 2878, 1654, 1531, 1202, 1099, 1051, 758 cm^{-1}; ^{1}H-NMR (600 MHz, $CDCl_3$): δ 7.35 (1H, br s), 4.13 (1H, d, *J* = 10.8 Hz), 4.01–3.97 (2H, m), 3.95–3.89 (2H, m), 3.86–3.83 (1H, m), 3.75 (1H, dd, *J* = 12.6, 4.2 Hz), 3.59 (1H, d, *J* = 10.8 Hz), 3.42–3.35 (1H, m), 3.28–3.22 (1H, m), 2.54 (1H, dddd, *J* = 14.4, 14.4, 14.4, 4.2 Hz), 2.16–2.05 (3H, m), 1.92–1.87 (2H,

m), 1.62–1.57 (2H, m), 1.49 (3H, s), 1.42 (3H, s), 1.33–1.24 (2H, m), 1.18 (3H, t, J = 7.2 Hz), 1.14 (1H, d, J = 14.4 Hz), 1.08–1.03 (1H, m); ^{13}C-NMR (150 MHz, $CDCl_3$): δ 172.9, 108.5, 100.0, 76.5, 70.8, 64.7, 63.6, 60.7, 49.4, 46.1, 44.3, 40.2, 35.1, 34.8, 34.0, 29.7, 24.6, 20.8, 18.6, 14.6; HRMS (ESI) *m/z*: $[M+Na]^+$ Calcd for $C_{20}H_{33}NNaO_6$ 406.2200, Found 406.2193.

Hemiaminal 37

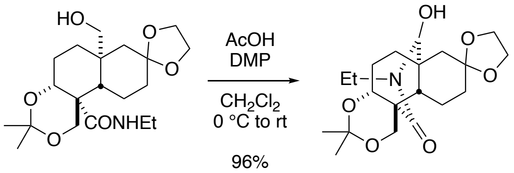

35 37

To a solution of amide **35** (5.29 g, 13.7 mmol) and AcOH (1.58 mL, 27.6 mmol) in CH_2Cl_2 (138 mL) was added Dess–Martin periodinane (8.78 g, 20.7 mmol) at 0 °C. After stirring for 2 h at room temperature, the reaction was quenched with saturated aqueous $Na_2S_2O_3$ at 0 °C, and the mixture was extracted with CH_2Cl_2 three times. The combined organic extracts were washed with brine, dried over anhydrous sodium sulfate, and filtered. The filtrate was concentrated under reduced pressure to remove organic solvent to give a crude material, which was purified by flash silica gel column chromatography (hexane-acetone = 2:1 to 1:1) to afford hemiaminal **37** (5.04 g, 13.2 mmol, 96%). A white solid; R_f = 0.26 (hexane-acetone = 1:1); mp: 248–251 °C (MeOH-CH_2Cl_2-hexane) $[\alpha]_D^{18}$ = +9.44 (*c* 2.00, MeOH); IR (film): 3272, 2943, 2872, 1631, 1538, 1505, 1454, 1257, 1099, 1048 cm^{-1}; ^{1}H-NMR (400 MHz, CD_3OD): δ 5.61 (1H, s), 4.05 (1H, d, J = 11.6 Hz), 4.01–3.92 (3H, m), 3.87–3.84 (2H, m), 3.66 (1H, d, J = 11.6 Hz), 3.56 (1H, dq, J = 14.4, 7.2 Hz), 3.44 (1H, dq, J = 14.4, 7.2 Hz), 2.27 (1H, dddd, J = 13.2, 2.8, 2.8, 2.8 Hz), 1.93 (1H, dd, J = 14.4, 2.8 Hz), 1.82 (1H, dddd, J = 13.2, 2.8, 2.8, 2.8 Hz), 1.73 (1H, dddd, J = 13.2, 13.2, 13.2, 4.4 Hz), 1.62–1.57 (1H, m), 1.55–1.44 (6H, m), 1.37 (3H, s), 1.26–1.17 (2H, m), 1.16–1.08 (1H, m), 1.13 (3H, t, J = 7.2 Hz); ^{13}C-NMR (150 MHz, CD_3OD): δ 171.1, 109.4, 100.1, 80.4, 76.0, 66.6, 65.7, 64.6, 47.0, 45.2, 43.4, 39.9, 38.6, 35.6, 34.1, 29.6, 27.3, 20.7, 19.6, 12.5; HRMS (ESI) *m/z*: $[M+H]^+$ Calcd for $C_{20}H_{32}NO_6$ 382.2224, Found 382.2215.

Ketone 38

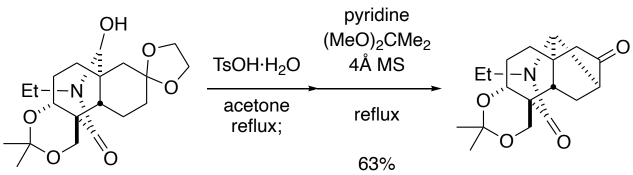

37 38

To a solution of hemiaminal **37** (5.00 g, 13.1 mmol) in acetone (262 mL) was added $TsOH{\cdot}H_2O$ (2.49 g, 13.1 mmol) at room temperature. After stirring for 16.5 h at 86 °C, pyridine (949 μL, 13.1 mmol) was added at room temperature, and the mixture was stirred for 2 h at the same temperature. To the resulting mixture were added 4Å MS (30.0 g) and 2,2-dimethoxypropane (8.03 mL, 65.5 mmol) at the same temperature, and then the mixture was allowed to warm up to 86 °C and stirred for 15 h. The resulting suspension was filtered through a pad of basic alumina and the filter cake was washed with acetone. The combined filtrate and washings were

concentrated under reduced pressure to give a crude material, which was purified by flash silica gel column chromatography (hexane-acetone = 1:1) to afford ketone **38** (2.64 g, 8.27 mmol, 63%). A white solid; R_f = 0.31 (hexane-acetone = 1:1); mp: 166–169 °C (hexane-EtOAc); $[\alpha]_D^{17}$ = –6.65 (*c* 9.60, $CHCl_3$); IR (film): 2991, 2943, 1754, 1651, 1457, 1377, 1257, 1200, 1093, 751 cm^{-1}; ^{1}H-NMR (400 MHz, $CDCl_3$): δ 4.37 (1H, d, *J* = 12.0 Hz), 3.82 (1H, dd, *J* = 12.0, 4.4 Hz), 3.66 (1H, dq, *J* = 14.0, 6.8 Hz), 3.55 (1H, d, *J* = 12.0 Hz), 3.38 (1H, s), 3.22 (1H dq, *J* = 14.0, 6.8 Hz), 2.70 (1H, d, *J* = 5.6 Hz), 2.19–2.06 (3H, m), 1.97–1.71 (6H, m), 1.54 (3H, s), 1.43 (3H, s), 1.17 (3H, t, *J* = 6.8 Hz); ^{13}C-NMR (100 MHz, $CDCl_3$): δ 211.0, 168.4, 99.7, 72.0, 67.2, 64.3, 55.6, 48.8, 48.5, 43.8, 43.5, 41.6, 27.8, 26.5, 26.1, 25.9, 21.7, 13.3; HRMS (ESI) *m/z*: $[M+Na]^+$ Calcd for $C_{18}H_{25}NNaO_4$ 342.1676, Found 342.1677.

Enol triflate 39

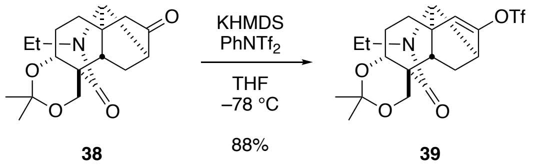

To a solution of ketone **38** (1.25 g, 3.91 mmol) and $PhNTf_2$ (3.49 g, 9.78 mmol) in THF (39 mL) was added KHMDS (0.5 M in toluene, 19.6 mL, 9.78 mmol) at –78 °C. After stirring for 1.5 h at –78 °C, the reaction was quenched with H_2O at the same temperature, and the temperature was allowed to raise to room temperature. Then, the mixture was extracted with CH_2Cl_2 three times. The combined organic extracts were washed with brine, dried over anhydrous sodium sulfate, and filtered. The filtrate was concentrated under reduced pressure to remove organic solvents to give a crude material, which was purified by flash silica gel column chromatography (hexane-EtOAc = 1:1 to 1:2) to afford enol triflate **39** (1.59 g, 3.56 mmol, 88%). A colorless oil; R_f = 0.53 (hexane-EtOAc = 1:2); $[\alpha]_D^{18}$ = –4.10 (*c* 2.00, $CHCl_3$); IR (film): 2988, 2943, 1657, 1457, 1421, 1212, 1138, 1077, 957, 819 cm^{-1}; ^{1}H-NMR (600 MHz, $CDCl_3$): δ 5.45 (1H, d, *J* = 2.4 Hz), 4.32 (1H, d, *J* = 12.0 Hz), 3.85 (1H, dd, *J* = 12.0, 3.6 Hz), 3.61 (1H, d, *J* = 12.0 Hz), 3.52 (1H, dq, *J* = 14.4, 7.2 Hz), 3.24 (1H, dq, *J* = 14.4, 7.2 Hz), 3.18 (1H, d, *J* = 1.8 Hz), 2.89–2.88 (1H, m), 2.29 (1H, ddd, *J* = 14.4, 6.0, 1.2 Hz), 2.09 (1H, ddd, *J* = 14.4, 14.4, 6.0 Hz), 1.82–1.79 (3H, m), 1.70–1.62 (2H, m), 1.52 (3H, s), 1.43 (3H, s), 1.14 (3H, t, *J* = 7.2 Hz); ^{13}C-NMR (150 MHz, $CDCl_3$): δ 168.7, 153.5, 121.1, 118.4 (q, ^1J = 320 kHz), 99.4, 74.3, 72.0, 65.5, 49.9, 48.4, 48.0, 42.1, 41.2, 29.5, 26.9, 26.7, 25.9, 21.3, 13.4; HRMS (ESI) *m/z*: $[M+H]^+$ Calcd for $C_{19}H_{25}F_3NO_6S$ 452.1349, Found 452.1350.

Ketol 41

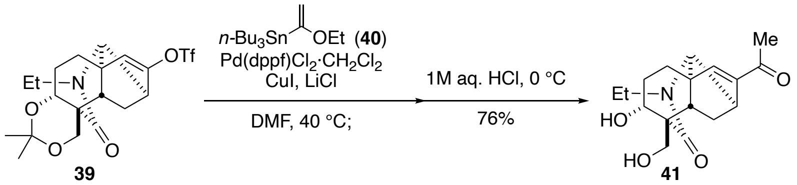

To a solution of enol triflate **39** (1.56 g, 3.46 mmol), $Pd(dppf)Cl_2·CH_2Cl_2$ (283 mg, 0.346 mmol), CuI (65.9

mg, 0.346 mmol), and LiCl (733 mg, 17.3 mmol) in DMF (35 mL) was added tributyl(1-ethoxyvinyl)tin (**40**) (1.38 mL, 4.15 mmol) at room temperature. The reaction mixture was degassed by three freeze-pump-thaw cycles, and the reaction mixture was stirred at 40 °C for 2.5 h. The reaction was quenched by addition of 1M aq. HCl (21 mL) at 0 °C. After stirring for 2.5 h at 0 °C, the reaction mixture was saturated by excess amount of $NaHCO_3$ and NaCl. The resulting suspension was passed through a pad of Celite and the filter cake was washed with $CHCl_3$. The filtrate was extracted with $CHCl_3$ five times. The combined organic extracts were washed with brine, dried over anhydrous sodium sulfate, and filtered. The filtrate was concentrated under reduced pressure to remove organic solvents to give a crude material, which was purified by flash silica gel column chromatography (100% EtOAc to EtOAc-MeOH = 9:1) to afford ketol **41** (800 mg, 2.62 mmol, 76%). A white foam; R_f = 0.36 (EtOAc-MeOH = 9:1); $[\alpha]_D^{18}$ = –12.1 (*c* 1.60, $CHCl_3$); IR (film): 3439, 2946, 2878, 1663, 1622, 1457, 1377, 1228, 1058, 755 cm^{-1}; ^{1}H-NMR (400 MHz, $CDCl_3$): δ 6.48 (1H, d, *J* = 2.0 Hz), 4.37 (1H, dd, *J* = 11.2, 2.4 Hz), 3.98 (1H, d, *J* = 6.0 Hz), 3.90–3.84 (1H, m), 3.72–3.62 (2H, m), 3.61 (1H, dq, *J* = 14.8, 8.0 Hz), 3.26–3.24 (1H, m), 3.14 (1H, dq, *J* = 14.8, 8.0 Hz), 3.08 (1H, s), 2.27 (1H, dd, *J* = 14.4, 6.0 Hz), 2.25 (3H, s), 2.10 (1H, dd, *J* = 14.4, 6.0 Hz), 2.04–1.99 (1H, m), 1.84 (1H, ddd, *J* = 13.2, 4.4, 2.0 Hz), 1.70 (1H, ddd, *J* = 7.6, 2.0, 2.0 Hz), 1.57–1.45 (2H, m), 1.15 (3H, t, *J* = 8.0 Hz); ^{13}C-NMR (100 MHz, $CDCl_3$): δ 194.2, 171.0, 149.7, 146.6, 74.8, 72.9, 65.4, 52.8, 51.3, 45.2, 41.0, 38.7, 31.2, 28.6, 26.1, 25.3, 13.3; HRMS (EI) *m/z*: $[M]^+$ Calcd for $C_{17}H_{23}NO_4$ 305.1622, Found 305.1641.

Phenol 45

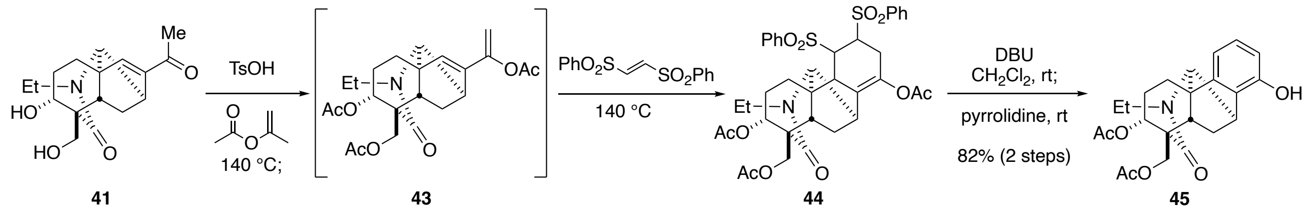

41 **43** **44** (mixture of diastereomers) **45**

To a suspension of 1,3-diol **41** (777 mg, 2.54 mmol) in isopropenyl acetate (20 mL) was added TsOH (131 mg, 0.763 mmol) at room temperature. After stirring for 1 h at 140 °C, *trans*-1,2-bis(phenylsulfonyl)ethylene (1.17 g, 3.81 mmol) was added at room temperature, and the mixture was stirred for 7 h at 140 °C. The solvent was removed under reduced pressure. The residue was roughly purified by flash silica gel column chromatography (hexane-EtOAc = 2:1 to 3:1) to afford a diastereomeric mixture of **44** containing inseparable impurities, which was used for the next reaction without further purification.

To the solution of the crude **44** in CH_2Cl_2 (25 mL) was added DBU (2.27 mL, 15.2 mmol) at room temperature. After stirring for 5 h at the same temperature, pyrrolidine (1.53 mL, 18.6 mmol) was added, and the mixture was stirred for 2 h at room temperature. The reaction was quenched with 2 M aq. HCl at room temperature, and the mixture was extracted with CH_2Cl_2 three times. The combined organic extracts were washed with brine, dried over anhydrous sodium sulfate, and filtered. The filtrate was concentrated under reduced pressure to remove organic solvents to give a crude material, which was purified by flash silica gel column chromatography (hexane-EtOAc = 1:2) to afford phenol **45** (860 mg, 2.08 mmol, 82%, 2 steps). A white

foam; R_f = 0.34 (hexane-EtOAc = 1:2); $[\alpha]_D^{18}$ = –24.5 (*c* 1.00, $CHCl_3$); IR (film): 3281, 2943, 1744, 1625, 1593, 1464, 1370, 1238, 1041, 755 cm^{-1}; ^1H-NMR (600 MHz, $CDCl_3$): δ 7.32 (1H, br s), 7.07 (1H, dd, *J* = 7.8, 7.8 Hz), 6.78 (1H, d, *J* = 7.8 Hz), 6.73 (1H, d, *J* = 7.8 Hz), 5.06 (1H, dd, *J* = 12.0, 4.8 Hz), 4.84 (1H, d, *J* = 11.4 Hz), 4.17 (1H, d, *J* = 11.4 Hz), 3.78 (1H, dq, *J* = 13.8, 6.6 Hz), 3.60 (1H, d, *J* = 4.8 Hz), 3.29 (1H, s), 3.15 (1H, dq, *J* = 13.8, 6.6 Hz), 2.51 (1H, ddd, *J* = 13.8, 13.8, 6.0 Hz), 2.29 (1H, ddd, *J* = 13.8, 6.0, 1.8 Hz), 2.16–2.13 (1H, m), 2.10 (3H, s), 1.99 (3H, s), 1.97 (1H, dd, *J* = 13.8, 3.0 Hz), 1.89 (1H, d, *J* = 7.8 Hz), 1.85 (1H, dddd, *J* = 13.8, 13.8, 13.8, 6.0 Hz), 1.60 (1H, dd, *J* = 13.8, 7.8 Hz), 1.20 (3H, t, *J* = 6.6 Hz); ^{13}C-NMR (150 MHz, $CDCl_3$): δ 170.9, 170.8, 167.5, 149.7, 146.6, 130.1, 127.5, 114.7, 112.3, 75.9, 71.8, 61.8, 51.8, 48.7, 42.9, 41.4, 39.7, 31.9, 25.5, 24.2, 21.1, 20.7, 13.3; HRMS (ESI) *m/z*: $[M+H]^+$ Calcd for $C_{23}H_{28}NO_6$ 414.1911, Found 414.1902.

Diol 46

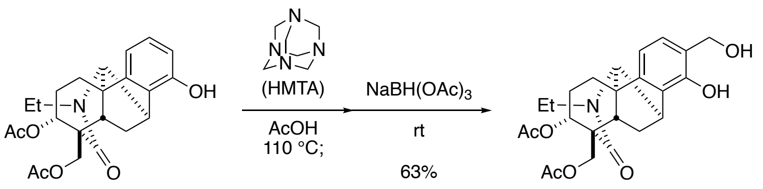

To a solution of phenol **45** (385 mg, 0.931 mmol) in AcOH (18 mL) was added hexamethylenetetramine (HTMA) (155 mg, 1.11 mmol) at room temperature. After stirring for 24 h at 110 °C, 2 M aq. HCl (27 mL) was added at 0 °C, and the mixture was stirred for 1 h at room temperature. To the resulting mixture were added $NaHCO_3$ (4.54 g, 54.0 mmol) and $NaBH(OAc)_3$ (3.90 g, 18.4 mmol) at 0 °C. After stirring for 24 h at room temperature, the reaction mixture was diluted with CH_2Cl_2 and was extracted with CH_2Cl_2 three times. The combined organic extracts were washed with brine, dried over anhydrous sodium sulfate, and filtered. The filtrate was concentrated under reduced pressure to remove organic solvents to give a crude material, which was purified by flash silica gel column chromatography (hexane-EtOAc = 1:3 to 100% EtOAc) to afford diol **46** (261 mg, 0.589 mmol, 63%). A colorless oil; R_f = 0.29 (hexane-EtOAc = 1:3); $[\alpha]_D^{18}$ = –5.93 (*c* 1.05, $CHCl_3$); IR (film): 2940, 1738, 1634, 1560, 1538, 1457, 1373, 1238, 1044, 751 cm^{-1}; ^1H-NMR (400 MHz, $CDCl_3$): δ 7.63 (1H, s), 6.89 (1H, d, *J* = 8.0 Hz), 6.68 (1H, d, *J* = 8.0 Hz), 5.03 (1H, dd, *J* = 12.4, 4.4 Hz), 4.88 (1H, d, *J* = 4.8 Hz), 4.83 (1H, d, *J* = 11.6 Hz), 4.13 (1H, d, *J* = 11.6 Hz), 3.76 (1H, dq, *J* = 14.4, 7.2 Hz), 3.58 (1H, d, *J* = 3.2 Hz), 3.26 (1H, s), 3.16 (1H, dq, *J* = 14.4, 7.2 Hz), 2.55–2.45 (2H, m), 2.25 (1H, dd, *J* = 14.4, 4.0 Hz), 2.15–2.07 (1H, m), 2.11 (3H, s), 2.00–1.94 (1H, m), 1.97 (3H, s), 1.89–1.78 (2H, m), 1.59 (1H, dd, *J* = 12.8, 8.0 Hz), 1.18 (3H, t, *J* = 7.2 Hz); ^{13}C-NMR (100 MHz, $CDCl_3$): δ 170.7, 170.6, 167.4, 149.5, 146.2, 131.0, 125.8, 124.3, 111.9, 75.8, 71.7, 64.4, 61.7, 51.7, 48.6, 42.8, 41.3, 39.8, 31.8, 25.4, 24.1, 21.1, 20.7, 13.3; HRMS (ESI) *m/z*: $[M+Na]^+$ Calcd for $C_{24}H_{29}NNaO_7$ 466.1836, Found 466.1822.

1,2-Diol 49

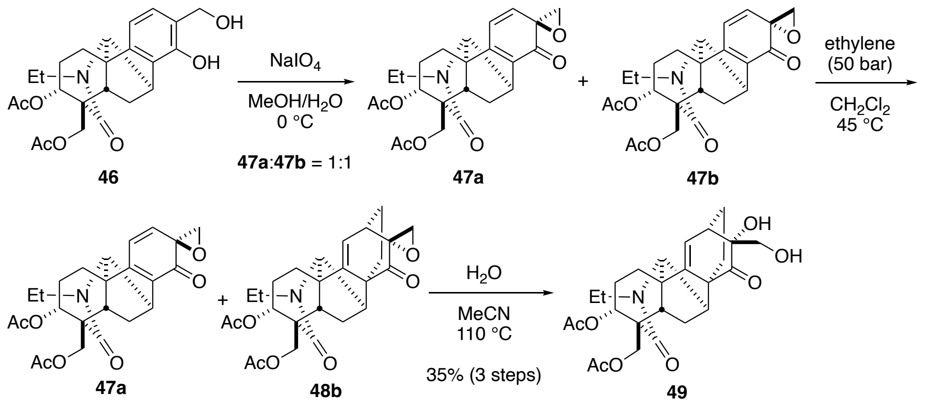

To a solution of diol **46** (355 mg, 0.800 mmol) in MeOH (8.0 mL) was added $NaIO_4$ (1.72 g, 8.04 mmol) in H_2O (19 mL) at 0 °C. After stirring for 30 min at 0 °C, the reaction mixture was diluted with H_2O at the same temperature, and the mixture was extracted with CH_2Cl_2 three times. The combined organic extracts were washed with brine, dried over anhydrous sodium sulfate, and filtered. The filtrate was concentrated under reduced pressure to remove organic solvents to give a crude material consisting of **47a** and **47b** as a 1:1 ratio, which was used for the next reaction without further purification.

A solution of the crude **47a** and **47b** in CH_2Cl_2 (11 mL) was placed in a Parr Pressure Vessel, which was then pressurized with ethylene gas (50 bar) at room temperature. After stirring for 118 h at 45 °C, ethylene pressure was released, and the resulting reaction mixture was concentrated under reduced pressure to remove the solvent. The residue was roughly purified by flash silica gel column chromatography (hexane-EtOAc = 2:1) to afford an inseparable mixture of cycloadduct **48b** and unreacted **47a**, which was used for the next reaction without further purification.

To a solution of a mixture of **48b** and **47a** in MeCN (5 mL) was added H_2O (10 mL) at room temperature. After stirring for 51 h at 110 °C, the solvents were removed under reduced pressure to give a crude material, which was purified by flash silica gel column chromatography (100% EtOAc to EtOAc-MeOH = 10:1) to afford 1,2-diol **49** (138 mg, 0.283 mmol, 35%, 3 steps). A white foam; R_f = 0.25 (100% EtOAc); $[\alpha]_D^{25}$ = +33.6 (*c* 0.320, $CHCl_3$); IR (film): 2962, 2875, 1735, 1637, 1461, 1377, 1241, 1048, 871, 770 cm^{-1}; ^{1}H-NMR (400 MHz, $CDCl_3$): δ 6.13 (1H, d, *J* = 6.8 Hz), 4.92 (1H, dd, *J* = 12.0, 4.4 Hz), 4.85 (1H, d, *J* = 12.0 Hz), 3.93 (1H, d, *J* = 12.0 Hz), 3.75 (1H, dq, *J* = 14.0, 7.2 Hz), 3.42 (2H, s), 3.35 (1H, s), 3.12 (1H, dq, *J* = 14.0, 7.2 Hz), 2.99–2.97 (1H, m), 2.57 (1H, d, *J* = 5.2 Hz), 2.29–2.23 (1H, m), 2.19–2.12 (2H, m), 2.08 (3H, s), 2.07–2.00 (5H, m), 1.94–1.70 (6H, m), 1.45 (1H, ddd, *J* = 12.0, 12.0, 4.4 Hz), 1.34 (1H, dddd, *J* = 12.0, 12.0, 3.2, 3.2 Hz), 1.18 (3H, t, *J* = 7.2 Hz); ^{13}C-NMR (100 MHz, $CDCl_3$): δ 212.9, 170.61, 170.55, 166.8, 148.0, 121.8, 77.2, 71.2, 69.4, 65.8, 60.4, 57.9, 51.8, 47.9, 43.2, 41.6, 41.5, 40.5, 27.8, 27.3, 25.0, 24.0, 21.0, 20.8, 19.6, 13.3; HRMS (ESI) *m/z*: $[M+H]^+$ Calcd for $C_{26}H_{34}NO_8$ 488.2279, Found 488.2277.

Acetal S6

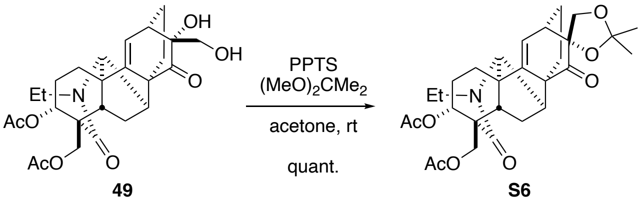

To a solution of 1,2-diol **48** (9.2 mg, 18.9 µmol) and PPTS (4.9 mg, 18.9 µmol) in acetone (920 µL) was added 2,2-dimethoxypropane (138 µL, 0.189 mmol) at room temperature. After stirring for 5 h at room temperature, the reaction was quenched with saturated aqueous $NaHCO_3$ at the same temperature, and the mixture was extracted with CH_2Cl_2 three times. The combined organic extracts were washed with brine, dried over anhydrous sodium sulfate, and filtered. The filtrate was concentrated under reduced pressure to remove organic solvents to give analytically pure acetal **S6** (10.0 mg, 19.0 µmol, quant.). A white foam.; R_f = 0.65 (EtOAc); IR (film): 2946, 1731, 1651, 1541, 1457, 1373, 1245, 1074, 1048, 751 cm^{-1}; ^{1}H-NMR (400 MHz, $CDCl_3$): δ 6.09 (1H, d, J = 6.4 Hz), 4.90 (1H, dd, J = 12.0, 4.4 Hz), 4.83 (1H, d, J = 12.0 Hz), 3.93 (1H, J = 12.0 Hz), 3.74 (1H, dq, J = 14.4, 7.2 Hz), 3.71 (2H, s), 3.33 (1H, s), 3.12 (1H, dq, J = 14.4, 7.2 Hz), 2.92–2.90 (1H, m), 2.54 (1H, d, J = 5.2 Hz), 2.19–1.99 (5H, m), 2.07 (3H, s), 2.03 (3H, s), 1.90–1.77 (2H, m), 1.77–1.68 (2H, m), 1.52 (3H, s), 1.45 (3H, s), 1.41–1.34 (2H, m), 1.18 (3H, t, J = 7.2 Hz); ^{13}C-NMR (150 MHz, $CDCl_3$): δ 209.4, 170.7, 170.4, 166.8, 149.8, 121.7, 112.3, 83.8, 72.1, 71.3, 69.6, 60.5, 57.7, 51.9, 48.1, 43.6, 43.2, 41.9, 41.6, 27.2, 27.1, 26.9, 25.9, 25.1, 24.2, 21.1, 20.8, 20.2, 13.4; HRMS (ESI) *m/z*: $[M+H]^+$ Calcd for $C_{29}H_{38}NO_8$ 528.2592, Found 528.2615.

*The stereochemistries at C8, C12, and C16 were determined on the basis of 2D NMR (COSY and NOESY).

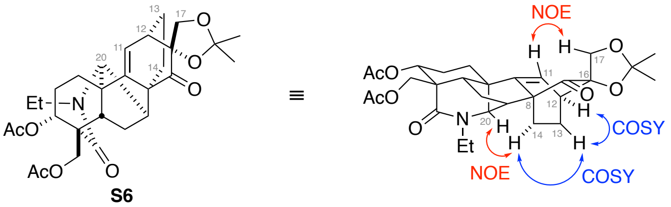

Figure S1. Key COSY and NOESY correlations of **S6** (Page S75–S76)

1,3-Diol 50

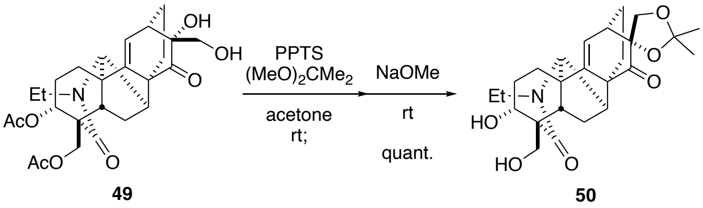

To a solution of 1,2-diol **49** (103 mg, 0.211 mmol) and PPTS (5.3 mg, 21.1 µmol) in acetone (4.2 mL) was added 2,2-dimethoxypropane (259 µL, 2.11 mmol) at room temperature. After stirring for 11 h at room temperature, NaOMe (114 mg, 2.11 mmol) was added. After stirring for 1 h at room temperature, the reaction was quenched with H_2O, and the mixture was extracted with CH_2Cl_2 three times. The combined organic extracts

were washed with brine, dried over anhydrous sodium sulfate, and filtered. The filtrate was concentrated under reduced pressure to remove organic solvents to give a crude material, which was purified by flash silica gel column chromatography (100% EtOAc to EtOAc-MeOH = 10:1) to afford 1,3-diol **50** (93.5 mg, 0.211 mmol, quant.). A white foam; R_f = 0.49 (EtOAc-MeOH = 10:1); $[\alpha]_D^{18}$ = +57.7 (*c* 0.320, $CHCl_3$); IR (film): 2940, 2878, 1731, 1622, 1538, 1373, 1219, 1067, 770, 751 cm^{-1}; ^{1}H-NMR (400 MHz, $CDCl_3$): δ 6.06 (1H, d, *J* = 6.4 Hz), 4.29 (1H, d, *J* = 11.6 Hz), 3.95 (1H, br s), 3.80 (1H, dd, *J* = 12.4, 4.4 Hz), 3.73 (1H, dq, *J* = 14.0, 7.2 Hz), 3.68 (2H, s), 3.58 (1H, br d, *J* = 10.8 Hz), 3.51 (1H, br d, *J* = 10.8 Hz), 3.31 (1H, s), 3.11 (1H, dq, *J* = 14.0, 7.2 Hz), 2.90 (1H, ddd, *J* = 6.4, 2.4, 2.4 Hz), 2.53 (1H, d, *J* = 5.2 Hz), 2.18–2.12 (1H, m), 2.07–1.96 (4H, m), 1.89–1.83 (1H, m), 1.72 (1H, ddd, *J* = 14.4, 5.2, 2.0 Hz), 1.62–1.55 (2H, m), 1.51 (3H, s), 1.45 (3H, s), 1.43–1.34 (2H, m), 1.19 (3H, t, *J* = 7.2 Hz); ^{13}C-NMR (100 MHz, $CDCl_3$): δ 209.5, 170.8, 149.9, 121.4, 112.1, 83.8, 72.5, 72.1, 69.3, 62.9, 57.7, 53.3, 48.0, 43.6, 43.0, 41.4, 41.3, 28.3, 27.3, 27.1, 27.0, 25.8, 24.2, 20.1, 13.2; HRMS (EI) *m/z*: $[M]^+$ Calcd for $C_{25}H_{33}NO_6$ 443.2302; Found 443.2312.

Tosylate S7

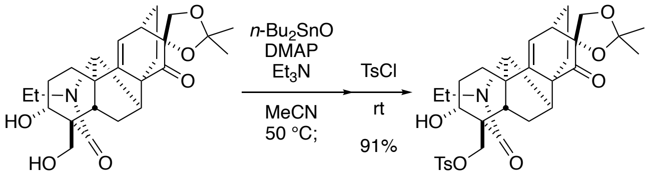

50 **S7**

To a suspension of 1,3-diol **50** (62.4 mg, 0.141 mmol), *n*-Bu_2SnO (70.0 mg, 0.281 mmol), and DMAP (34.3 mg, 0.281 mmol) in MeCN (7.5 mL) was added Et_3N (98.0 μL, 0.703 mmol) at room temperature. After stirring for 2 h at 50 °C, TsCl (40.2 mg, 0.211 mmol) was added at room temperature. After stirring for 5.5 h at room temperature, the reaction mixture was filtered through a short column of silica gel using EtOAc as an eluent. The eluate was concentrated under reduced pressure to give a crude material, which was purified by flash silica gel column chromatography (hexane-EtOAc = 1:2) to afford tosylate **S7** (77.1 mg, 0.129 mmol, 91%). A white foam; R_f = 0.41 (100% EtOAc); $[\alpha]_D^{18}$ = −3.07 (*c* 0.400, $CHCl_3$); IR (film): 2943, 2872, 1731, 1634, 1454, 1363, 1176, 967, 755, 671 cm^{-1}; ^{1}H-NMR (400 MHz, $CDCl_3$): δ 7.77 (2H, d, *J* = 8.4 Hz), 7.34 (2H, d, *J* = 8.4 Hz), 6.04 (1H, d, *J* = 6.8 Hz), 4.72 (1H, d, *J* = 10.4 Hz), 3.91 (1H, d, *J* = 10.4 Hz), 3.75–3.69 (1H, m), 3.70 (2H, s), 3.60 (1H, dq, *J* = 14.4, 7.2 Hz), 3.25 (1H, s), 3.09 (1H, dq, *J* = 14.4, 7.2 Hz), 2.89 (1H, ddd, *J* = 6.8, 2.4, 2.4 Hz), 2.51 (1H, d, *J* = 11.2 Hz), 2.49 (3H, s), 2.44 (1H, d, *J* = 5.6 Hz), 2.16–2.05 (2H, m), 1.98–1.94 (2H, m), 1.85–1.79 (1H, m), 1.73 (1H, d, *J* = 7.6 Hz), 1.62–1.54 (1H, m), 1.55 (3H, s), 1.46 (3H, s), 1.43–1.35 (4H, m), 1.13 (3H, t, *J* = 7.2 Hz); ^{13}C-NMR (100 MHz, $CDCl_3$): δ 209.2, 168.0, 149.6, 145.3, 132.1, 130.0 (2C), 128.1(2C), 121.6, 112.2, 83.9, 72.0, 69.2, 69.1, 66.5, 57.6, 54.1, 48.0, 43.5, 43.2, 41.6, 40.5, 28.8, 27.3, 27.0, 26.8, 25.9, 24.1, 21.7, 20.1, 13.1; HRMS (ESI) *m/z*: $[M+Na]^+$ Calcd for $C_{32}H_{39}NNaO_8S$ 620.2289, Found 620.2276.

Alcohol 51

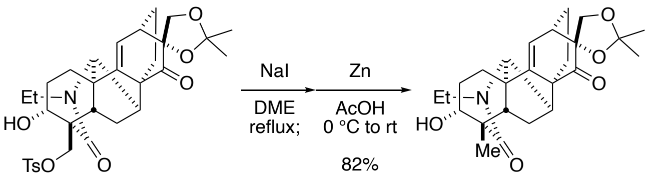

S7 **51**

To a solution of tosylate **S7** (25.6 mg, 42.8 μmol) in DME (1.1 mL) was added NaI (193 mg, 1.28 mmol) at room temperature. After stirring for 36 h at 100 °C, AcOH (1.1 mL) and Zn (28.0 mg, 0.428 mmol) were added at 0 °C and the mixture was stirred for 16 h at room temperature. The reaction was quenched with saturated aqueous $NaHCO_3$, and the mixture was extracted with CH_2Cl_2 three times. The combined organic extracts were washed with brine, dried over anhydrous sodium sulfate, and filtered. The filtrate was concentrated under reduced pressure to remove organic solvents to give a crude material, which was purified by flash silica gel column chromatography (hexane-EtOAc = 1:2) to afford alcohol **51** (15.1 mg, 35.3 μmol, 82%). A white foam; R_f = 0.30 (hexane-EtOAc = 1:2); $[\alpha]_D^{18}$ = +55.8 (*c* 0.390, $CHCl_3$); IR (film): 2976, 2936, 2875, 1731, 1625, 1457, 1260, 1216, 1074, 751 cm^{-1}; ^{1}H-NMR (600 MHz, $CDCl_3$): δ 6.03 (1H, d, *J* = 6.6 Hz), 3.69–3.63 (1H, m), 3.68 (2H, s), 3.33 (1H, ddd, *J* = 12.0, 12.0, 4.2 Hz), 3.27 (1H, s), 3.15 (1H, dq, *J* = 14.4, 7.2 Hz), 2.93 (1H, d, *J* = 10.2 Hz), 2.89 (1H, ddd, *J* = 6.6, 3.0, 3.0 Hz), 2.53 (1H, d, *J* = 5.4 Hz), 2.16 (1H, dddd, *J* = 9.0, 9.0, 4.2, 3.0 Hz), 2.06–1.94 (4H, m), 1.85 (1H, ddd, *J* = 12.6, 9.0, 4.2 Hz), 1.73 (1H, ddd, *J* = 14.4, 5.4, 3.0 Hz), 1.52 (3H, s), 1.46–1.42 (2H, m), 1.45 (3H, s), 1.42–1.35 (2H, m), 1.27 (3H, s), 1.17 (3H, *J* = 7.2 Hz); ^{13}C-NMR (150 MHz, $CDCl_3$): δ 209.7, 172.3, 150.2, 121.0, 112.1, 83.9, 75.2, 72.1, 69.4, 57.7, 50.6, 48.0, 46.6, 43.6, 43.1, 41.6, 29.4, 27.7, 27.2, 27.0, 25.8, 24.3, 20.2, 18.0, 13.2; HRMS (EI) *m/z*: $[M]^+$ Calcd for $C_{25}H_{33}NO_5$ 427.2353, Found 427.2349.

Proposed structure of acochlearine (7)

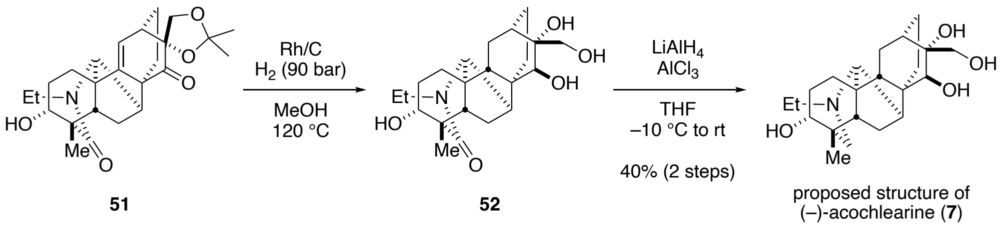

51 **52** proposed structure of (–)-acochlearine (**7**)

A suspension of alcohol **51** (9.0 mg, 21.1 μmol) and 5% Rh/C (55% suspension in H_2O) (42.6 mg, 11.3 μmol) in MeOH (1.7 mL) was placed in a Parr Pressure Vessel, which was pressurized with hydrogen gas (90 bar) at room temperature. After stirring for 16.5 h at 120 °C, hydrogen pressure was released, and the resulting reaction mixture was filtered through a pad of Celite and the filter cake was washed with CH_2Cl_2 and MeOH. The filtrate was concentrated under reduced pressure to give a crude **52**, which was used for the next reaction without further purification.

To a solution of crude **52** and $AlCl_3$ (8.4 mg, 63.3 μmol) in THF (850 μL) was added $LiAlH_4$ (8.0 mg, 0.211 mmol) at –10 °C. After stirring for 2 h at room temperature, the reaction was quenched by slow addition of H_2O (8.0 μL), 15% aqueous NaOH (8.0 μL), and H_2O (24.0 μL), sequentially, at –10 °C. The resulting suspension

was diluted with 15% aqueous NaOH at room temperature and extracted with 33% EtOH in $CHCl_3$ five times. The combined organic extracts were washed with brine, dried over anhydrous sodium sulfate, and filtered. The filtrate was concentrated under reduced pressure to remove organic solvents to give a crude material, which was purified by preparative TLC (CH_2Cl_2-MeOH-Et_3N = 88:10:2 then $CHCl_3$-MeOH-Et_3N = 75:5:1) to afford proposed structure of acochlearine (**7**) (3.2 mg, 8.48 μmol, 40%, 2 steps). A white foam; R_f = 0.26 (CH_2Cl_2-MeOH-NH_4OH = 88:10:2); $[\alpha]_D^{18}$ = –53.3 (*c* 0.120, $CHCl_3$); IR (film): 3372, 2952, 2917, 2869, 2849, 1541, 1502, 1457, 1032, 758 cm^{-1}; ^{1}H-NMR (600 MHz, $CDCl_3$): δ 4.20 (1H, d, *J* = 11.4 Hz), 4.04 (1H, s), 3.46 (1H, d, *J* = 11.4 Hz), 3.40 (1H, s), 3.38 (1H, dd, *J* = 13.2, 4.8 Hz), 2.90 (1H, d, *J* = 11.4 Hz), 2.55 (1H, dd, *J* = 13.8, 8.4 Hz), 2.51–2.47 (1H, m), 2.45–2.41 (1H, m), 2.36 (1H, dddd, *J* = 13.2, 13.2, 13.2, 5.4 Hz), 2.06 (1H, d, *J* = 4.8 Hz), 1.99–1.90 (4H, m), 1.81 (1H, dd, *J* = 12.0, 9.6 Hz), 1.70–1.67 (1H, m), 1.57–1.47 (3H, m), 1.35–1.31 (2H, m), 1.30–1.27 (1H, m), 1.22–1.15 (2H, m), 1.05 (3H, t, *J* = 8.4 Hz), 0.81 (3H, s); ^{13}C-NMR (150 MHz, $CDCl_3$): δ 88.1, 79.2, 77.7, 71.9, 68.0, 52.7, 50.8, 50.2, 44.9, 42.9, 42.1, 39.9, 39.6, 36.8, 29.4, 27.3, 24.7, 23.4, 23.1, 22.2, 21.9, 13.6; HRMS (ESI) *m/z*: $[M+H]^+$ Calcd for $C_{22}H_{36}NO_4$ 378.2639, Found 378.2641. The spectral data of **7** were not identical with those of the reported natural product (Table S1 and S2).[2] The stereochemistry of **7** was determined on the basis of 2D NMR (COSY, HMQC, HMBC, and NOESY) (Figure S3 and S4).

Table S1. Comparison of ^{1}H-NMR chemical shifts between the synthetic proposed structure of (–)-acochlearine (7) and the reported natural (–)-acochlearine (7)

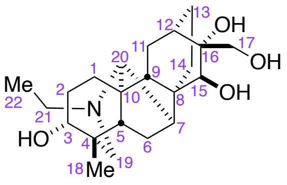

proposed structure of (–)-acochlearine (**7**)

	Synthetic proposed structure of (–)-acochlearine (7) ($CDCl_3$, 600 MHz)	Reported natural (–)-acochlearine ($CDCl_3$, 500 MHz)[2]
**H-17A**	4.20 (1H, d, *J* = 11.4 Hz)	4.15 (1H, d, *J* = 11.0 Hz)
**H-15**	4.04 (1H, s)	4.00 (1H, br s)
**H-20**	3.40 (1H, s)	3.88 (1H, s)
**H-3**	3.38 (1H, dd, *J* = 13.2, 4.8 Hz)	3.80 (1H, dd, *J* = 10.0, 6.5 Hz)
**H-17B**	3.46 (1H, d, *J* = 11.4 Hz)	3.51 (1H, d, *J* = 11.0 Hz)
**H-6A**	2.55 (1H, dd, *J* = 13.8, 8.4 Hz)	2.80 (1H, dd, *J* = 14.0, 7.0 Hz)
**H-19A**	2.90 (1H, d, *J* = 11.4 Hz)	2.69 (1H, d, *J* = 11.0 Hz)
**H-21A**	2.51–2.47 (1H, m)	2.60 (1H, m)
**H-21B**	2.45–2.41 (1H, m)	2.54 (1H, m)
**H-19B**	1.99–1.90 (1H, m)	2.28 (1H, d, *J* = 11.0 Hz)
**H-12**	1.57–1.47 (1H, m)	2.14 (1H, m)
**H-2A**	2.36 (1H, dddd, *J* = 13.2, 13.2, 13.2, 5.4 Hz)	2.13 (1H, m)
**H-14A**	1.99–1.90 (1H, m)	2.12 (1H, m)
**H-2B**	1.70–1.67 (1H, m)	1.93 (1H, m)
**H-13A**	1.99–1.90 (1H, m)	1.93 (1H, m)
**H-11A**	1.57–1.47 (1H, m)	1.92 (1H, m)
**H-5**	1.30–1.27 (1H, m)	1.87 (1H, d, *J* = 8.5 Hz)
**H-11B**	1.22–1.15 (1H, m)	1.62 (1H, m)
**H-6B**	1.35–1.31 (1H, m)	1.60 (1H, m)
**H-1A**	1.99–1.90 (1H, m)	1.55 (1H, m)
**H-7**	2.06 (1H, d, *J* = 4.8 Hz)	1.50 (1H, br s)
**H-9**	1.81 (1H, dd, *J* = 12.0, 9.6 Hz)	1.34 (1H, dd, *J* = 9.0, 8.0 Hz)
**H-1B**	1.57–1.47 (1H, m)	1.25 (1H, m)
**H-13B**	1.35–1.31 (1H, m)	1.23 (1H, m)
**H-22**	1.05 (3H, t, *J* = 4.8 Hz)	1.23 (3H, t, *J* = 7.3 Hz)
**H-14B**	1.22–1.15 (1H, m)	1.12 (1H, m)
**H-18**	0.81 (3H, s)	0.72 (3H, s)

Titration experiments of the synthetized proposed acochlearine (7) with acids.

Considering that the discrepancy in the ^{1}H-NMR shift values from the literature values would be due to protonation of the highly basic amino group, we measured ^{1}H-NMR by mixing the synthesized compound with various acids at different molar ratios according to Sarpong's reports.[3] Initially, **7** was treated with 0.5 to 1.0 equivalent of TFA to generate its TFA salt. However, **7** was gradually decomposed when TFA was added to the $CDCl_3$ solution of **7**. In contrast, formic acid salt of **7** was stable. Figure S2 showed the ^{1}H-NMR spectra of **7** with 0, 0.25, 0.50, and 1.0 equivalent of formic acid. However, the ^{1}H-NMR spectra on Figure S2 were inconsistent with those reported for the compound isolated from nature.[2]

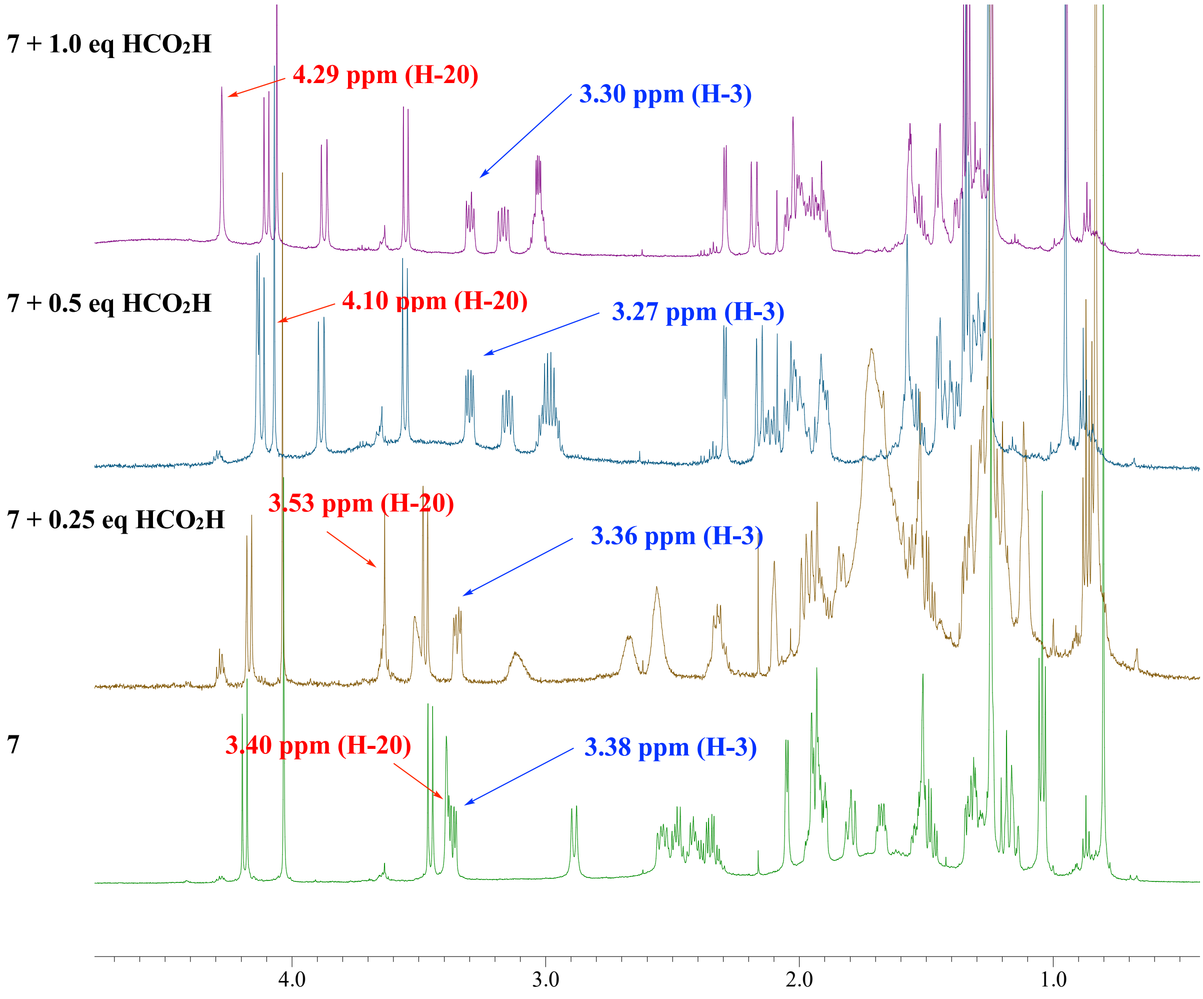

Figure S2. ^{1}H-NMRs of mixtures of 7 and formic acid

Table S2. Comparison of ^{13}C-NMR chemical shifts between the synthetic proposed (–)-acochlearine (7) and the reported natural (–)-acochlearine (7)

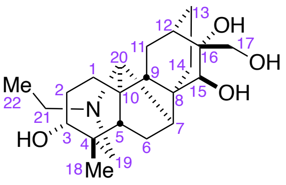

proposed
(–)-acochlearine (**7**)

	Synthetic proposed structure of (–)-acochlearine (7) (150 MHz, $CDCl_3$)	Reported natural (–)-acochlearine (125 MHz, $CDCl_3$)[2]	Deviation (synthetic–natural) $\Delta\delta$ (ppm)
**C-15**	88.1	86.7	1.5
**C-16**	79.2	78.8	0.4
**C-3**	77.7	70.0	7.7
**C-17**	68.0	67.8	0.2
**C-20**	71.9	66.8	5.1
**C-19**	50.2	56.9	–6.7
**C-9**	39.9	52.3	–12.4
**C-21**	50.8	51.7	–1.1
**C-10**	44.9	51.5	–6.6
**C-12**	36.8	42.6	5.8
**C-8**	42.1	42.2	–0.1
**C-5**	52.7	40.8	–11.9
**C-1**	24.7	38.0	–13.3
**C-7**	42.9	36.5	6.4
**C-4**	39.6	33.9	5.7
**C-2**	29.4	30.9	–1.5
**C-14**	27.3	26.9	-3.7
**C-18**	22.2	24.1	–1.9
**C-6**	23.1	23.7	–0.8
**C-13**	21.9	23.5	3.9
**C-11**	23.4	21.3	0.6
**C-22**	13.6	12.4	1.2

*We assigned the signals of ^{1}H- and ^{13}C-NMR on the basis of 2D NMR (COSY, HMQC, and HMBC), and determined the stereochemistries by NOESY (Page S81–S84).

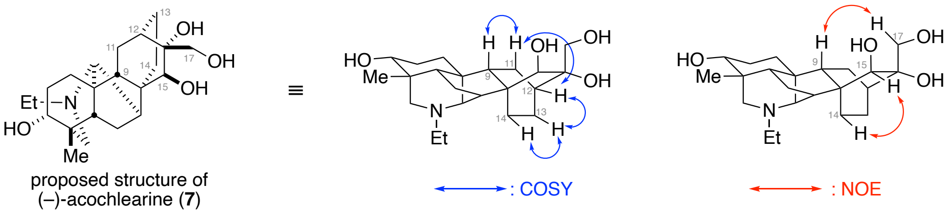

Figure S3. Key COSY and NOESY correlations of proposed structure of acochlearine (**7**) (Page S81, S84)

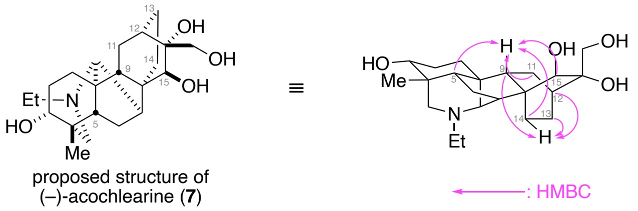

Figure S4. Key HMBC correlations of proposed structure of acochlearine (**7**) (Page S83)

Synthesis of S11 (C3 epimer of 7)

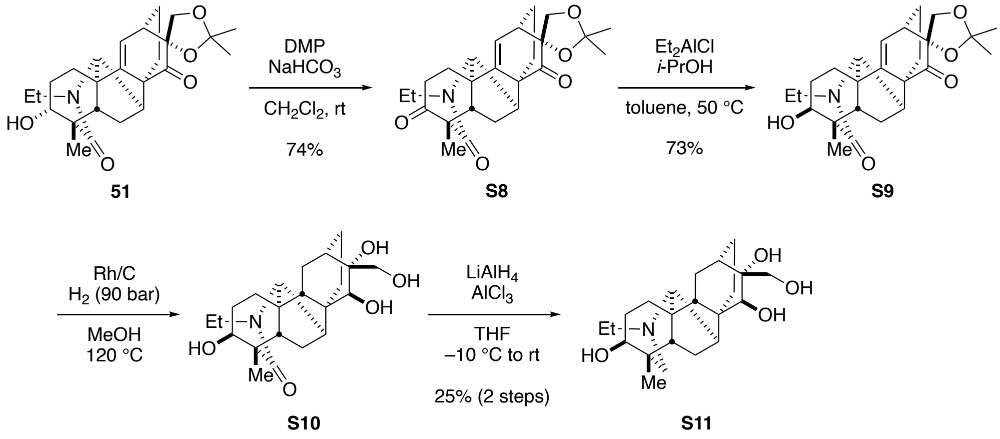

Ketone S8

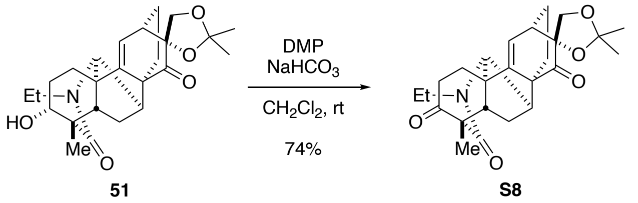

To a solution of alcohol **51** (16.4 mg, 38.4 μmol) and $NaHCO_3$ (32.3 mg, 0.384 mmol) in CH_2Cl_2 (1.0 mL) was added Dess–Martin periodinane (48.8 mg, 0.115 mmol) at room temperature. After stirring for 50 min at room temperature, the reaction was quenched with saturated aqueous $Na_2S_2O_3$ at the same temperature, and the

mixture was extracted with CH_2Cl_2 three times. The combined organic extracts were washed with brine, dried over anhydrous sodium sulfate, and filtrated. The filtrate was concentrated under reduced pressure to remove organic solvents to give a crude material, which was purified by preparative TLC (hexane-acetone = 3:1) to afford ketone **S8** (12.1 mg, 28.4 μmol, 74%). A white foam; R_f = 0.42 (hexane-acetone = 2:1); $[\alpha]_D^{16}$ = +143 (*c* 0.380, $CHCl_3$); IR (film): 2914, 1725, 1644, 1457, 1432, 1370, 1257, 1074, 903, 755 cm^{-1}; ^{1}H-NMR (600 MHz, $CDCl_3$): δ 6.09 (1H, d, *J* = 7.2 Hz), 3.72 (1H, dq, *J* = 14.4, 7.2 Hz), 3.67 (2H, s), 3.52 (1H, d, *J* = 1.2 Hz), 3.20 (1H, dq, *J* = 14.4, 7.2 Hz), 2.91 (1H, ddd, *J* = 7.2, 3.0, 3.0 Hz), 2.74 (1H, ddd, *J* = 14.4, 12.0, 9.6 Hz), 2.66 (1H, d, *J* = 5.4 Hz), 2.43 (1H, ddd, *J* = 14.4, 12.0, 3.6 Hz), 2.36–2.27 (2H, m), 2.21–2.13 (2H, m), 1.91 (1H, ddd, *J* = 13.2, 9.6, 3.6 Hz), 1.79–1.76 (2H, m), 1.53–1.48 (4H, m), 1.45 (3H, s), 1.41 (1H, dddd, *J* = 12.0, 12.0, 3.6, 3.6 Hz), 1.24 (3H, s), 1.19 (3H, t, *J* = 7.2 Hz); ^{13}C-NMR (150 MHz, $CDCl_3$): δ 209.4, 206.1, 168.6, 148.9, 121.8, 112.2, 83.8, 72.1, 70.3, 60.6, 57.8, 49.5, 48.6, 44.1, 43.1, 42.2, 35.6, 28.8, 27.3, 27.1, 25.83, 25.76, 20.2, 14.9, 12.8; HRMS (ESI) *m/z*: $[M+H]^+$ Calcd for $C_{25}H_{32}NO_5$ 426.2275, Found 426.2293.

Alcohol S9

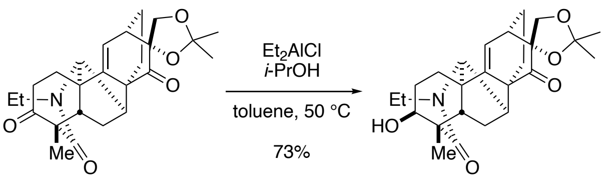

S8 **S9**

A stock solution of aluminum alkoxide was prepared by addition of *i*-PrOH (109 μL, 1.42 mmol) to a solution of Et_2AlCl (0.871 M in *n*-hexane, 326 μL, 0.284 mmol) in toluene (500 μL) at room temperature, and the resulting mixture was stirred for 10 min at room temperature. To a solution of ketone **S8** (12.1 mg, 28.4 μmol) in toluene (500 μL) was added the solution of aluminum alkoxide at room temperature. After stirring for 1.5 h at 50 °C, the reaction was quenched with saturated aqueous Rochelle's salt at room temperature, and the mixture was stirred for 30 min at room temperature. The mixture was extracted with EtOAc three times. The combined organic extracts were washed with brine, dried over anhydrous sodium sulfate, and filtrated. The filtrate was concentrated under reduced pressure to remove organic solvents to give a crude material, which was purified by flash silica gel column chromatography (CH_2Cl_2-MeOH = 20:1) to afford alcohol **S9** (8.9 mg, 20.8 μmol, 73%). A white foam; R_f = 0.50 (CH_2Cl_2-MeOH = 10:1); $[\alpha]_D^{16}$ = +41.0 (*c* 0.120, $CHCl_3$) ; IR (film): 3416, 2940, 1728, 1628, 1461, 1373, 1257, 1216, 1074, 900 cm^{-1}; ^{1}H-NMR (600 MHz, $CDCl_3$): δ 6.04 (1H, d, *J* = 6.6 Hz), 3.88 (1H, br s), 3.71–3.63 (3H, m), 3.26 (1H, s), 3.07 (1H, dq, *J* = 14.4, 7.2 Hz), 2.88 (1H, ddd, *J* = 6.6, 3.0, 3.0 Hz), 2.49 (1H, d, *J* = 4.8 Hz), 2.28 (1H, ddd, *J* = 13.2, 13.2, 6.0 Hz), 2.14 (1H, dddd, *J* = 14.4, 9.6, 4.8, 2.4 Hz), 2.00 (1H, ddd, *J* = 14.4, 4.8, 1.2 Hz), 1.87–1.81 (3H, m), 1.78–1.74 (2H, m), 1.62 (1H, ddd, *J* = 14.4, 6.0, 2.4 Hz), 1.52 (3H, s), 1.46–1.42 (4H, m), 1.37 (1H, dddd, *J* = 12.0, 12.0, 3.6, 3.0 Hz), 1.20 (3H, s), 1.13 (3H, t, *J* = 7.2 Hz); ^{13}C-NMR (150 MHz, $CDCl_3$): δ 209.9, 172.5, 150.8, 120.6, 112.1, 84.1, 72.3, 72.2, 69.4, 57.9, 50.4, 48.1, 43.4, 43.2, 41.6, 40.4, 27.4, 27.1, 26.92, 26.87, 25.9, 20.9, 20.3, 18.5, 12.9; HRMS (EI) *m/z*: $[M]^+$ Calcd for $C_{25}H_{33}NO_5$ 427.2353, Found 427.2362.

Tetraol S11

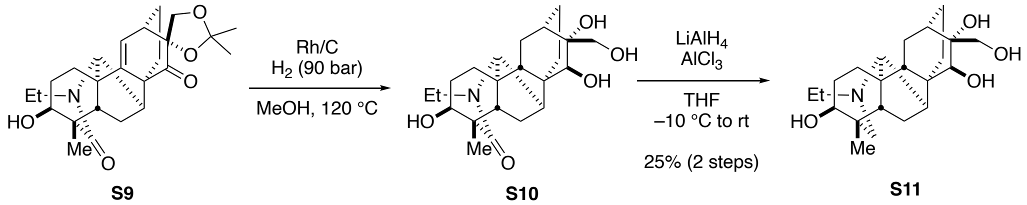

S9 **S10** **S11**

To a suspension of alcohol **S9** (8.9 mg, 20.8 μmol) and 5% Rh/C (55% suspension in H_2O) (77.8 mg, 20.8 μmol) in MeOH (2.1 mL) was placed in a Parr Pressure Vessel, which was pressurized with hydrogen gas (90 bar) at room temperature. After stirring for 12 h at 120 °C, hydrogen pressure was released, and the resulting reaction mixture was filtered through a pad of Celite and the filter cake was washed with MeOH. The combined filtrate and washings were concentrated under reduced pressure to remove organic solvents to give a crude **S10**, which was used for the next reaction without further purification.

To a solution of the crude **S10** and $AlCl_3$ (55.5 mg, 0.416 mmol) in THF (1.0 mL) was added $LiAlH_4$ (47.4 mg, 1.25 mmol) at –10 °C. After stirring for 5 h at room temperature, the reaction was quenched by slow addition of H_2O (8.0 μL), 15% aqueous NaOH (8.0 μL), and H_2O (24.0 μL), sequentially, at –10 °C. The resulting suspension was diluted with 15% aqueous NaOH at room temperature and extracted with 33% EtOH in $CHCl_3$ five times. The combined organic extracts were washed with brine, dried over anhydrous sodium sulfate, and filtered. The filtrate was concentrated under reduced pressure to remove organic solvents to give a crude material, which was purified by preparative TLC ($CHCl_3$-MeOH-NH_4OH = 88:10:2) to afford tetraol **S11** (2.0 mg, 5.30 μmol, 25%, 2 steps). A white foam; R_f = 0.22 ($CHCl_3$-MeOH-NH_4OH = 88:10:2); $[\alpha]_D^{18}$ = –64.2 (*c* 0.200, $CHCl_3$); IR (film): 3320, 2955, 2921, 2869, 1451, 1373, 1209, 1048, 945, 755 cm^{-1}; ^1H-NMR (600 MHz, $CDCl_3$): δ 4.20 (1H, d, *J* = 11.4 Hz), 4.04 (1H, s), 3.57 (1H, br s), 3.46 (1H, d, *J* = 11.4 Hz), 3.41 (1H, br s), 2.61–2.48 (3H, m), 2.44–2.40 (1H, m), 2.33 (1H, d, *J* = 10.8 Hz), 2.19 (1H, d, *J* = 10.8 Hz), 2.04 (1H, d, *J* = 4.8 Hz), 1.98–1.91 (2H, m), 1.86 (1H, dd, *J* = 11.4, 11.4 Hz), 1.72–1.69 (2H, m), 1.65–1.52 (4H, m), 1.34–1.29 (1H, m), 1.26–1.14 (3H, m), 1.03 (3H, t, *J* = 6.6 Hz), 0.83 (3H, s); ^{13}C-NMR (150 MHz, $CDCl_3$): δ 88.2, 79.2, 77.2, 72.3, 67.9, 55.8, 50.6, 46.5, 44.8, 42.9, 42.5, 40.0, 38.3, 36.8, 28.2, 27.4, 23.2, 22.9, 22.2, 21.9, 20.8, 13.6; HRMS (EI) *m/z*: $[M]^+$ Calcd for $C_{22}H_{35}NO_4$ 377.2561, Found 377.2560.

Table S3. Comparison of ^{1}H-NMR chemical shifts between the C3 epimer of the proposed structure of (–)-acochlearine (S11) and the reported natural (–)-acochlearine

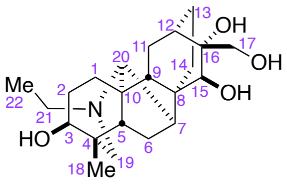

S11
(C3 epimer of **7**)

	C3 epimer of the proposed structure of (–)-acochlearine (**S11**) ($CDCl_3$, 600 MHz)	Reported natural (–)-acochlearine ($CDCl_3$, 500 MHz)[2]
**H-17A**	4.20 (1H, d, *J* = 11.4 Hz)	4.15 (1H, d, *J* = 11.0 Hz)
**H-15**	4.04 (1H, s)	4.00 (1H, br s)
**H-20**	3.41 (1H, br s)	3.88 (1H, s)
**H-3**	3.57 (1H, br s)	3.80 (1H, dd, *J* = 10.0, 6.5 Hz)
**H-17B**	3.46 (1H, d, *J* = 11.4 Hz)	3.51 (1H, d, *J* = 11.0 Hz)
**H-6A**	2.61–2.48 (1H, m)	2.80 (1H, dd, *J* = 14.0, 7.0 Hz)
**H-19A**	2.33 (1H, d, *J* = 10.8 Hz)	2.69 (1H, d, *J* = 11.0 Hz)
**H-21A**	2.61–2.48 (1H, m)	2.60 (1H, m)
**H-21B**	2.44–2.40 (1H, m)	2.54 (1H, m)
**H-19B**	2.19 (1H, d, *J* = 10.8 Hz)	2.28 (1H, d, *J* = 11.0 Hz)
**H-12**	1.65–1.52 (1H, m)	2.14 (1H, m)
**H-2A**	2.61–2.48 (1H, m)	2.13 (1H, m)
**H-14A**	1.98–1.91 (1H, m)	2.12 (1H, m)
**H-2B**	1.65–1.52 (1H, m)	1.93 (1H, m)
**H-13A**	1.98–1.91 (1H, m)	1.93 (1H, m)
**H-11A**	1.65–1.52 (1H, m)	1.92 (1H, m)
**H-5**	1.65–1.52 (1H, m)	1.87 (1H, d, *J* = 8.5 Hz)
**H-11B**	1.26–1.14 (1H, m)	1.62 (1H, m)
**H-6B**	1.26–1.14 (1H, m)	1.60 (1H, m)
**H-1A**	1.72–1.69 (1H, m)	1.55 (1H, m)
**H-7**	2.04 (1H, d, *J* = 4.8 Hz)	1.50 (1H, br s)
**H-9**	1.86 (1H, dd, *J* = 11.4, 11.4 Hz)	1.34 (1H, dd, *J* = 9.0, 8.0 Hz)
**H-1B**	1.72–1.69 (1H, m)	1.25 (1H, m)
**H-13B**	1.34–1.29 (1H, m)	1.23 (1H, m)
**H-22**	1.03 (3H, t, *J* = 6.6 Hz)	1.23 (3H, t, *J* = 7.3 Hz)
**H-14B**	1.26–1.14 (1H, m)	1.12 (1H, m)
**H-18**	0.83 (3H, s)	0.72 (3H, s)

Table S4. Comparison of ^{13}C-NMR chemical shifts between the C3 epimer of the proposed (–)-acochlearine (S11) and the reported natural (–)-acochlearine

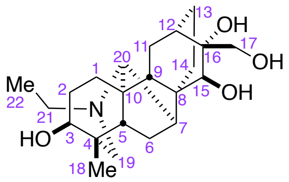

S11
(C3 epimer of **7**)

	C3 epimer of the proposed (–)-acochlearine (**S11**) (150 MHz, $CDCl_3$)	Reported natural (–)-acochlearine (125 MHz, $CDCl_3$)[2]	Deviation (synthetic–natural) $\Delta\delta$ (ppm)
**C-15**	88.2	86.7	1.5
**C-16**	79.2	78.8	0.4
**C-3**	77.2	70.0	7.2
**C-17**	67.9	67.8	0.1
**C-20**	72.3	66.8	5.5
**C-19**	55.8	56.9	–1.1
**C-9**	40.0	52.3	–12.3
**C-21**	50.6	51.7	–1.1
**C-10**	44.8	51.5	–6.7
**C-12**	36.8	42.6	–5.8
**C-8**	42.5	42.2	0.3
**C-5**	46.5	40.8	–5.7
**C-1**	20.8	38.0	–17.2
**C-7**	42.9	36.5	6.4
**C-4**	38.3	33.9	4.4
**C-2**	28.2	30.9	–2.7
**C-14**	27.4	26.9	0.5
**C-18**	22.2	24.1	–1.9
**C-6**	22.9	23.7	–0.8
**C-13**	21.9	23.5	–1.6
**C-11**	23.2	21.3	1.9
**C-22**	13.6	12.4	1.2

*We assigned the signals of ^{1}H- and ^{13}C-NMR on the basis of 2D NMR (COSY, HMQC, and HMBC) (Page S88–S90).

Diene 53

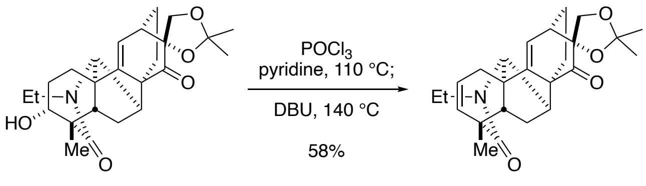

To a solution of alcohol **51** (33.2 mg, 77.7 μmol) in pyridine (2.6 mL) was added $POCl_3$ (36.3 μL, 0.388 mmol) at room temperature. After stirring for 18 h at 110 °C, DBU (2.6 mL) was added to the reaction mixture at room temperature and stirred for 24 h at 140 °C. The solvent removed under reduced pressure, and the residue was filtered through a short column of silica gel using 66% EtOAc in hexane as an eluent. The eluate was concentrated under reduced pressure to give a crude material, which was purified by flash silica gel column chromatography (hexane-EtOAc = 1:1) to afford diene **53** (18.6 mg, 45.4 μmol, 58%). A white foam; R_f = 0.49 (hexane-EtOAc = 1:2); $[\alpha]_D^{14}$ = –24.9 (*c* 0.220, $CHCl_3$); IR (film): 2969, 2930, 1731, 1644, 1457, 1367, 1260, 1212, 1064, 755 cm^{-1}; ^{1}H-NMR (600 MHz, $CDCl_3$): δ 6.09 (1H, d, *J* = 6.0 Hz), 5.82 (1H, ddd, *J* = 9.6, 3.6, 3.6 Hz), 5.69 (1H, ddd, *J* = 9.6, 2.4, 2.4 Hz), 3.69 (2H, s), 3.56 (1H, dq, *J* = 14.4, 7.2 Hz), 3.26 (1H, s), 3.09 (1H, dq, *J* = 14.4, 7.2 Hz), 2.91 (1H, ddd, *J* = 6.0, 3.0, 3.0 Hz), 2.64 (1H, ddd, *J* = 19.2, 1.8, 1.8 Hz), 2.47 (1H, d, *J* = 6.0 Hz), 2.31 (1H, ddd, *J* = 19.2, 3.0, 3.0 Hz), 2.19–2.14 (1H, m), 2.03 (1H, ddd, *J* = 14.4, 7.2, 1.2 Hz), 1.88 (1H, ddd, *J* = 12.0, 9,6, 4.8 Hz), 1.64 (1H, ddd, *J* = 14.4, 4.8, 2.4 Hz), 1.53 (3H, s), 1.49–1.47 (2H, m), 1.45 (3H, s), 1.41 (1H, dddd, *J* = 12.0, 12.0, 2.4, 2.4 Hz), 1.25 (3H, s), 1.07 (3H, t, *J* = 7.2 Hz); ^{13}C-NMR (150 MHz, $CDCl_3$): δ 209.9, 172.0, 150.1, 134.0, 126.7, 120.9, 112.2, 84.0, 72.0, 71.6, 58.6, 48.2, 46.1, 43.7, 43.2, 42.3, 41.6, 27.5, 27.11, 27.08, 26.5, 25.9, 20.6, 20.2, 13.3; HRMS (ESI) *m/z*: $[M+H]^+$ Calcd for $C_{25}H_{32}NO_4$ 410.2326, Found 410.2324.

Enone 54

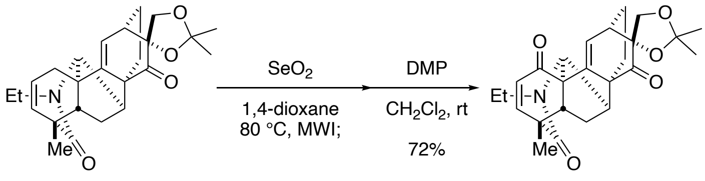

To a solution of diene **53** (10.9 mg, 26.6 μmol) in 1,4-dioxane (1.3 mL) was added SeO_2 (57.1 mg, 0.515 mmol) at room temperature. After stirring for 6 h at 80 °C under irradiation of microwave, Dess–Martin periodinane (54.7 mg, 0.129 mmol) and CH_2Cl_2 (1.3 mL) were added to the reaction mixture and the resulting mixture was stirred for 12 h at room temperature. The mixture was directly filtered through a short column of silica gel using EtOAc as an eluent. The eluate was concentrated under reduced pressure to give a crude material, which was purified by flash silica gel column chromatography (hexane-acetone = 5:1 to 3:1) to afford enone **54** (8.1 mg, 19.1 μmol, 72%). A white foam; R_f = 0.38 (hexane-acetone = 3:1); $[\alpha]_D^{23}$ = –54.0 (*c* 0.360, $CHCl_3$); IR (film): 2979, 2936, 2875, 1735, 1683, 1651, 1464, 1370, 1264, 1212 cm^{-1}; ^{1}H-NMR (600 MHz, $CDCl_3$): δ 6.89 (1H, d, *J* = 9.6 Hz), 6.21 (1H, d, *J* = 7.2 Hz), 6.19 (1H, d, *J* = 9.6 Hz), 3.70–3.66 (3H, m), 3.52 (1H, dq, *J* = 14.4, 7.2 Hz), 3.13 (1H, dq, *J* = 14.4, 7.2 Hz), 2.94–2.92 (1H, m), 2.67 (1H, d, *J* = 4.8 Hz), 2.35–2.33 (1H,

m), 2.19–2.14 (2H, m), 1.92–1.89 (1H, m), 1.71 (1H, ddd, *J* = 14.4, 7.2, 1.8 Hz), 1.63–1.56 (2H, m), 1.52 (3H, s), 1.45 (3H, s), 1.38 (3H, s), 1.05 (3H, t, *J* = 7.2 Hz); ^{13}C-NMR (150 MHz, $CDCl_3$): δ 209.6, 195.1, 169.7, 156.0, 143.3, 130.1, 125.2, 112.2, 83.9, 72.0, 69.8, 58.9, 58.5, 48.6, 47.2, 44.6, 43.3, 42.3, 27.5, 27.2, 27.0, 25.9, 20.1, 20.0, 12.9; HRMS (EI) *m/z*: $[M]^+$ Calcd for $C_{25}H_{29}NO_5$ 423.2040, Found 423.2042.

(–)-Cochlearenine (4)

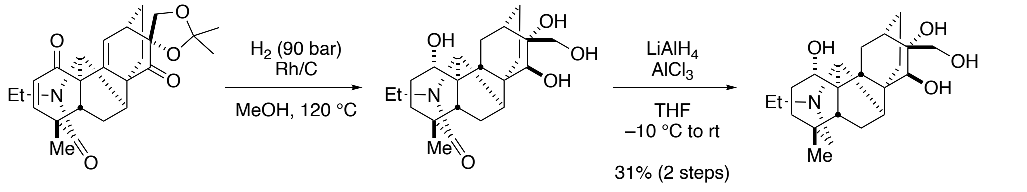

A suspension of enone **54** (8.1 mg, 19.1 μmol) and 5% Rh/C (55% suspension in H_2O) (71.5 mg, 19.1 μmol) in MeOH (1.9 mL) was placed in a Parr Pressure Vessel and pressurized hydrogen gas (90 bar) at room temperature. After stirring for 11 h at 120 °C, hydrogen pressure was released, and the reaction mixture was filtered through a pad of Celite and the filter cake was washed with MeOH. The combined filtrate and washings were concentrated under reduced pressure to give a crude **55**, which was used for the next reaction without further purification.

To a solution of the crude **55** and $AlCl_3$ (25.5 mg, 0.191 mmol) in THF (1.0 mL) was added $LiAlH_4$ (21.7 mg, 0.573 mmol) at –10 °C. After stirring for 4.5 h at room temperature, the reaction was quenched by slow addition of H_2O (21.7 μL), 15% aqueous NaOH (21.7 μL), and H_2O (65.1 μL), sequentially, at –10 °C. The resulting suspension was diluted with 15% aqueous NaOH at room temperature and extracted with 33% EtOH in $CHCl_3$ five times. The combined organic extracts were washed with brine, dried over anhydrous sodium sulfate, and filtered. The filtrate was concentrated under reduced pressure to remove organic solvents to give a crude material, which was purified by preparative TLC (CH_2Cl_2-MeOH-NH_4OH = 88:10:2) to afford (–)-cochlearenine (**4**) (2.2 mg, 5.8 μmol, 31%, 2 steps). A white foam.; R_f = 0.31 (CH_2Cl_2-MeOH-NH_4OH = 88:10:2); $[\alpha]_D^{18}$ = –41.9 (*c* 0.220, $CHCl_3$); IR (film): 3394, 2924, 2856, 1721, 1574, 1457, 1377, 1260, 1216, 1064 cm^{-1}; ^{1}H-NMR (600 MHz, $CDCl_3$): δ 4.21 (1H, d, *J* = 11.4 Hz), 4.03 (1H, s), 3.83 (1H, dd, *J* = 10.2, 6.0 Hz), 3.72 (1H, s), 3.49 (1H, d, *J* = 11.4 Hz), 2.62 (1H, dd, *J* = 12.0, 4.8 Hz), 2.55–2.49 (2H, m), 2.40 (1H, dq, *J* = 7.2 Hz), 2.25–2.18 (2H, m), 2.09 (1H, d, *J* = 6.0 Hz), 2.04–1.90 (3H, m), 1.87–1.83 (2H, m), 1.68–1.63 (1H, m), 1.58 (1H, ddd, *J* = 12.0, 4.2, 4.2 Hz), 1.51 (1H, br s), 1.39 (1H, ddd, *J* = 12.0, 12.0, 3.0, 3.0 Hz), 1.31–1.26 (3H, m), 1.13 (1H, ddd, *J* = 11.4, 11.4, 1.8 Hz), 1.04 (3H, t, *J* = 7.2 Hz), 0.71 (3H, s); ^{13}C-NMR (150 MHz, $CDCl_3$): δ 87.5, 78.8, 70.9, 67.8, 67.1, 57.0, 53.1, 51.1, 50.8, 42.6, 42.3, 40.2, 38.4, 36.8, 33.5, 31.6, 26.9, 26.0, 23.8, 23.6, 21.5, 13.5; HRMS (EI) *m/z*: $[M]^+$ Calcd for $C_{22}H_{35}NO_4$ 377.2561, Found 377.2573. The spectral data of **4** were identical with those reported in the literature.[3,4]

Table S5. Comparison of ^{1}H-NMR chemical shifts between synthetic (–)-cochlearenine (4) and the reported synthetic (±)-cochlearenine by Sarpong and co-workers

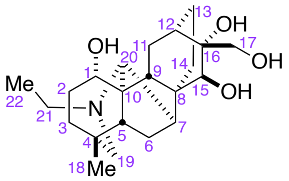

(–)-cochlearenine (**4**)

	Synthetic (–)-cochlearenine (This Work)* ($CDCl_3$, 600 MHz)	Synthetic (±)-cochlearenine (Reported by Sarpong and co-workers) ($CDCl_3$, 500 MHz)[4]
**H-17A**	4.21 (1H, d, *J* = 11.4 Hz)	4.20 (1H, dd, *J* = 11.3, 2.6 Hz)
**H-15**	4.03 (1H, s)	4.01 (1H, d, *J* = 2.1 Hz)
**H-1**	3.83 (1H, dd, *J* = 10.2, 6.0 Hz)	3.83 (1H, dd, *J* = 10.6, 6.6 Hz)
**H-20**	3.72 (1H, s)	3.69 (1H, s)
**H-17B**	3.49 (1H, d, *J* = 11.4 Hz)	3.49 (1H, d, *J* = 11.5 Hz)
**H-13A**	2.62 (1H, dd, *J* = 12.0, 4.8 Hz)	2.64 (1H, dd, *J* = 13.1, 8.7 Hz)
**H-19A**	2.53–2.49 (2H, m)	2.58–2.44 (1H, m)
**H-21A**		2.48 (1H, d, *J* = 10.6 Hz)
**H-21B**	2.40 (1H, dq, *J* = 14.4, 7.2 Hz)	2.39 (1H, dq, *J* = 14.0, 6.9 Hz)
**H-2A**	2.25–2.18 (2H, m)	2.28–2.16 (1H, m)
**H-19B**		2.21 (1H, m)
**H-12**	2.09 (1H, d, *J* = 5.4 Hz)	2.08 (1H, d, *J* = 5.3 Hz)
**H-6A**	2.04–1.90 (3H, m)	2.05–1.88 (4H, m)<br>(*see Note*)
**H-11A**		
**H-14A**		
**H-9**	1.87–1.83 (2H, m)	1.88–1.80 (2H, m)
**H-2B**		
**H-6B**	1.68–1.63 (1H, m)	1.71–1.60 (1H, m)
**H-3A**	1.58 (1H, ddd, *J* = 12.0, 4.2, 4.2 Hz)	1.58 (1H, dt, *J* = 13.3, 3.8 Hz)
**H-7**	1.51 (1H, br s)	1.53–1.49 (1H, br s)
**H-11B**	1.39 (1H, dddd, *J* = 12.0, 12.0, 3.0, 3.0 Hz)	1.37 (1H, t, *J* = 12.2 Hz)
**H-3B**	1.31–1.26 (3H, m)	1.32–1.20 (3H, m)
**H-5**		
**H-13B**		
**H-14B**	1.13 (1H, ddd, *J* = 11.4, 11.4, 1.8 Hz)	1.12 (1H, t, *J* = 12.1 Hz)
**H-22**	1.04 (3H, t, *J* = 7.2 Hz)	1.03 (3H, t, *J* = 7.2 Hz)
**H-18**	0.71 (3H, s)	0.70 (3H, s)

*We assigned the signals of ^{1}H-NMR on the basis of 2D NMR (COSY and HMQC) (Page S94–S95).
Note. Sarpong and co-workers estimated integral values for the signal at δ 2.05–1.88 as 4H. On the other hand, we estimated integral values of 3H for the signal at δ 2.04–1.90. This discrepancy in the integral value would be due to hydrogen atoms corresponding to the hydroxy groups.

Table S6. Comparison of ^{13}C-NMR chemical shifts between synthetic (–)-cochlearenine (4) and the reported synthetic (±)-cochlearenine by Sarpong and co-workers

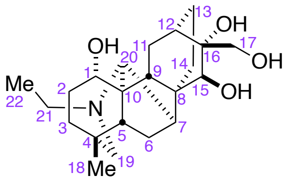

(–)-cochlearenine (**4**)

	Synthetic (–)-cochlearenine (This Work) (150 MHz, $CDCl_3$)	Synthetic (±)-cochlearenine (Sarpong's Report) ($CDCl_3$)[4]	Deviation (our data–Sarpong's data) $\Delta\delta$ (ppm)
**C-15**	87.5	87.8	–0.3
**C-16**	78.8	78.8	0
**C-1**	70.9	71.1	–0.2
**C-17**	67.8	68.0	–0.2
**C-20**	67.1	67.3	–0.2
**C-19**	57.0	57.2	–0.2
**C-5**	53.1	53.3	–0.2
**C-21**	51.1	51.1	0
**C-10**	50.8	50.8	0
**C-8**	42.6	42.8	–0.2
**C-12**	42.3	42.4	–0.1
**C-9**	40.2	40.3	–0.1
**C-3**	38.4	38.5	–0.1
**C-7**	36.8	37.0	–0.2
**C-4**	33.5	33.6	–0.1
**C-2**	31.6	31.9	–0.3
**C-14**	26.9	27.0	–0.1
**C-18**	26.0	26.2	–0.2
**C-13**	23.8	23.8	0
**C-6**	23.6	24.0	–0.4
**C-11**	21.5	21.6	–0.1
**C-22**	13.5	13.8	–0.3

Epoxide 56

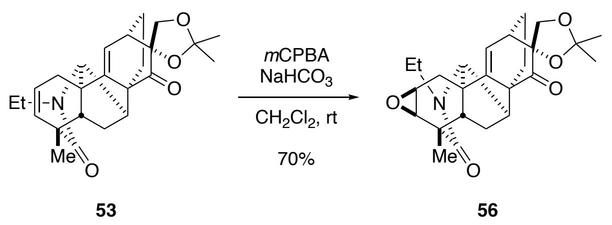

To a solution of diene **53** (16.0 mg, 39.1 μmol) and $NaHCO_3$ (32.8 mg, 0.391 mmol) in CH_2Cl_2 (1.30 mL) was added *m*CPBA (77% purity, 26.3 mg, 0.117 mmol) at room temperature. After stirring for 7 h at room temperature, the reaction was quenched with saturated aqueous $Na_2S_2O_3$ and saturated aqueous Na_2CO_3 at the same temperature, and the mixture was extracted with CH_2Cl_2 three times. The combined organic extracts were washed with brine, dried over anhydrous sodium sulfate, and filtered. The filtrate was concentrated under reduced pressure to remove organic solvents to give a crude material, which was purified by flash silica gel column chromatography (hexane-EtOAc = 2:3) to afford epoxide **56** (11.7 mg, 27.5 μmol, 70%). A white foam; R_f = 0.35 (hexane-EtOAc = 1:2); $[\alpha]_D^{15}$ = +6.84 (*c* 0.170, $CHCl_3$); IR (film): 2988, 2917, 1731, 1644, 1541, 1457, 1373, 1257, 1070, 745 cm^{-1}; ^{1}H-NMR (600 MHz, $CDCl_3$): δ 6.11 (1H, d, *J* = 6.6 Hz), 3.69–3.65 (2H, m), 3.59 (1H, dq, *J* = 14.4, 7.2 Hz), 3.24 (2H, s), 3.18 (1H, dq, *J* = 14.4, 7.2 Hz), 3.16 (1H, s), 2.91 (1H, ddd, *J* = 6.6, 3.0, 3.0 Hz), 2.57 (1H, d, *J* = 17.4 Hz), 2.46 (1H, d, *J* = 5.4 Hz), 2.16–2.12 (1H, m), 2.05 (1H, d, *J* = 17.4 Hz), 1.96 (1H, ddd, *J* = 14.4, 5.4, 1.8 Hz), 1.84 (1H, ddd, *J* = 12.6, 10.2, 4.2 Hz), 1.75 (1H, ddd, *J* = 8.4, 1.8, 1.8 Hz), 1.63 (1H, ddd, *J* = 14.4, 5.4, 1.8 Hz), 1.52 (3H, s), 1.45 (3H, s), 1.42 (1H, dd, *J* = 12.6, 4.2 Hz), 1.39–1.36 (1H, m), 1.34 (3H, s), 1.13 (3H, t, *J* = 7.2 Hz); ^{13}C-NMR (150 MHz, $CDCl_3$): δ 209.7, 171.2, 149.1, 121.5, 112.2, 83.9, 72.1, 60.9, 58.1, 51.2, 46.3, 45.8, 42.3, 41.9, 40.1, 36.2, 27.5, 27.1, 26.6, 26.4, 25.8, 24.3, 20.1, 19.1, 13.5; HRMS (ESI) *m/z*: $[M+H]^+$ Calcd for $C_{25}H_{32}NO_5$ 426.2275, Found 426.2262.

1,2-Diol 58

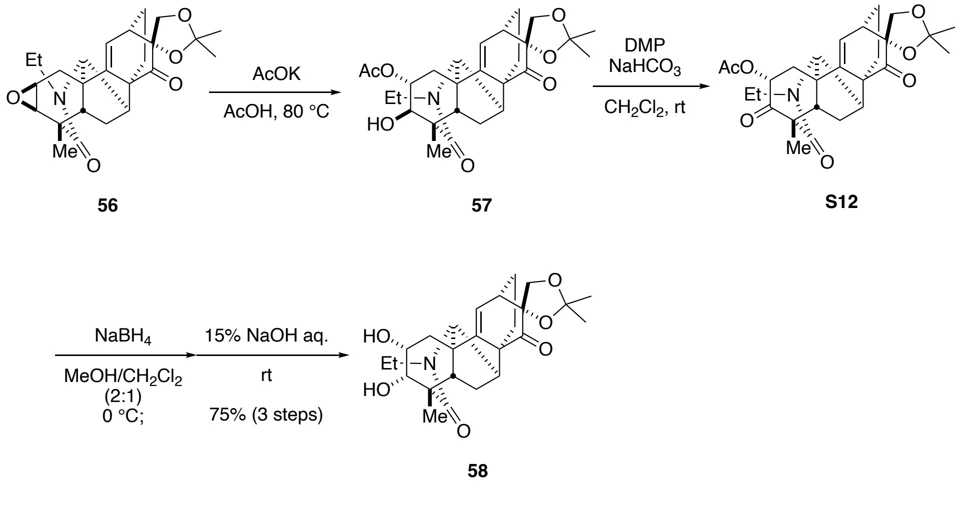

To a solution of epoxide **56** (11.7 mg, 27.5 μmol) in AcOH (920 μL) was added AcOK (81.0 mg, 0.825 mmol) at room temperature. After stirring for 10 h at 80 °C, the reaction was quenched with saturated aqueous $NaHCO_3$ at room temperature, and the mixture was extracted with CH_2Cl_2 three times. The combined organic extracts were washed with brine, dried over anhydrous sodium sulfate, and filtered. The filtrate was concentrated

under reduced pressure to remove organic solvents to give a residue, which was passed through a short column of silica gel using EtOAc as an eluent. The eluate was concentrated under reduced pressure to give a crude **57**, which was used for the next reaction without further purification.

To the solution of the crude **57** and $NaHCO_3$ (18.8 mg, 0.224 mmol) in CH_2Cl_2 (11 mL) was added Dess–Martin periodinane (28.5 mg, 67.2 μmol) at room temperature. After stirring for 1 h at room temperature, the reaction was quenched with saturated aqueous $Na_2S_2O_3$ at the same temperature, and the mixture was extracted with CH_2Cl_2 three times. The combined organic extracts were washed with brine, dried over anhydrous sodium sulfate, and filtered. The filtrate was concentrated under reduced pressure to remove organic solvents to give a residue, which was passed through a short column of silica gel using 66% EtOAc in hexane as a eluent. The eluate was concentrated under reduced pressure to give a crude **S12**, which was used for the next reaction without further purification.

To the solution of the crude **S12** in MeOH (700 μL) and CH_2Cl_2 (350 μL) was added $NaBH_4$ (8.0 mg, 0.211 mmol) at –10 °C. After stirring for 0.5 h at –10 °C, 15 % aqueous NaOH was added and stirred for 1 h at room temperature. The resulting mixture was diluted with CH_2Cl_2 and extracted with CH_2Cl_2 three times. The combined organic extracts were washed with brine, dried over anhydrous sodium sulfate, and filtered. The filtrate was concentrated under reduced pressure to remove organic solvents to give a crude material, which was purified by flash silica gel column chromatography (CH_2Cl_2-MeOH = 20:1 to 15:1) to afford 1,2-diol **58** (9.2 mg, 20.7 μmol, 75%, 3 steps). A white foam; R_f = 0.45 (CH_2Cl_2-MeOH = 10:1); $[\alpha]_D^{15}$ = +62.1 (*c* 0.400, $CHCl_3$); IR (film): 3442, 2976, 2933, 1728, 1618, 1370, 1264, 1212, 1074, 755 cm^{-1}; ^1H-NMR (600 MHz, $CDCl_3$): δ 6.07 (1H, d, *J* = 7.2 Hz), 4.11–4.09 (1H, m), 3.69 (2H, s), 3.42–3.38 (2H, m), 3.34 (1H, dq, *J* = 14.4, 7.2 Hz), 3.30 (1H, s), 2.90 (1H, ddd, *J* = 7.2, 3.0, 3.0 Hz), 2.53 (1H, d, *J* = 5.4 Hz), 2.22–2.14 (3H, m), 2.00 (1H, ddd, *J* = 14.4, 8.4, 1.2 Hz), 1.87 (1H, ddd, *J* = 14.4, 9.6, 2.4 Hz), 1.73 (1H, ddd, *J* = 14.4, 5.4, 2.4 Hz), 1.52 (3H, s), 1.49–1.44 (2H, m), 1.45 (3H, s), 1.39 (1H, dddd, *J* = 12.0, 12.0, 4.2, 2.4, Hz), 1.26 (3H, s), 1.13 (3H, t, *J* = 7.2 Hz); ^{13}C-NMR (150 MHz, $CDCl_3$): δ 209.7, 172.9, 150.4, 121.3, 112.2, 83.8, 77.6, 72.1, 68.7, 67.1, 57.8, 47.7, 46.68, 46.67, 43.9, 43.2, 41.9, 30.5, 27.2, 27.1, 25.9, 20.2, 18.9, 12.4 (One signal is missing due to overlap); HRMS (ESI) *m/z*: $[M+H]^+$ Calcd for $C_{25}H_{34}NO_6$ 444.2381, Found 444.2377.

(–)-Macrocentrine (8)

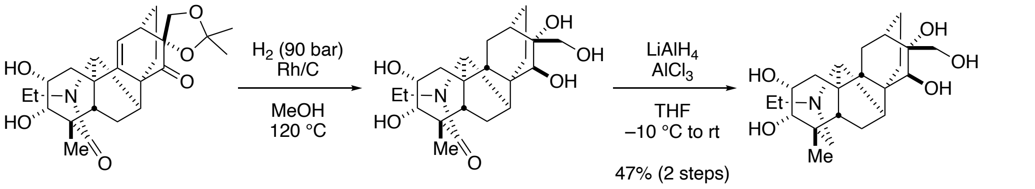

58 **S13** (–)-macrocentrine (**8**)

$[\alpha]_D^{18}$ = –10.1 (c 0.20, MeOH)
$[\alpha]_D$ is not reported in the literature.

A suspension of 1,2-diol **58** (4.8 mg, 10.8 μmol) and 5% Rh/C (55% suspension in H_2O) (40.5 mg, 10.8 μmol) in MeOH (1.0 mL) was placed in a Parr Pressure Vessel and pressurized hydrogen gas (90 bar) at room temperature. After stirring for 8.5 h at 120 °C, the hydrogen pressure was released, and the reaction mixture was filtered through a pad of Celite and the filter cake was washed with MeOH. The combined filtrate and washings

were concentrated under reduced pressure to give a crude **S13**, which was used for the next reaction without further purification.

To a solution of the crude **S13** and $AlCl_3$ (8.2 mg, 61.3 μmol) in THF (400 μL) was added $LiAlH_4$ (7.0 mg, 0.184 mmol) at –10 °C. After stirring for 3 h at room temperature, the reaction was quenched by slow addition of H_2O (7.0 μL), 15% aqueous NaOH (7.0 μL), and H_2O (21.0 μL) sequentially at –10 °C. The resulting suspension was diluted with 15% aqueous NaOH at room temperature and extracted with 33% EtOH in $CHCl_3$ five times. The combined organic extracts were washed with brine, dried over anhydrous sodium sulfate, and filtered. The filtrate was concentrated under reduced pressure to remove organic solvents to give a crude material, which was purified by preparative TLC (EtOAc-MeOH-NH_4OH = 24:2:1) to afford (–)-macrocentrine (**8**) (2.0 mg, 5.1 μmol, 47%, 2 steps). A white foam.; R_f = 0.20 (CH_2Cl_2-MeOH-NH_4OH = 88:10:2); $[\alpha]_D^{18}$ = –10.1 (*c* 0.200, MeOH); IR (film): 2955, 2921, 2846, 1651, 1538, 1502, 1457, 1338, 1219, 770 cm^{-1}; ^{1}H-NMR (600 MHz, CD_3OD): δ 3.97 (1H, d, *J* = 12.0 Hz), 3.91 (1H, s), 3.77 (1H, d, *J* = 1.8 Hz), 3.59 (1H, d, *J* = 12.0 Hz), 3.34 (1H, s), 3.22 (1H, d, *J* = 4.2 Hz), 2.87 (1H, dd, *J* = 14.4, 8.4 Hz), 2.81 (1H, d, *J* = 12.0 Hz), 2.67–2.57 (2H, m), 2.14 (1H, d, *J* = 6.0 Hz), 2.01–1.84 (6H, m), 1.68–1.65 (2H, m), 1.32–1.30 (2H, m), 1.28–1.26 (2H, m), 1.24–1.18 (1H, m), 1.11 (3H, t, *J* = 7.2 Hz), 0.81 (3H, s); ^{13}C-NMR (150 MHz, CD_3OD): δ 86.7, 81.1, 77.4, 70.5, 69.7, 67.8, 53.4, 50.9, 50.1, 47.0, 44.3, 43.2, 41.7, 40.0, 36.4, 33.4, 29.1, 24.7, 23.4, 22.8, 22.5, 12.8; ^{13}C-NMR (150 MHz, C_6D_5N): δ 86.5, 80.4, 76.7, 70.3, 68.5, 67.4, 52.3, 50.3, 49.5, 46.1, 43.8, 42.4, 41.1, 39.4, 36.2, 32.9, 28.8, 24.4, 22.9, 22.8, 22.7, 12.6; HRMS (ESI) *m/z*: $[M+H]^+$ Calcd for $C_{22}H_{36}NO_5$ 394.2588, Found 393.2594. The spectral data of **8** were identical with those reported in the literature.[5]

Table S7. Comparison of ^{1}H-NMR chemical shifts between synthetic (–)-macrocentrine (8) and the reported natural (–)-macrocentrine (8)[5]

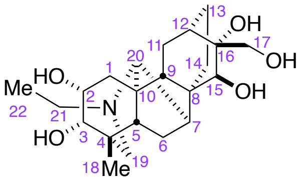

(–)-macrocentrine (**8**)

	Synthetic (–)-Macrocentrine (CD_3OD, 600 MHz)	Natural (–)-Macrocentrine (CD_3OD, 200 MHz)[5*]
**H-17A**	3.97 (1H, d, *J* = 12.0 Hz)	4.00 (1H, d, *J* = 11.5 Hz)
**H-15**	3.91 (1H, s)	3.90 (1H, s)
**H-2**	3.77 (1H, d, *J* = 1.8 Hz)	3.76 (1H, m)
**H-17B**	3.59 (1H, d, *J* = 12.0 Hz)	3.52 (1H, d, *J* = 11.5 Hz)
**H-20**	3.34 (1H, s)	*Not reported*
**H-3**	3.22 (1H, *J* = 4.2 Hz)	3.19 (1H, d, *J* = 4.5 Hz)
**H-6A**	2.87 (1H, dd, *J* = 14.4, 8.4 Hz)	*Not reported*
**H-19A**	2.81 (1H, d, *J* = 12.0 Hz)	
**H-21A**	2.67–2.57 (2H, m)	
**H-21B**		
**H-7**	2.14 (1H, d, *J* = 6.0 Hz)	
**H-12**	2.09 (1H, d, *J* = 5.4 Hz)	
**H-1A**	2.01–1.84 (6H, m)	
**H-1B**		
**H-9**		
**H-13A**		
**H-14B**		
**H-19B**		
**H-11A**	2.67–2.57 (2H, m)	
**H-12**		
**H-11B**	1.32–1.30 (2H, m)	
**H-13B**		
**H-5**	1.28–1.26 (2H, m)	
**H-6B**		
**H-14B**	1.24–1.18 (1H, m)	
**H-22**	1.11 (3H, t, *J* = 7.2 Hz)	1.11 (3H, t, *J* = 7.0 Hz)
**H-18**	0.81 (3H, s)	0.81 (3H, s)

*We assigned the signals of ^{1}H-NMR on the basis of 2D NMR (COSY, HMQC, and HMBC) (Page S100–S102).

Table S8. Comparison of ^{13}C-NMR chemical shifts between synthetic (–)-macrocentrine (8) and the reported natural (–)-macrocentrine (8)[5]

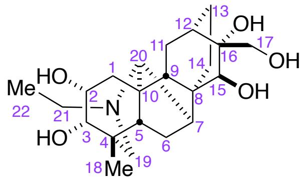

(–)-macrocentrine (**8**)

	Synthetic (–)-macrocentrine (150 MHz, C_5D_5N)	Natural (–)-macrocentrine (50 MHz, C_5D_5N)[5]	Deviation (synthetic–natural) $\Delta\delta$ (ppm)
**C-15**	86.5	86.4	0.1
**C-16**	80.4	80.4	0.0
**C-20**	76.7	76.8	–0.1
**C-2**	70.3	70.1	0.2
**C-3**	68.5	68.5	0.0
**C-17**	67.4	67.3	0.1
**C-9**	52.3	52.5	–0.2
**C-19**	50.3	50.1	0.2
**C-21**	49.5	49.4	0.1
**C-10**	46.1	46.0	0.1
**C-12**	43.8	43.6	0.2
**C-8**	42.4	42.4	0.0
**C-5**	41.1	41.0	0.1
**C-4**	39.4	39.3	0.1
**C-7**	36.2	36.1	0.1
**C-1**	32.9	33.0	–0.1
**C-6**	28.8	28.7	0.1
**C-13**	24.4	24.4	0.0
**C-11**	22.9	22.8	0.1
**C-18**	22.8	22.8	0.0
**C-14**	22.7	22.6	0.1
**C-22**	12.6	12.7	–0.1

Amide S14

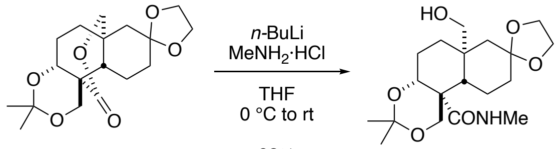

34 **S14**

To a suspension of $MeNH_2{\cdot}HCl$ (3.78 g, 56.0 mmol) in THF (37 mL) was added *n*-BuLi (2.40 M in *n*-hexane, 42.0 mL, 101 mmol) at 0 °C. After stirring for 50 min at room temperature, lactone **34** (3.80 g, 11.2 mmol) in THF (37 mL) was added at 0 °C. After stirring for 1 h at room temperature, the reaction was quenched with H_2O at 0 °C, and the mixture was extracted with CH_2Cl_2 five times. The combined organic extracts were washed with brine, dried over anhydrous sodium sulfate, and filtered. The filtrate was concentrated under reduced pressure to remove organic solvents to give a crude material, which was purified by flash silica gel column chromatography (hexane-acetone = 3:2 to 1:1) to afford amide **S14** (3.85 g, 10.4 mmol, 93%). A white foam; R_f = 0.29 (100% EtOAc); mp: 115–118 °C (hexane-CH_2Cl_2); $[\alpha]_D^{18}$ = +35.1 (*c* 0.500, $CHCl_3$); IR (film): 3391, 2985, 2943, 2878, 1654, 1544, 1370, 1197, 1096, 1051 cm^{-1}; ^{1}H-NMR (400 MHz, $CDCl_3$): δ 7.33–7.30 (1H, m), 4.13 (1H, d, *J* = 10.8 Hz), 4.01–3.84 (5H, m), 3.76 (1H, dd, *J* = 12.0, 4.0 Hz), 3.59 (1H, d, *J* = 10.8 Hz), 3.34 (1H, dd, *J* = 10.8, 10.8 Hz), 2.83 (3H, d, *J* = 4.8 Hz), 2.55 (1H, dddd, *J* = 14.0, 14.0, 14.0, 4.0 Hz), 2.18–2.04 (3H, m), 1.91–1.87 (2H, m), 1.62–1.57 (1H, m), 1.49 (3H, s), 1.40 (3H, s), 1.34–1.25 (2H, m), 1.14 (1H, d, *J* = 14.0 Hz), 1.11–1.02 (1H, m); ^{13}C-NMR (100 MHz, $CDCl_3$): δ 173.8, 108.3, 99.9, 76.4, 70.7, 64.6, 63.5, 60.4, 49.2, 46.2, 44.1, 40.1, 35.0, 34.6, 29.7, 25.8, 24.5, 20.6, 18.5; HRMS (ESI) *m/z*: $[M+H]^+$ Calcd for $C_{19}H_{32}NO_6$ 370.2224, Found 370.2234.

Hemiaminal S15

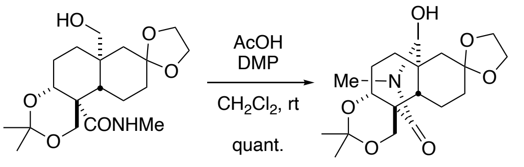

S14 **S15**

To a solution of amide **S14** (2.00 g, 5.41 mmol) and Dess–Martin periodinane (3.44 g, 8.12 mmol) in CH_2Cl_2 (54 mL) was added AcOH (617 μL, 10.8 mmol) at room temperature. After stirring for 3 h at room temperature, the reaction was quenched with saturated aqueous $Na_2S_2O_3$ at room temperature, and the mixture was extracted with CH_2Cl_2 three times. The combined organic extracts were washed with brine, dried over anhydrous sodium sulfate, and filtered. The filtrate was concentrated under reduced pressure to remove organic solvents to give a crude material, which was purified by flash silica gel column chromatography (hexane-acetone = 1:1 to 2:3) to afford hemiaminal **S15** (1.99 g, 5.41 mmol, quant.). A white solid; R_f = 0.19 (hexane-acetone = 1:1); mp: 215–218 °C (hexane-CH_2Cl_2-MeOH); $[\alpha]_D^{18}$ = +19.4 (*c* 1.00, MeOH); IR (film): 3384, 2943, 2878, 1637, 1380, 1303, 1260, 1164, 1106, 1041 cm^{-1}; ^{1}H-NMR (400 MHz, CD_3OD): δ 5.48 (1H, s), 4.04 (1H, d, *J* = 11.6 Hz), 4.02–3.91 (3H, m), 3.87–3.83 (2H, m), 3.68 (1H, d, *J* = 11.6 Hz), 2.91 (3H, s), 2.27 (1H, ddd, *J* = 13.2, 3.2, 3.2 Hz), 1.93 (1H, dd, *J* = 14.4, 2.4 Hz), 1.82 (1H, dddd, *J* = 13.2, 3.2, 3.2, 3.2 Hz), 1.69–

1.53 (3H, m), 1.51–1.45 (2H, m), 1.48 (3H, s), 1.37 (3H, s), 1.25 (1H, d, J = 14.4 Hz), 1.14 (2H, dddd, J = 13.2, 13.2, 13.2, 3.2 Hz); ^{13}C-NMR (150 MHz, CD_3OD): δ 171.8, 109.3, 100.1, 82.6, 75.9, 66.5, 65.7, 64.5, 46.9, 45.3, 43.4, 40.1, 35.5, 34.0, 30.7, 29.5, 27.4, 20.7, 19.6; HRMS (ESI) *m/z*: $[M+H]^+$ Calcd for $C_{19}H_{30}NO_6$ 368.2068, Found 368.2081.

Ketone S16

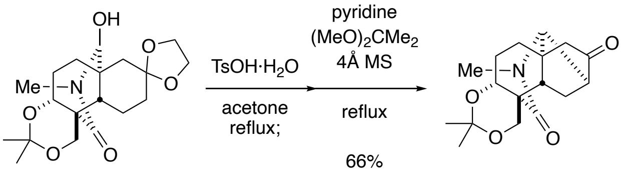

S15 S16

To a solution of hemiaminal **S15** (1.71 g, 4.65 mmol) in acetone (93 mL) was added TsOH·H_2O (885 mg, 4.65 mmol) at room temperature. After stirring for 18.5 h at 86 °C, pyridine (375 μL, 4.65 mmol) was added at room temperature, and the mixture was stirred for 2 h at the same temperature. To the resulting mixture were added 4Å MS (6.48 g) and 2,2-dimethoxypropane (5.70 mL, 46.5 mmol) at the same temperature, and then the mixture was allowed to warm up to 86 °C and stirred for 35 h. The resulting suspension was filtered through a pad of basic alumina and the filter cake was washed with acetone. The combined filtrate and washings were concentrated under reduced pressure to give a crude material, which was purified by flash silica gel column chromatography (hexane-acetone = 1:1) to afford ketone **S16** (935 mg, 3.06 mmol, 66%). A white solid; R_f = 0.35 (hexane-acetone = 1:1); mp: 204–207 °C (hexane-CH_2Cl_2); $[\alpha]_D^{18}$ = –6.10 (*c* 0.500, $CHCl_3$); IR (film): 2991, 2943, 1750, 1647, 1457, 1396, 1260, 1209, 1080, 871 cm^{-1}; ^{1}H-NMR (400 MHz, $CDCl_3$): δ 4.35 (1H, d, J = 11.6 Hz), 3.85 (1H, dd, J = 12.0, 4.4 Hz), 3.57 (1H, d, J = 11.6 Hz), 3.37 (1H, s), 3.00 (3H, s), 2.77 (1H, d, J = 5.2 Hz), 2.20–2.12 (3H, m), 1.98–1.70 (6H, m), 1.54 (3H, s), 1.45 (3H, s); ^{13}C-NMR (100 MHz, $CDCl_3$): δ 210.6, 169.1, 99.5, 71.9, 69.2, 64.4, 54.4, 48.8, 48.6, 43.9, 43.4, 34.1, 27.8, 26.8, 26.1, 26.0, 21.4; HRMS (ESI) *m/z*: $[M+H]^+$ Calcd for $C_{17}H_{24}NO_4$ 306.1700, Found 306.1694.

Enol triflate S17

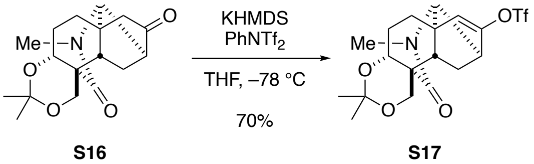

S16 S17

To a solution of ketone **S16** (935 mg, 3.06 mmol) and $PhNTf_2$ (2.73 g, 7.65 mmol) in THF (31 mL) was added KHMDS (0.500 M in toluene, 15.3 mL, 7.65 mmol) at –78 °C. After stirring for 2 h –78 °C, the reaction was quenched with H_2O at the same temperature, and the mixture was extracted with CH_2Cl_2 three times. The combined organic extracts were washed with brine, dried over anhydrous sodium sulfate, and filtered. The filtrate was concentrated under reduced pressure to remove organic solvents to give a crude material, which was purified by flash silica gel column chromatography (hexane-acetone = 3:1 to 5:2) to afford enol triflate **S17** (943 mg, 2.16 mmol, 70%). A colorless oil; R_f = 0.62 (hexane-acetone = 1:1); $[\alpha]_D^{18}$ = –27.1 (*c* 1.98, $CHCl_3$); IR (film): 3413, 2943, 2885, 1631, 1421, 1209, 1142, 1058, 822, 609 cm^{-1}; ^{1}H-NMR (400 MHz, $CDCl_3$): δ 5.44

(1H, d, J = 1.6 Hz), 4.29 (1H, d, J = 11.6 Hz), 3.86 (1H, dd, J = 12.0, 4.4 Hz), 3.61 (1H, d, J = 11.6 Hz), 3.17 (1H, s), 2.95–2.94 (1H, m), 2.92 (3H, s), 2.32 (1H, dd, J = 14.0, 4.8 Hz), 2.09 (1H, ddd, J = 14.0, 14.0, 4.8 Hz), 1.85–1.79 (3H, m), 1.77–1.57 (2H, m), 1.52 (3H, s), 1.44 (3H, s); ^{13}C-NMR (150 MHz, $CDCl_3$): δ 169.4, 153.5, 121.0, 118.4 (q, ^1J = 320 kHz), 99.3, 76.1, 72.0, 65.6, 50.0, 48.2, 47.3, 41.9, 33.5, 29.6, 27.1, 26.7, 26.1, 21.1; HRMS (ESI) *m/z*: $[M+H]^+$ Calcd for $C_{18}H_{23}F_3NO_6S$ 438.1193, Found 438.1184.

Ketol S18

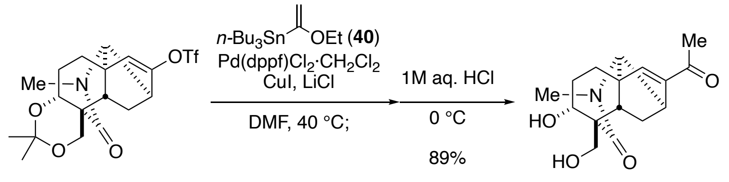

S17 **S18**

To a solution of enol triflate **S17** (910 mg, 2.08 mmol), Pd(dppf)Cl_2·CH_2Cl_2 (170 mg, 0.208 mmol), CuI (39.6 mg, 0.208 mmol) and LiCl (441 mg, 10.4 mmol) in DMF (21 mL) was added tributyl(1-ethoxyvinyl)tin (**40**) (828 μL, 2.50 mmol) at room temperature. The reaction mixture was degassed by three cycles of freeze-pump-thaw cycle, and the reaction mixture was stirred at 40 °C for 2.5 h, and added 1M aqueous HCl (21 mL) at 0 °C. After stirring for 2 h at 0 °C, the reaction was added $NaHCO_3$ and NaCl, then the reaction mixture was filtered through a pad of Celite and the filter cake was washed with $CHCl_3$. The filtrate was extracted with $CHCl_3$ five times. The combined organic extracts were washed with brine, dried over anhydrous sodium sulfate, and filtered. The filtrate was concentrated under reduced pressure to remove organic solvents to give a crude material, which was purified by flash silica gel column chromatography (EtOAc-MeOH = 9:1 to 6:1) to afford ketol **S18** (542 mg, 1.86 mmol, 89%). A white foam; R_f = 0.17 (EtOAc-MeOH = 9:1); $[\alpha]_D^{18}$ = –10.4 (*c* 2.21, $CHCl_3$); IR (film): 3407, 2940, 1657, 1622, 1399, 1228, 1051, 1029, 748, 629 cm^{-1}; ^{1}H-NMR (400 MHz, $CDCl_3$): δ 6.48 (1H, s), 4.36 (1H, d, J = 11.6 Hz), 4.09 (1H, br s), 3.90 (2H, br d, J = 5.2 Hz), 3.68 (1H, dd, J = 11.2, 8.8 Hz), 3.30 (1H, d, J = 4.4 Hz), 3.05 (1H, s), 2.93 (3H, s), 2.29 (1H, dd, J = 14.4, 5.6 Hz), 2.25 (3H, s), 2.13–2.00 (2H, m), 1.81 (1H, dd, J = 12.4, 3.6 Hz), 1.69–1.67 (1H, m), 1.61–1.45 (2H, m); ^{13}C-NMR (100 MHz, $CDCl_3$): δ 194.1, 171.5, 149.5, 146.4, 76.7, 73.3, 65.9, 52.8, 51.3, 44.1, 38.5, 33.2, 31.2, 28.6, 26.0, 25.4; HRMS (ESI) *m/z*: $[M+H]^+$ Calcd for $C_{16}H_{22}NO_4$ 292.1543, Found 292.1545.

Phenol S21

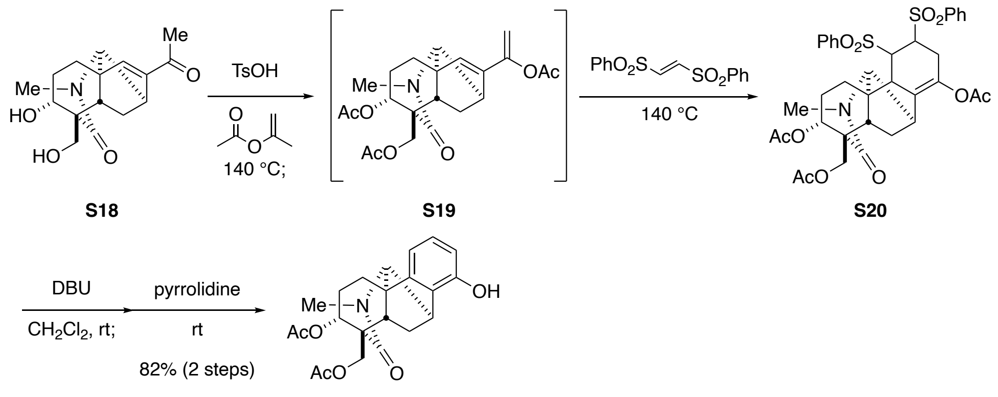

S18 **S19** **S20**

S21

To a suspension of 1,3-diol **S18** (542 mg, 1.86 mmol) in isopropenyl acetate (19 mL) was added TsOH (96.1 mg, 0.558 mmol) at room temperature. After stirring for 3 h at 140 °C, *trans*-1,2-bis(phenylsulfonyl)ethylene (860 mg, 2.79 mmol) was added at room temperature, and the mixture was stirred for 19 h at 140 °C. The solvent was removed under reduced pressure. The residue was roughly purified by flash silica gel column chromatography (hexane-acetone = 2:1 to 1:1) to afford a diastereomeric mixture of **S20** containing inseparable impurities, which was used for the next reaction without further purification.

To the solution of the crude **S20** in CH_2Cl_2 (37 mL) was added DBU (1.67 mL, 11.2 mmol) at the same temperature. After stirring for 5.5 h at room temperature, pyrrolidine (1.53 mL, 18.6 mmol) was added, and the mixture was stirred for 2 h at room temperature. The reaction was quenched with 2 M aq. HCl at room temperature, and the mixture was extracted with CH_2Cl_2 three times. The combined organic extracts were washed with brine, dried over anhydrous sodium sulfate, and filtered. The filtrate was concentrated under reduced pressure to remove organic solvents to give a crude material, which was purified by flash silica gel column chromatography (hexane-acetone = 1:1) to afford phenol **S21** (613 mg, 1.53 mmol, 82%, 2 steps). A white solid; R_f = 0.41 (hexane-acetone = 1:1); mp: 256–259 °C (hexane-CH_2Cl_2); $[\alpha]_D^{18}$ = –16.1 (*c* 1.00, $CHCl_3$); IR (film): 2946, 2878, 1738, 1631, 1593, 1470, 1367, 1241, 1038, 755 cm^{-1}; ^{1}H-NMR (400 MHz, $CDCl_3$): δ 7.09 (1H, dd, *J* = 8.4, 8.4 Hz), 6.77 (1H, d, *J* = 8.4 Hz), 6.70 (1H, d, *J* = 8.4 Hz), 5.36 (1H, br s), 5.06 (1H, dd, *J* = 12.0, 4.4 Hz), 4.85 (1H, d, *J* = 11.6 Hz), 4.13 (1H, d, *J* = 11.6 Hz), 3.58 (1H, d, *J* = 4.0 Hz), 3.25 (1H, s), 3.02 (3H, s), 2.50 (1H, ddd, *J* = 14.0, 14.0, 5.6 Hz), 2.29 (1H, ddd, *J* = 14.4, 4.0, 0.8 Hz), 2.19–2.13 (1H, m), 2.11 (3H, s), 1.99 (3H, s), 1.98–1.80 (3H, m), 1.59 (1H, dd, *J* = 13.2, 7.6 Hz); ^{13}C-NMR (100 MHz, $CDCl_3$): δ 170.82, 170.76, 168.2, 149.4, 146.6, 129.9, 127.6, 114.7, 112.6, 78.1, 71.7, 61.8, 52.0, 48.8, 41.9, 39.7, 33.9, 32.0, 25.6, 24.3, 21.1, 20.8; HRMS (ESI) *m/z*: $[M+H]^+$ Calcd for $C_{22}H_{26}NO_6$ 400.1755, Found 400.1765.

Diol S22

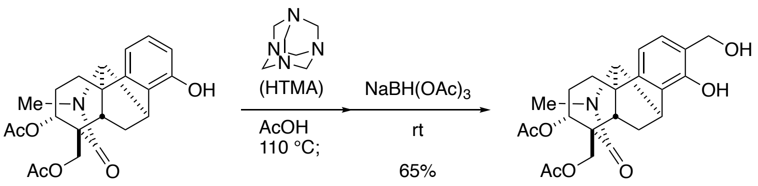

S21 **S22**

To a solution of phenol **S21** (297 mg, 0.743 mmol) in AcOH (15 mL) was added HTMA (125 mg, 0.892 mmol) at room temperature. After stirring for 15 h at 110 °C, additional amount of HTMA (20.8 mg, 0.149 mmol) was added at room temperature. After stirring for 6.5 h at 110 °C, 2 M aq. HCl (10 mL) was added at 0 °C, and the mixture was stirred for 1 h at room temperature. To the resulting mixture were added $NaHCO_3$ (1.68 g, 20.0 mmol) and $NaBH(OAc)_3$ (3.15 g, 14.9 mmol) at 0 °C. After stirring for 15.5 h at room temperature, the reaction mixture was diluted with CH_2Cl_2 and extracted with CH_2Cl_2 three times. The combined organic extracts were washed with brine, dried over anhydrous sodium sulfate, and filtered. The filtrate was concentrated under reduced pressure to remove organic solvents to give a crude material, which was purified by flash silica gel column chromatography (hexane-acetone = 1:1) to afford diol **S22** (209 mg, 0.487 mmol, 65%). A white foam; R_f = 0.21 (hexane-acetone = 1:1); $[\alpha]_D^{18}$ = +5.20 (*c* 1.20, $CHCl_3$); IR (film): 2940, 2875, 1735, 1634, 1454, 1373, 1235, 1212, 1038, 748 cm^{-1}; ^{1}H-NMR (400 MHz, $CDCl_3$): δ 7.85 (1H, br s), 6.89 (1H, d, *J* = 7.2

Hz), 6.67 (1H, d, *J* = 7.2 Hz), 5.04 (1H, dd, *J* = 12.0, 4.4 Hz), 4.88 (2H, s), 4.83 (1H, d, *J* = 11.6 Hz), 4.09 (1H, d, *J* = 11.6 Hz), 3.63 (1H, dd, *J* = 4.4, 1.2 Hz), 3.22 (1H, s), 2.99 (3H, s), 2.48 (1H, ddd, *J* = 14.0, 14.0, 6.0 Hz), 2.27 (1H, ddd, *J* = 14.0, 6.0, 1.2 Hz), 2.16–2.11 (1H, m), 2.10 (3H, s), 1.96 (3H, s), 1.94–1.78 (3H, m), 1.58 (1H, dd, *J* = 12.8, 7.2 Hz); ^{13}C-NMR (100 MHz, $CDCl_3$): δ 170.7, 170.6, 168.0, 149.6, 146.6, 131.0, 125.9, 124.0, 112.1, 78.1, 71.6, 64.9, 61.8, 52.0, 48.8, 41.8, 39.8, 33.7, 31.9, 25.6, 24.3, 21.2, 20.8; HRMS (ESI) *m/z*: $[M+H]^+$ Calcd for $C_{23}H_{28}NO_7$ 430.1860, Found 430.1850.

1,2-Diol S25

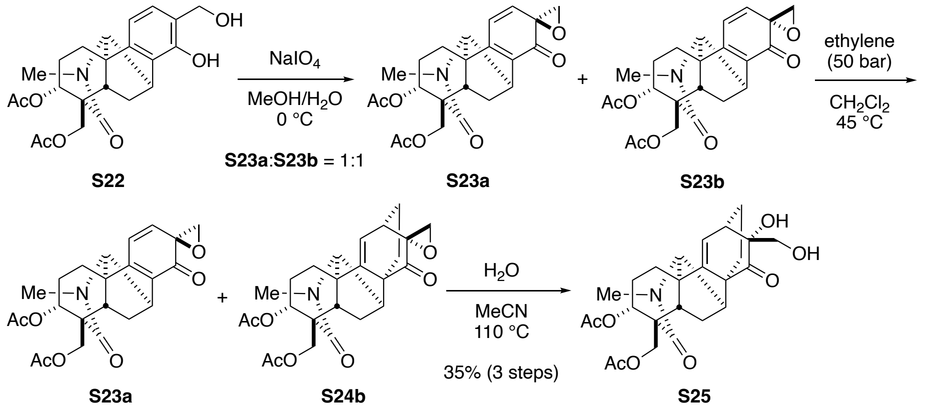

To a solution of diol **S22** (422 mg, 0.983 mmol) in MeOH (10 mL) was added $NaIO_4$ (2.10 g, 9.83 mmol) in H_2O (19 mL) at 0 °C. After stirring for 30 min at 0 °C, the reaction mixture was diluted with H_2O at the same temperature, and the mixture was extracted with CH_2Cl_2 three times. The combined organic extracts were washed with brine, dried over anhydrous sodium sulfate, and filtered. The filtrate was concentrated under reduced pressure to remove organic solvents to give a crude material consisting of **S23a** and **S23b** as a 1:1 ratio, which was used for the next reaction without further purification.

A solution of the crude **S23a** and **S23b** in CH_2Cl_2 (11 mL) was placed in a Parr Pressure Vessel, which was then pressurized with ethylene gas (50 bar) at room temperature. After stirring for 133 h at 45 °C, ethylene pressure was released, and the resulting reaction mixture was concentrated under reduced pressure to remove the solvent. The residue was roughly purified by flash silica gel column chromatography (hexane-acetone = 3:2 to 1:1) to afford an inseparable mixture of cycloadduct **S24b** and unreacted **S23a**, which was used for the next reaction without further purification.

To a solution of a mixture of **S24b** and **S23a** in MeCN (5 mL) was added H_2O (10 mL) at room temperature. After stirring for 48 h at 110 °C, the solvents were removed under reduced pressure to give a crude material, which was purified by flash silica gel column chromatography (hexane-acetone = 1:1 to 1:2) to afford 1,2-diol **S25** (172 mg, 0.363 mmol, 35%, 3 steps). A white foam; R_f = 0.14 (hexane-acetone = 1:1); $[\alpha]_D^{18}$ = +30.1 (*c* 0.400, $CHCl_3$); IR (film): 3456, 2959, 1735, 1641, 1464, 1377, 1245, 1044, 861, 751 cm^{-1}; ^{1}H-NMR (400 MHz, $CDCl_3$): δ 6.13 (1H, d, *J* = 6.4 Hz), 4.92 (1H, dd, *J* = 12.0, 4.4 Hz), 4.84 (1H, *J* = 12.0 Hz), 3.92 (1H, d, *J* = 12.0 Hz), 3.42 (2H, s), 3.34 (1H, s), 3.18 (1H, br s), 2.99 (3H, s), 2.98 (1H, ddd, *J* = 6.4, 4.0, 4.0 Hz), 2.62 (1H, d, *J* = 5.2 Hz), 2.29–2.15 (3H, m), 2.13 –2.03 (2H, m), 2.99 (3H, s), 2.98 (3H, s), 1.95–1.67 (5H, m), 1.43 (1H, ddd, *J* = 12.0, 12.0, 4.0 Hz), 1.32 (1H, dddd, *J* = 12.0, 12.0, 4.0, 2.8 Hz); ^{13}C-NMR (150 MHz, $CDCl_3$): δ 213.3,

170.6, 170.5, 167.5, 148.2, 121.9, 71.7, 71.1, 66.1, 60.4, 57.9, 52.1, 48.1, 42.1, 41.6, 40.4, 34.1, 27.8, 27.4, 25.2, 24.2, 21.1, 20.9, 19.7 (One signal is missing due to overlap); HRMS (ESI) *m/z*: $[M+H]^+$ Calcd for $C_{25}H_{32}NO_8$ 474.2122, Found 474.2104.

1,3-Diol S26

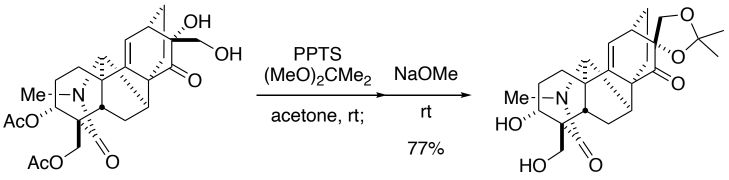

S25 **S26**

To a solution of 1,2-diol **S25** (109 mg, 0.230 mmol) and PPTS (11.6 mg, 46.0 μmol) in acetone (10 mL) was added 2,2-dimethoxypropane (282 μL, 2.30 mmol) at room temperature. After stirring for 12.5 h at room temperature, NaOMe (124 mg, 2.30 mmol) was added, and the mixture was stirred for 2 h at room temperature. The reaction was quenched with H_2O, and the mixture was extracted with $CHCl_3$ five times. The combined organic extracts were washed with brine, dried over anhydrous sodium sulfate, and filtered. The filtrate was concentrated under reduced pressure to remove organic solvents to give a crude material, which was purified by flash silica gel column chromatography (CH_2Cl_2-MeOH = 20:1) to afford 1,3-diol **S26** (75.9 mg, 0.177 mmol, 77%). A white foam; R_f = 0.22 (CH_2Cl_2-MeOH = 20:1); $[\alpha]_D^{18}$ = +79.2 (*c* 0.500, $CHCl_3$); IR (film): 3416, 2940, 1728, 1622, 1457, 1367, 1257, 1074, 903, 751 cm^{-1}; ^{1}H-NMR (600 MHz, CD_3OD): δ 6.15 (1H, d, *J* = 7.2 Hz), 4.26 (1H, d, *J* = 11.4 Hz), 3.75–3.72 (2H, m), 3.64 (1H, d, *J* = 9.0 Hz), 3.50–3.48 (2H, m), 3.34 (1H, s), 2.93 (3H, s), 2.89–2.88 (1H, m), 2.55 (1H, d, *J* = 4.8 Hz), 2.10–1.93 (5H, m), 1.82 (1H, d, *J* = 7.2 Hz), 1.65 (1H, dd, *J* = 14.4, 5.4 Hz), 1.62–1.55 (2H, m), 1.46 (3H, s), 1.41–1.36 (4H, m); ^{13}C-NMR (150 MHz, CD_3OD): δ 211.7, 172.3, 151.7, 122.5, 113.2, 85.5, 72.9, 70.7, 59.4, 59.1, 56.5, 44.8, 43.7, 42.0, 33.9, 29.7, 28.10, 28.08, 27.2, 26.4, 25.5, 21.2 (Two signals are missing due to overlap); HRMS (ESI) *m/z*: $[M+H]^+$ Calcd for $C_{24}H_{32}NO_6$ 430.2224, Found 430.2226.

Tosylate S27

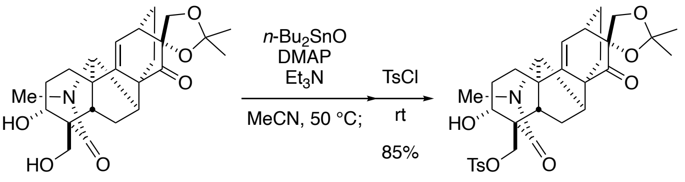

S26 **S27**

To a suspension of 1,3-diol **S26** (75.1 mg, 0.175 mmol), *n*-Bu_2SnO (87.1 mg, 0.350 mmol), and DMAP (42.8 mg, 0.350 mmol) in MeCN (12 mL) was added Et_3N (0.122 mL, 0.875 mmol) at room temperature. After stirring for 12 h at 50 °C, TsCl (50.0 mg, 0.263 mmol) was added in five portions every 20 min at room temperature. After additional stirring for 4 h, the reaction mixture was filtered through a short column of silica gel using 50% acetone in hexane as an eluent. The eluate was concentrated under reduced pressure to give a crude material, which was purified by flash silica gel column chromatography (hexane-acetone = 2:1) to afford tosylate **S27** (86.4 mg, 0.148 mmol, 85%). A white foam; R_f = 0.52 (hexane-acetone = 1:1); $[\alpha]_D^{18}$ = –1.10 (*c*

0.280, $CHCl_3$); IR (film): 2940, 1731, 1628, 1363, 1248, 1171, 1074, 977, 842, 671 cm^{-1}; ^{1}H-NMR (600 MHz, $CDCl_3$): δ 7.77 (2H, d, J = 8.4 Hz), 7.34 (2H, d, J = 8.4 Hz), 6.05 (1H, d, J = 6.6 Hz), 4.74 (1H, d, J = 10.8 Hz), 3.88 (1H, d, J = 10.8 Hz), 3.75–3.68 (2H, m), 3.73 (1H, ddd, J = 12.0, 12.0, 4.8 Hz), 3.24 (1H, s), 2.91 (3H, s), 2.89 (1H, ddd, J = 6.6, 2.4, 2.4 Hz), 2.50–2.49 (1H, m), 2.49 (3H, s), 2.42 (1H, d, J = 10.8 Hz), 2.18–2.07 (2H, m), 1.98–1.95 (2H, m), 1.83–1.80 (1H, m), 1.75 (1H, ddd, J = 7.2, 1.8, 1.8 Hz), 1.58–1.55 (1H, m), 1.55 (3H, s), 1.46 (3H, s), 1.45–1.32 (4H, m); ^{13}C-NMR (150 MHz, $CDCl_3$): δ 209.2, 168.4, 149.5, 145.4, 132.1, 130.0 (2C), 128.1 (2C), 121.7, 112.2, 83.8, 72.0, 71.4, 69.1, 66.5, 57.5, 54.5, 48.1, 43.2, 42.5, 40.5, 33.8, 28.9, 27.2, 27.0, 26.9, 25.9, 24.2, 21.7, 20.2; HRMS (ESI) *m/z*: $[M+H]^+$ Calcd for $C_{31}H_{38}NO_8S$ 584.2313, Found 584.2294.

Alkene 59

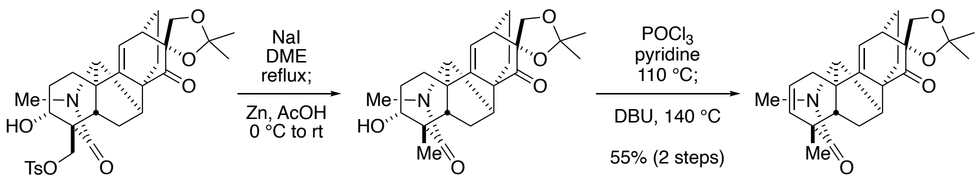

S27 **S28** **59**

To a solution of tosylate **S27** (43.2 mg, 74.0 μmol) in DME (2.4 mL) was added NaI (333 mg, 2.22 mmol) at room temperature. After stirring for 36 h at 110 °C, AcOH (1.2 mL) and Zn (58.1 mg, 0.888 mmol) were added at 0 °C and the mixture was stirred for 1.5 h at room temperature. The reaction was quenched with saturated aqueous $NaHCO_3$, and the mixture was extracted with EtOAc three times. The combined organic extracts were washed with brine, dried over anhydrous sodium sulfate, and filtered. The filtrate was concentrated under reduced pressure to remove organic solvents to give a crude **S28**, which was used for the next reaction without further purification.

To the solution of the crude **S28** in pyridine (2.5 mL) was added $POCl_3$ (35.0 μL, 0.370 mmol) at room temperature. After stirring for 11 h at 110 °C, DBU (245 μL) was added at room temperature and the mixture was stirred for 22 h at 140 °C. The solvent removed under reduced pressure, and the residue was filtered through a short column of silica gel using 50% acetone in hexane as an eluent. The eluate was concentrated under reduced pressure to give a crude material, which was purified by flash silica gel column chromatography (hexane-acetone = 2:1) to afford alkene **59** (16.0 mg, 40.5 μmol, 55%, 2 steps). A white foam; R_f = 0.46 (hexane-acetone = 2:1); $[\alpha]_D^{18}$ = –1.90 (*c* 0.280, $CHCl_3$); IR (film): 2930, 1728, 1657, 1641, 1461, 1367, 1257, 1216, 1058, 893, cm^{-1}; ^{1}H-NMR (600 MHz, $CDCl_3$): δ 6.10 (1H, d, J = 6.6 Hz), 5.84 (1H, ddd, J = 9.6, 3.6, 3.6 Hz), 5.69 (1H, d, J = 9.6 Hz), 3.68 (2H, s), 3.26 (1H, s), 2.91 (1H, ddd, J = 6.6, 3.0, 3.0 Hz), 2.89 (3H, s), 2.65 (1H, ddd, J = 19.8, 3.6, 1.8 Hz), 2.53 (1H, d, J = 5.4 Hz), 2.38 (1H, ddd, J = 19.8, 1.8, 1.8 Hz), 2.18–2.13 (1H, m), 2.03 (1H, dd, J = 13.8, 7.8 Hz), 1.87 (1H, ddd, J = 13.8, 10.2, 5.4 Hz), 1.62–1.60 (1H, m), 1.53 (3H, s), 1.50–1.47 (2H, m), 1.45 (3H, s), 1.40 (1H, dddd, J = 12.0, 12.0, 3.6, 3.6 Hz), 1.24 (3H, s); ^{13}C-NMR (150 MHz, $CDCl_3$): δ 209.9, 172.8, 150.0, 133.7, 126.9, 121.1, 112.2, 84.0, 73.7, 72.1, 58.5, 48.3, 46.2, 43.7, 43.2, 41.0, 34.5, 27.5, 27.2, 27.1, 26.6, 25.9, 20.8, 20.2; HRMS (EI) *m/z*: $[M]^+$ Calcd for $C_{24}H_{29}NO_4$ 395.2091, Found 395.2081.

Triol 60

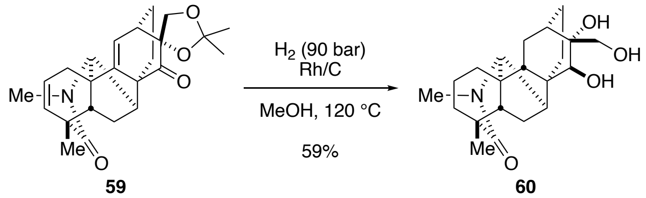

To a suspension of alkene **59** (13.9 mg, 35.1 μmol) and 5% Rh/C (55% suspension in H_2O) (132 mg, 35.1 μmol) in MeOH (1.8 mL) was pressurized with hydrogen gas (90 bar) at room temperature. After stirring for 10 h at 120 °C, hydrogen pressure was released, and the reaction mixture was filtered through a pad of Celite and the filter cake was washed with MeOH. The combined filtrate and washings were concentrated under reduced pressure to give a crude material, which was purified by preparative TLC (CH_3CN-MeOH = 20:1) to afford triol **60** (7.5 mg, 20.7 μmol, 59%). A white foam; R_f = 0.30 (CH_2Cl_2-MeOH = 10:1); $[\alpha]_D^{14}$ = –38.2 (*c* 0.220, $CHCl_3$); IR (film): 3387, 2926, 1622, 1457, 1399, 1370, 1325, 1235, 1067, 751 cm^{-1}; ^{1}H-NMR (600 MHz, $CDCl_3$): δ 4.17 (1H, d, *J* = 11.4 Hz), 4.03 (1H, s), 3.67 (1H, s), 3.49 (1H, d, *J* = 11.4 Hz), 2.98–2.92 (4H, m), 2.86 (1H, dd, *J* = 13.8, 8.4 Hz), 2.06 (1H, d, *J* = 4.8 Hz), 2.03–1.96 (2H, m), 1.92–1.80 (3H, m), 1.64–1.59 (2H, m), 1.51–1.42 (2H, m), 1.38–1.27 (4H, m), 1.24–1.16 (2H, m), 1.07 (3H, s); ^{13}C-NMR (150 MHz, $CDCl_3$): δ 174.7, 86.4, 78.9, 73.2, 67.8, 51.9, 47.4, 45.8, 44.8, 42.5, 40.5, 37.3, 36.5, 34.4, 26.6, 26.3, 26.1, 23.0, 22.1, 21.4, 20.4; HRMS (ESI) *m/z*: $[M+H]^+$ Calcd for $C_{21}H_{32}NO_4$ 362.2326, Found 362.2324.

(–)-Dictizine (9)

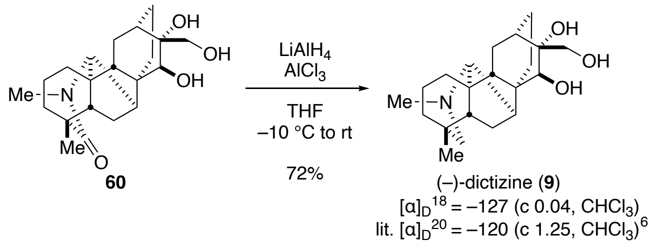

To a solution of triol **60** (3.2 mg, 8.97 μmol) and $AlCl_3$ (12.0 mg, 89.7 μmol) in THF (900 μL) was added $LiAlH_4$ (10.2 mg, 0.269 mmol) at –10 °C. After stirring for 6 h at room temperature, the reaction was quenched by slow addition of H_2O (3.4 μL), 15% aqueous NaOH (3.4 μL), and H_2O (10.2 μL), sequentially, at –10 °C. The resulting suspension was diluted with 15% aqueous NaOH at room temperature and extracted with 33% EtOH in $CHCl_3$ five times. The combined organic extracts were washed with brine, dried over anhydrous sodium sulfate, and filtered. The filtrate was concentrated under reduced pressure to remove organic solvents to give a crude material, which was purified by preparative TLC (CH_2Cl_2-MeOH- NH_4OH = 88:10:2) to afford (–)-dictizine (**9**) (2.2 mg, 6.33 μmol, 72%). A white foam; R_f = 0.30 (CH_2Cl_2-MeOH-NH_4OH = 88:10:2); $[\alpha]_D^{18}$ = –127 (*c* 0.0400, $CHCl_3$); IR (film): 3391, 2921, 2866, 1557, 1538, 1502, 1457, 1061, 1010, 770 cm^{-1}; ^{1}H-NMR (600 MHz, $CDCl_3$): δ 4.22 (1H, d, *J* = 11.4 Hz), 4.05 (1H, s), 3.46 (1H, d, *J* = 11.4 Hz), 3.33 (1H, br s), 2.50–2.43 (2H, m), 2.27–2.20 (5H, m), 2.11 (1H, d, *J* = 5.4 Hz), 1.99–1.93 (2H, m), 1.89–1.82 (2H, m), 1.58–1.51 (3H, m), 1.46–1.44 (1H, m), 1.39–1.30 (2H, m), 1.26–1.14 (5H, m), 0.71 (3H, s); ^{13}C-NMR (150 MHz, $CDCl_3$): δ 88.2, 79.2, 73.4, 68.0, 59.6, 52.8, 45.4, 44.0, 42.4, 42.3, 40.5, 40.1, 36.8, 34.2, 27.4, 26.5, 23.3, 21.9,

20.7 (Two signals are missing due to overlap); HRMS (ESI) *m/z*: $[M+H]^+$ Calcd for $C_{21}H_{34}NO_3$ 348.2533, Found 348.2525. The spectral data of **9** were identical with those reported in the literature.[6,7]

Table S9. ^{1}H-NMR comparison table for (–)-dictizine (9)

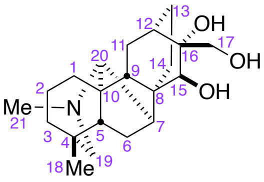

(–)-dictizine (**9**)

	Synthetic (–)-dictizine (CDCl$_3$, 600 MHz)	Natural (–)-dictizine (CDCl$_3$, 500 MHz)[7]
**H-17A**	4.22 (1H, d, *J* = 11.4 Hz)	4.21 (1H, d, *J* = 11.4 Hz)
**H-15**	4.05 (1H, s)	4.05 (1H, s)
**H-17B**	3.46 (1H, d, *J* = 11.4 Hz)	3.47 (1H, d, *J* = 11.4 Hz)
**H-20**	3.33 (1H, br s)	3.39 (1H, s)
**H-6A**	2.50–2.43 (2H, m)	2.55 (2H, m)
**H-19A**		
**H-21**	2.27–2.20 (5H, m)	2.32 (3H, s)
**H-19B**		2.30 (1H, br d, *J* = 12.1 Hz)
**H-2A**		2.24 (1H, m)
**H-7**	2.11 (1H, d, *J* = 5.4 Hz)	2.13 (1H, br d, *J* = 6.8 Hz)
**H-13A**	1.99–1.93 (2H, m)	1.95 (2H, m)
**H-14A**		
**H-1A**	1.89–1.82 (2H, m)	1.91 (1H, m)
**H-9**		1.86 (1H, m)
**H-12**	1.58–1.51 (3H, m)	1.62 (1H, m)
**H-3A**		1.60 (1H, m)
**H-11A**		1.53 (1H, m)
**H-2B**	1.46–1.44 (1H, m)	1.43 (1H, m)
**H-1B**	1.39–1.30 (2H, m)	1.40 (1H, m)
**H-13B**		1.32 (1H, m)
**H-6B**	1.26–1.14 (5H, m)	1.25 (1H, m)
**H-3B**		1.23 (1H, m)
**H-11B**		1.20 (1H, m)
**H-14B**		1.17 (1H, m)
**H-5**		1.11 (1H, m)
**H-18**	0.70 (3H, s)	0.72 (3H, s)

Table S10. ^{13}C-NMR comparison table for (–)-dictizine (9)

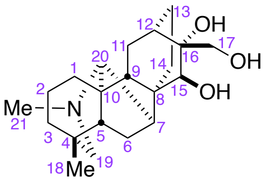

(–)-dictizine (**9**)

	Synthetic (–)-dictizine (150 MHz, $CDCl_3$)	Natural (–)-dictizine (125 MHz, $CDCl_3$)[6]	Deviation (synthetic–natural) Δδ (ppm)
**C-15**	88.2	88.0	0.2
**C-16**	79.2	79.2	0.0
**C-20**	73.4	73.3	0.1
**C-2**	68.0	68.0	0.0
**C-3**	59.6	59.4	0.2
**C-17**	52.8	52.6	0.2
**C-9**	45.4	45.5	–0.1
**C-19**	44.0	44.1	–0.1
**C-21**	42.4	42.6	–0.2
**C-10**	42.3	42.3	0.0
**C-12**	40.5	40.6	–0.1
**C-8**	40.1	40.0	0.1
**C-5**	36.8	36.7	0.1
**C-4**	34.2	34.2	0.0
**C-7**	27.4	27.4	0.0
**C-1**	26.5	26.5	0.0
**C-6**		26.4	0.1
**C-13**	23.3	23.2	0.1
**C-11**		23.2	0.1
**C-18**	21.9	21.9	0.0
**C-14**	20.7	20.6	0.1

(–)-15-Veratroyl-17-acetyl-19-oxodictizine (10)

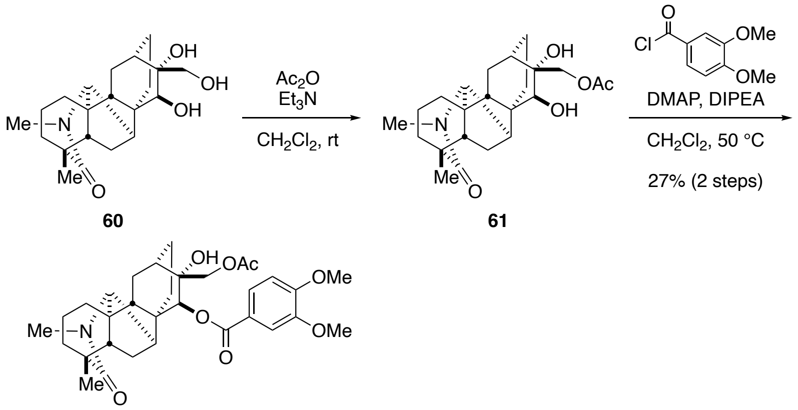

(–)-15-veratroyl-17-acetyl-19-oxodictizine (**10**)
$[\alpha]_D^{19}$ = –113 (c 0.25, $CHCl_3$)
lit. $[\alpha]_D^{20}$ = +49.3 (c 0.15, $CHCl_3$)[8]

To a solution of triol **60** (7.5 mg, 20.7 μmol) and Et_3N (57.8 μL, 0.414 mmol) in CH_2Cl_2 (1.0 mL) was added Ac_2O (19.6 μL, 0.207 mmol) at room temperature. After stirring for 11.5 h at room temperature, the reaction was quenched by saturated aqueous NH_4Cl, and the mixture was extracted with CH_2Cl_2 three times. Combined organic extracts were washed with brine, dried over anhydrous sodium sulfate, and filtered. The filtrate was concentrated under reduced pressure to remove organic solvents to give a residue, which was passed through a short column of silica gel using 5% MeOH in CH_2Cl_2 as an eluent. The eluate was concentrated under reduced pressure to give a crude **61**, which was used for the next reaction without further purification. To the solution of crude **61**, DMAP (38.0 mg, 0.311 mmol) and DIPEA (108 μL, 0.621 mmol) in CH_2Cl_2 (400 μL) was added 3,4-dimethoxybenzoyl chloride (62.4 mg, 0.311 mmol) at room temperature. After stirring for 9.5 h at 50 °C, the reaction was quenched by saturated aqueous $NaHCO_3$, and the mixture was extracted with CH_2Cl_2 three times. Combined organic extracts were washed with 0.5 M aqueous HCl and brine, dried over anhydrous sodium sulfate, and filtered. The filtrate was concentrated under reduced pressure to remove organic solvents to give a residue, which was passed through a short column of N-H silica gel using EtOAc as an eluent. The eluate was concentrated under reduced pressure to give a crude material, which was purified by preparative TLC (hexane-acetone = 2:1) to afford (–)-15-veratroyl-17-acetyl-19-oxodictizine (**10**) (3.2 mg, 5.64 μmol, 27%, 2 steps). A white foam; R_f = 0.43 (hexane-acetone = 1:1; $[\alpha]_D^{18}$ = –113 (*c* 0.250, $CHCl_3$); IR (film): 3410, 2917, 2849, 1728, 1628, 1541, 1515, 1461, 1415, 1377, 1274, 1222, 1132, 1025, 758 cm^{-1}; ^{1}H-NMR (600 MHz, $CDCl_3$): δ 7.65 (1H, dd, *J* = 8.4, 1.8 Hz), 7.52 (1H, d, *J* = 1.8 Hz), 6.94 (1H, d, *J* = 8.4 Hz), 5.22 (1H, s), 4.31 (1H, d, *J* = 12.0 Hz), 4.25 (1H, d, *J* = 12.0 Hz), 3.96 (3H, s), 3.93 (3H, s), 3.74 (1H, s), 3.36 (1H, s), 2.92 (3H, s), 2.61 (1H, dd, *J* = 13.8, 7.2 Hz), 2.26–2.21 (1H, m), 2.13 (1H, d, *J* = 4.8 Hz), 2.05–2.00 (2H, m), 1.94 (1H, dd, *J* = 10.8, 10.8 Hz), 1.88 (1H, br s), 1.84 (1H, br d, *J* = 13.2 Hz), 1.79 (3H, s), 1.74–1.69 (1H, m), 1.62–1.59 (1H, m), 1.53–1.50 (2H, m), 1.48–1.41 (2H, m), 1.39–1.33 (3H, m), 1.21 (1H, ddd, *J* = 13.2, 13.2, 4.2 Hz), 1.05 (3H, s); ^{13}C-NMR (150 MHz, $CDCl_3$): δ 174.2, 170.8, 167.3, 153.6, 148.9, 123.8, 121.8, 111.8, 110.6, 86.1, 78.1, 72.7, 68.5, 56.1, 55.9, 52.2, 47.2, 45.8, 44.9, 42.7, 41.6, 37.2, 35.7, 34.4, 27.2, 26.5, 26.2, 22.8, 22.1, 20.8, 20.6, 20.3; HRMS (ESI) *m/z*: $[M+H]^+$ Calcd for $C_{32}H_{42}NO_8$ 568.2905, Found 568.2920.

The spectral data of **10** were identical with those reported in the literature except for one signal in ^{1}H-NMR and specific optical rotation.[8] The reported δ 0.81 for H13 in ^{1}H-NMR is not consistent with our result and the

reported value is much lower in chemical shift than H13 protons on related denudatine-type natural products (Table S11). One possibility for the discrepancies present in the data reported by Herz et al. might be due to impurities in their sample leading to confusion and misassignment. Furthermore, these impurities could cause the discrepancy in specific optical rotation.

Table S11. ^{1}**H-NMR comparison table for (–)-15-veratroyl-17-acetyl-19-oxodictizine (10)**

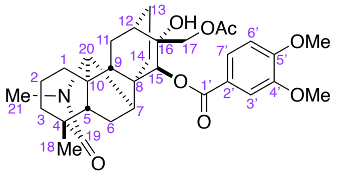

(–)-15-veratroyl-17-acetyl-19-oxodictizine (**10**)

	Synthetic (–)-15-Veratroyl-17-acetyl-19-oxodictizine (**10**) ($CDCl_3$, 600 MHz)	Natural (–)-15-Veratroyl-17-acetyl-19-oxodictizine (**10**) ($CDCl_3$, 500 MHz)[8]
**H-7'**	7.65 (1H, dd, *J* = 8.4, 1.8 Hz)	7.62 (1H, dd, *J* = 8.5, 2.0 Hz)
**H-3'**	7.52 (1H, d, *J* = 1.8 Hz)	7.49 (1H, d, *J* = 2.0 Hz)
**H-6'**	6.94 (1H, d, *J* = 8.4 Hz)	6.91 (1H, d, *J* = 8.5 Hz)
**H-15**	5.22 (1H, s)	5.19 (1H, s)
**H-17A**	4.31 (1H, d, *J* = 12.0 Hz)	4.29 (1H, d, *J* = 11.6 Hz)
**H-17B**	4.25 (1H, d, *J* = 12.0 Hz)	4.22 (1H, d, *J* = 11.6 Hz)
**C-5'-OMe**	3.96 (3H, s)	3.94 (3H, s)
**C-4'-OMe**	3.93 (3H, s)	3.90 (3H, s)
**H-20**	3.74 (1H, s)	3.72 (1H, br s)
**C-16-OH**	3.36 (1H, s)	-
**H-21**	2.92 (3H, s)	2.89 (3H, br s)
**H-6A**	2.61 (1H, dd, *J* = 13.8, 7.2 Hz)	2.58 (1H, dd, *J* = 13.9, 7.9 Hz)
**H-2A**	2.26–2.21 (1H, m)	2.21 (1H, m)
**H-7**	2.13 (1H, d, *J* = 4.8 Hz)	2.10 (1H, br d, *J* = 13.1 Hz)
**H-1A**	2.05–2.00 (2H, m)	2.01 (2H, m)
**H-14A**		
**H-9**	1.94 (1H, dd, *J* = 10.8, 10.8 Hz)	1.93 (1H, t, *J* = 10.5 Hz)
**H-12**	1.88 (1H, br s)	1.86 (1H, br s)
**H-3A**	1.84 (1H, br d, *J* = 13.2 Hz)	1.82 (1H, br d, *J* = 13.1 Hz)
**C-15-OAc**	1.79 (3H, s)	1.76 (3H, s)
**H-11A**	1.74–1.69 (1H, m)	1.67 (1H, m)
**H-13A**	1.62–1.59 (1H, m)	1.56 (1H, m)
**H-14B**	1.53–1.50 (2H, m)	1.50 (1H, m)
**H-13B**		0.81 (1H, m)
**H-1B**	1.48–1.41 (2H, m)	1.42 (2H, m)
**H-2B**		
**H-11B**	1.39–1.33 (3H, m)	1.40 (1H, m)
**H-5**		1.35 (1H, br d, *J* = 8.6 Hz)
**H-6B**		1.32 (1H, m)
**H-3B**	1.21 (1H, ddd, *J* = 13.2, 13.2, 4.2 Hz)	1.18 (1H, m)
**H-18**	1.05 (3H, s)	1.02 (3H, s)

Table S12. ^{13}**C-NMR comparison table for (–)-15-veratroyl-17-acetyl-19-oxodictizine (10)**

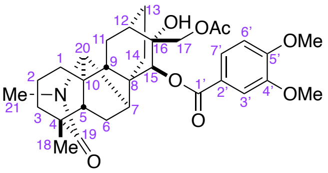

(–)-15-veratroyl-17-acetyl-19-oxodictizine (**10**)

	Synthetic (–)-15-veratroyl-17-acetyl-19-oxodictizine (**10**) (150 MHz, $CDCl_3$)	Natural (–)-15-veratroyl-17-acetyl-19-oxodictizine (**10**) (100 MHz, $CDCl_3$)[8]	Deviation (synthetic–natural) $\Delta\delta$ (ppm)
**C-19**	174.2	174.2	0.0
**C17-OAc**	170.8	170.8	0.0
**C-1’**	167.3	167.3	0.0
**C-5’**	153.6	153.6	0.0
**C-4’**	148.9	148.9	0.0
**C-7’**	123.8	123.8	0.0
**C-2’**	121.8	121.8	0.0
**C-3’**	111.9	111.9	0.0
**C-6’**	110.6	110.6	0.0
**C-15**	86.1	86.0	0.1
**C-16**	78.1	78.1	0.0
**C-20**	72.7	72.7	0.0
**C-17**	68.5	68.4	0.1
**C-4’-OCH_3**	56.1	56.1	0.0
**C-5’-OCH_3**	55.9	55.9	0.0
**C-5**	52.2	52.1	0.1
**C-7**	47.2	47.2	0.0
**C-4**	45.8	45.8	0.0
**C-10**	44.9	44.8	0.1
**C-9**	42.7	42.7	0.0
**C-8**	41.6	41.6	0.0
**C-3**	37.2	37.2	0.0
**C-12**	35.7	35.7	0.0
**C-21**	34.4	34.4	0.0
**C-14**	27.2	27.1	0.1
**C-6**	26.5	26.5	0.0
**C-1**	26.2	26.1	0.1
**C-11**	22.8	22.7	0.1
**C-18**	22.1	22.1	0.0
**C-2**	20.8	20.8	0.0
**C17-OAc**	20.6	20.5	0.1
**C-13**	20.3	20.3	0.0

Computational Experiments

To rationalize the experimentally observed stereo- and diastereomeric selectivity of the intermolecular Diels–Alder reaction (**47→48**), preliminary studies on density functional theory (DFT) calculations were carried out.

All calculations were performed with Spartan '20 (Wavefunction Inc.). Geometry optimizations and frequency analyses were carried out at the B3LYP/6-31G(d) level of theory[9,10] in the gas phase. Stationary points were confirmed by frequency calculations (zero imaginary frequencies for minima and one imaginary frequency for transition states). Transition states were further verified by intrinsic reaction coordinate (IRC) calculations to ensure proper connection between reactants and products. Single-point electronic energies were computed at the M06-2X/6-31G(d) level[11] on the B3LYP-optimized geometries. Gibbs free energies at 298 K and 1 atm were obtained by combining the M06-2X electronic energies with zero-point and thermal corrections from the B3LYP frequency calculations. Activation free energies ($\Delta G^{\ddagger}$) were reported relative to the sum of the individually optimized reactants.

<The reaction Profile of intermolecular Diels–Alder reaction>

The calculated Gibbs free energies (ΔG, 298 K, 1 atm) for the reaction between **S29a**/**S29b** and ethylene are summarized in Figure S5. Among the four possible transition states, **TS-b-α**, corresponding to the α-face approach of ethylene to **S29b**, exhibited the lowest activation free energy ($\Delta G^{\ddagger}$ = 11.7 kcal/mol) and is consistent with the experimentally observed reaction pathway. The remaining transition states, including the α-approach to **S29a** (**TS-a-α**) and the β-face approaches to **S29a**/**S29b** (**TS-a-β** and **TS-b-β**), were higher in energy ($\Delta G^{\ddagger}$ = 14.9, 14.7, and 15.0 kcal/mol, respectively), rationalizing the exclusive selectivity.

The low reactivity of **S29a** would be attributed to steric hindrances of the α- and β-faces caused by the two hydrogen atoms at C5 and C6 and by the methylene carbon of epoxide, respectively. In contrast, the sterically less-hindered α-face of **S29b** could be approached by ethylene while its β-face was shielded by the C5 and C6 hydrogen atoms, and the methylene carbon of epoxide.

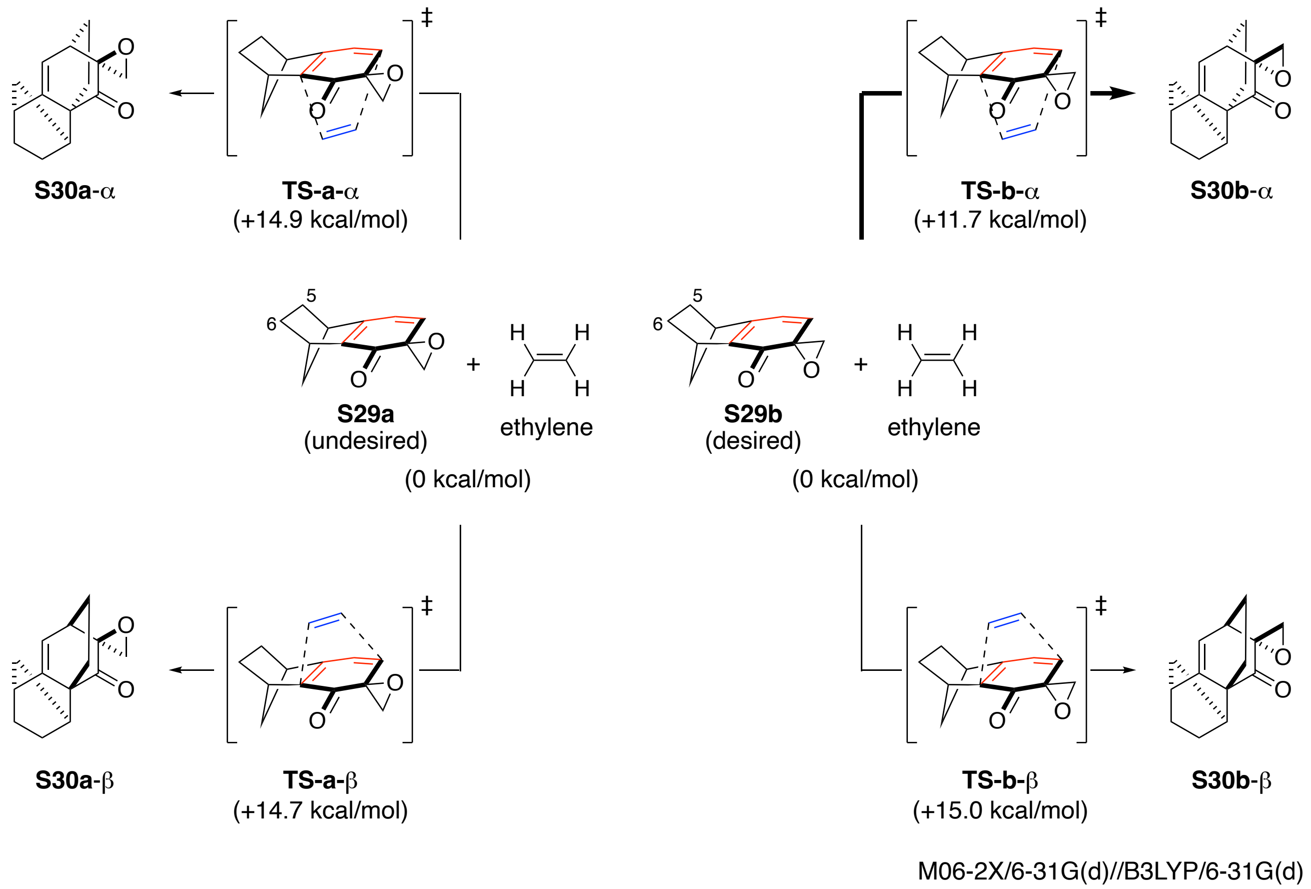

Figure S5. Calculated reaction profile of Diels–Alder reaction using simplified model structures **S29a** and **S29b**

<Optimization of the structure S29a>

The simplified model structure **S29a**, which retains the reactive core of **47a**, was manually constructed and used as the starting point for DFT calculations. Geometry optimization and frequency analysis were performed at the B3LYP/6-31G(d) level of theory in the gas phase (298 K, 1 atm). The optimized structure was confirmed as a true minimum by the absence of imaginary frequencies. Single-point electronic energies were subsequently calculated at the M06-2X/6-31G(d) level on the B3LYP-optimized geometry. Gibbs free energies were obtained by combining the M06-2X electronic energy with zero-point and thermal corrections from the B3LYP frequency calculation.

Structure, Gibbs free energy, and Cartesian coordinate of **S29a**:

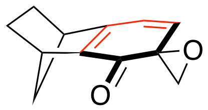

S29a

Gibbs free energy (298 K)
–385,543.12 kcal/mol

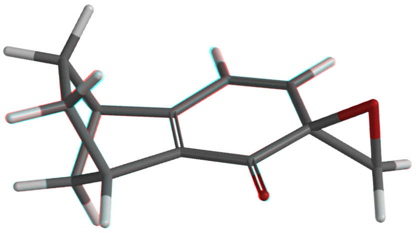

	X	Y	Z
H	0.9464	-1.4139	-1.9757
C	1.7503	-0.9870	-1.3698
C	1.6945	1.1093	-0.0976
C	0.1092	-0.5030	0.4032
C	0.2414	0.8485	0.2504
C	1.4657	-1.1336	0.1633
C	1.8993	0.5557	-1.5532
H	1.5912	-2.1430	0.5567
H	1.1742	0.9753	-2.2569
H	2.6772	-1.5082	-1.6334
H	2.0472	2.1334	0.0484
H	2.8998	0.8218	-1.9112
C	2.3785	-0.0138	0.7264
H	3.4379	-0.1447	0.4752
H	2.2727	0.1229	1.8073
C	-1.1704	-1.1306	0.6753
C	-0.8640	1.7778	0.3621
H	-0.6851	2.8442	0.2526
C	-2.3412	-0.1490	0.7730
C	-2.0949	1.3036	0.6488
H	-2.9468	1.9613	0.7999
O	-1.3505	-2.3381	0.7970
C	-3.6691	-0.7017	0.3644
H	-3.6779	-1.7346	0.0178
H	-4.4198	-0.0289	-0.0527
O	-3.3661	-0.5236	1.7286

<Optimization of the structure S29b>

The simplified model structure **S29b**, which retains the reactive core of **47b**, was manually constructed and used as the starting point for DFT calculations. Geometry optimization and frequency analysis were performed at the B3LYP/6-31G(d) level of theory in the gas phase (298 K, 1 atm). The optimized structure was confirmed as a true minimum by the absence of imaginary frequencies. Single-point electronic energies were subsequently calculated at the M06-2X/6-31G(d) level on the B3LYP-optimized geometry. Gibbs free energies were obtained by combining the M06-2X electronic energy with zero-point and thermal corrections from the B3LYP frequency calculation.

Structure, Gibbs free energy, and Cartesian coordinate of **S29b**:

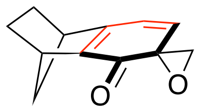

S29b

Gibbs free energy (298 K)
–385,542.61 kcal/mol

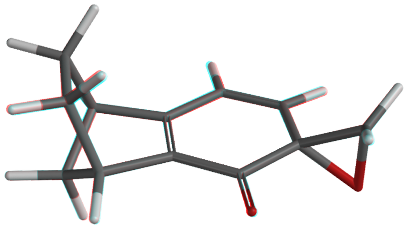

	X	Y	Z
H	-1.6204	1.9751	-0.9221
C	-2.1748	1.1228	-0.5202
C	-1.4930	-1.2346	-0.5362
C	-0.0892	0.2859	0.5017
C	-0.0539	-0.8107	-0.3133
C	-1.5407	0.5920	0.8097
C	-2.1313	-0.1294	-1.4516
H	-1.7114	1.2250	1.6815
H	-1.5476	0.0354	-2.3624
H	-3.2057	1.4497	-0.3444
H	-1.6444	-2.2567	-0.8926
H	-3.1372	-0.4387	-1.7556
C	-2.1080	-0.8507	0.8347
H	-3.2039	-0.8893	0.8394
H	-1.7174	-1.4514	1.6623
C	1.1023	1.0549	0.8156
C	1.1620	-1.3193	-0.9143
H	1.1159	-2.1742	-1.5838
C	2.3989	0.4405	0.2837
C	2.3368	-0.7157	-0.6355
H	3.2751	-1.0467	-1.0728
O	1.1185	2.0883	1.4759
O	3.4486	1.3974	-0.0055
C	3.5962	0.6225	1.1614
H	3.4347	1.1541	2.0991
H	4.3897	-0.1261	1.1455

<Optimization of the structure ethylene>

Ethylene was manually constructed and used as the starting point for DFT calculations. Geometry optimization and frequency analysis were performed at the B3LYP/6-31G(d) level of theory in the gas phase (298 K, 1 atm). The optimized structure was confirmed as a true minimum by the absence of imaginary frequencies. Single-point electronic energies were subsequently calculated at the M06-2X/6-31G(d) level on the B3LYP-optimized geometry. Gibbs free energies were obtained by combining the M06-2X electronic energy with zero-point and thermal corrections from the B3LYP frequency calculation.

Structure, Gibbs free energy, and Cartesian coordinate of ethylene

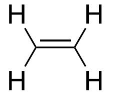

ethylene
Gibbs free energy (298 K)
−49249.49 kcal/mol

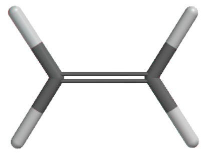

	X	Y	Z
C	0	0	0.6654
H	-0.9227	0	1.2411
H	0.9227	0	1.2411
C	0	0	-0.6654
H	0.9227	0	-1.2411
H	-0.9227	0	-1.2411
C	0	0	0.6654

<Optimization of TS-a-α>

The preliminary transition state (TS) structure for the α-face approach of ethylene to **S29a** (**TS-a-α**) was obtained from a constrained distance scan between the reacting centers of the B3LYP/6-31G(d)-optimized structures of **S29a** and ethylene. The structure corresponding to the highest energy point in the scan was used as the initial guess for full TS optimization without constraints at the B3LYP/6-31G(d) level of theory in the gas phase (298 K, 1 atm). Frequency analysis confirmed the optimized structure as a true transition state by the presence of a single imaginary frequency. The connectivity of the TS to the reactants and the product was further validated by intrinsic reaction coordinate (IRC) calculations. Single-point electronic energy at the M06-2X/6-31G(d) level was calculated on the B3LYP-optimized TS geometry, and Gibbs free energy was obtained by combining these electronic energies with zero-point and thermal corrections from the B3LYP frequency calculation.

Structure, Gibbs free energy, and Cartesian coordinate of **TS-a-α**:

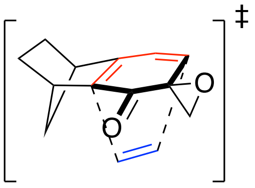

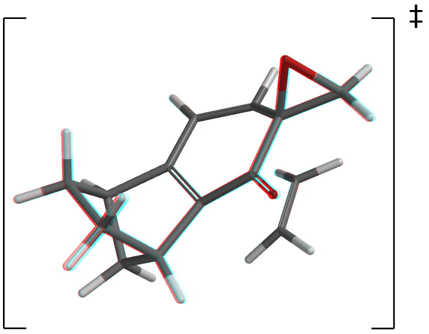

TS-a-α
Gibbs free energy (298 K)
–434777.70 kcal/mol

	X	Y	Z
H	-1.7374	2.3380	-0.7759
C	-2.3645	1.4626	-0.5878
C	-1.8279	-0.8729	-1.0986
C	-0.3781	0.3183	0.3062
C	-0.3741	-0.6024	-0.7602
C	-1.8309	0.6195	0.6160
C	-2.3660	0.4375	-1.7658
H	-2.0223	1.0624	1.5944
H	-1.7403	0.7521	-2.6058
H	-3.3770	1.8215	-0.3723
H	-2.0242	-1.7843	-1.6689
H	-3.3790	0.2730	-2.1493
C	-2.4725	-0.7538	0.3058
H	-3.5672	-0.7180	0.2688
H	-2.1652	-1.5482	0.9935
C	0.7483	1.2295	0.4928
C	0.8164	-1.1155	-1.2601
H	0.8252	-1.9342	-1.9754
C	2.0473	0.7058	-0.1083
C	2.0018	-0.6944	-0.6380
H	2.9519	-1.0829	-0.9988
O	0.6818	2.3244	1.0389
O	2.7234	1.6614	-0.9655
C	3.3063	1.3526	0.2960
H	3.2552	2.1499	1.0360
H	4.2345	0.7791	0.2590
C	0.7674	-1.0107	1.9562
H	-0.1595	-1.5617	2.0598
H	0.8962	-0.1795	2.6414

C	1.8639	-1.5773	1.3123
H	2.8614	-1.2483	1.5890
H	1.8053	-2.6036	0.9647

<Optimization of TS-a-β>

The preliminary transition state (TS) structure for the β-face approach of ethylene to **S29a** (**TS-a-β**) was obtained from a constrained distance scan between the reacting centers of the B3LYP/6-31G(d)-optimized structures of **S29a** and ethylene. The structure corresponding to the highest energy point in the scan was used as the initial guess for full TS optimization without constraints at the B3LYP/6-31G(d) level of theory in the gas phase (298 K, 1 atm). Frequency analysis confirmed the optimized structure as a true transition state by the presence of a single imaginary frequency. The connectivity of the TS to the reactants and the product was further validated by intrinsic reaction coordinate (IRC) calculations. Single-point electronic energy at the M06-2X/6-31G(d) level was calculated on the B3LYP-optimized TS geometry, and Gibbs free energy was obtained by combining these electronic energies with zero-point and thermal corrections from the B3LYP frequency calculation.

Structure, Gibbs free energy, and Cartesian coordinate of **TS-a-β**:

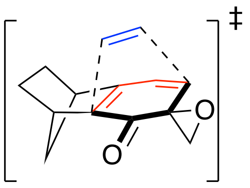

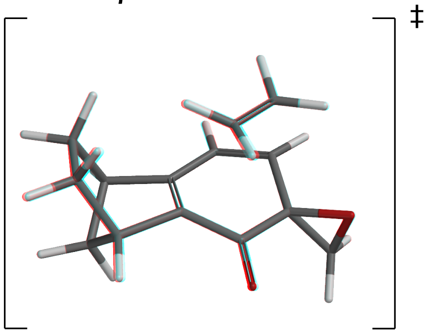

TS-a-β
Gibbs free energy (298 K)
−434777.92 kcal/mol

	X	Y	Z
C	0.5475	1.5216	-1.4569
H	-0.4333	1.3675	-1.8891
H	0.6858	2.4470	-0.9079
C	1.6437	0.7828	-1.9038
H	2.6331	1.2219	-1.8017
H	1.5196	0.1320	-2.7643
H	-2.3158	1.4960	-0.9415
C	-2.6308	0.6874	-0.2775
C	-1.6739	-1.5375	0.0895
C	-0.2938	0.2983	0.5524
C	-0.2525	-1.0059	0.0158
C	-1.7291	0.5539	0.9845
C	-2.5359	-0.7272	-0.9342

H	-1.8406	1.3531	1.7186
H	-2.0722	-0.7063	-1.9254
H	-3.6589	0.9064	0.0314
H	-1.7716	-2.6223	-0.0011
H	-3.5251	-1.1846	-1.0446
C	-2.1195	-0.8873	1.4285
H	-3.1912	-1.0004	1.6345
H	-1.5438	-1.2418	2.2891
C	0.8988	0.9196	1.1147
C	0.9119	-1.5264	-0.5288
H	0.9169	-2.4612	-1.0840
C	2.1853	0.2539	0.6397
C	2.0490	-0.6920	-0.5123
H	2.9918	-1.0797	-0.8950
O	0.9072	1.8294	1.9375
O	3.3451	1.0968	0.6305
C	3.2756	0.0898	1.6335
H	3.1026	0.4555	2.6449
H	3.9741	-0.7404	1.5232

<Optimization of TS-b-α>

The preliminary transition state (TS) structure for the α-face approach of ethylene to **S29b** (**TS-b-α**) was obtained from a constrained distance scan between the reacting centers of the B3LYP/6-31G(d)-optimized structures of **S29b** and ethylene. The structure corresponding to the highest energy point in the scan was used as the initial guess for full TS optimization without constraints at the B3LYP/6-31G(d) level of theory in the gas phase (298 K, 1 atm). Frequency analysis confirmed the optimized structure as a true transition state by the presence of a single imaginary frequency. The connectivity of the TS to the reactants and the product was further validated by intrinsic reaction coordinate (IRC) calculations. Single-point electronic energy at the M06-2X/6-31G(d) level was calculated on the B3LYP-optimized TS geometry, and Gibbs free energy was obtained by combining these electronic energies with zero-point and thermal corrections from the B3LYP frequency calculation.

Structure, Gibbs free energy, and Cartesian coordinate of **TS-b-α**:

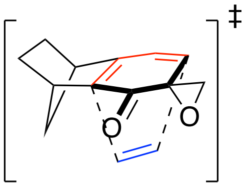

TS-b-α
Gibbs free energy (298 K)
–434780.37 kcal/mol

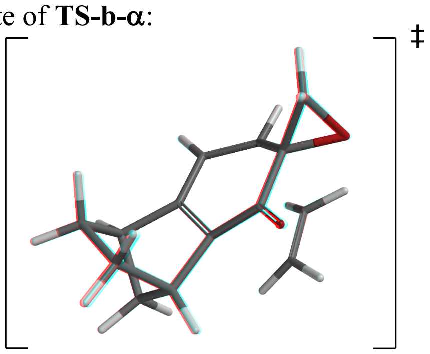

	X	Y	Z
C	-0.2390	-0.2916	2.4110
H	0.7990	-0.0272	2.5760
H	-0.4773	-1.3456	2.5089
C	-1.2438	0.6707	2.4798
H	-2.2498	0.3534	2.7395
H	-0.9871	1.6835	2.7741
H	0.9873	-1.6386	-2.2602
C	1.8285	-1.1255	-1.7863
C	1.8083	1.1048	-0.7693
C	0.3161	-0.5053	0.0536
C	0.3920	0.8624	-0.2796
C	1.6825	-1.1004	-0.2290
C	1.9187	0.3904	-2.1577
H	1.8895	-2.0557	0.2557
H	1.1287	0.7101	-2.8437
H	2.7427	-1.6587	-2.0698
H	2.1414	2.1458	-0.7680
H	2.8805	0.6299	-2.6248
C	2.5892	0.1050	0.1224
H	3.6288	-0.0291	-0.1981
H	2.5725	0.3687	1.1847
C	-0.9609	-1.2093	0.0062
C	-0.6873	1.7122	-0.0731
H	-0.5868	2.7907	-0.1669
C	-2.1553	-0.2729	0.1455
C	-1.8373	1.1434	0.5026
H	-2.7012	1.7737	0.7039
O	-1.0989	-2.4141	-0.1766
C	-3.3752	-0.6106	-0.6309

H	-3.3510	-1.5048	-1.2525
H	-4.0422	0.1919	-0.9476
O	-3.3125	-0.8470	0.7703

<Optimization of TS-b-β>

The preliminary transition state (TS) structure for the β-face approach of ethylene to **S29b** (**TS-b-β**) was obtained from a constrained distance scan between the reacting centers of the B3LYP/6-31G(d)-optimized structures of **S29b** and ethylene. The structure corresponding to the highest energy point in the scan was used as the initial guess for full TS optimization without constraints at the B3LYP/6-31G(d) level of theory in the gas phase (298 K, 1 atm). Frequency analysis confirmed the optimized structure as a true transition state by the presence of a single imaginary frequency. The connectivity of the TS to the reactants and the product was further validated by intrinsic reaction coordinate (IRC) calculations. Single-point electronic energy at the M06-2X/6-31G(d) level was calculated on the B3LYP-optimized TS geometry, and Gibbs free energy was obtained by combining these electronic energies with zero-point and thermal corrections from the B3LYP frequency calculation.

Structure, Gibbs free energy, and Cartesian coordinate of **TS-b-β**:

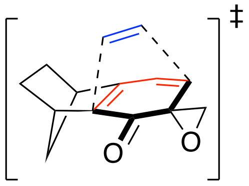

TS-b-β
Gibbs free energy (298 K)
−434777.08 kcal/mol

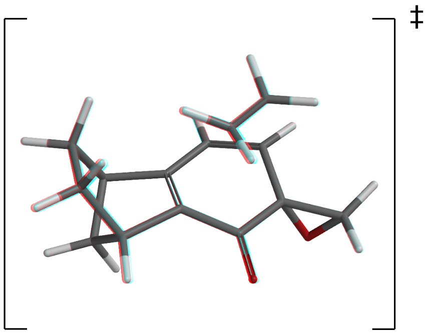

	X	Y	Z
H	1.7380	-1.0701	-2.0149
C	2.3427	-0.8189	-1.1402
C	2.0650	0.9722	0.5090
C	0.3147	-0.5702	0.3095
C	0.5564	0.8007	0.5258
C	1.6713	-1.2470	0.1992
C	2.5566	0.7179	-0.9548
H	1.6530	-2.3219	0.3839
H	1.9995	1.3192	-1.6805
H	3.3022	-1.3360	-1.2517
H	2.4340	1.9004	0.9523
H	3.6138	0.9887	-1.0498
C	2.4809	-0.3607	1.1903

H	3.5609	-0.5502	1.1540
H	2.1302	-0.4418	2.2237
C	-0.9242	-1.1954	0.7554
C	-0.4849	1.7128	0.6309
H	-0.3048	2.7847	0.6568
C	-2.0360	-0.1854	1.0306
C	-1.7940	1.2059	0.5195
H	-2.6273	1.8932	0.6564
O	-1.0747	-2.3941	0.9679
C	-3.3897	-0.6955	1.2966
H	-3.5485	-1.7715	1.2478
H	-4.2512	-0.0635	1.0730
O	-2.5691	-0.2540	2.3769
C	-1.9784	0.6931	-1.4860
H	-3.0332	0.4441	-1.4000
H	-1.7852	1.6818	-1.8907
C	-1.0787	-0.3360	-1.7647
H	-0.1462	-0.1285	-2.2740
H	-1.3932	-1.3737	-1.7522

References

1) L. C. Sequeira, P. R. R. Costa, A. Neves, P. Esteves, *Tetrahedron: Asymmetry* **1994**, *8*, 1433–1434.
2) (a) A. H. Meriçli, S. Süzgeç, L. Biriş, F. Meriçli, H. Özçelik, J. Zapp, H. Becker, *Pharmazie* **2006**, *61*, 483–485. (b) S. Suzgec, L. Bitis, U. Sozer, H. Ozcelik, J. Zapp, A. K. Kiemer, F. Mericli, A. H. Meriçli, *Chem. Nat. Compd.* **2009**, *45*, 287–289.
3) (a) U. Kolak, M. Öztürk, F. Özgökçe, A. Ulubelen, *Phytochemistry* **2006**, *67*, 2170–2175. (b) Wada, K.; Kawahara, N. *Helv. Chim. Acta* **2009**, 92, 629–637.
4) a) K. G. M. Kou, B. X. Li, J. C. Lee, G. M. Gallego, T. P. Lebold, A. G. Dipasquale, R. Sarpong, *J. Am. Chem. Soc.* **2016**, *138*, 10830–10833. b) K. G. M. Kou, S. Kulyk, C. J. Marth, J. C. Lee, N. A. Doering, B. X. Li, G. M. Gallego, T. P. Lebold, R. Sarpong, *J. Am. Chem. Soc.* **2017**, *139*, 13882–13896.
5) M. H. Benn, F. Okanga, J. F. Richardson, *Heterocycles* **1987**, *26*, 2331–2334.
6) B. T. Salimov, M. S. Yunusov, Y. V. Rashkes, S. Y. Yunusov. *Khim. Prir. Soedin.* **1979**, 812–817.
7) P. M. Shrestha, A. Katz, *J. Nat. Prod.* **2004**, *67*, 1574-1576.
8) J. G. Díaz, J. G. Ruiza, W. Herz, *Phytochemistry* **2005**, *66*, 837–846.
9) A. D. Becke, *J. Chem. Phys.* **1993**, *98*, 5648–5652.
10) C. Lee, W. Yang, R. G. Parr, *Phys. Rev. B* **1988**, *37*, 785–789.
11) Y. Zhao, D. G. Truhlar, *Theor. Chem. Acc.* **2008**, *120*, 215–241.

^{1}H- and ^{13}C-NMR Spectra

^{1}H-NMR (400 MHz, $CDCl_3$)

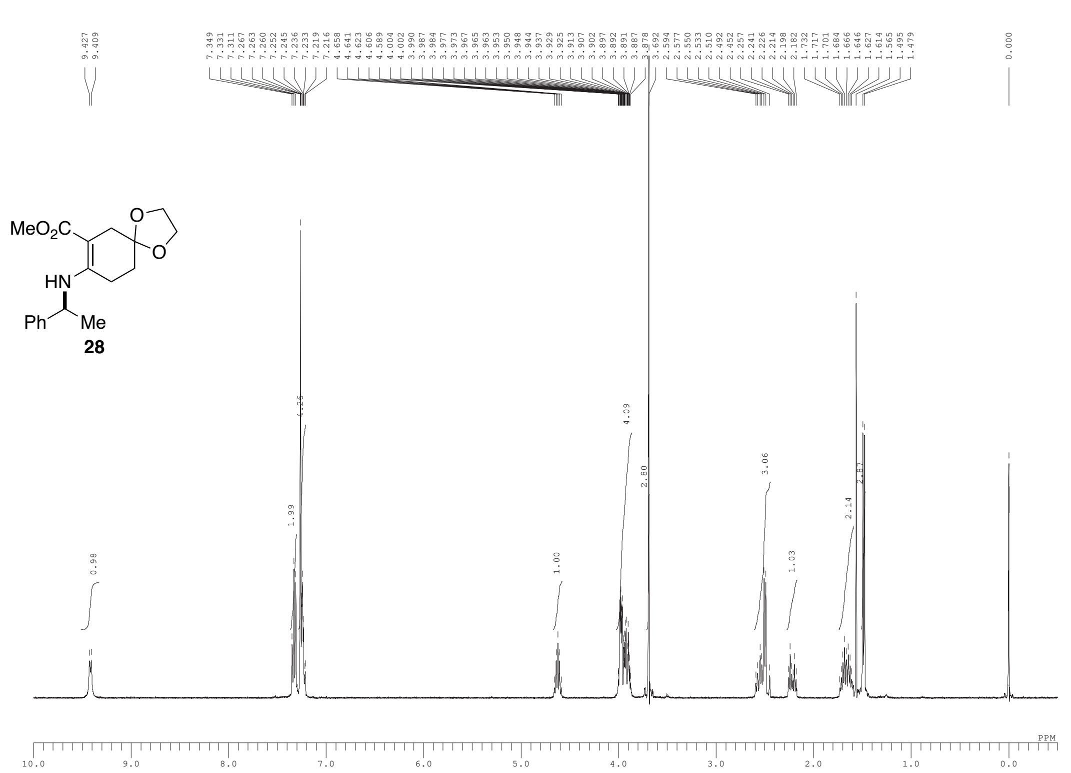

^{13}C-NMR (100 MHz, $CDCl_3$)

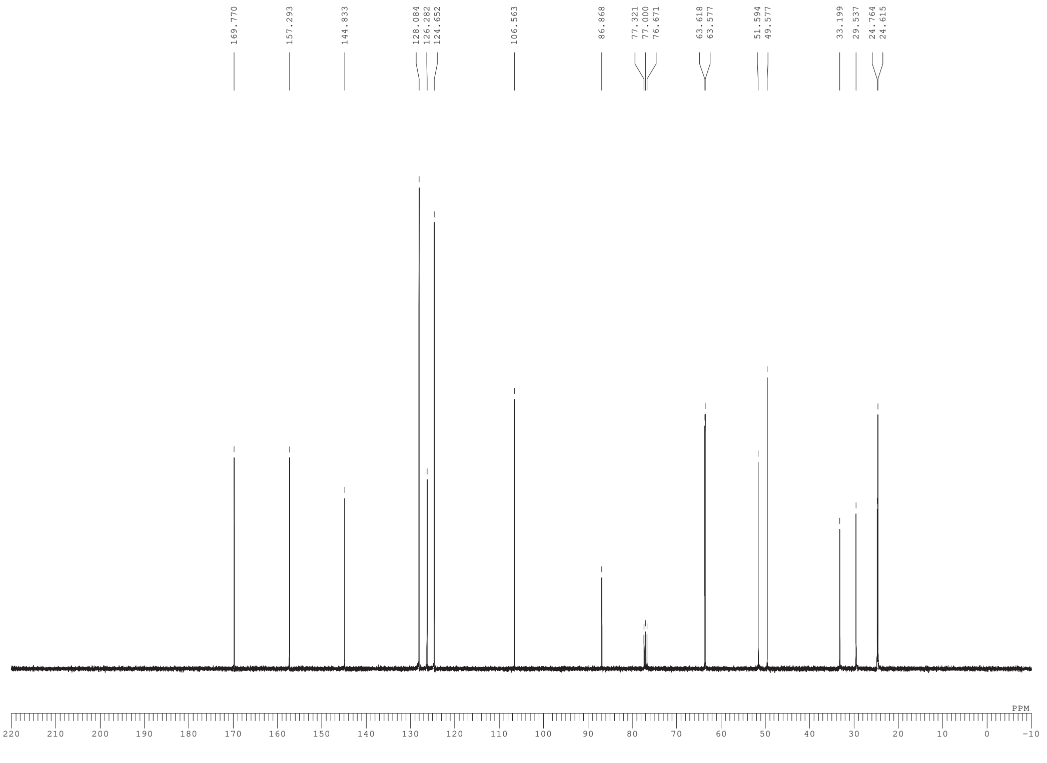

^{1}H-NMR (400 MHz, $CDCl_3$)

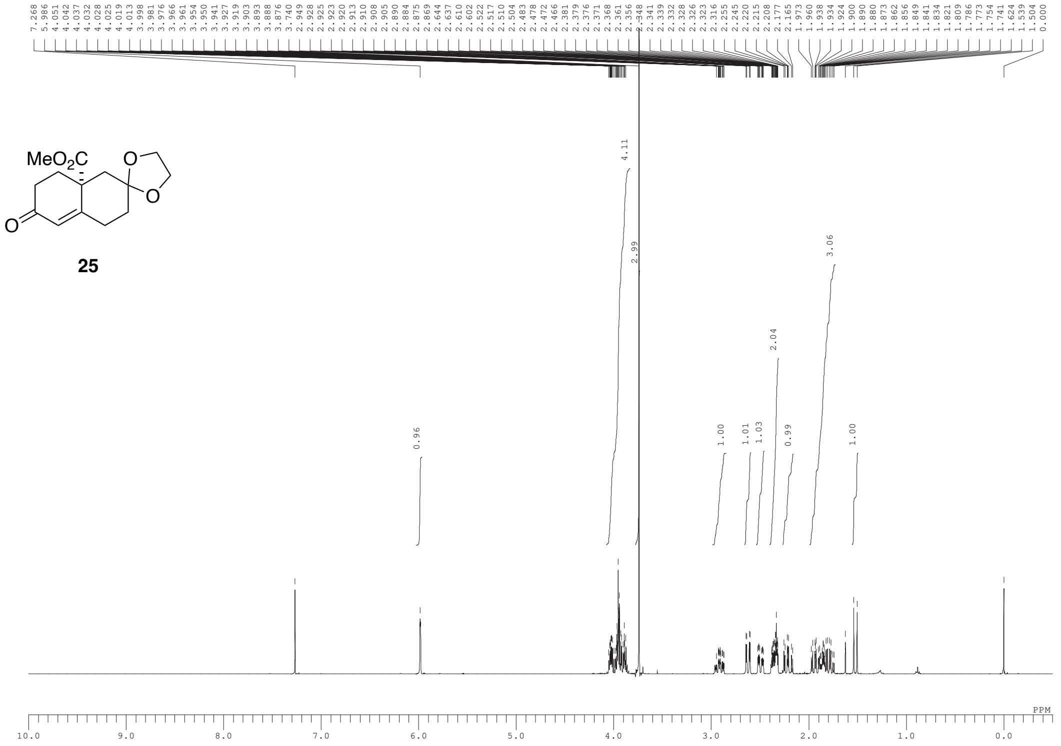

25

^{13}C-NMR (150 MHz, $CDCl_3$)

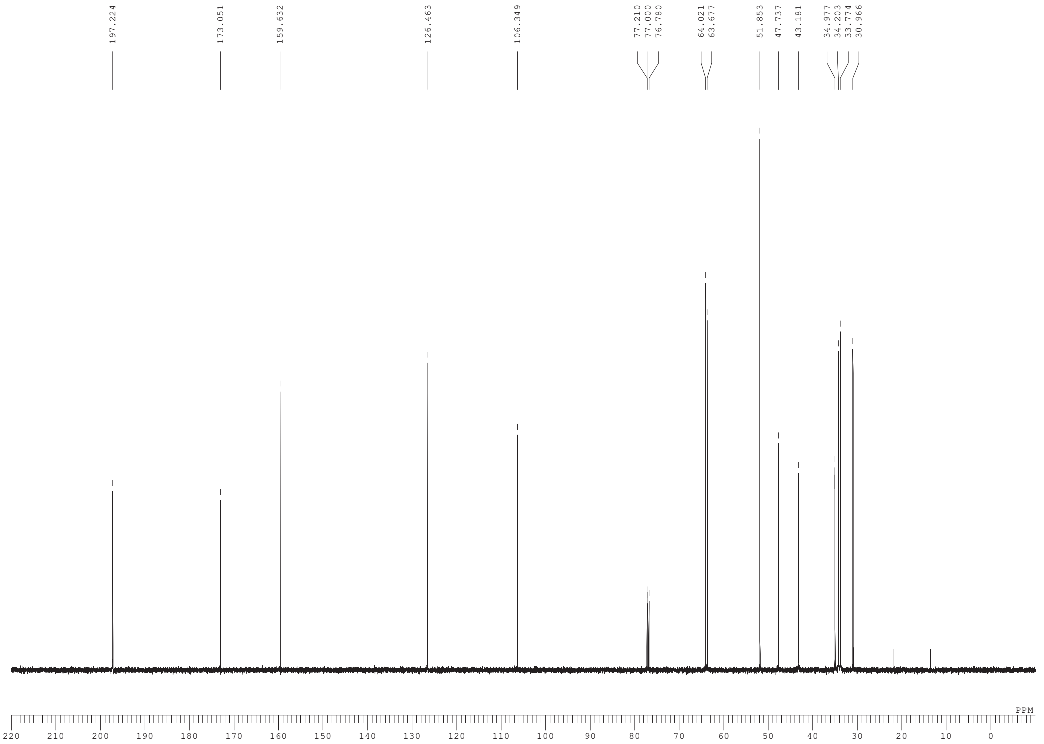

^{1}H-NMR (400 MHz, $CDCl_3$)

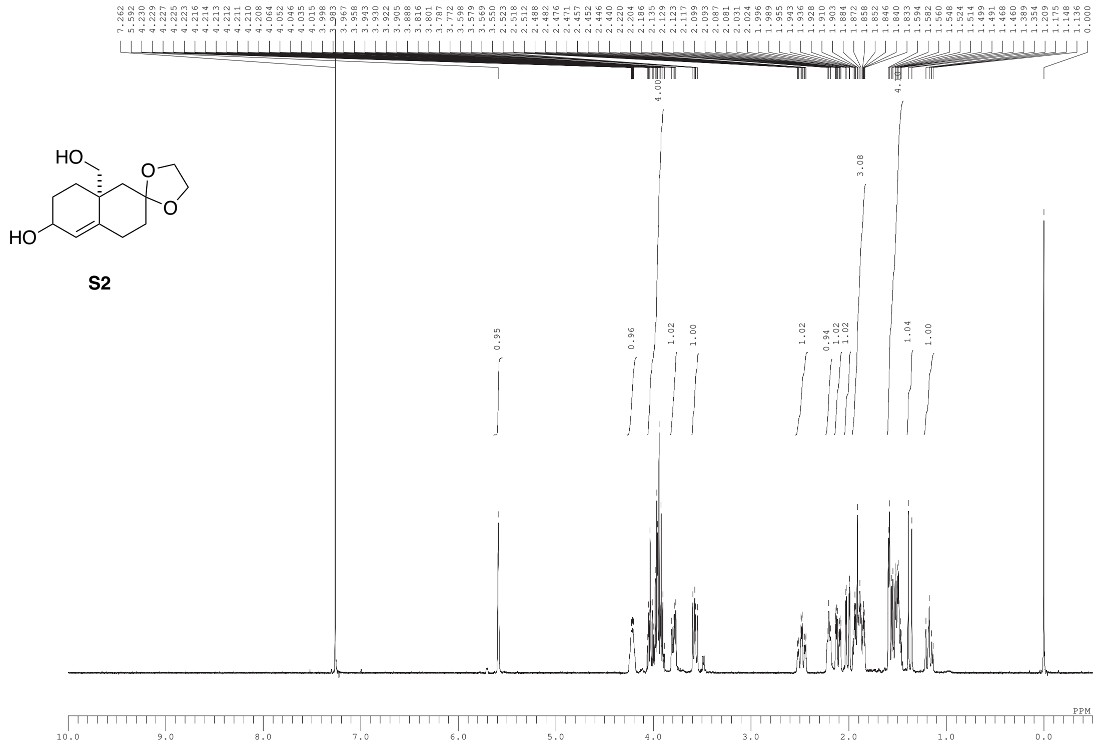

^{13}C-NMR (150 MHz, $CDCl_3$)

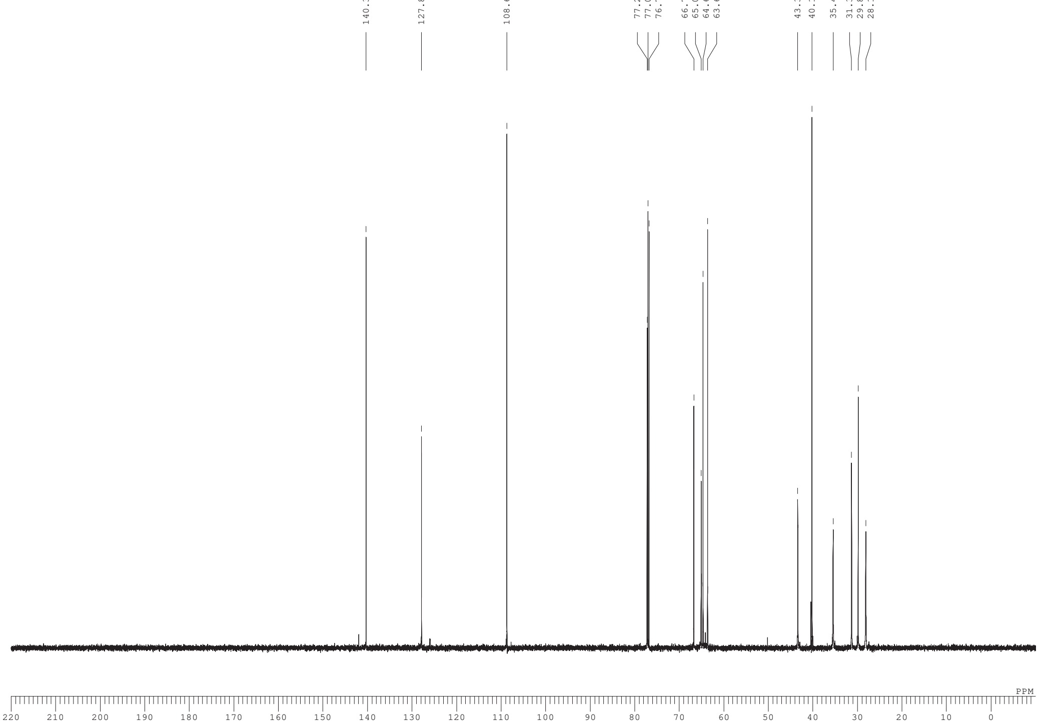

^{1}H-NMR (400 MHz, $CDCl_3$)

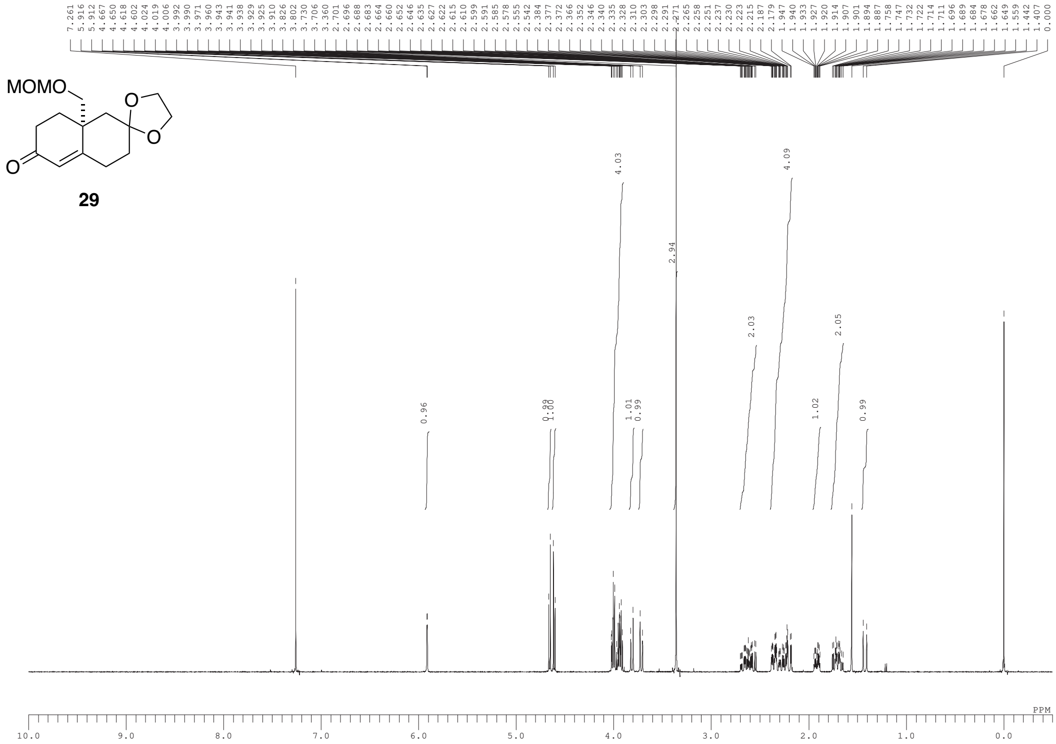

29

^{13}C-NMR (150 MHz, $CDCl_3$)

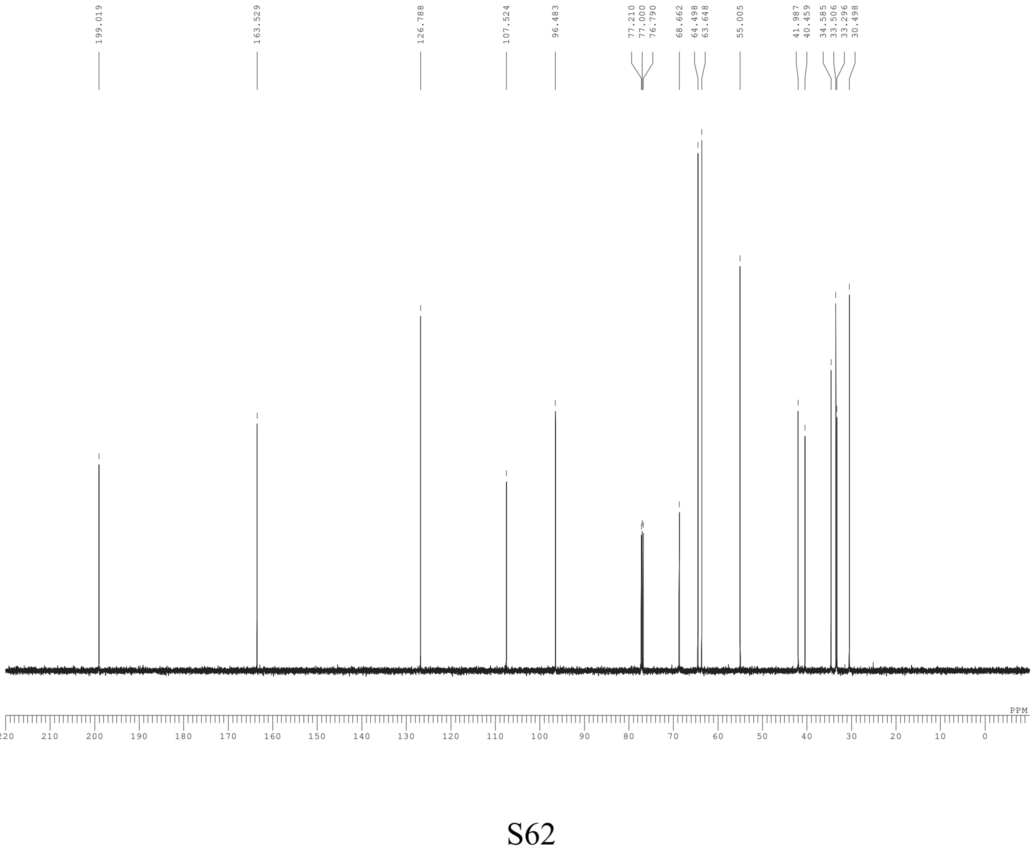

^{1}H-NMR (600 MHz, C_6D_6)

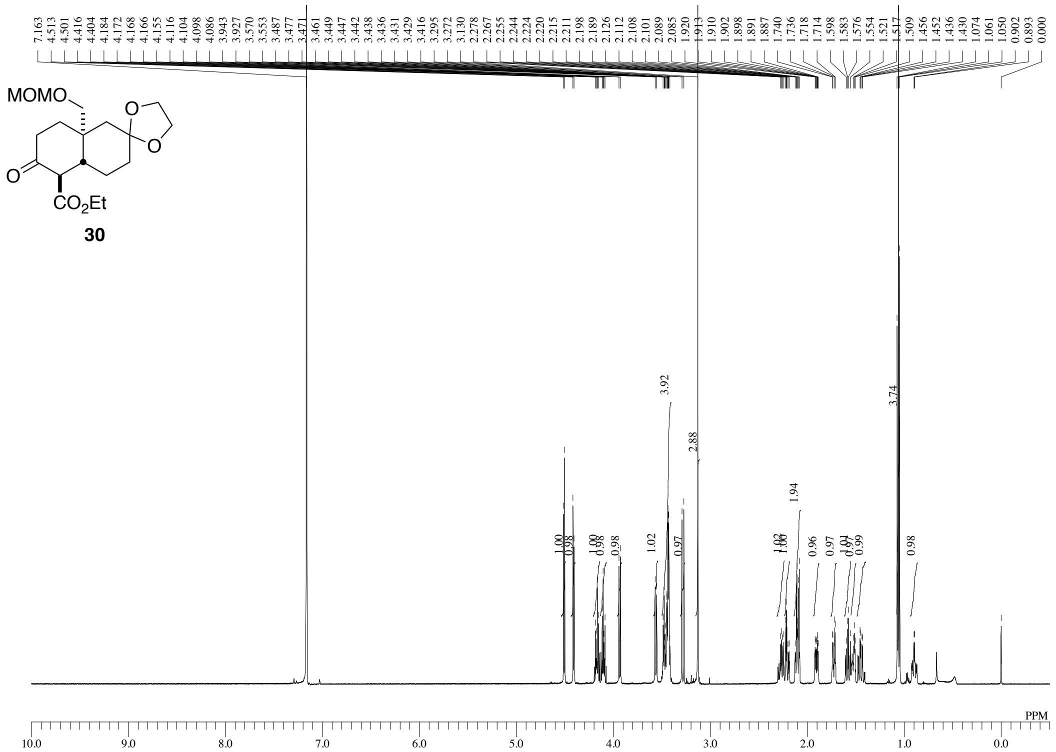

^{13}C-NMR (150 MHz, $CDCl_3$)

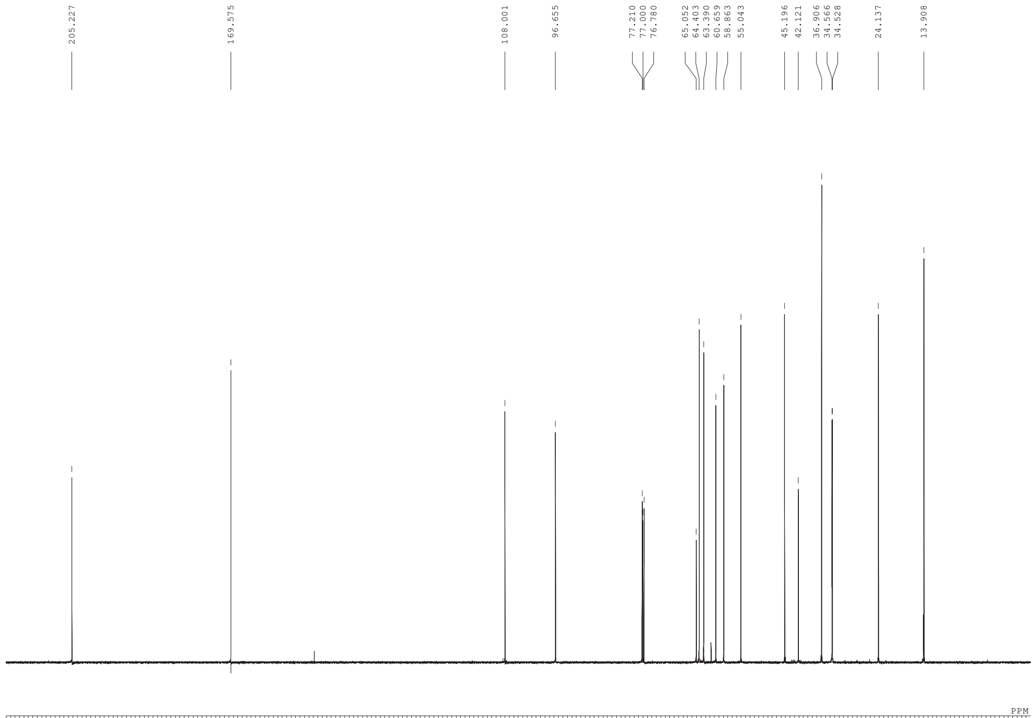

^{1}H-NMR (400 MHz, $CDCl_3$)

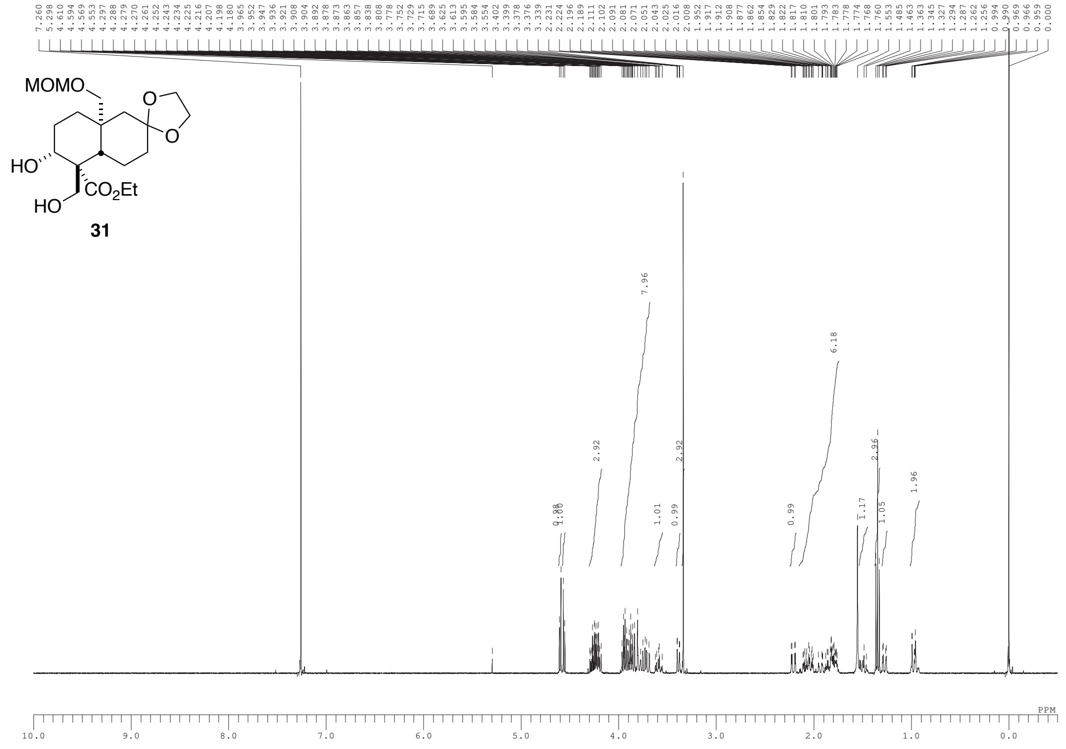

^{13}C-NMR (150 MHz, $CDCl_3$)

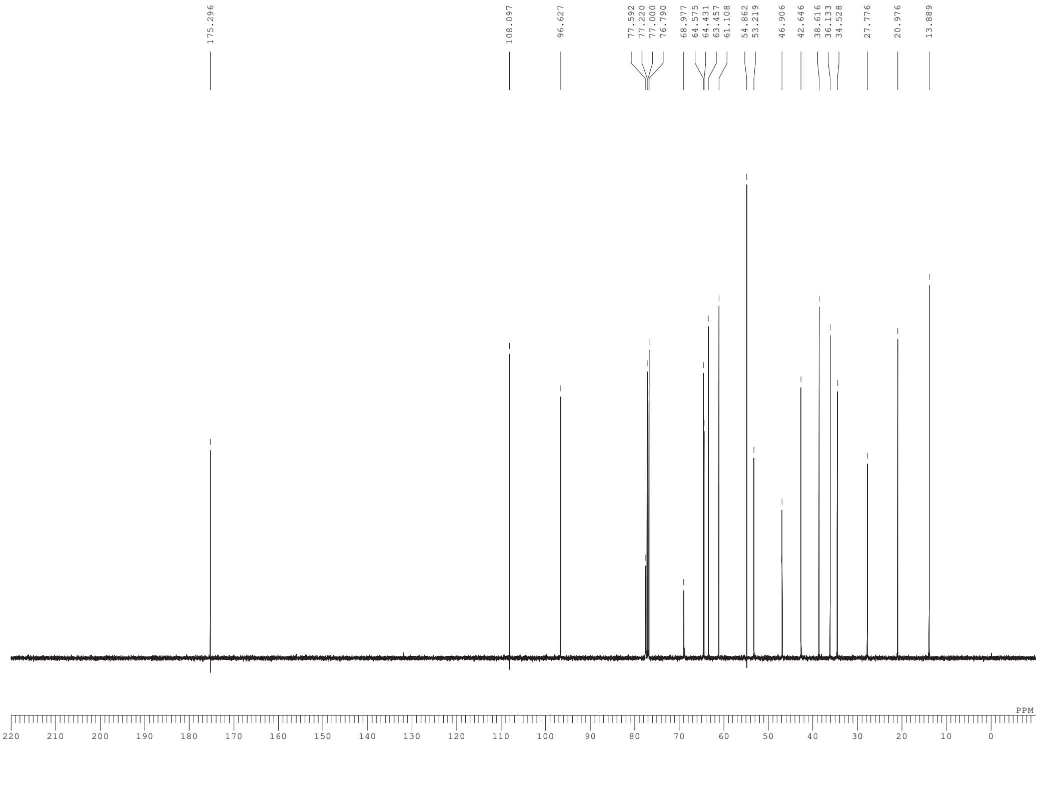

^{1}H-NMR (400 MHz, $CDCl_3$)

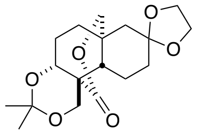

34

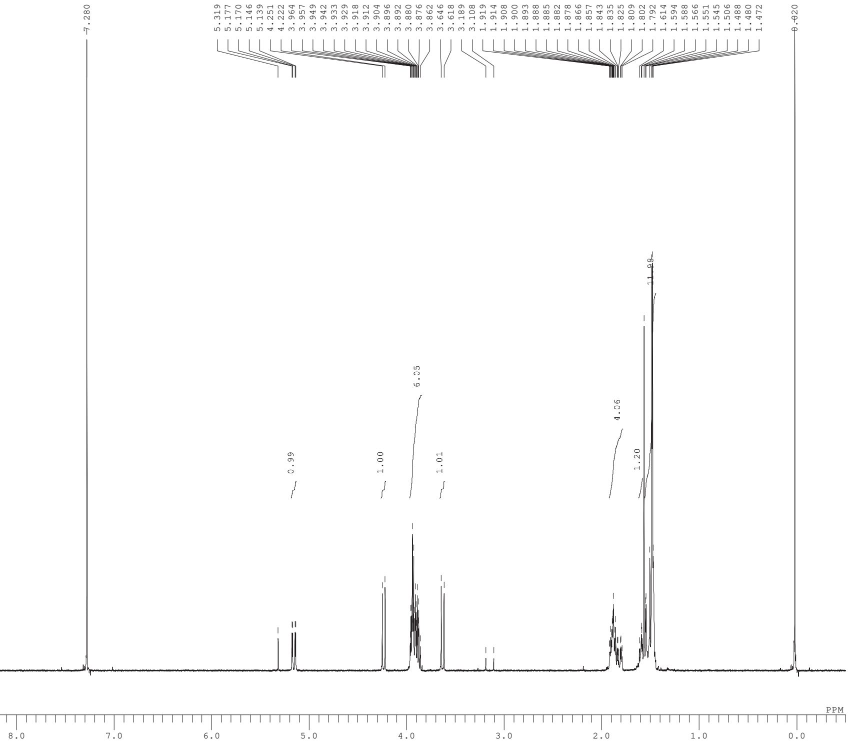

^{13}C-NMR (150 MHz, $CDCl_3$)

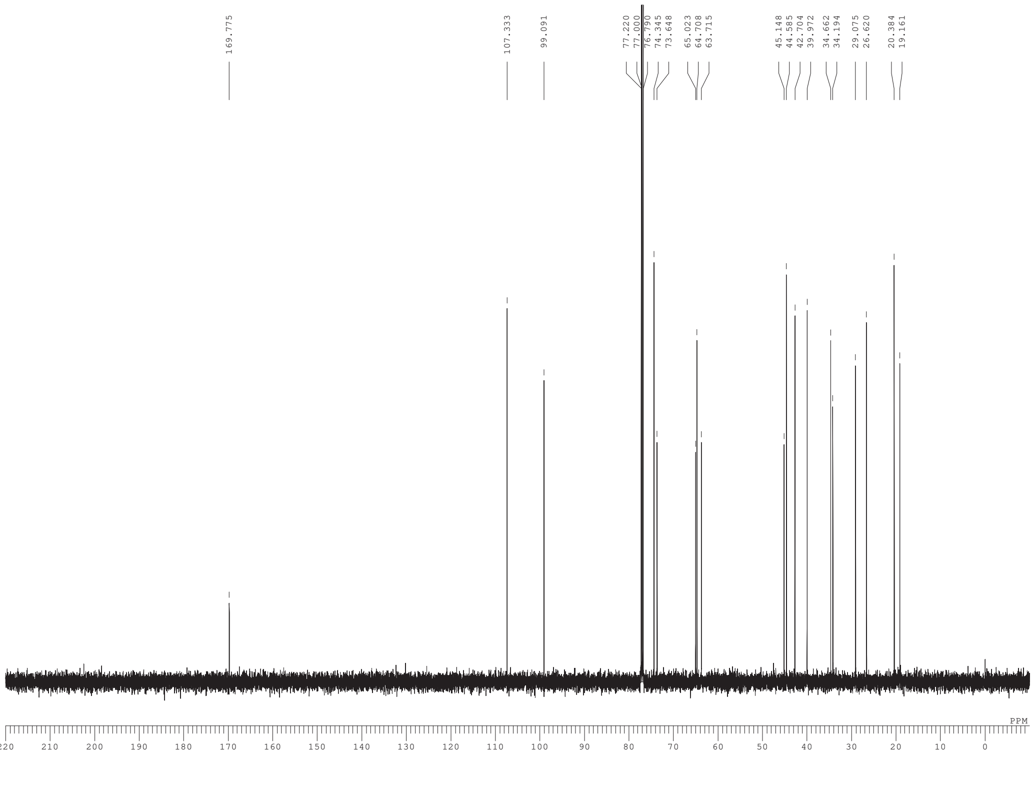

^{1}H-NMR (600 MHz, $CDCl_3$)

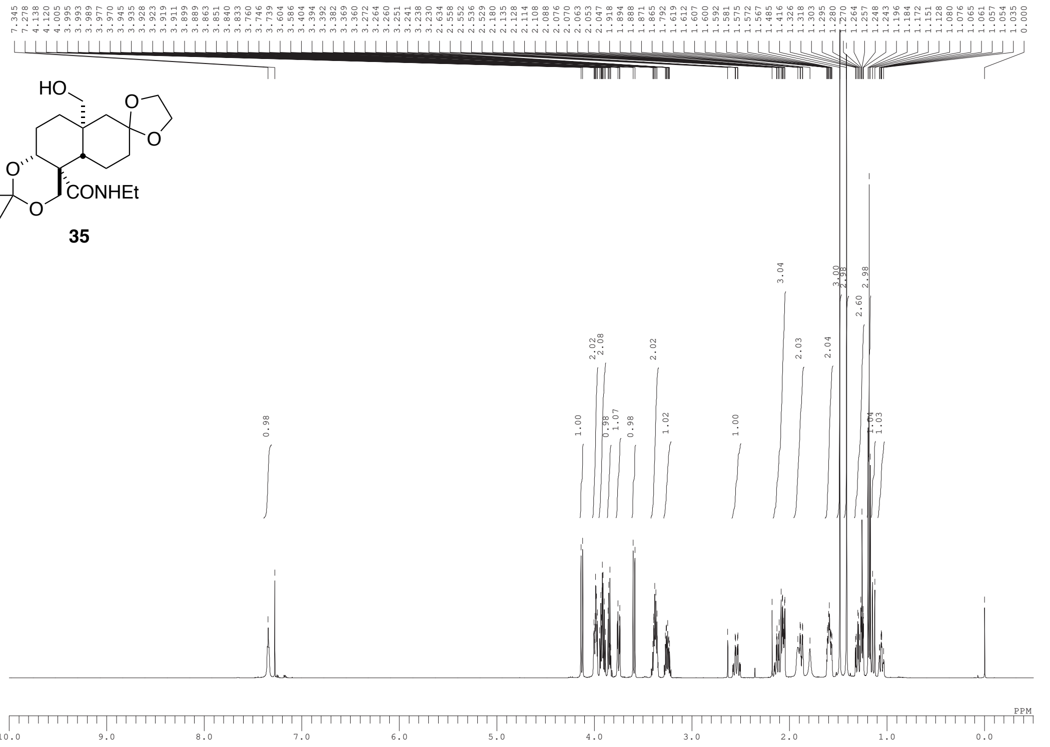

^{13}C-NMR (150 MHz, $CDCl_3$)

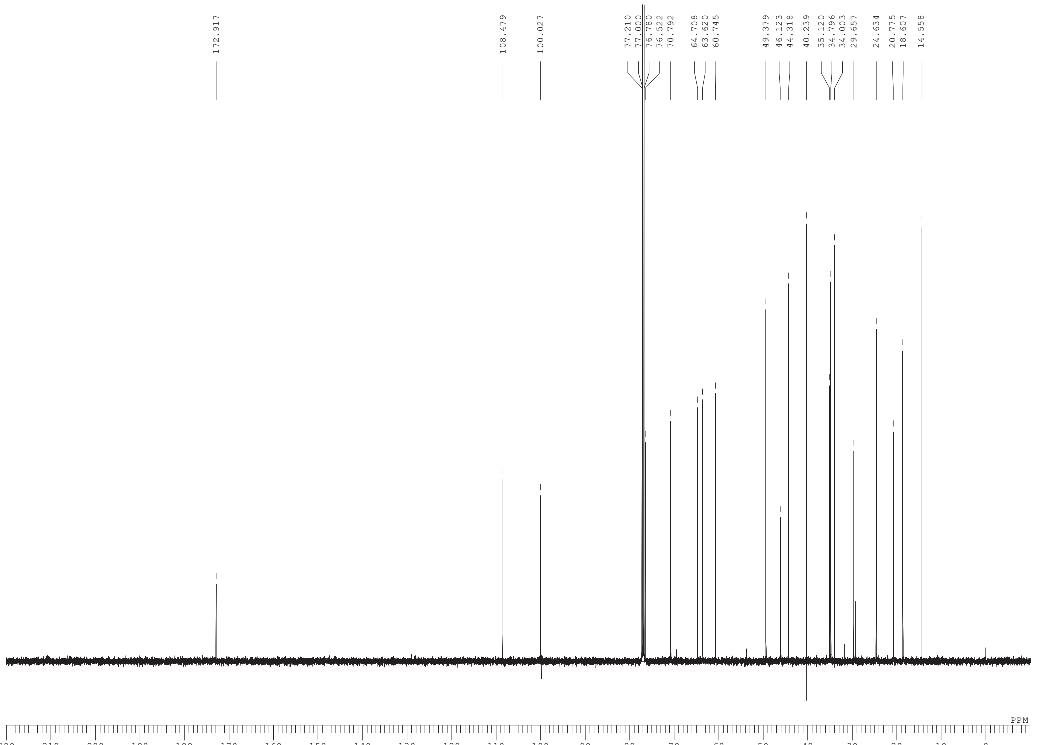

^{1}H-NMR (400 MHz, CD_3OD)

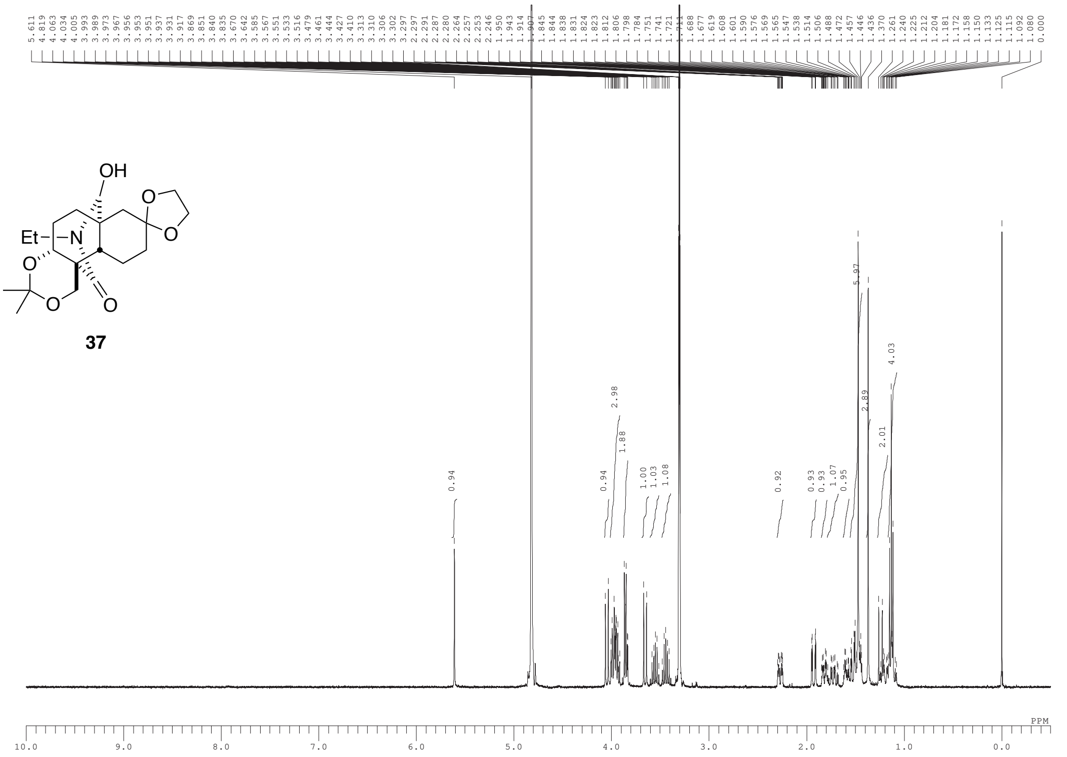

^{13}C-NMR (150 MHz, CD_3OD)

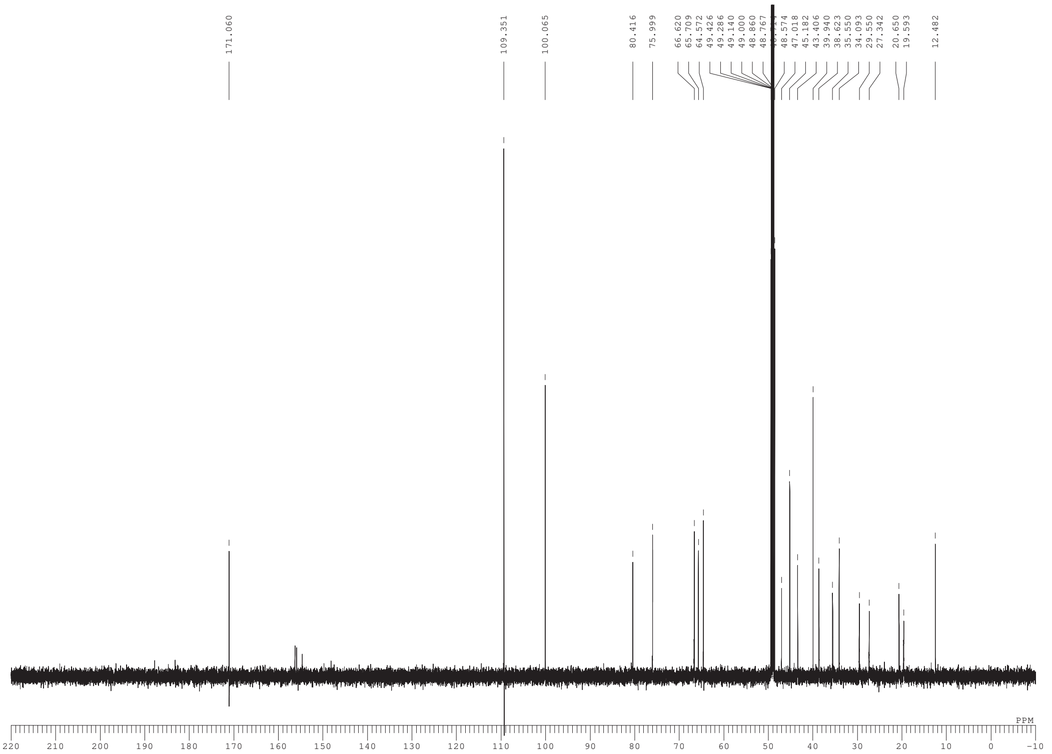

^{1}H-NMR (400 MHz, $CDCl_3$)

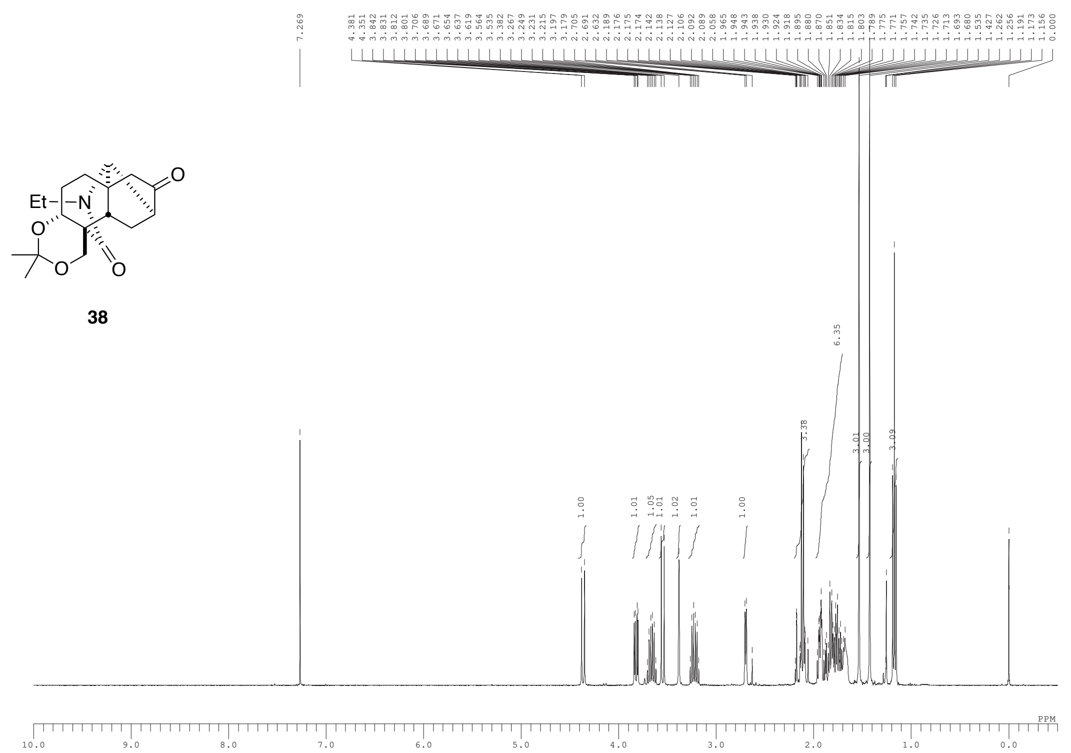

^{13}C-NMR (150 MHz, $CDCl_3$)

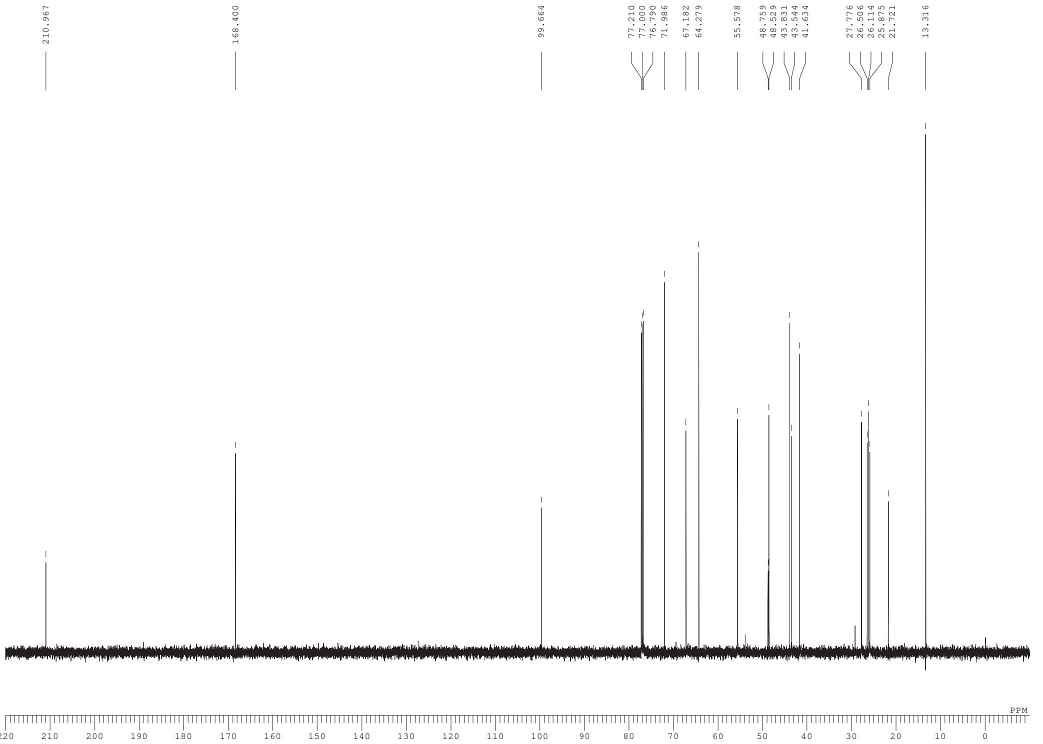

^{1}H-NMR (600 MHz, $CDCl_3$)

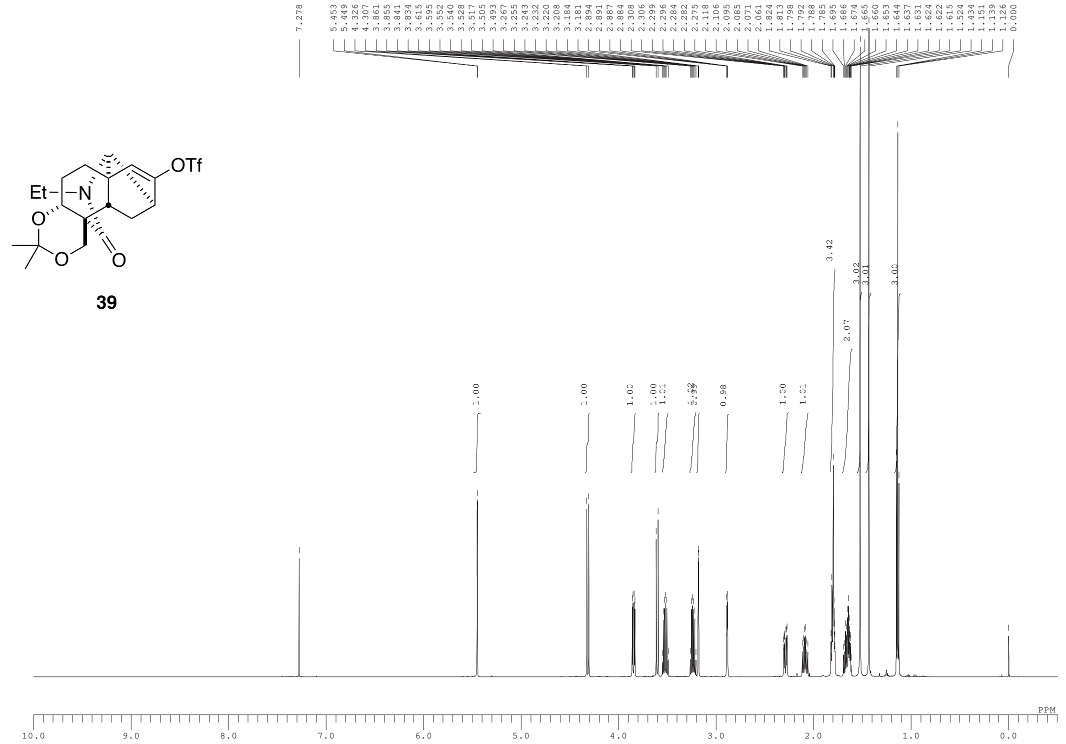

^{13}C-NMR (150 MHz, $CDCl_3$)

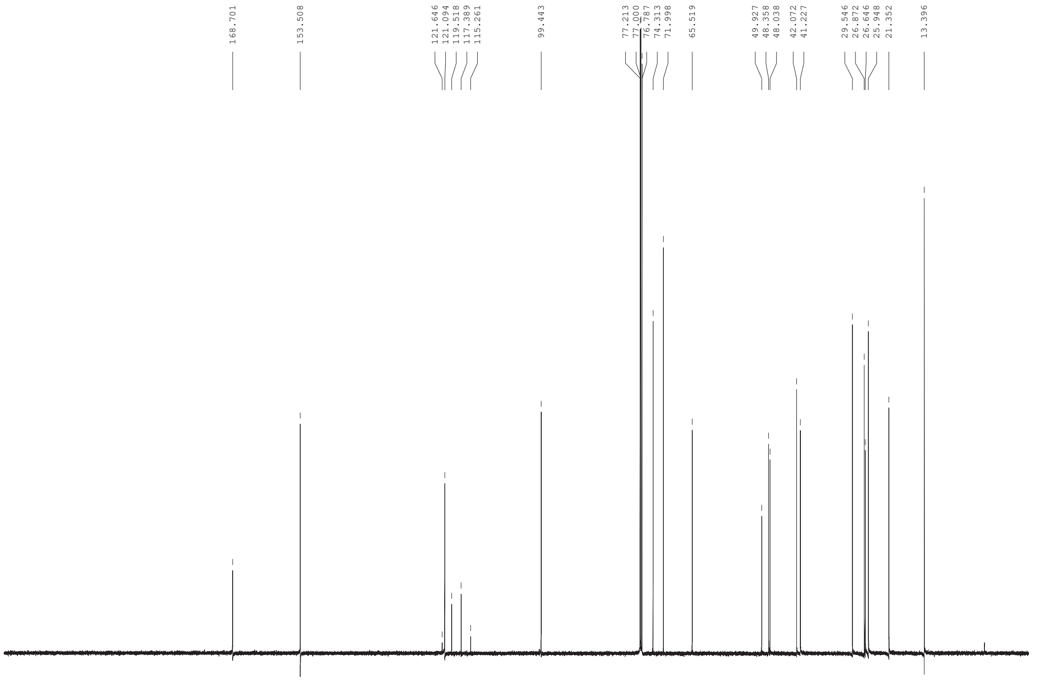

^{1}H-NMR (400 MHz, $CDCl_3$)

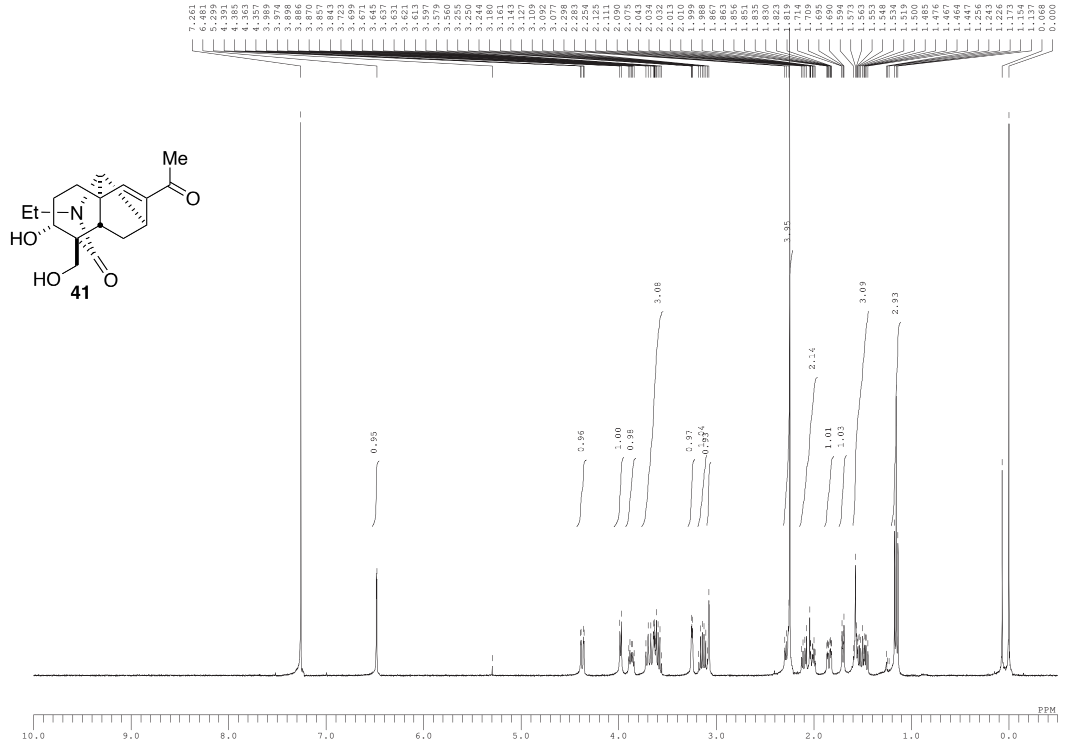

^{13}C-NMR (100 MHz, $CDCl_3$)

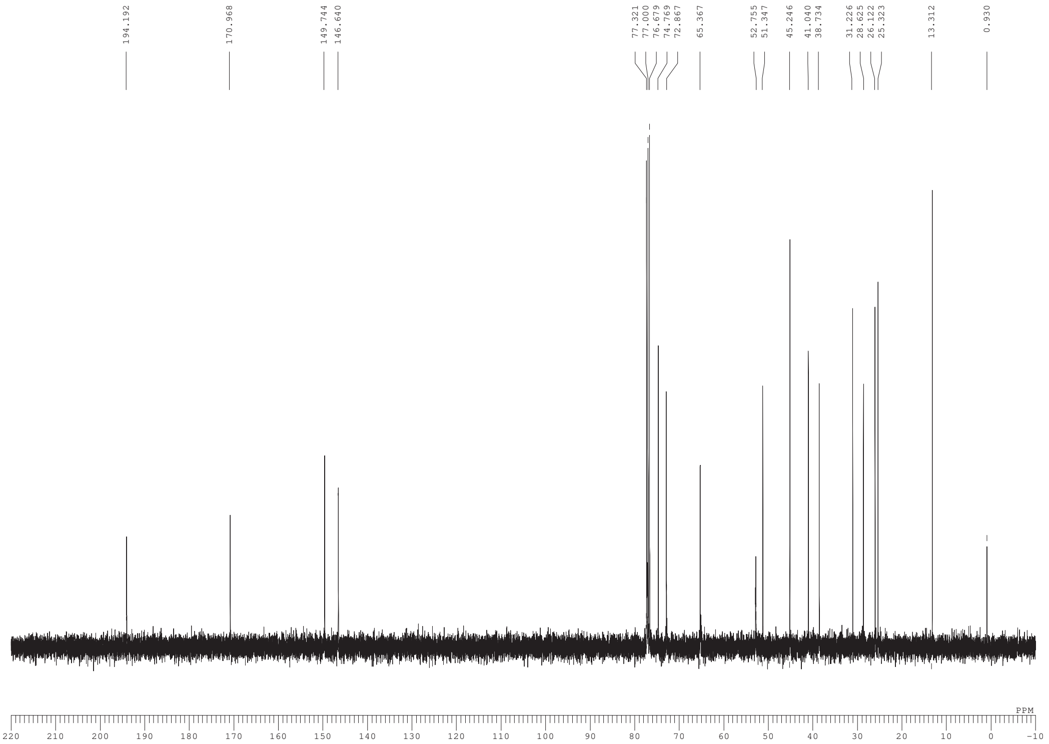

^{1}H-NMR (600 MHz, $CDCl_3$)

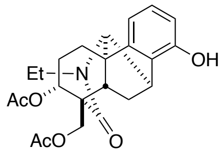

45

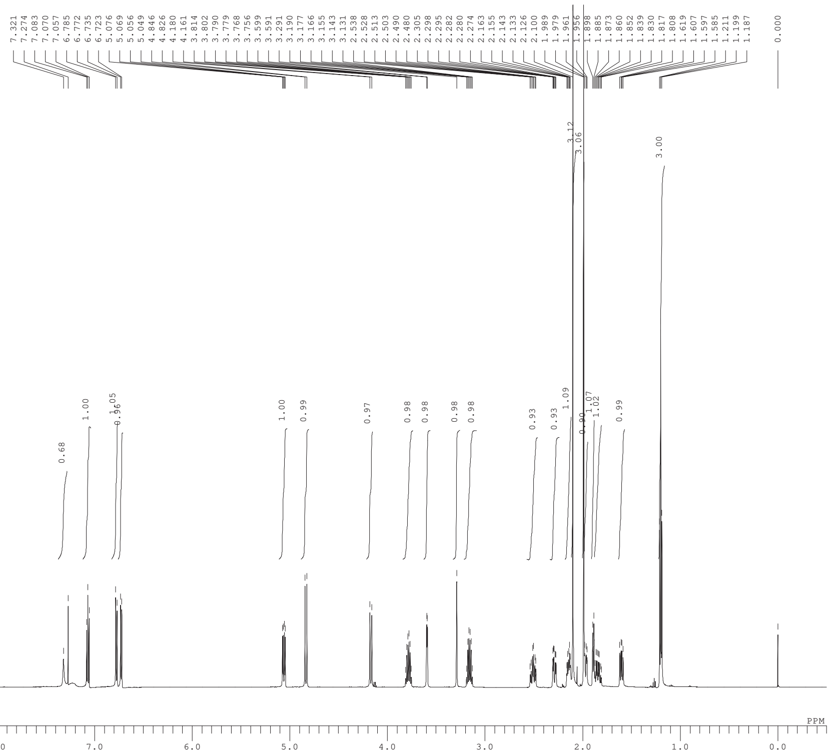

^{13}C-NMR (150 MHz, $CDCl_3$)

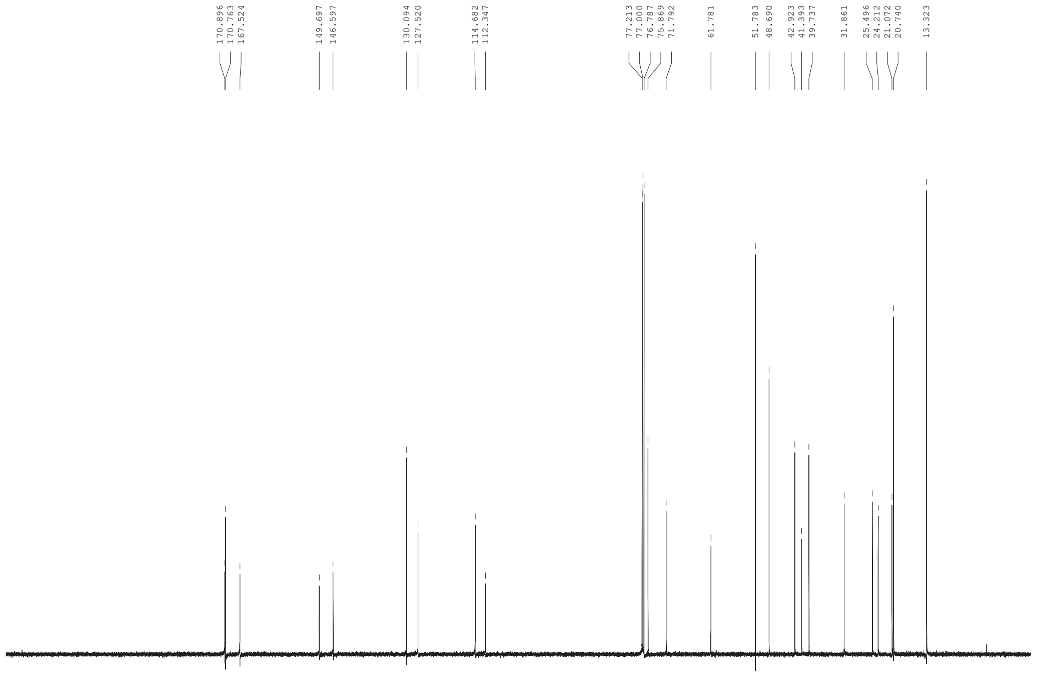

^{1}H-NMR (400 MHz, $CDCl_3$)

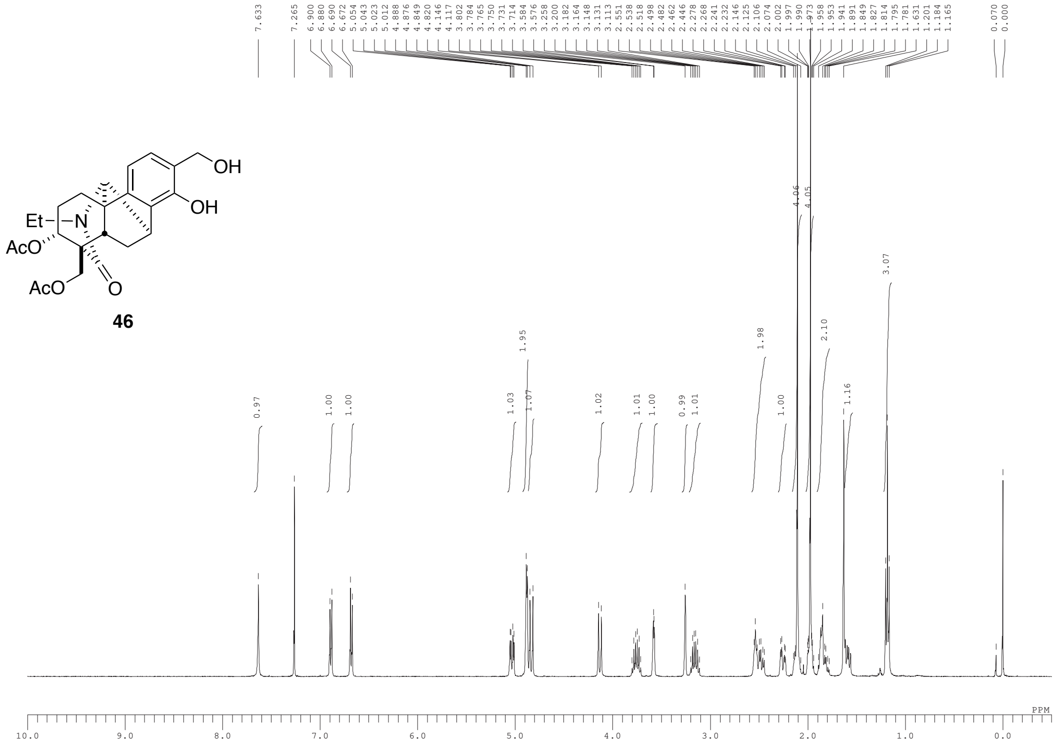

46

^{13}C-NMR (100 MHz, $CDCl_3$)

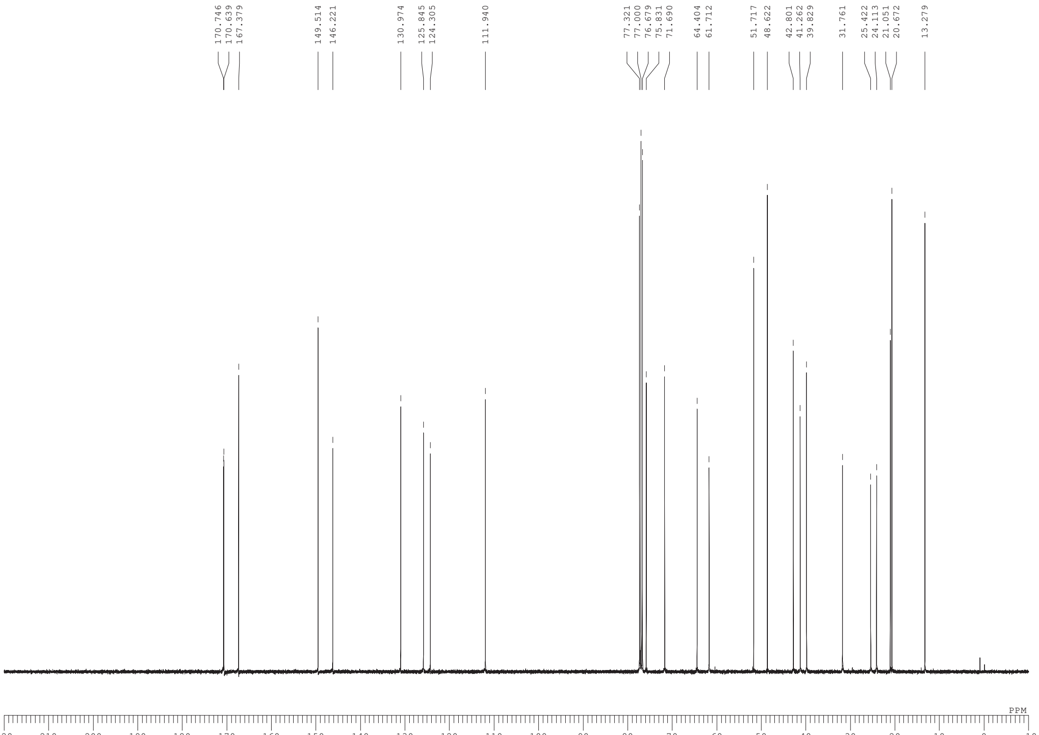

^{1}H-NMR (400 MHz, $CDCl_3$)

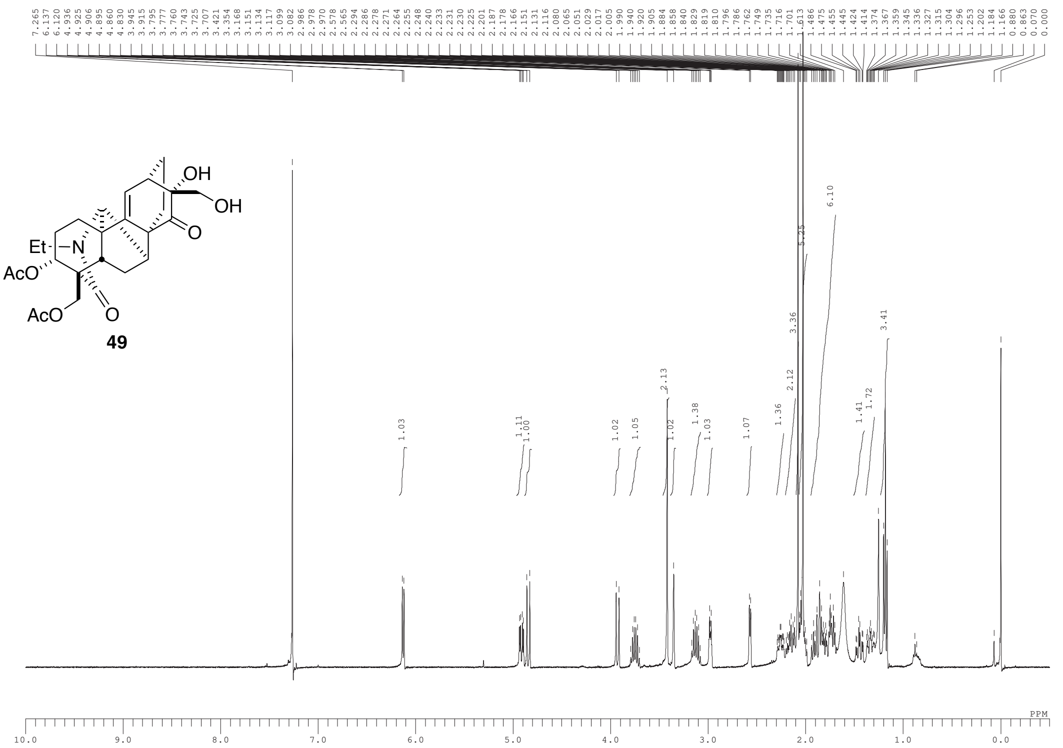

^{13}C-NMR (100 MHz, $CDCl_3$)

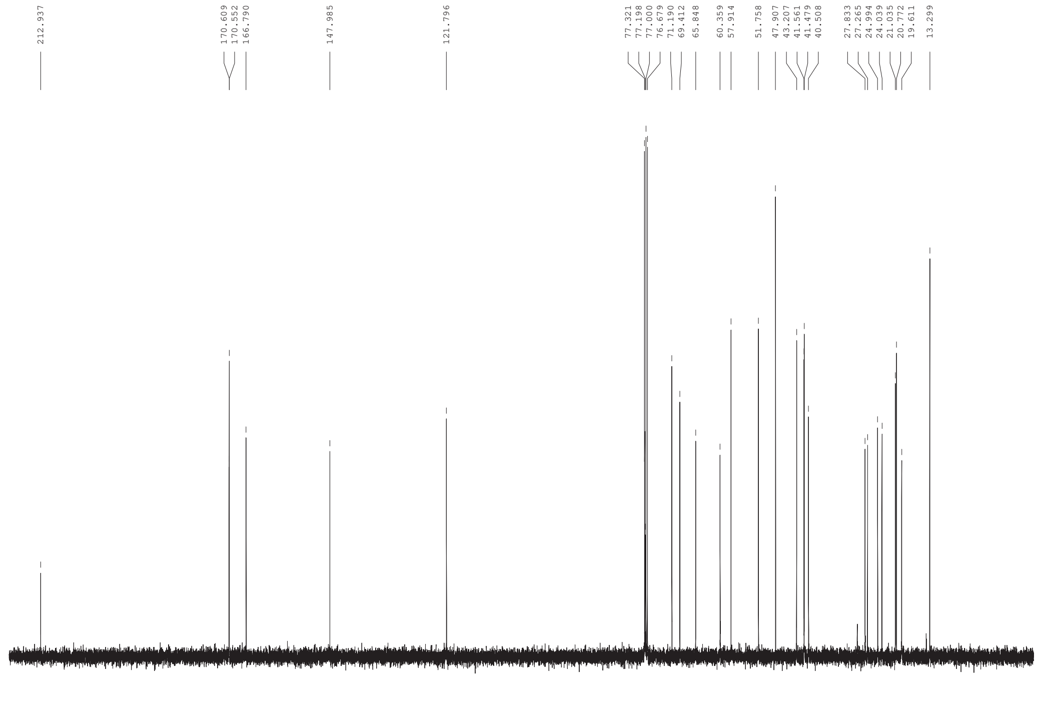

^{1}H-NMR (600 MHz, $CDCl_3$)

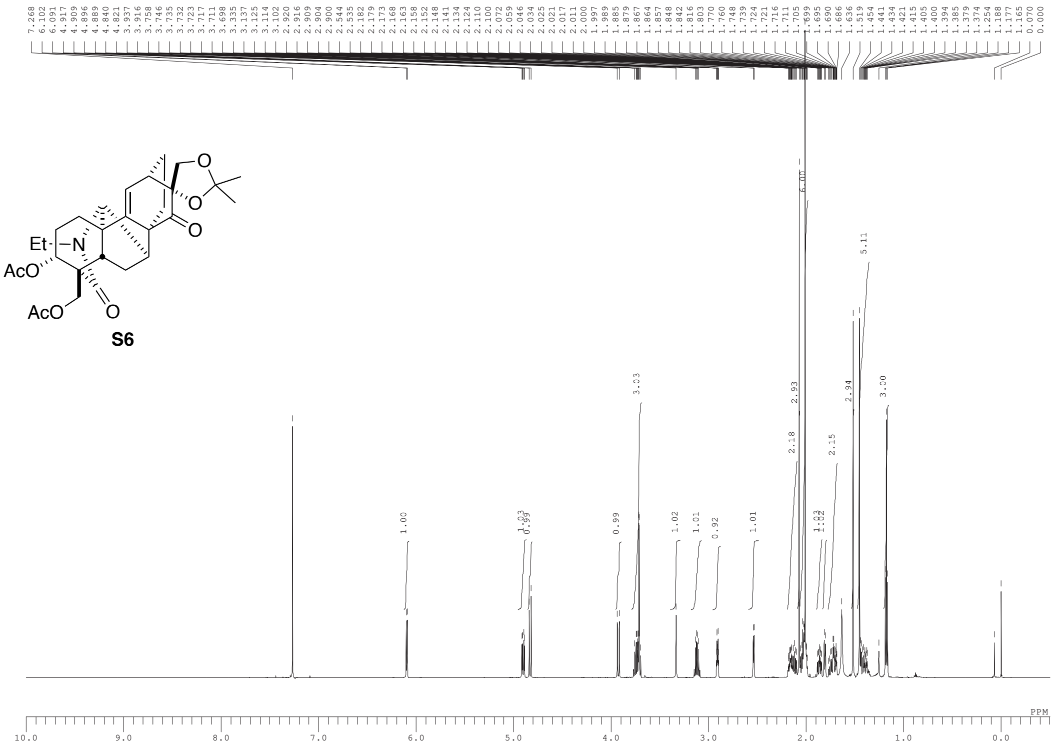

^{13}C-NMR (100 MHz, $CDCl_3$)

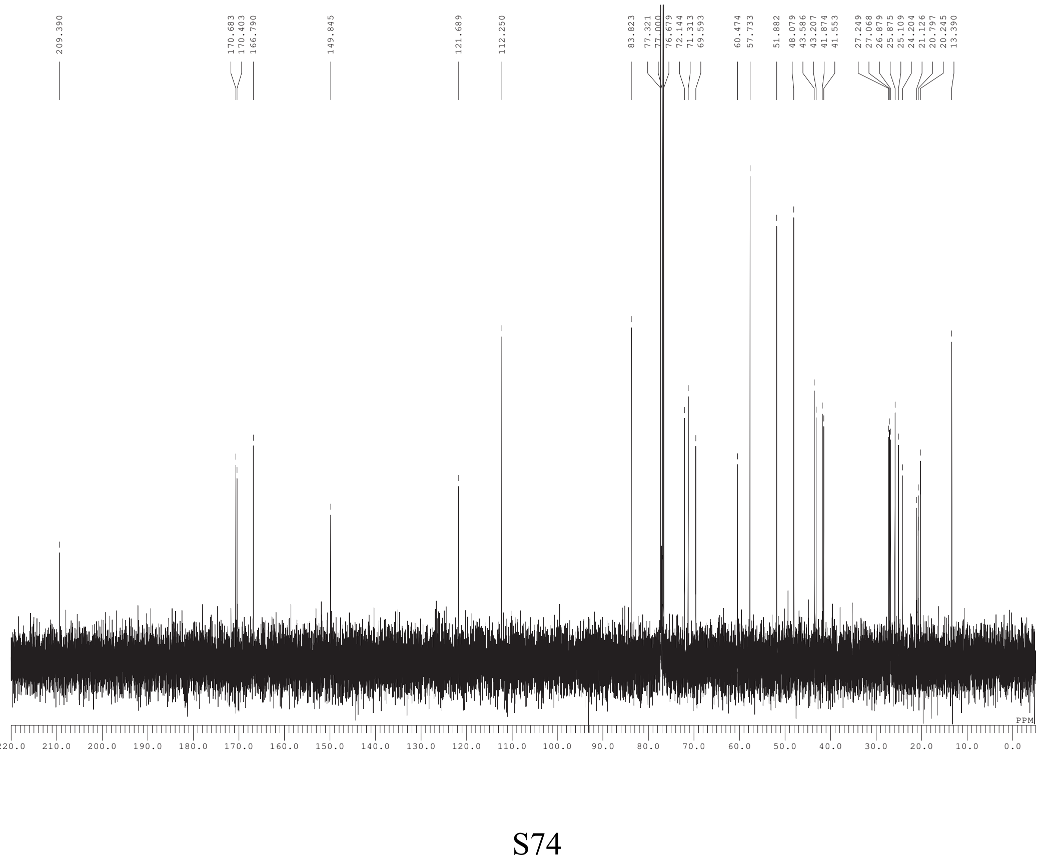

COSY ($CDCl_3$)

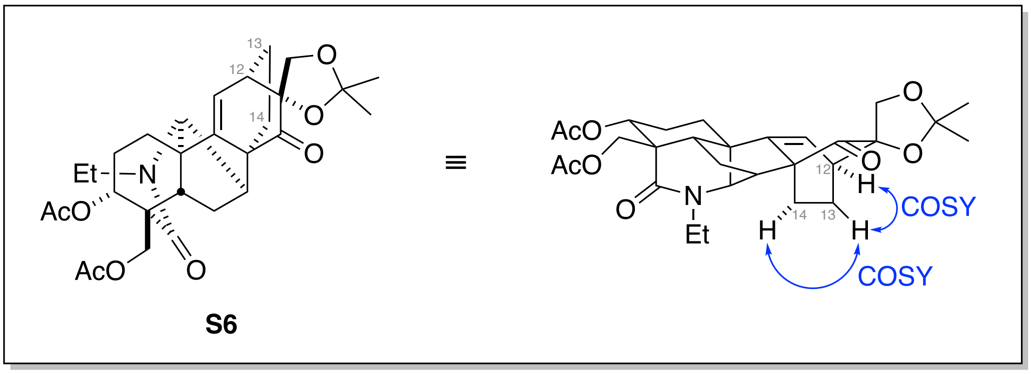

13
12
14
Et
N
AcO
AcO
O
O
O
O
S6
≡
AcO
AcO
O
N
Et
O
O
O
12
H
14
13
H
H
COSY
COSY

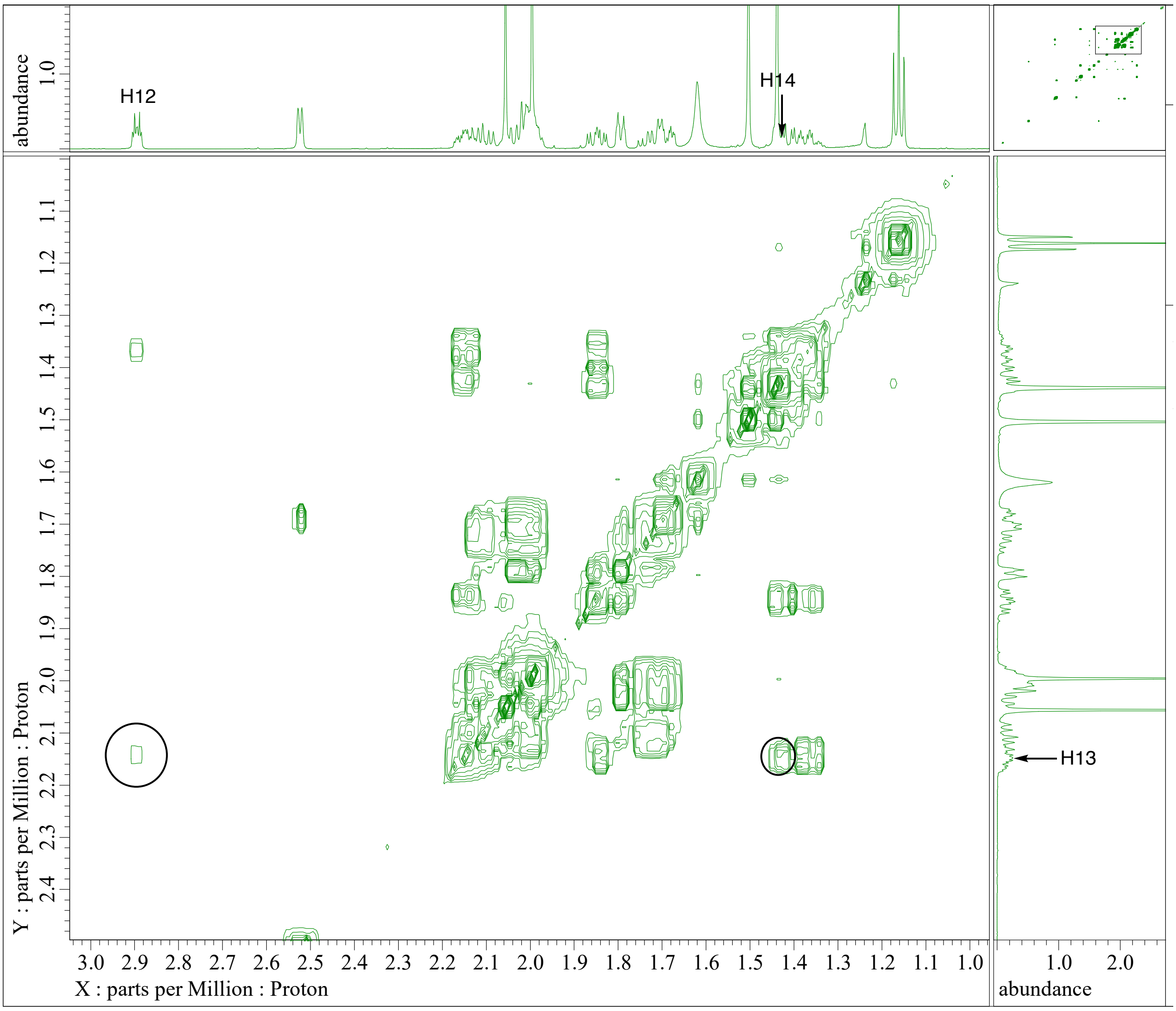

abundance
1.0
H12
H14
H13
Y : parts per Million : Proton
X : parts per Million : Proton
3.0 2.9 2.8 2.7 2.6 2.5 2.4 2.3 2.2 2.1 2.0 1.9 1.8 1.7 1.6 1.5 1.4 1.3 1.2 1.1 1.0
1.1 1.2 1.3 1.4 1.5 1.6 1.7 1.8 1.9 2.0 2.1 2.2 2.3 2.4
1.0 2.0
abundance

NOESY ($CDCl_3$)

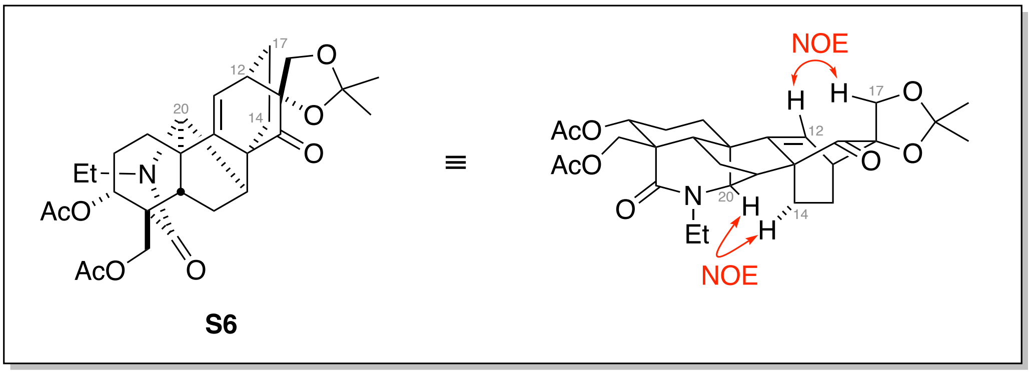

S6
NOE
NOE
AcO
AcO
Et
N
H
H
12
14
17
20

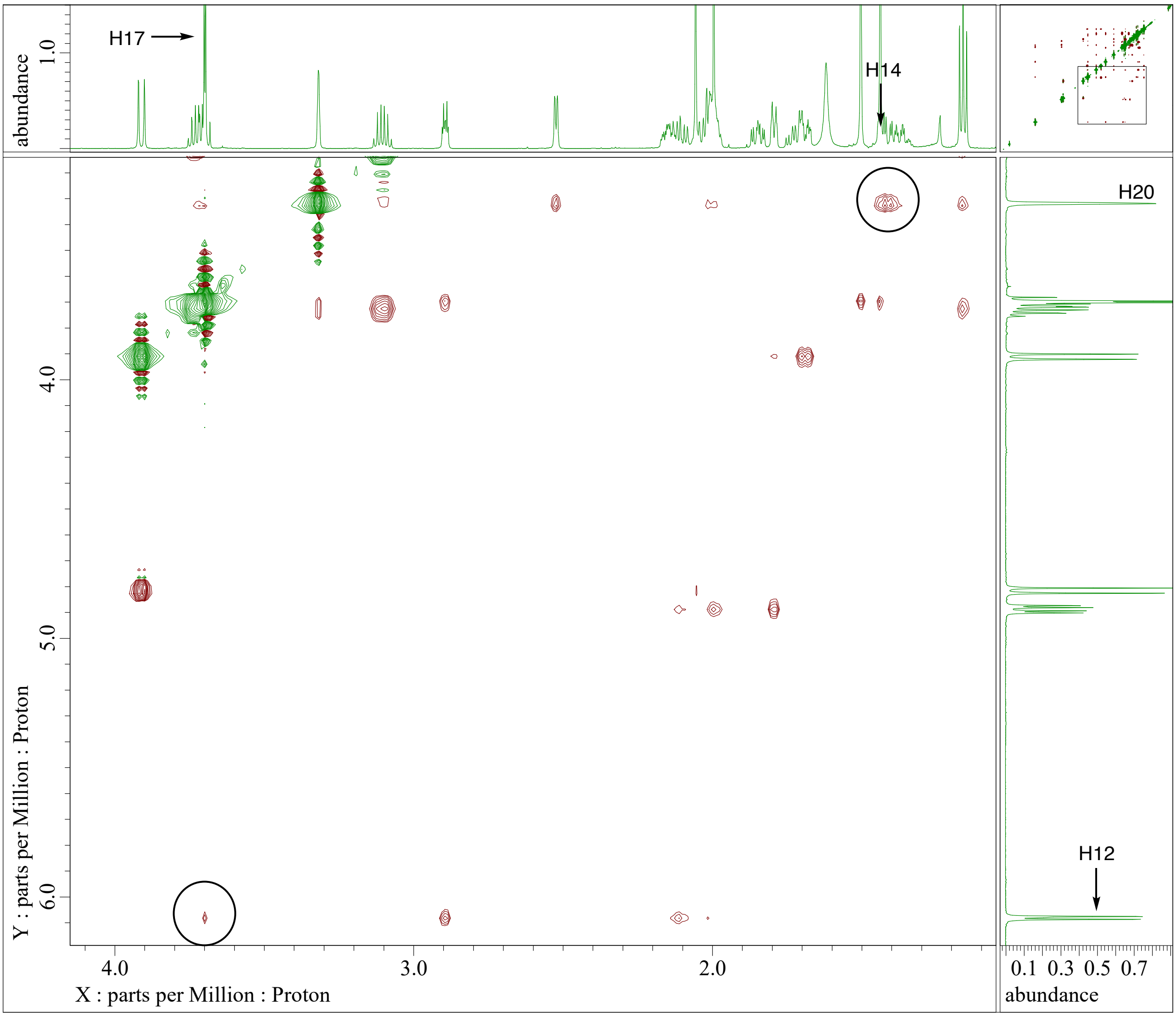

H17
H14
H20
H12
abundance
Y : parts per Million : Proton
X : parts per Million : Proton

^{1}H-NMR (400 MHz, $CDCl_3$)

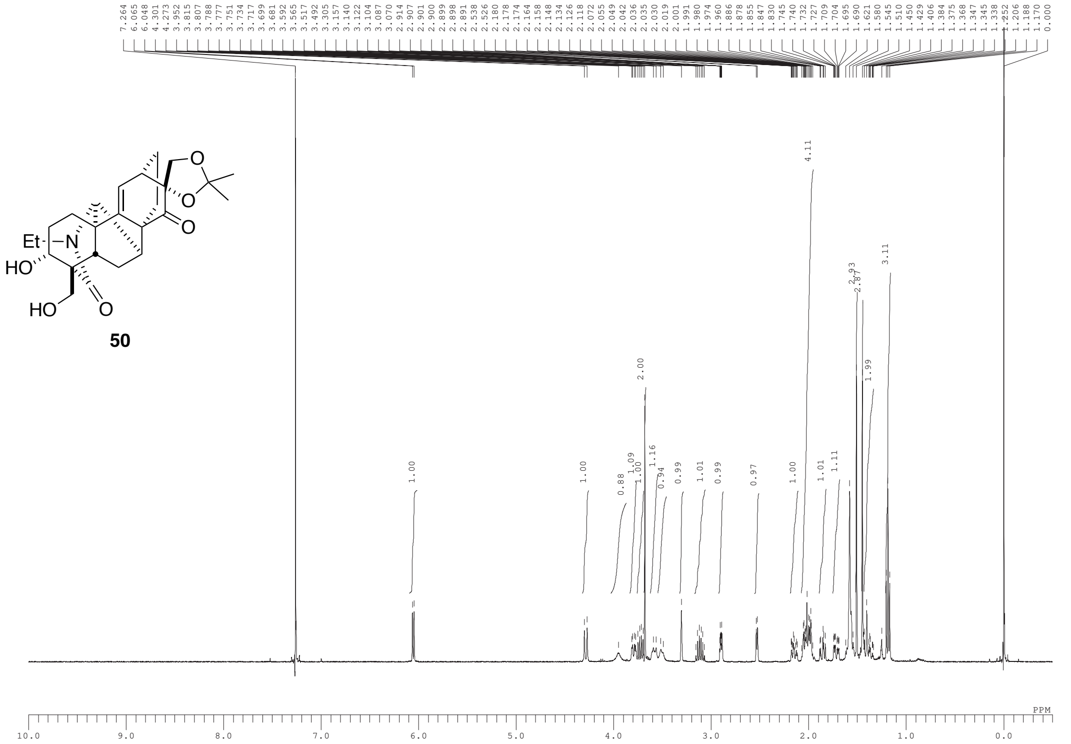

50

^{13}C-NMR (100 MHz, $CDCl_3$)

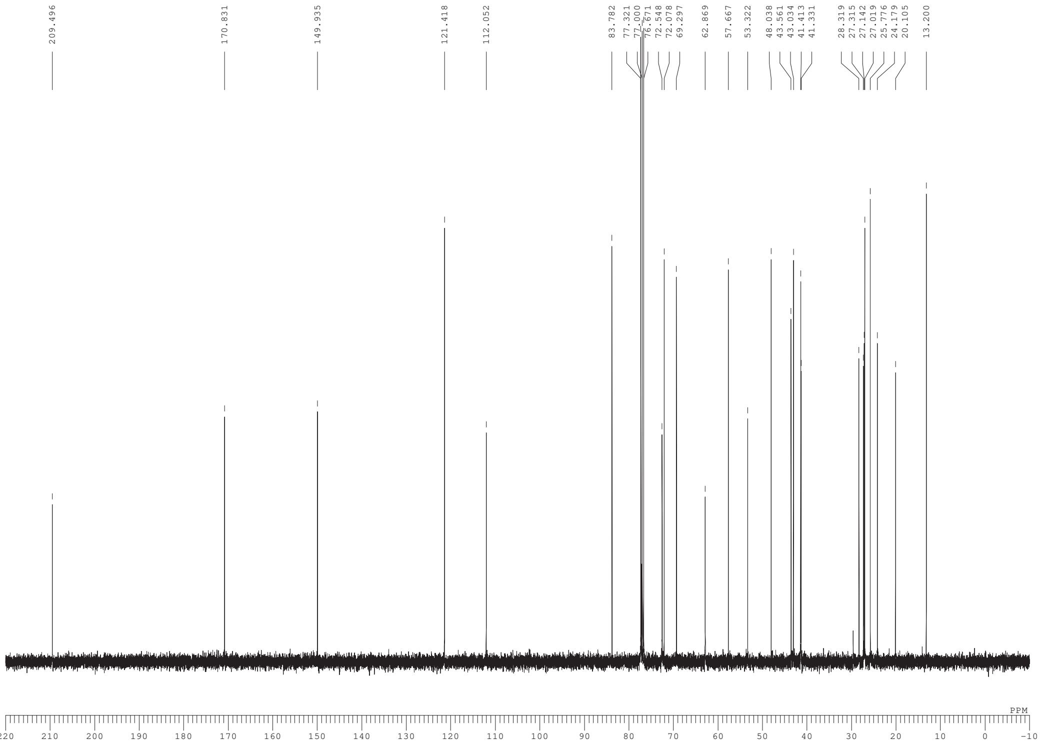

^{1}H-NMR (400 MHz, $CDCl_3$)

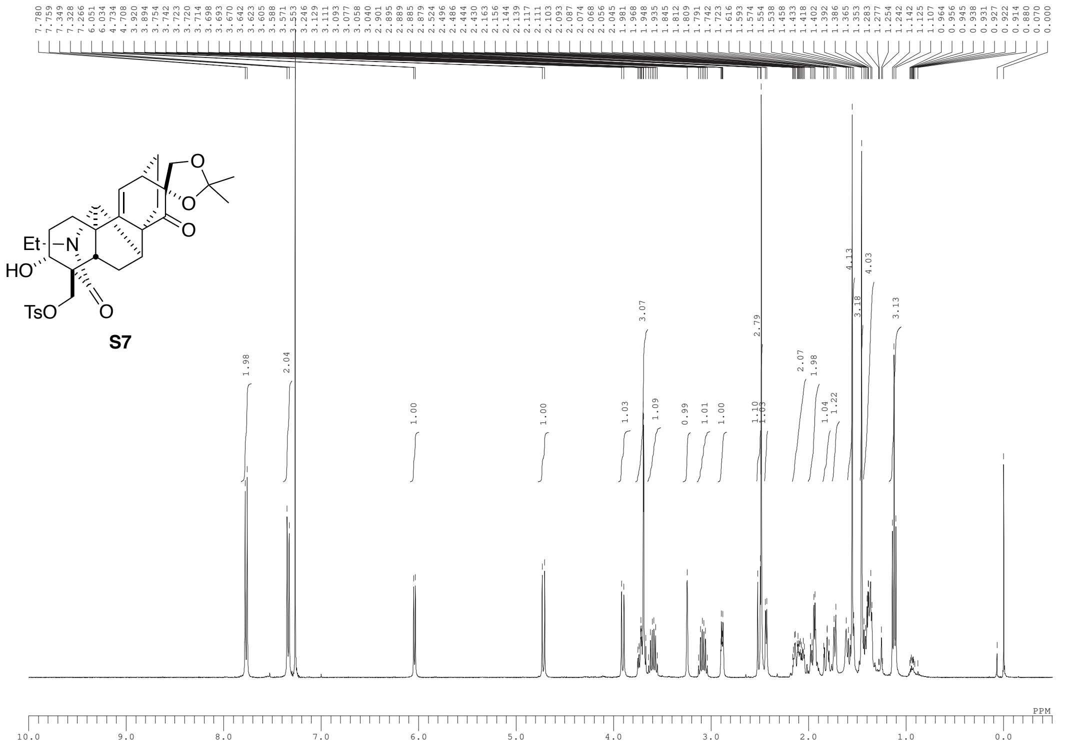

^{13}C-NMR (100 MHz, $CDCl_3$)

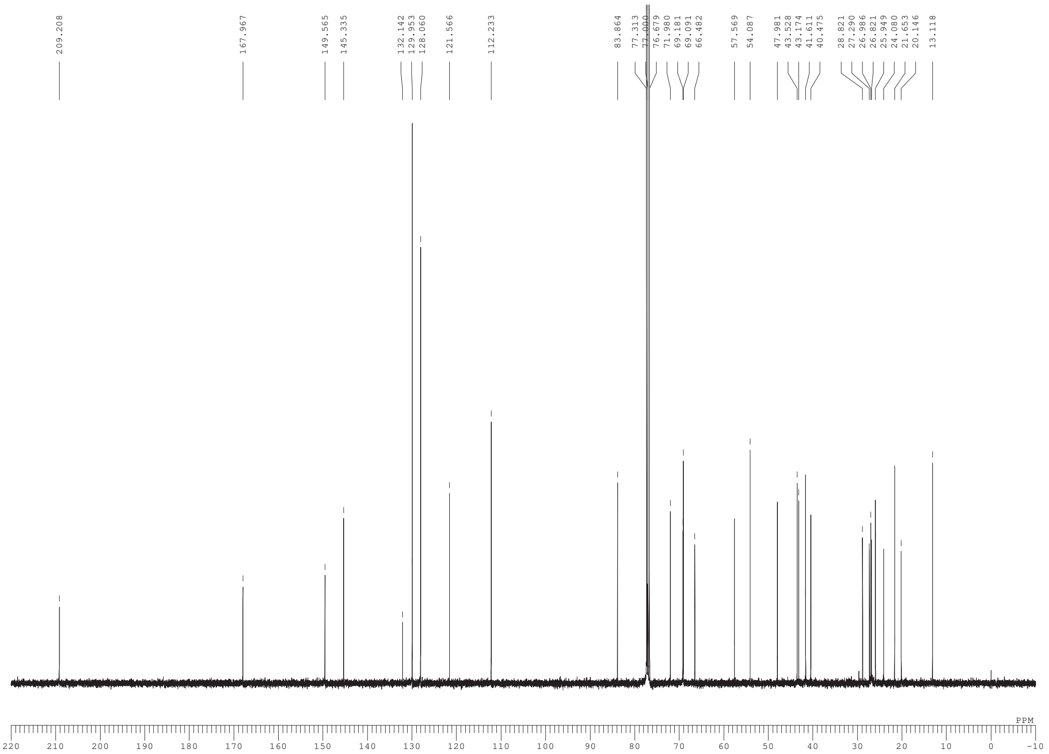

^{1}H-NMR (600 MHz, $CDCl_3$)

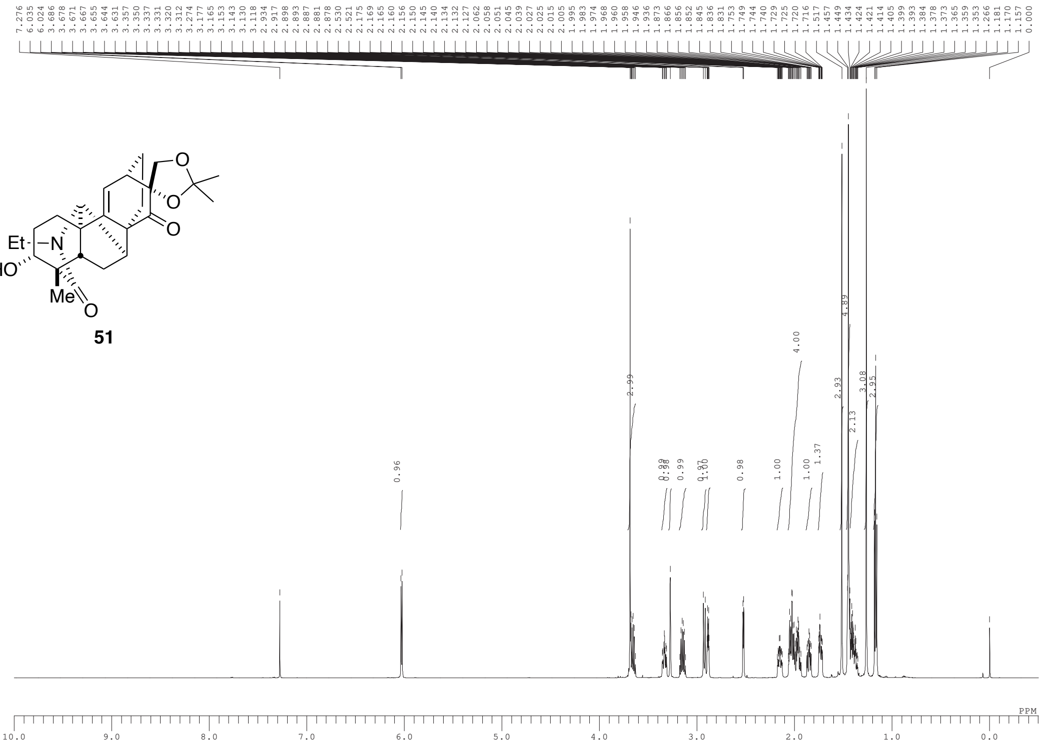

^{13}C-NMR (150 MHz, $CDCl_3$)

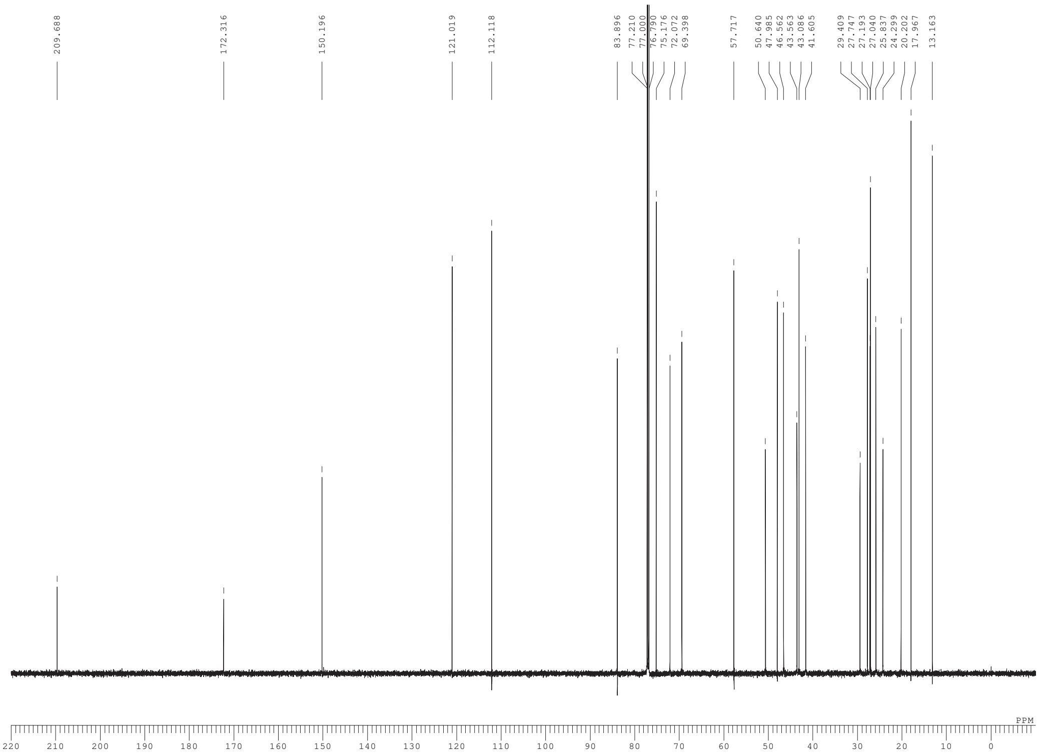

^{1}H-NMR (600 MHz, $CDCl_3$)

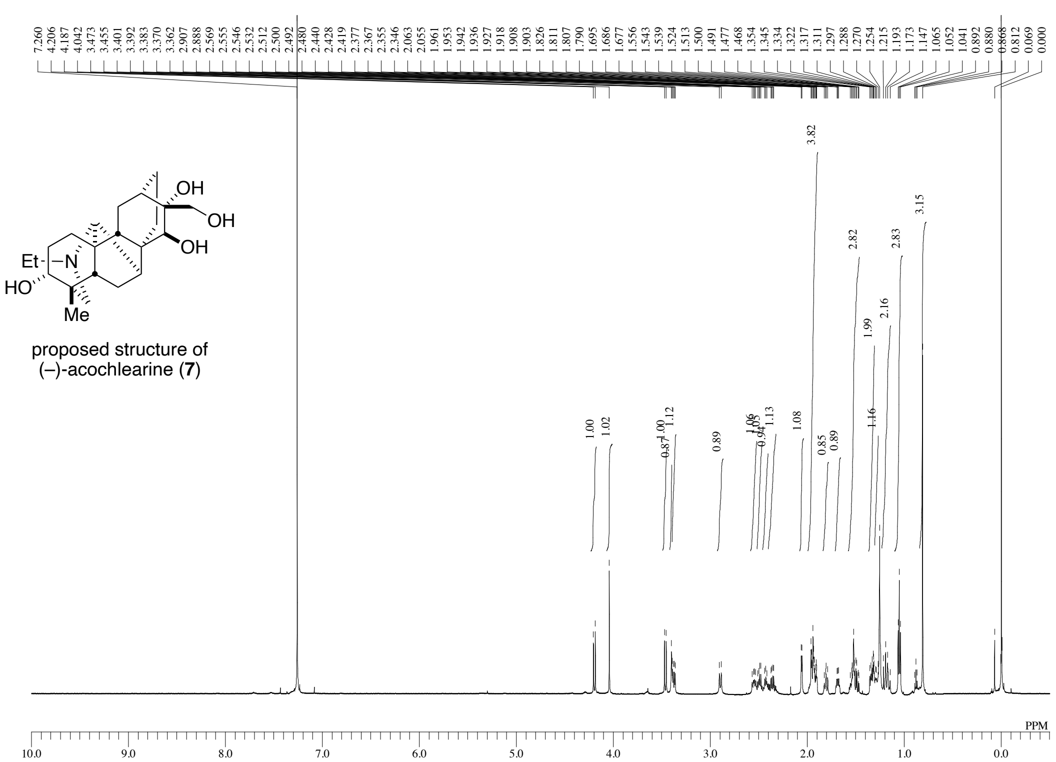

proposed structure of
(–)-acochlearine (**7**)

^{13}C-NMR (150 MHz, $CDCl_3$)

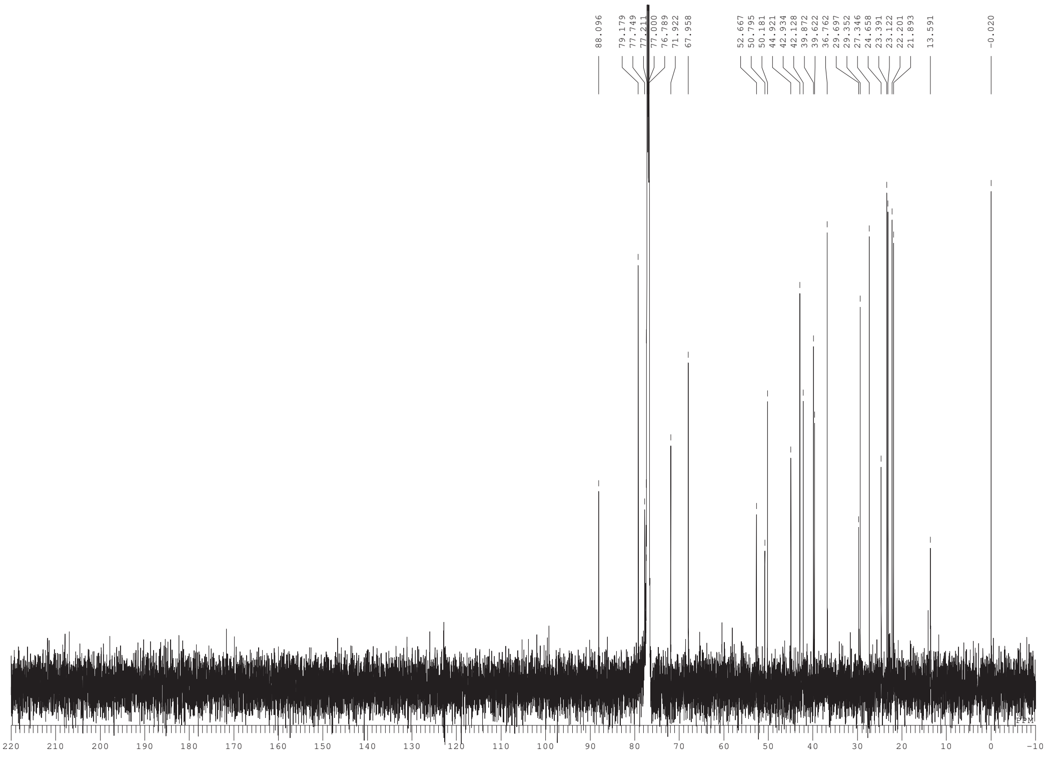

COSY ($CDCl_3$)

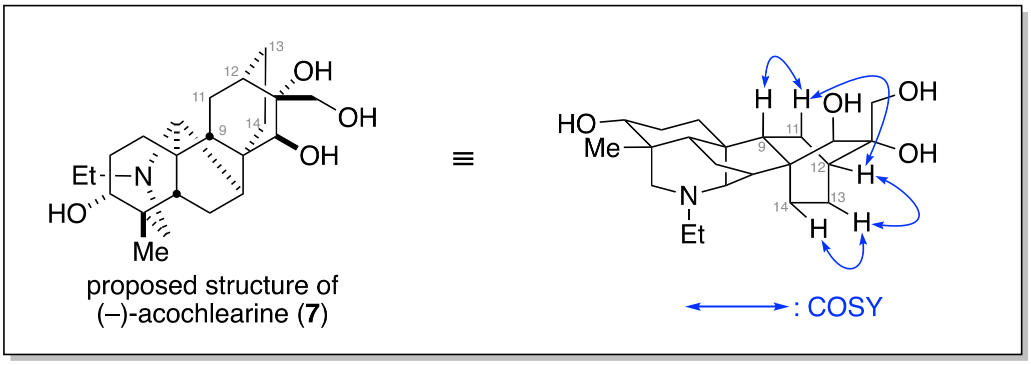

OH
OH
OH
Et
N
HO
Me
proposed structure of
(–)-acochlearine (7)
≡
HO
Me
N
Et
H
H
OH
OH
OH
H
H
H
: COSY

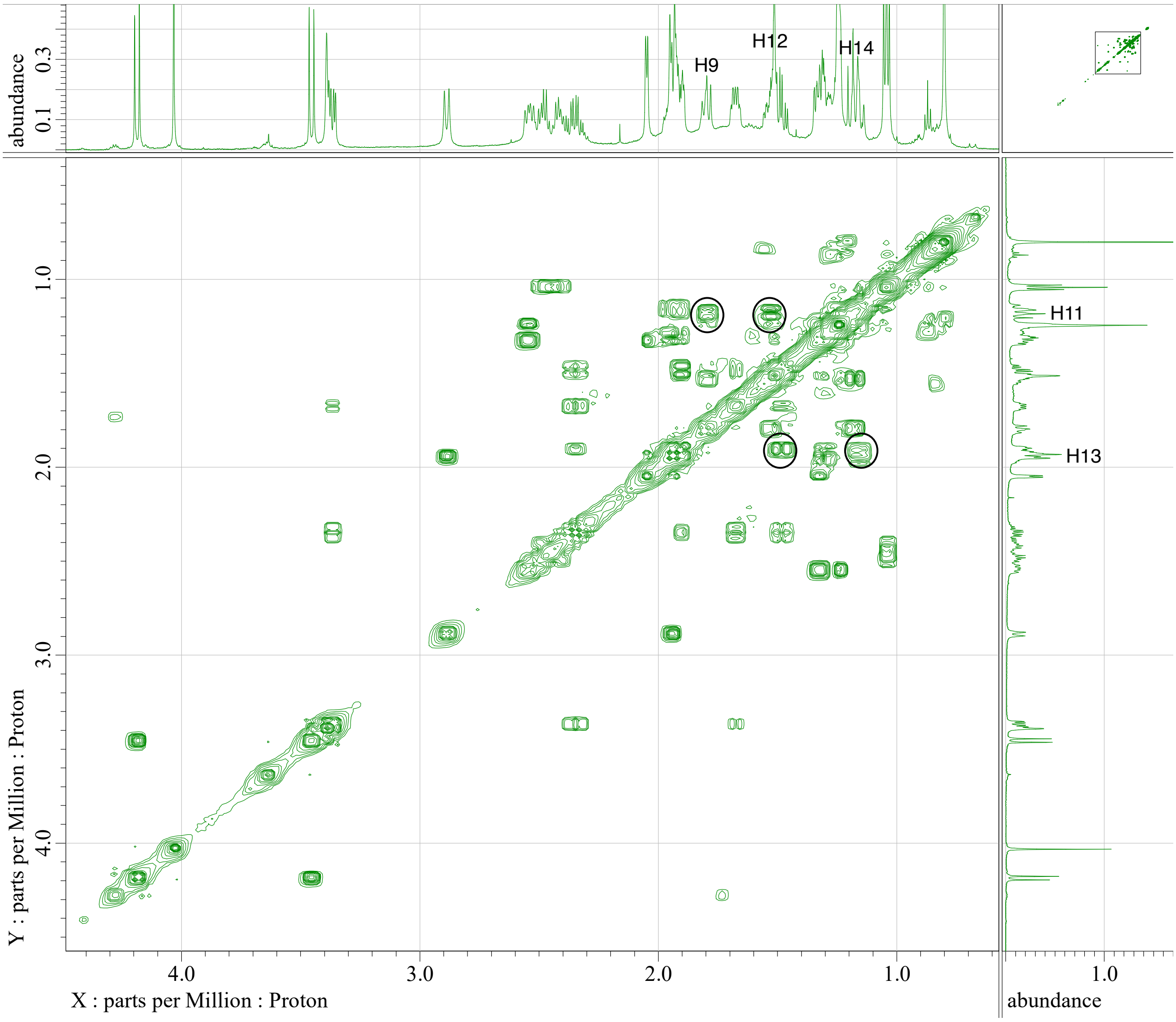

abundance
0.1
0.3
H9
H12
H14
H11
H13
Y : parts per Million : Proton
1.0
2.0
3.0
4.0
X : parts per Million : Proton
abundance

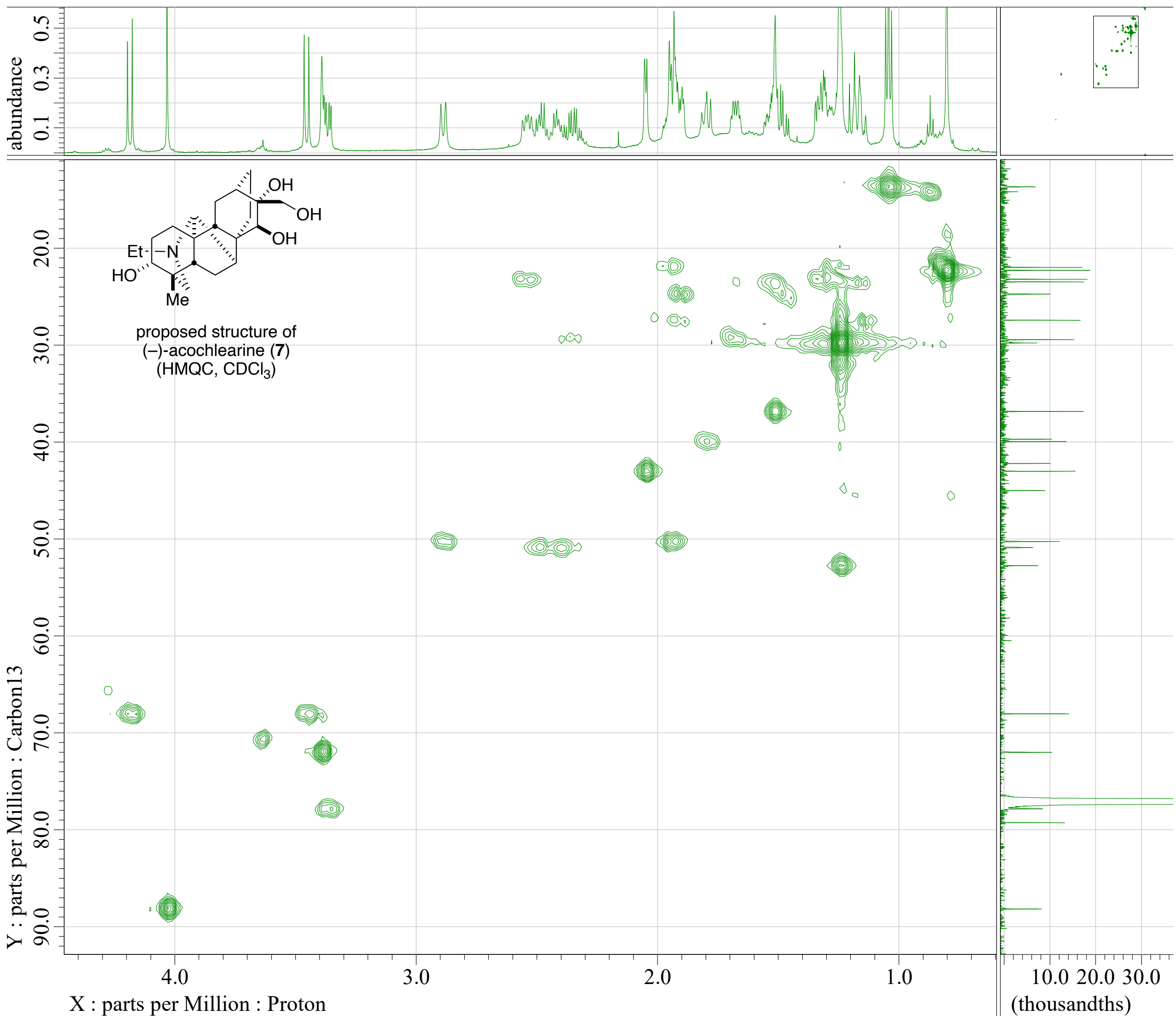

abundance
0.1
0.3
0.5
OH
OH
OH
Et
N
HO
Me
proposed structure of
(–)-acochlearine (7)
(HMQC, CDCl3)
20.0
30.0
40.0
50.0
60.0
70.0
80.0
90.0
Y : parts per Million : Carbon13
4.0
3.0
2.0
1.0
X : parts per Million : Proton
10.0 20.0 30.0
(thousandths)

HMBC ($CDCl_3$)

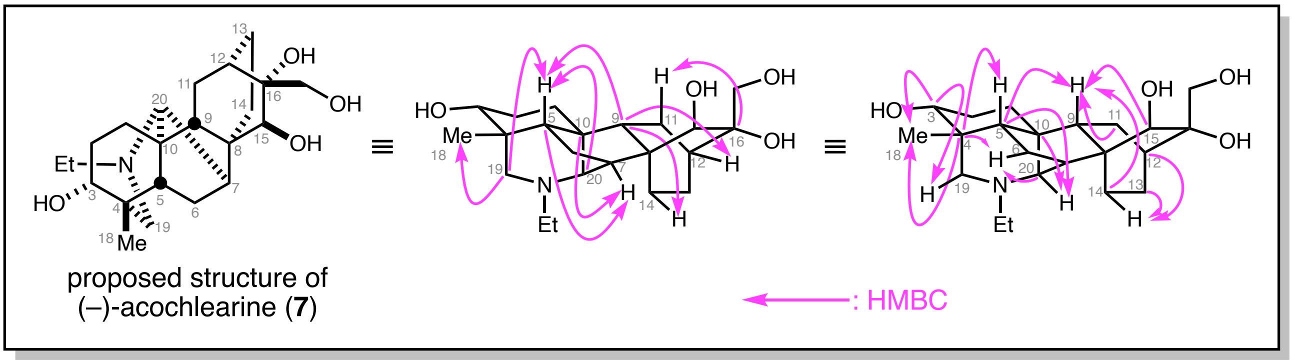

13
12
OH
11
20
16
OH
14
9
Et
N
10
15
OH
8
HO
3
5
7
4
6
18
Me
19
proposed structure of
(–)-acochlearine (7)
HO
Me
18
19
N
Et
5
10
9
H
20
7
H
H
11
OH
OH
16
OH
12
14
H
3
4
H
5
6
20
N
Et
H
10
9
11
OH
OH
15
OH
12
13
14
: HMBC

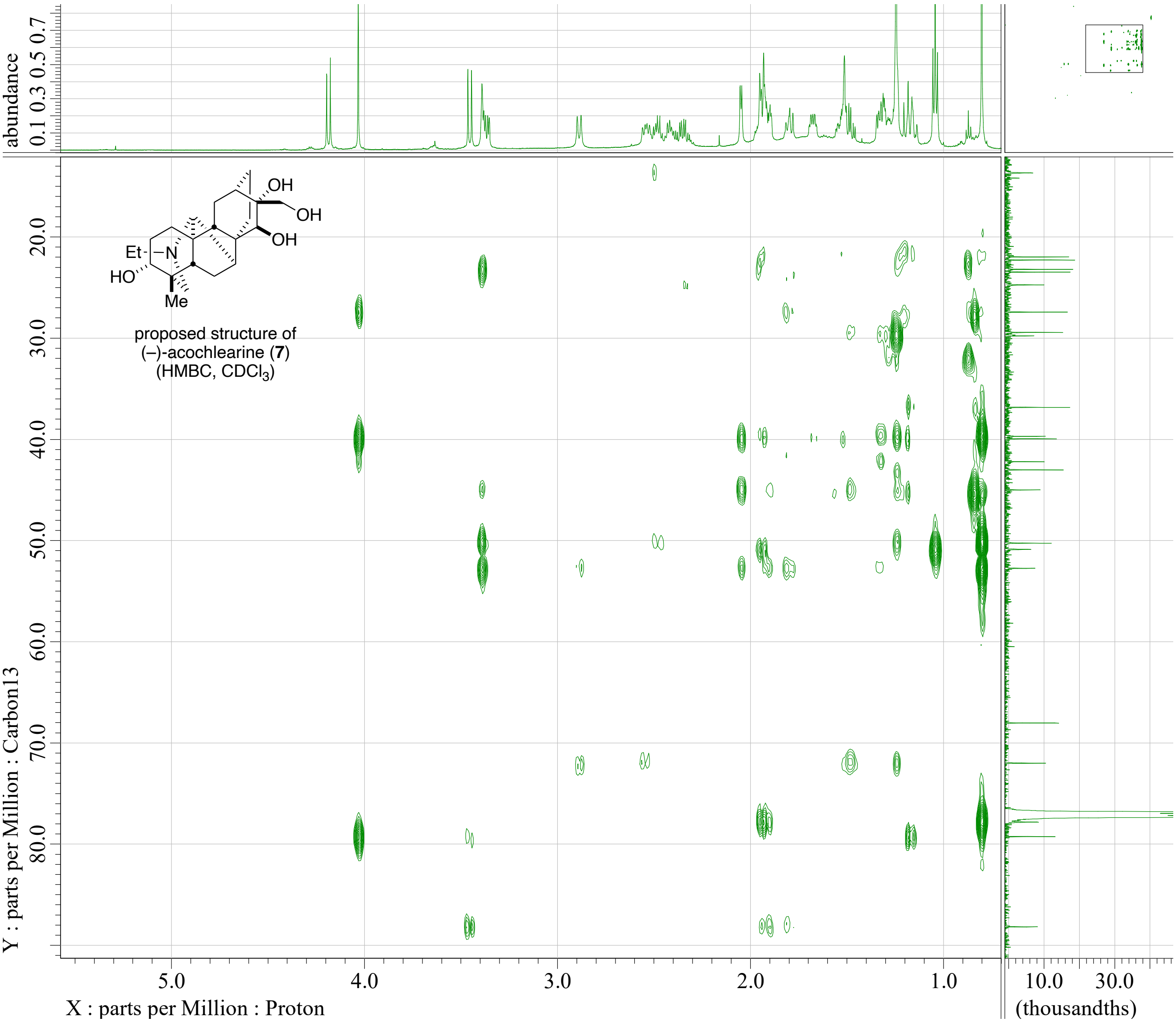

abundance
0.1 0.3 0.5 0.7
OH
Et
N
OH
HO
OH
Me
proposed structure of
(–)-acochlearine (7)
(HMBC, CDCl3)
Y : parts per Million : Carbon13
20.0
30.0
40.0
50.0
60.0
70.0
80.0
5.0
4.0
3.0
2.0
1.0
X : parts per Million : Proton
10.0
30.0
(thousandths)

NOESY ($CDCl_3$)

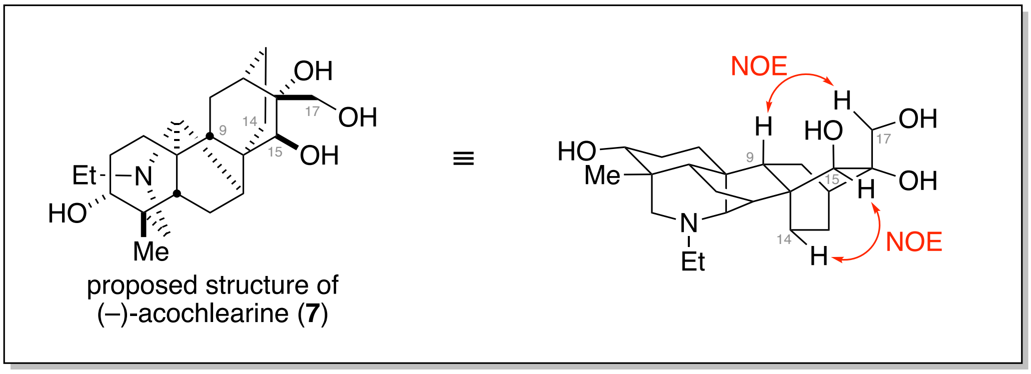

OH
17
OH
9
14
15
OH
Et
N
HO
Me
proposed structure of
(–)-acochlearine (7)
≡
NOE
H
H
HO
OH
HO
Me
9
17
15
H
OH
N
Et
14
H
NOE

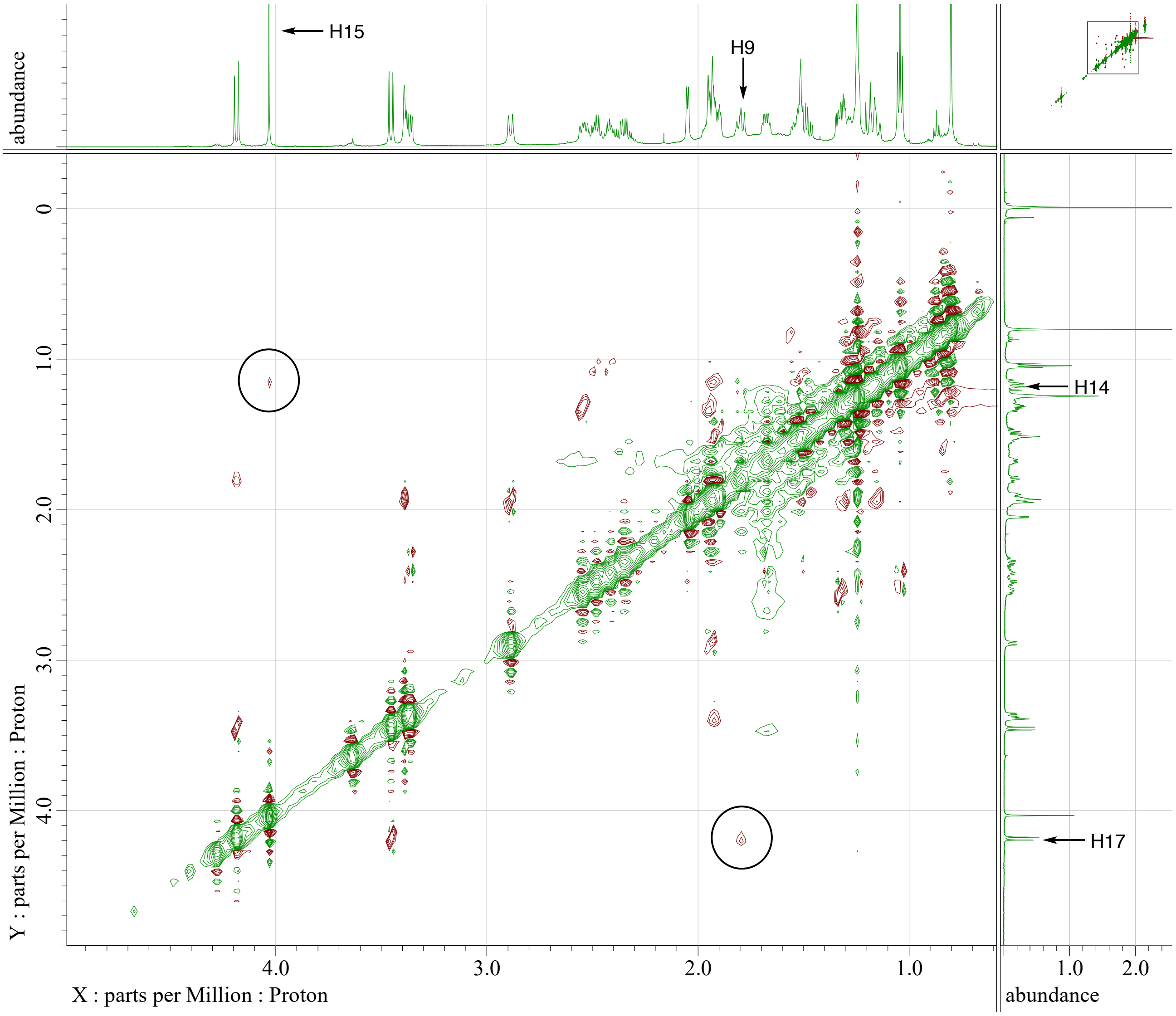

abundance
H15
H9
H14
H17
4.0
3.0
2.0
1.0
0
1.0
2.0
3.0
4.0
Y : parts per Million : Proton
X : parts per Million : Proton
1.0
2.0
abundance

^{1}H-NMR (600 MHz, $CDCl_3$)

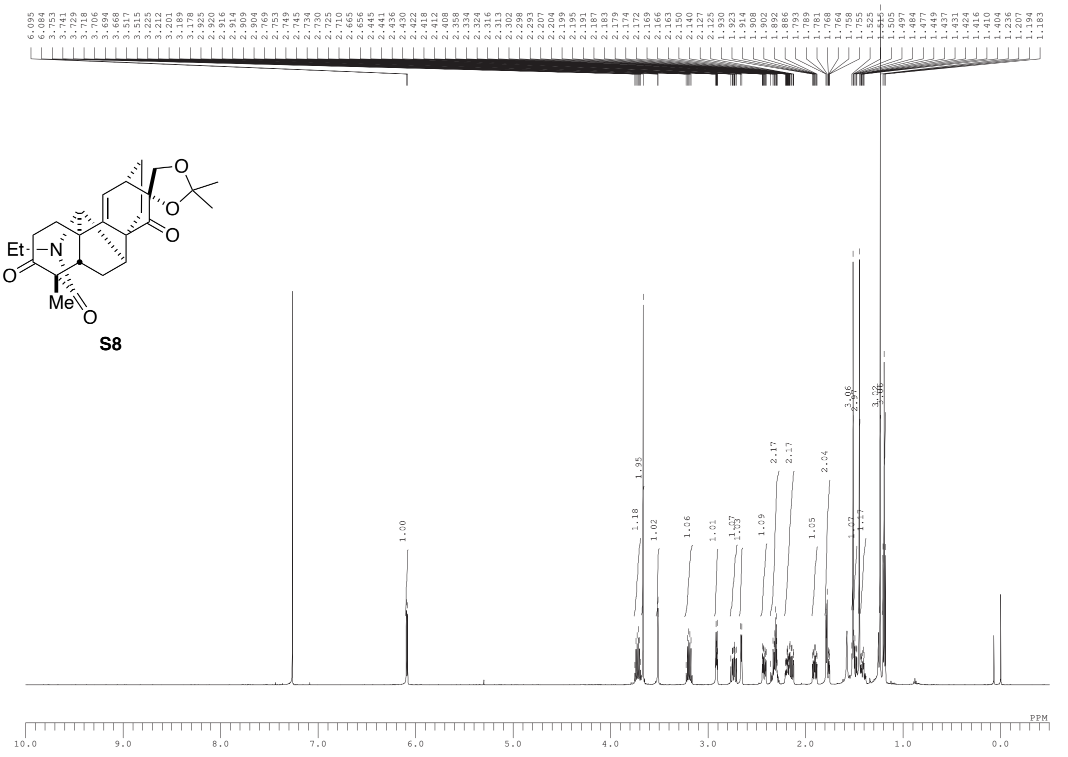

^{13}C-NMR (150 MHz, $CDCl_3$)

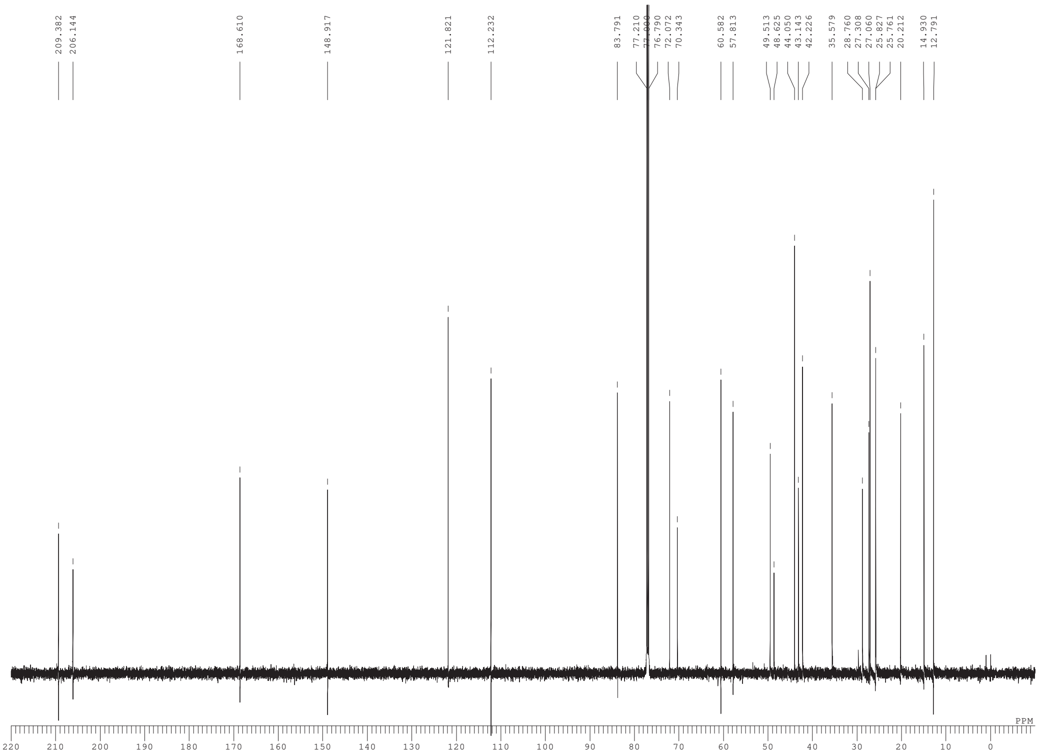

^{1}H-NMR (600 MHz, $CDCl_3$)

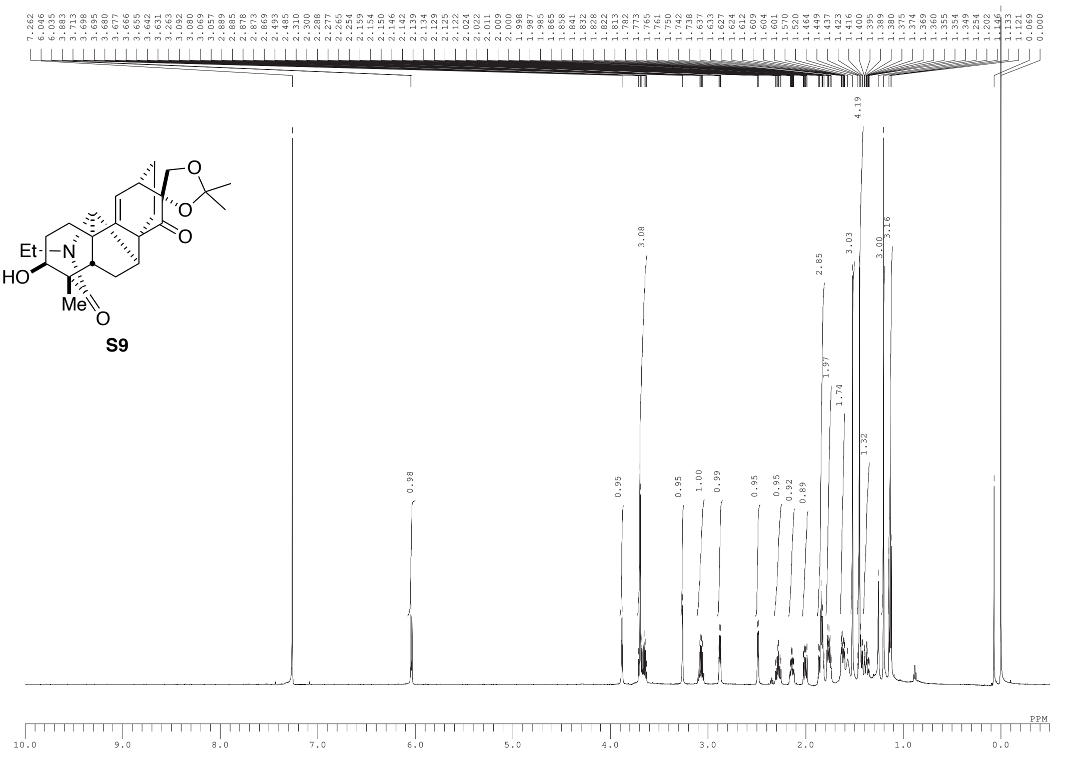

^{13}C-NMR (150 MHz, $CDCl_3$)

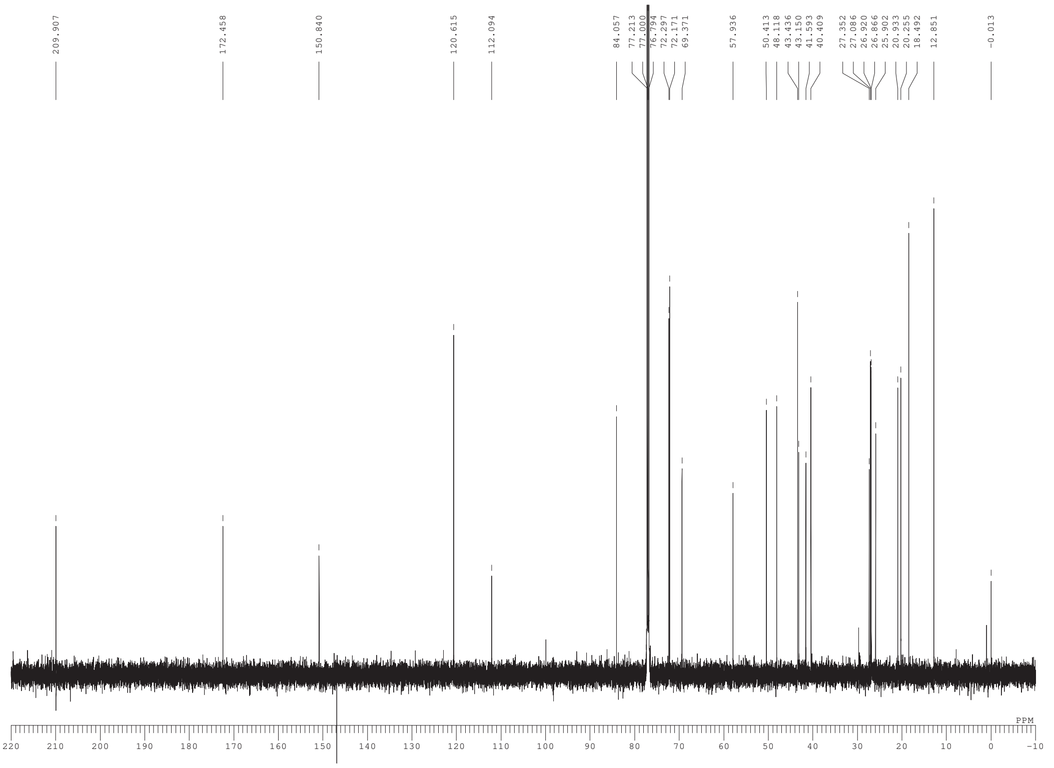

^{1}H-NMR (600 MHz, $CDCl_3$)

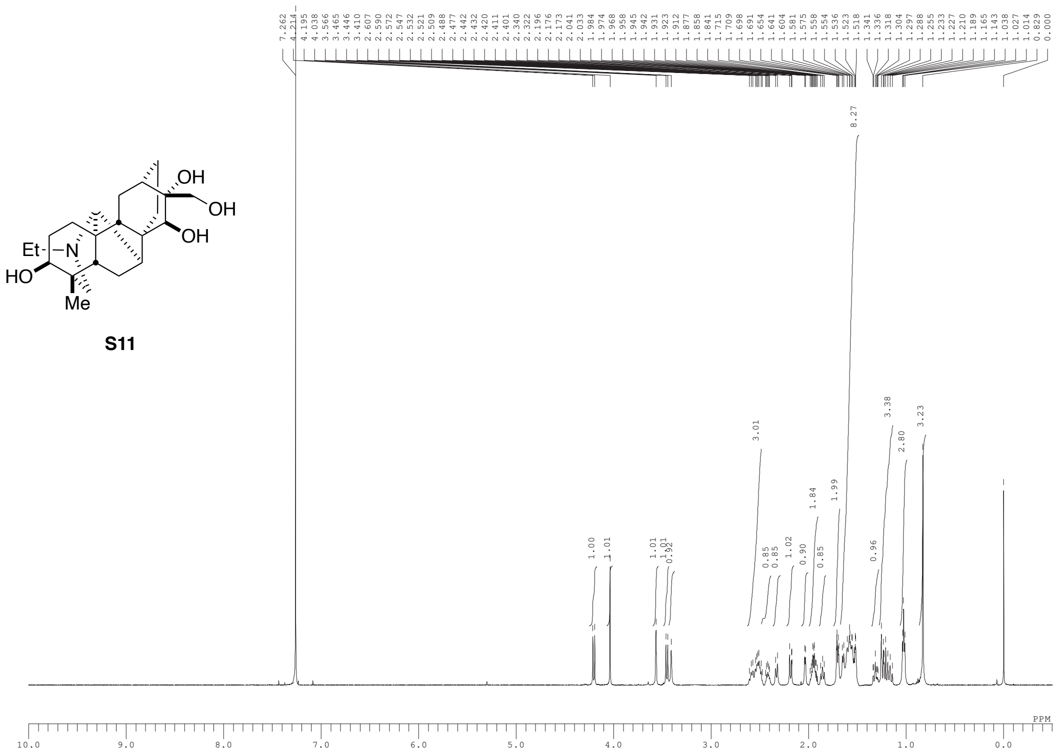

^{13}C-NMR (150 MHz, $CDCl_3$)

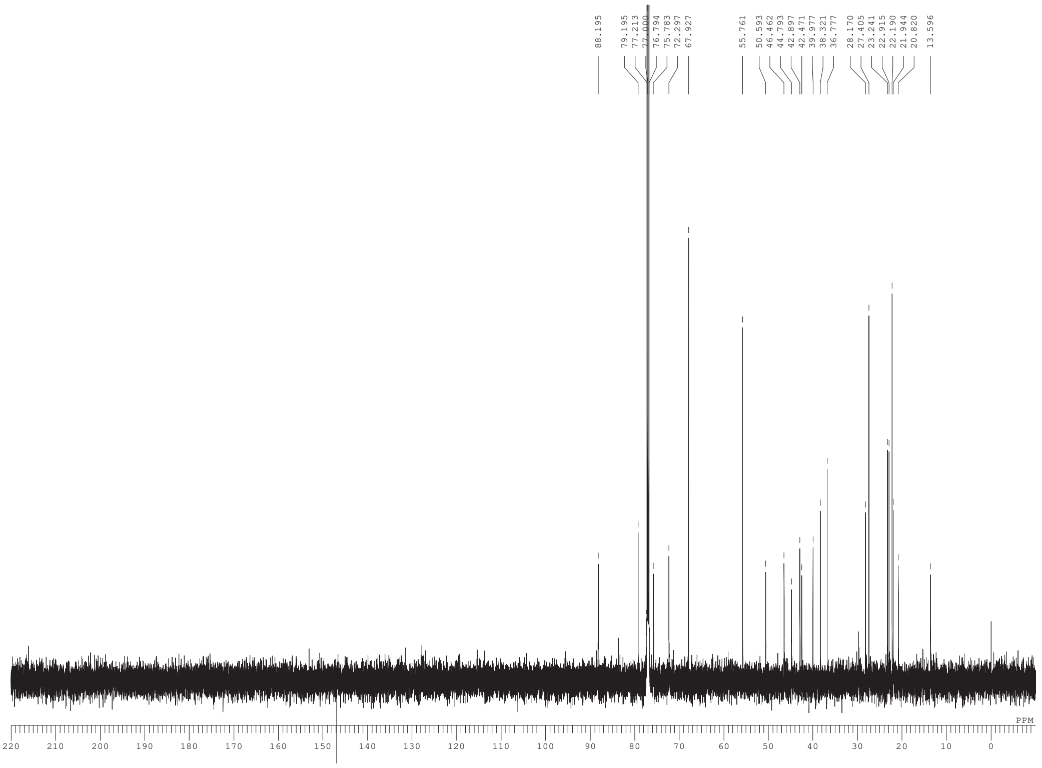

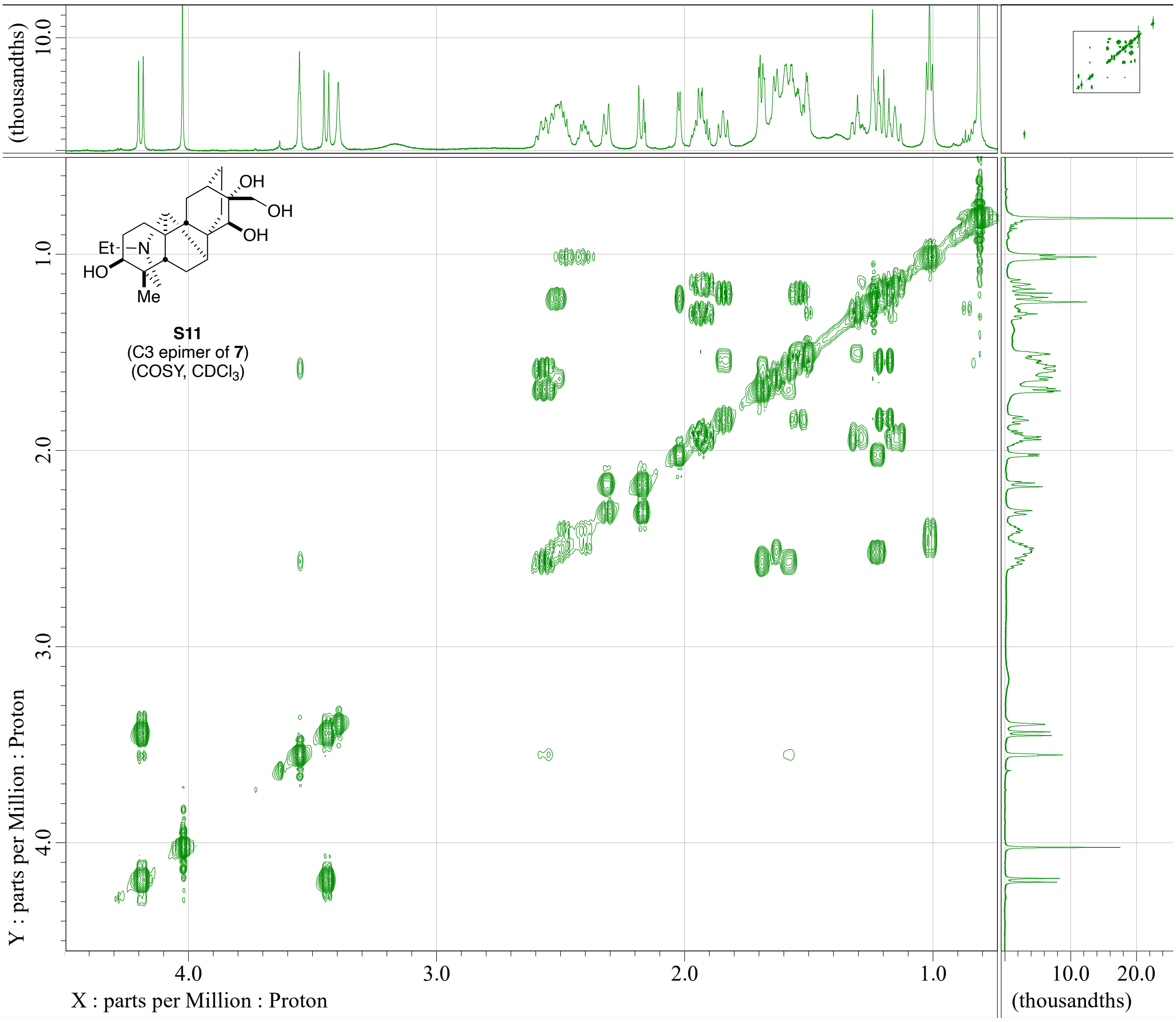

OH
OH
OH
Et
N
HO
Me
S11
(C3 epimer of 7)
(COSY, CDCl3)
(thousandths)
10.0
1.0
2.0
3.0
4.0
Y : parts per Million : Proton
X : parts per Million : Proton
20.0

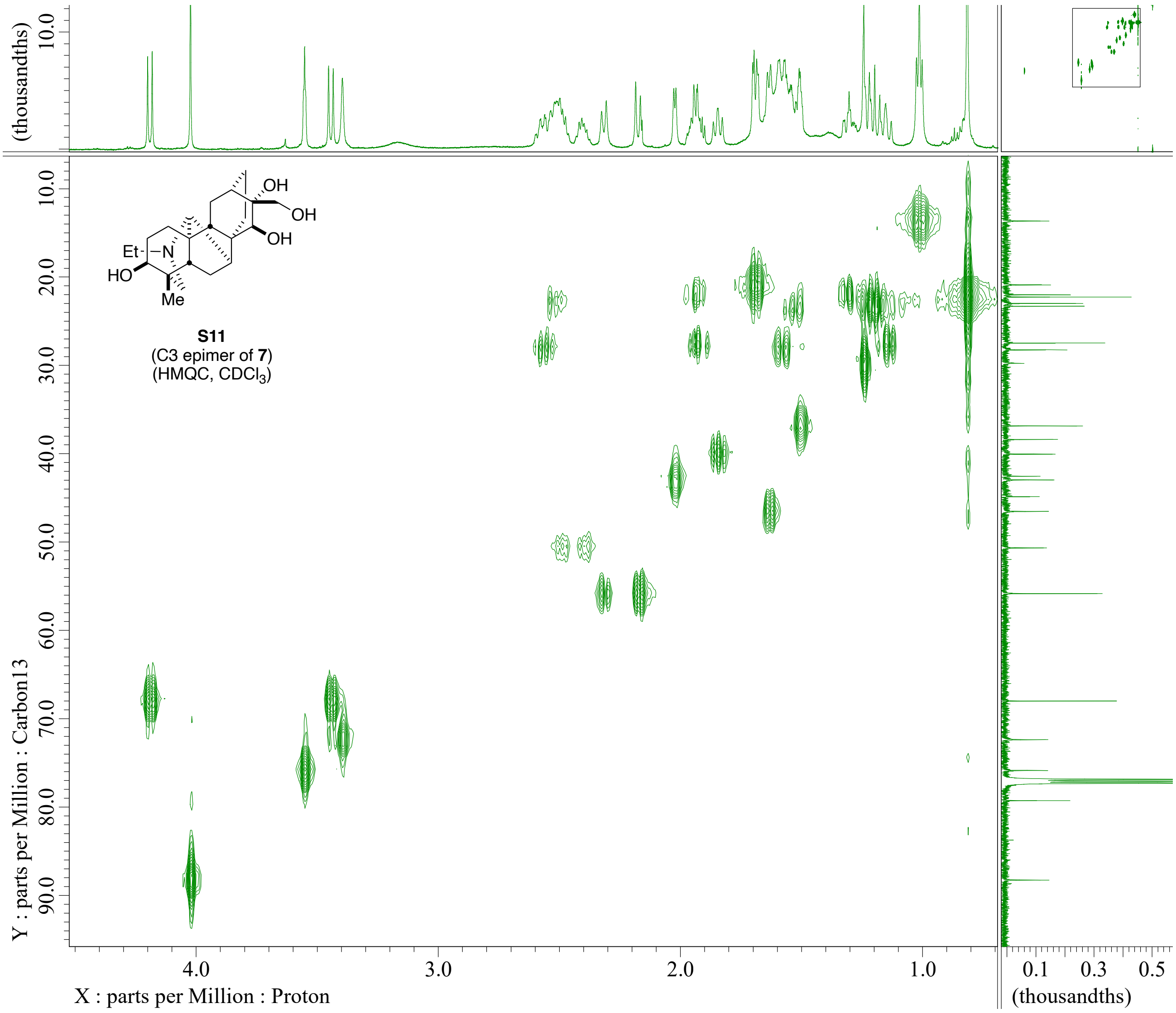

(thousandths)
10.0
OH
OH
Et
N
OH
HO
Me
S11
(C3 epimer of 7)
(HMQC, CDCl3)
Y : parts per Million : Carbon13
10.0
20.0
30.0
40.0
50.0
60.0
70.0
80.0
90.0
4.0
3.0
2.0
1.0
X : parts per Million : Proton
0.1
0.3
0.5
(thousandths)

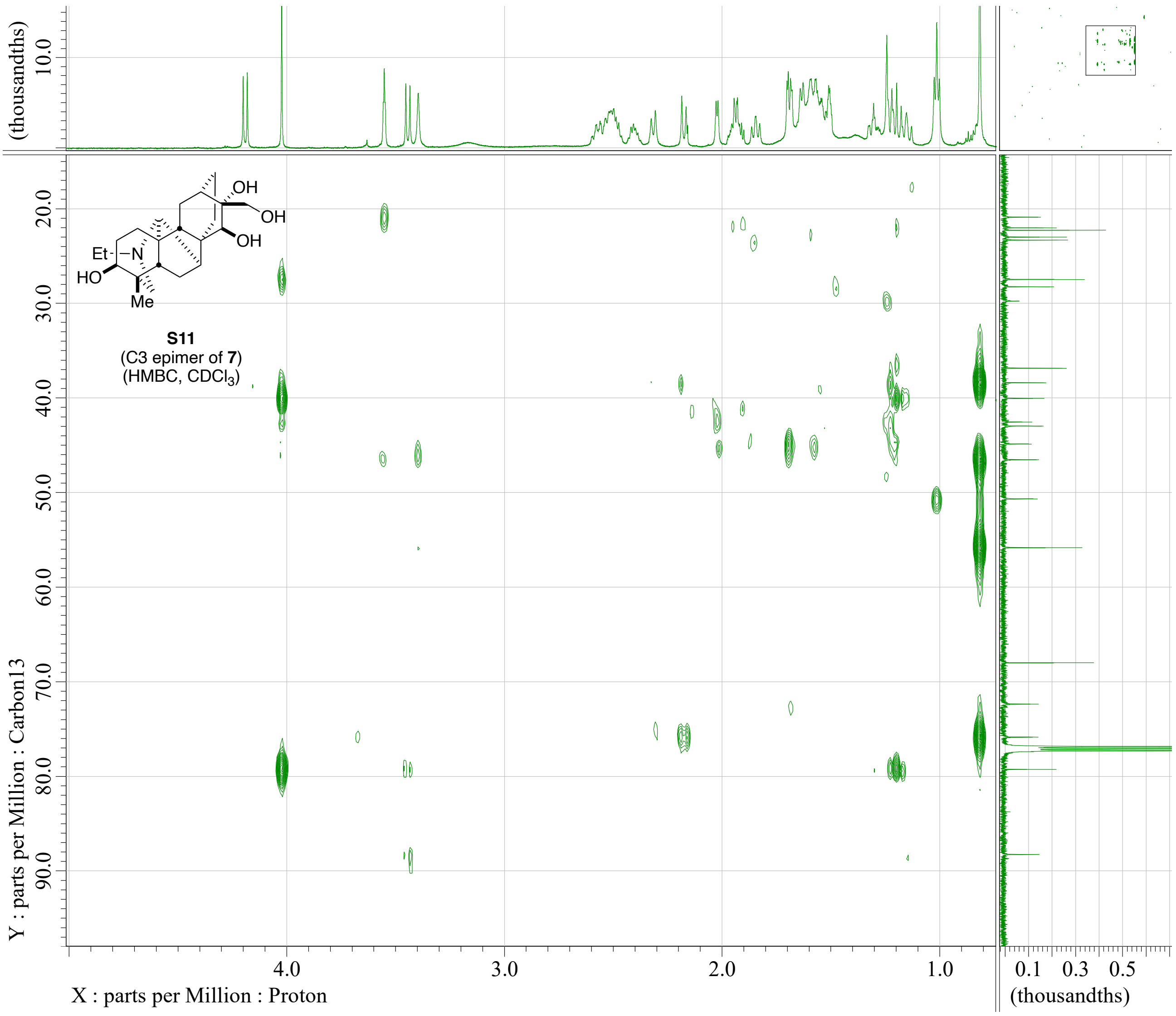
OH
OH
OH
Et
N
HO
Me
S11
(C3 epimer of 7)
(HMBC, CDCl3)
(thousandths)
10.0
Y : parts per Million : Carbon13
X : parts per Million : Proton
4.0
3.0
2.0
1.0
0.1
0.3
0.5

^{1}H-NMR (600 MHz, $CDCl_3$)

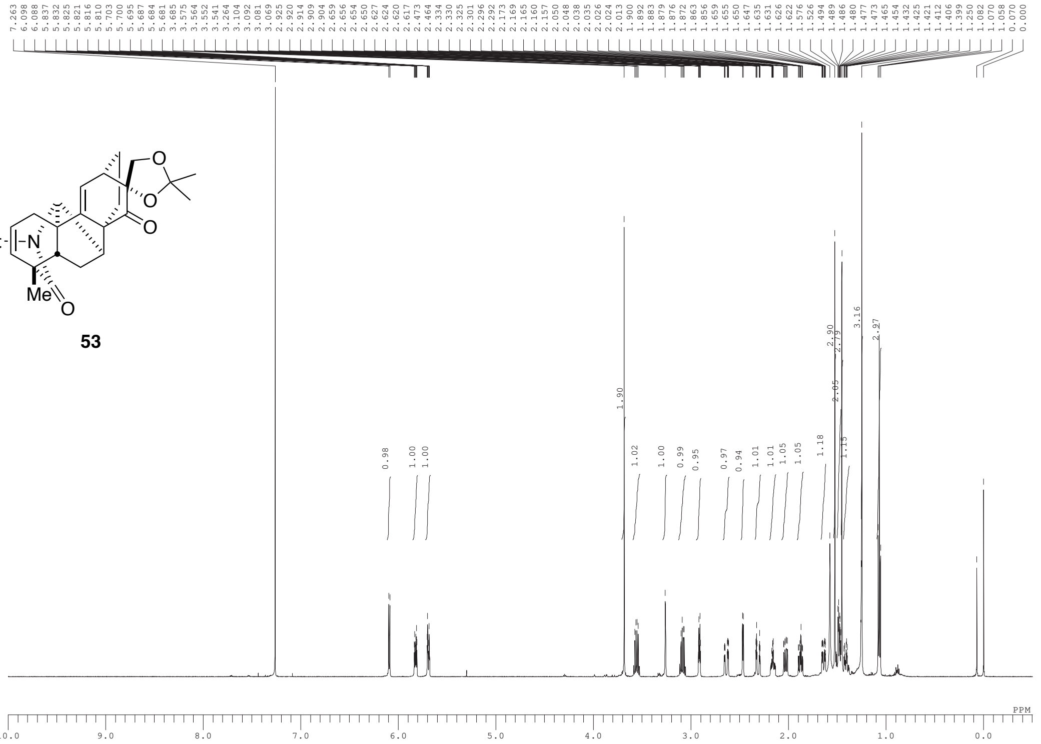

^{13}C-NMR (150 MHz, $CDCl_3$)

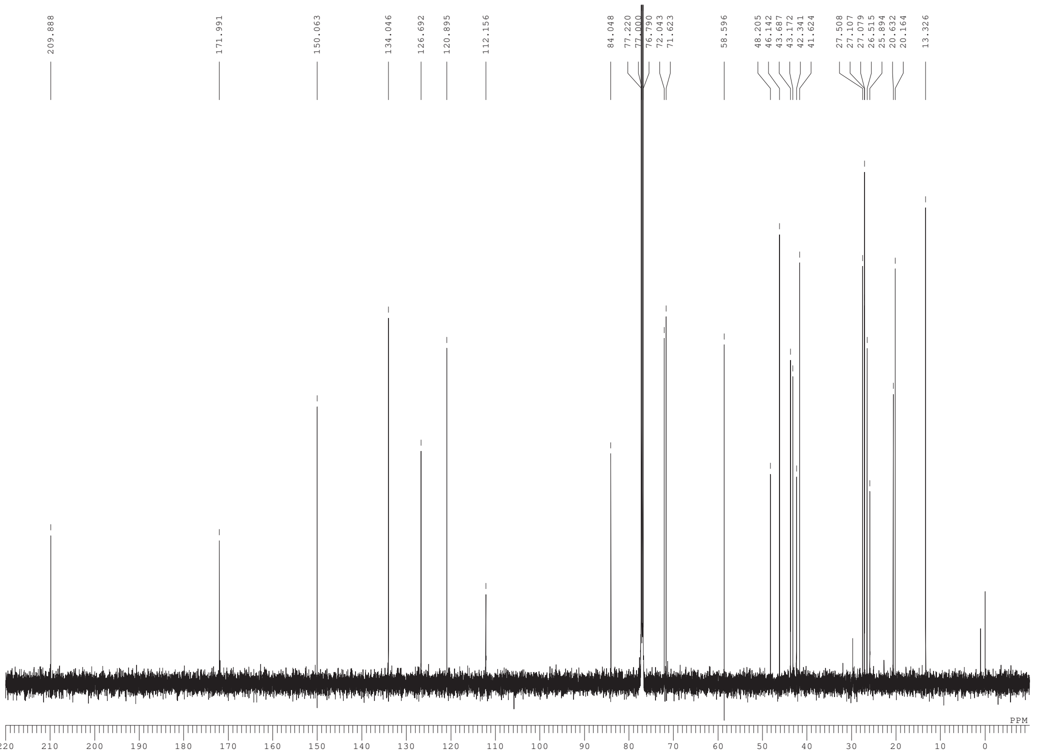

^{1}H-NMR (600 MHz, $CDCl_3$)

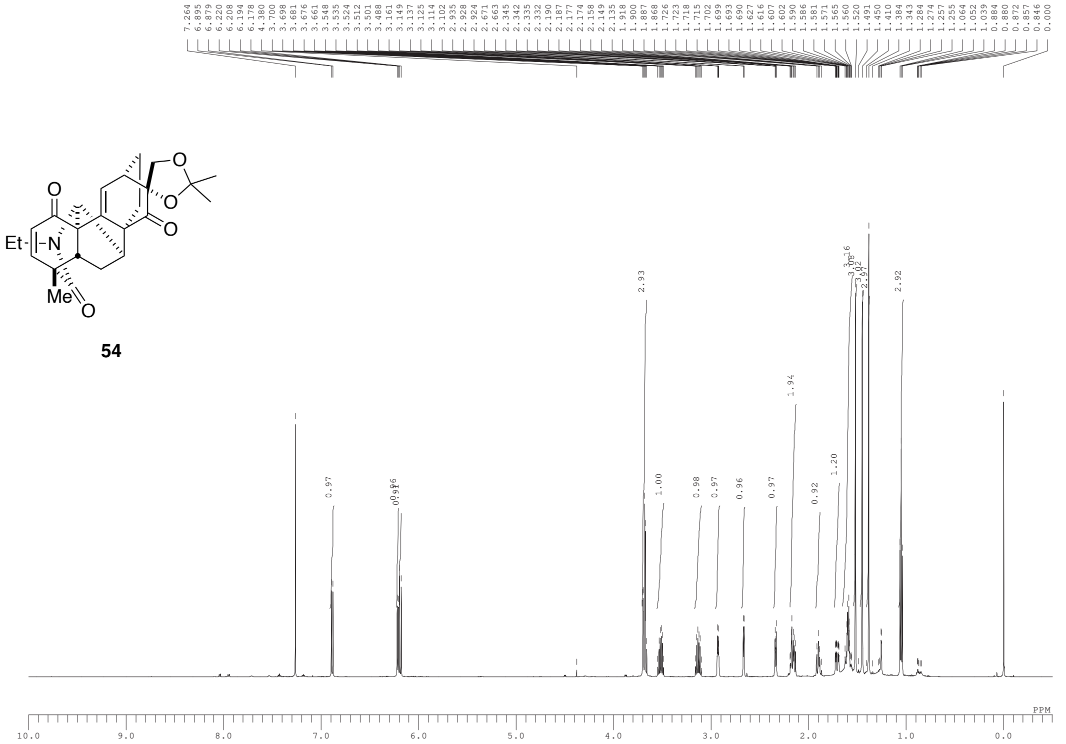

54

^{13}C-NMR (150 MHz, $CDCl_3$)

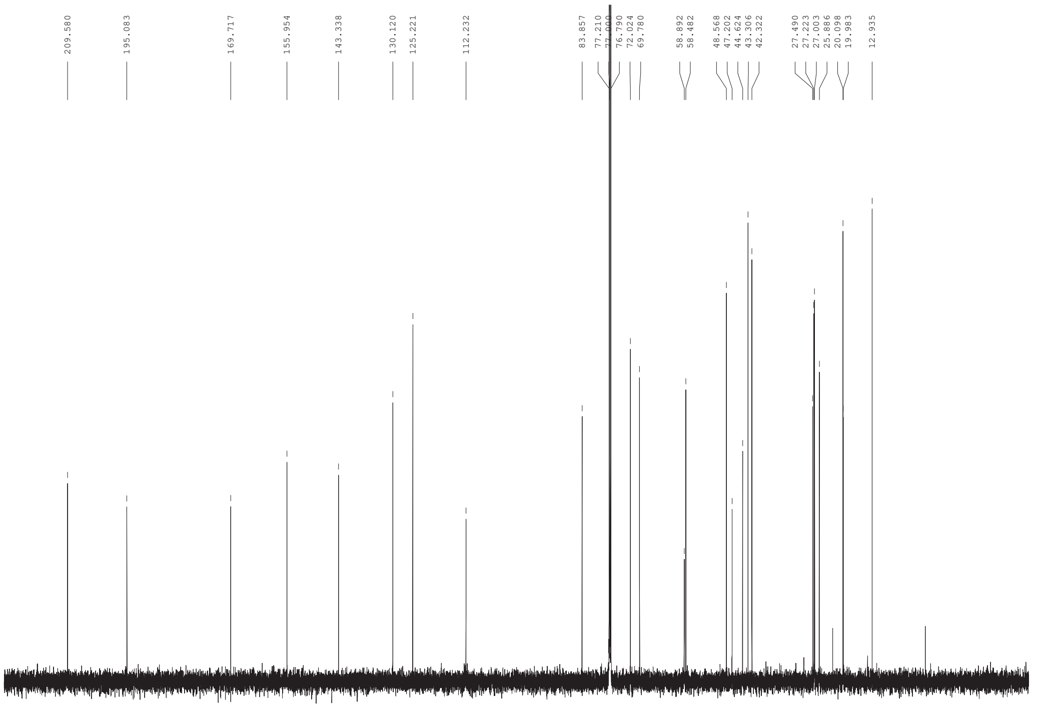

^{1}H-NMR (600 MHz, CDCl$_3$)

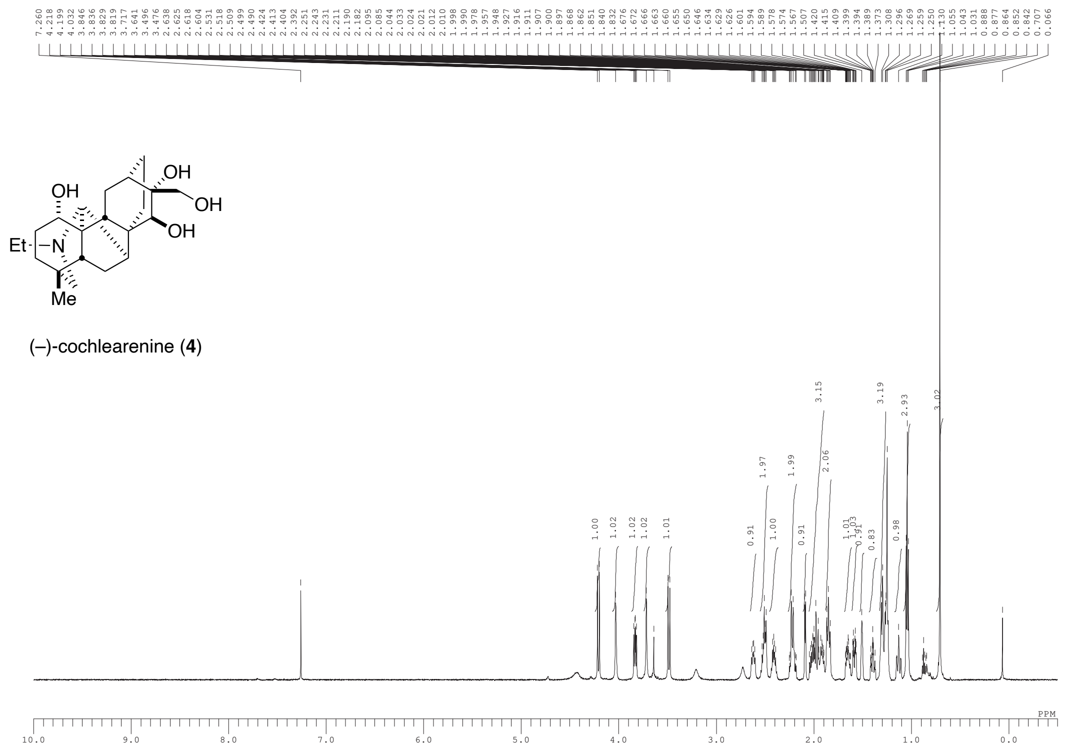

^{13}C-NMR (150 MHz, CDCl$_3$)

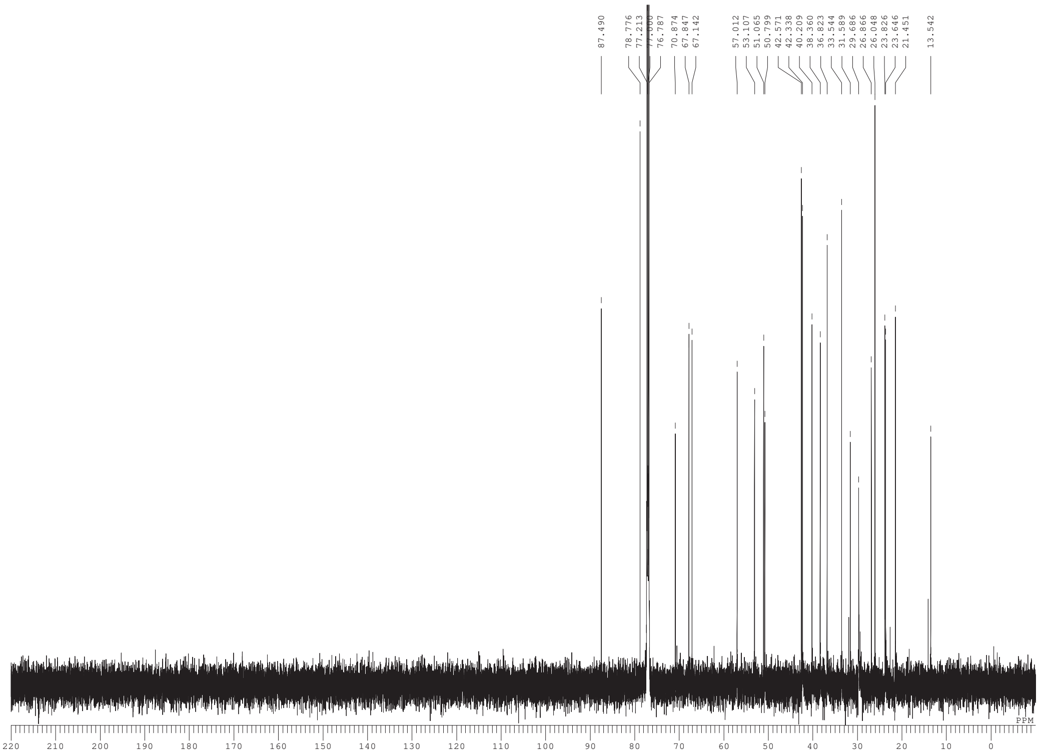

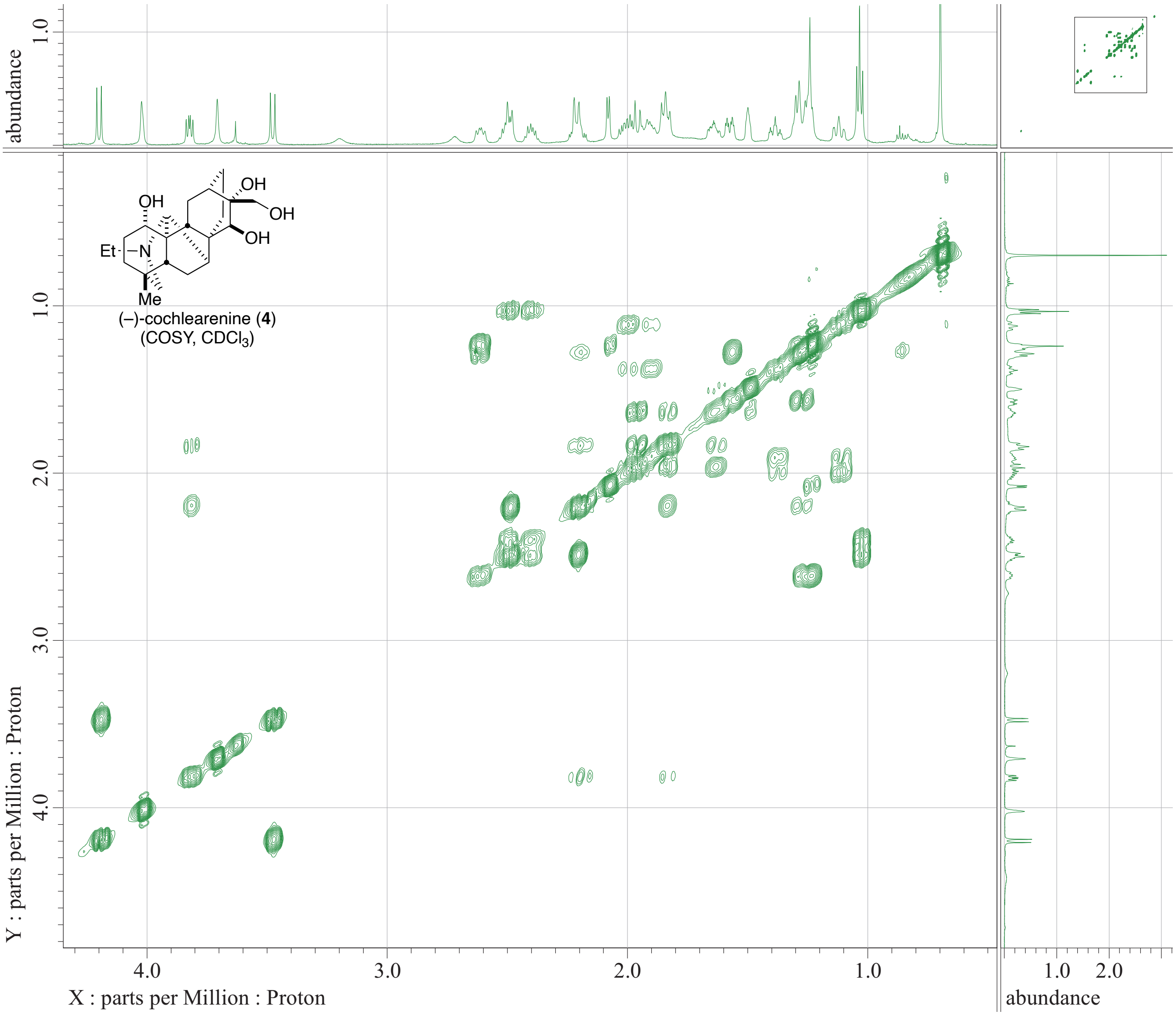

OH
OH
OH
OH
Et
N
Me
(–)-cochlearenine (4)
(COSY, CDCl3)
abundance
1.0
Y : parts per Million : Proton
X : parts per Million : Proton
4.0
3.0
2.0
1.0
abundance

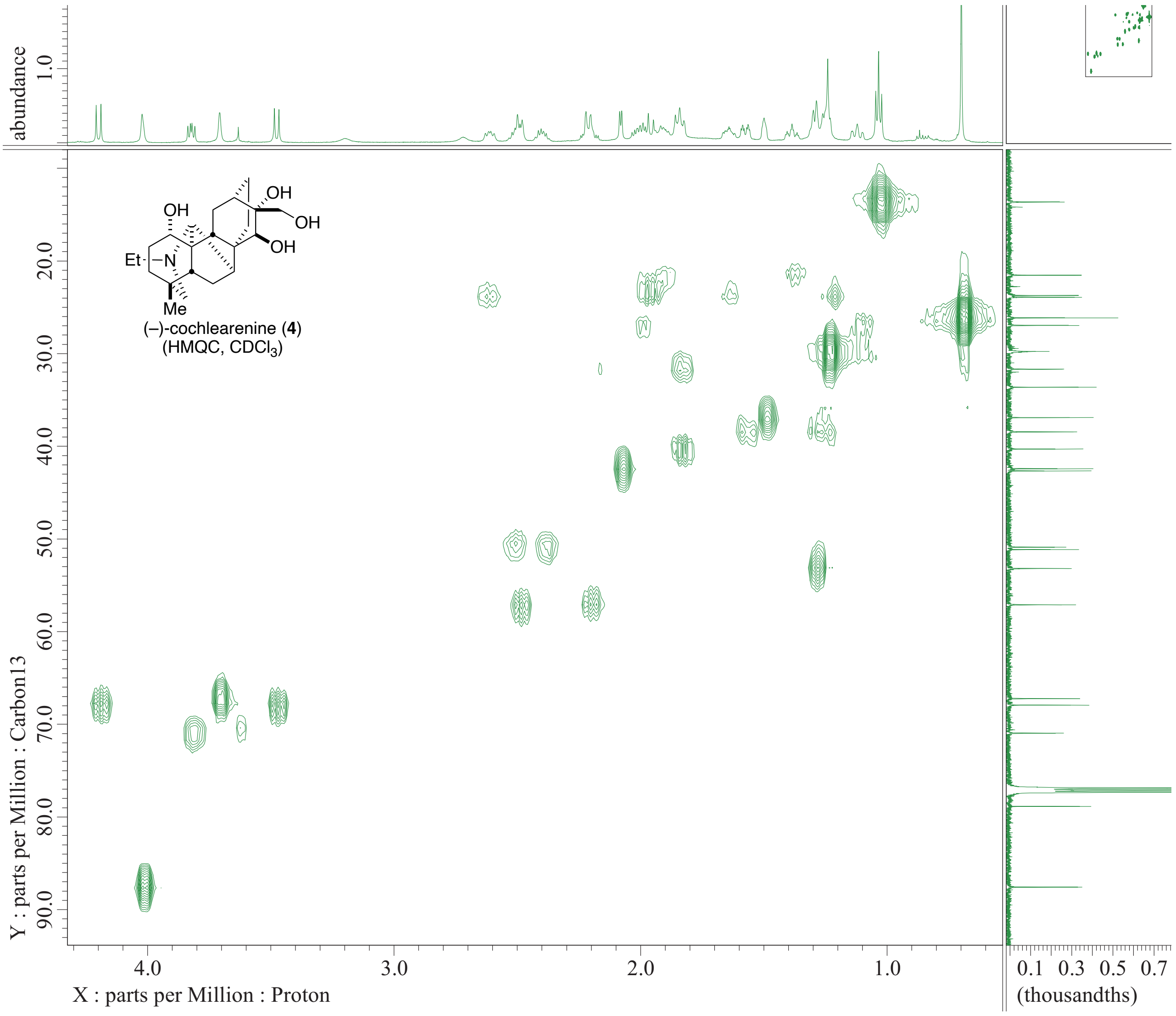
OH
OH
OH
OH
Et
N
Me
(–)-cochlearenine (4)
(HMQC, CDCl3)
abundance
1.0
Y : parts per Million : Carbon13
X : parts per Million : Proton
(thousandths)

^{1}H-NMR (600 MHz, $CDCl_3$)

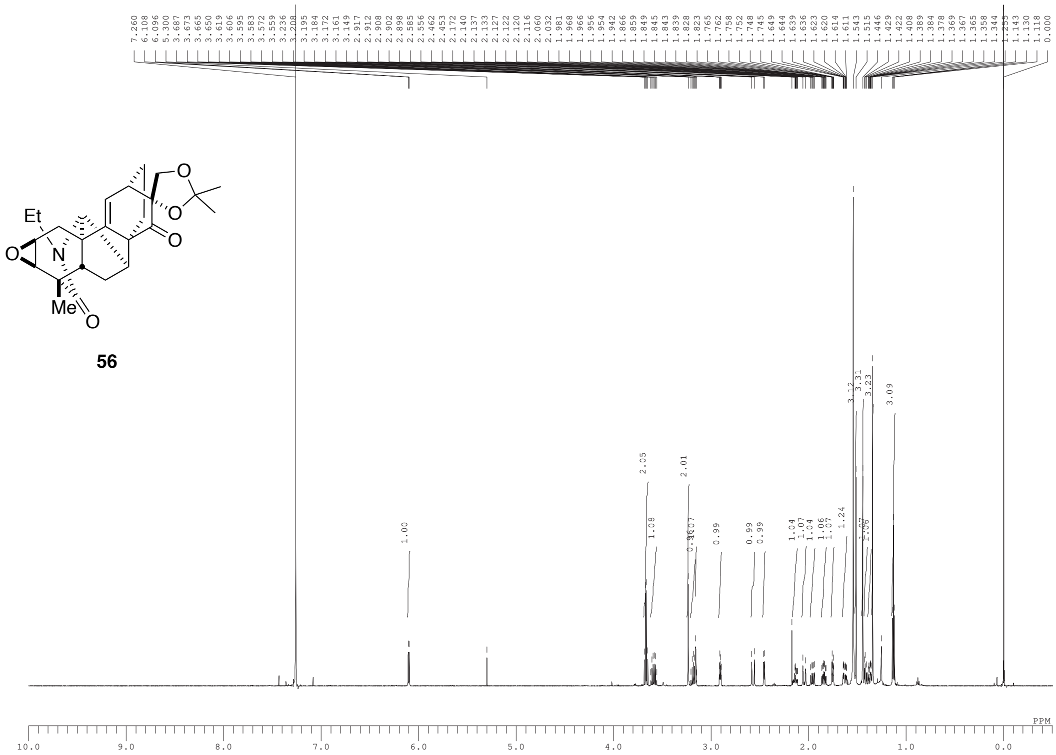

^{13}C-NMR (150 MHz, $CDCl_3$)

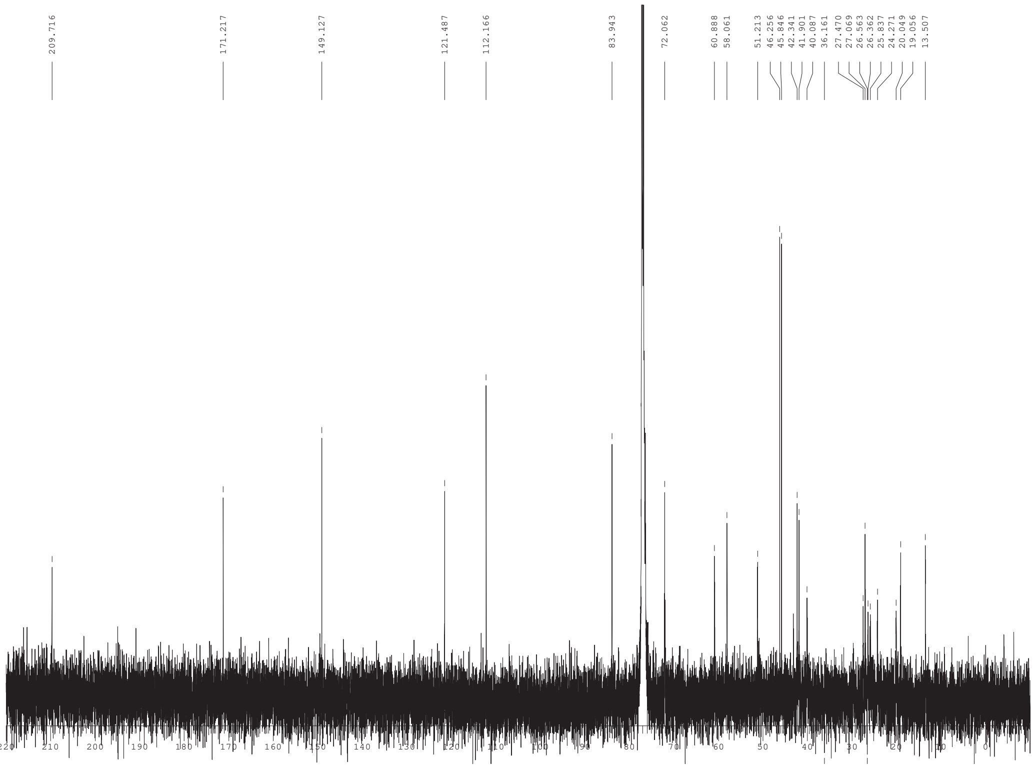

^{1}H-NMR (600 MHz, $CDCl_3$)

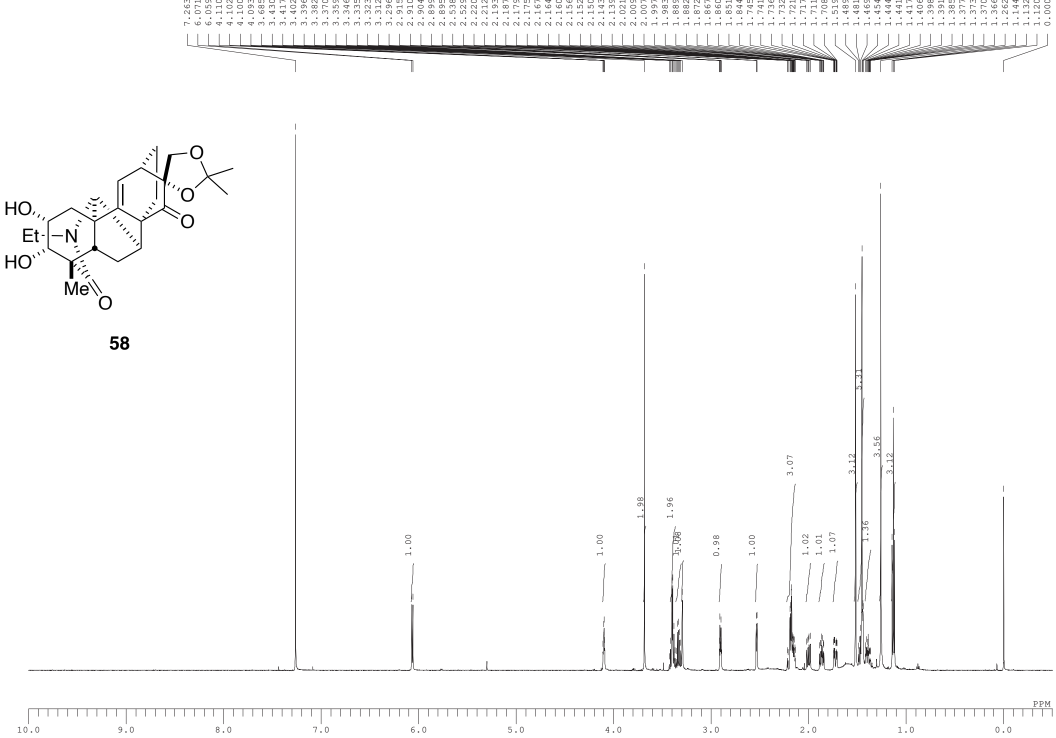

58

^{13}C-NMR (150 MHz, $CDCl_3$)

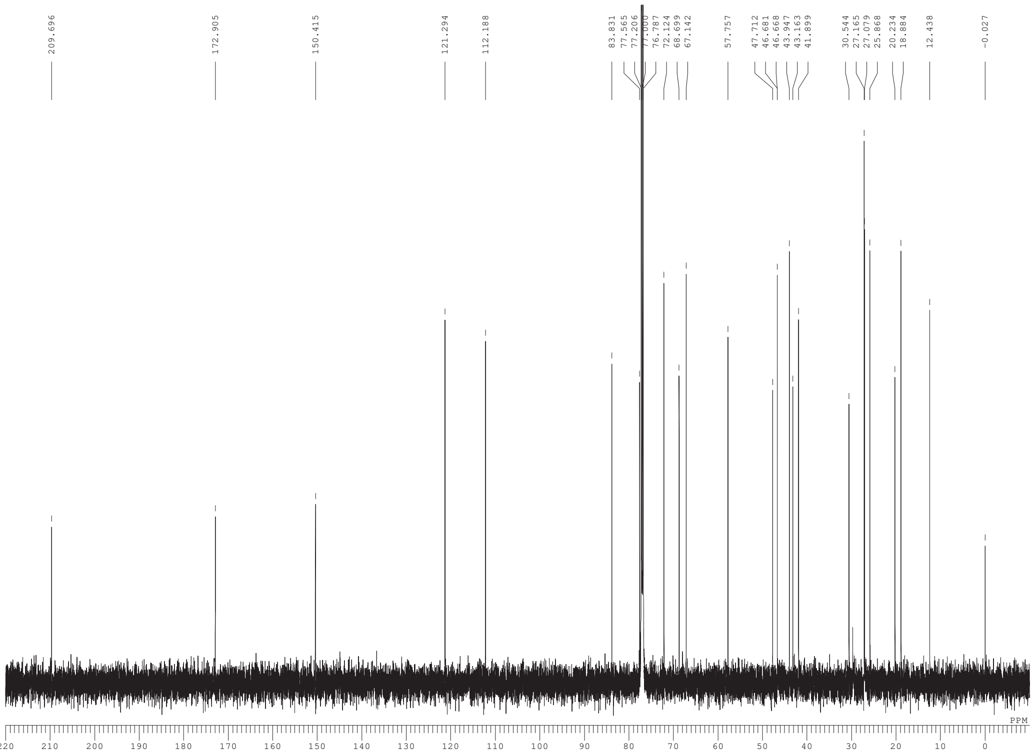

^{1}H-NMR (600 MHz, CD_3OD)

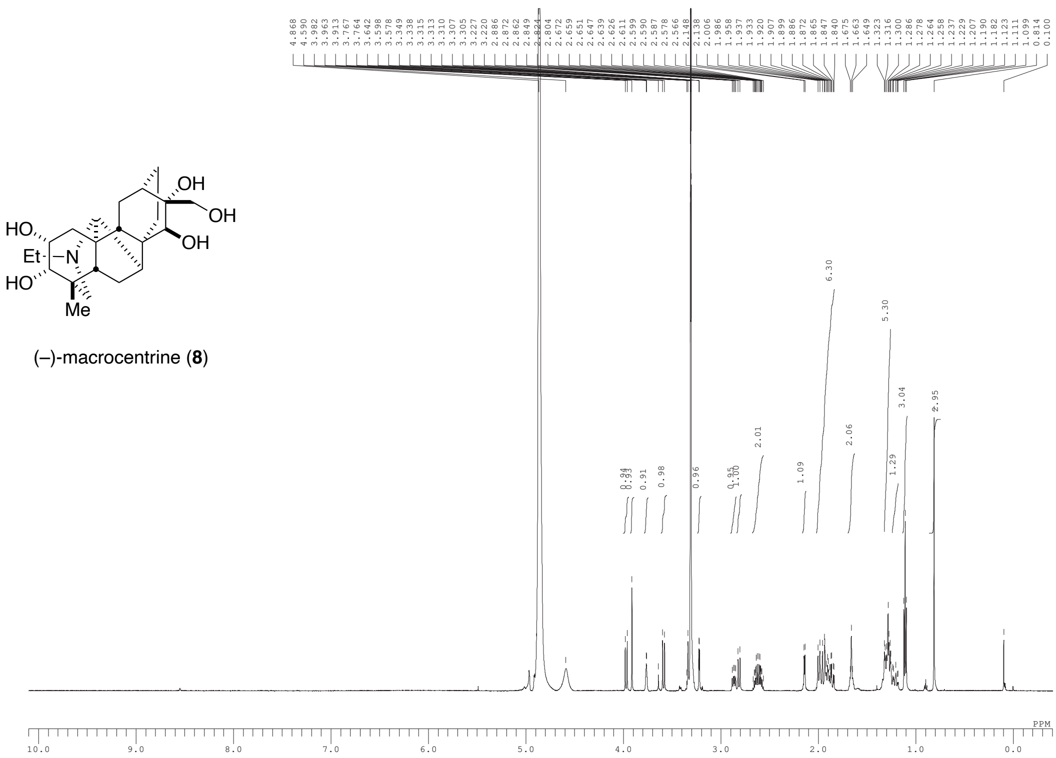

(–)-macrocentrine (**8**)

^{13}C-NMR (150 MHz, CD_3OD)

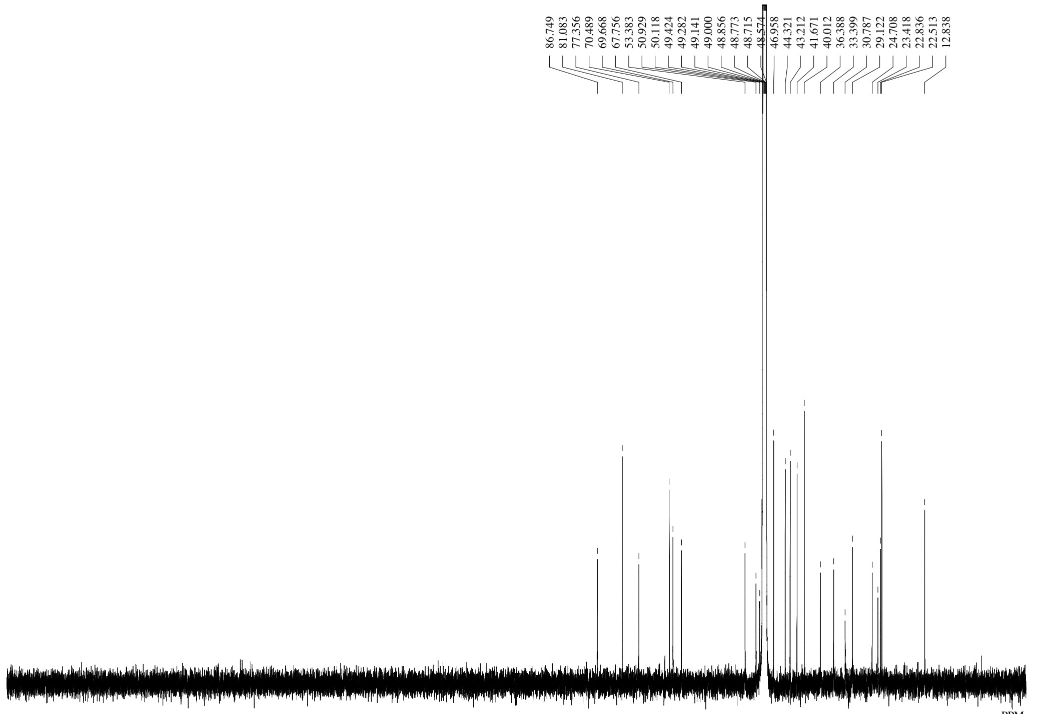

^{13}C-NMR (150 MHz, C_5D_5N)

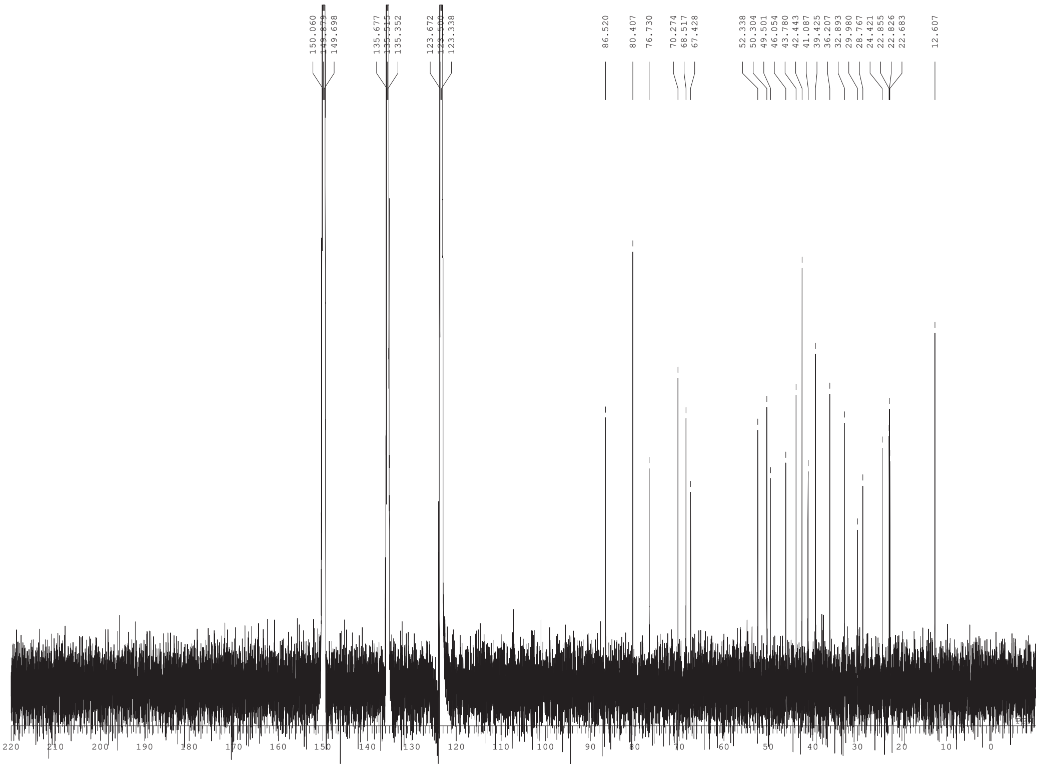

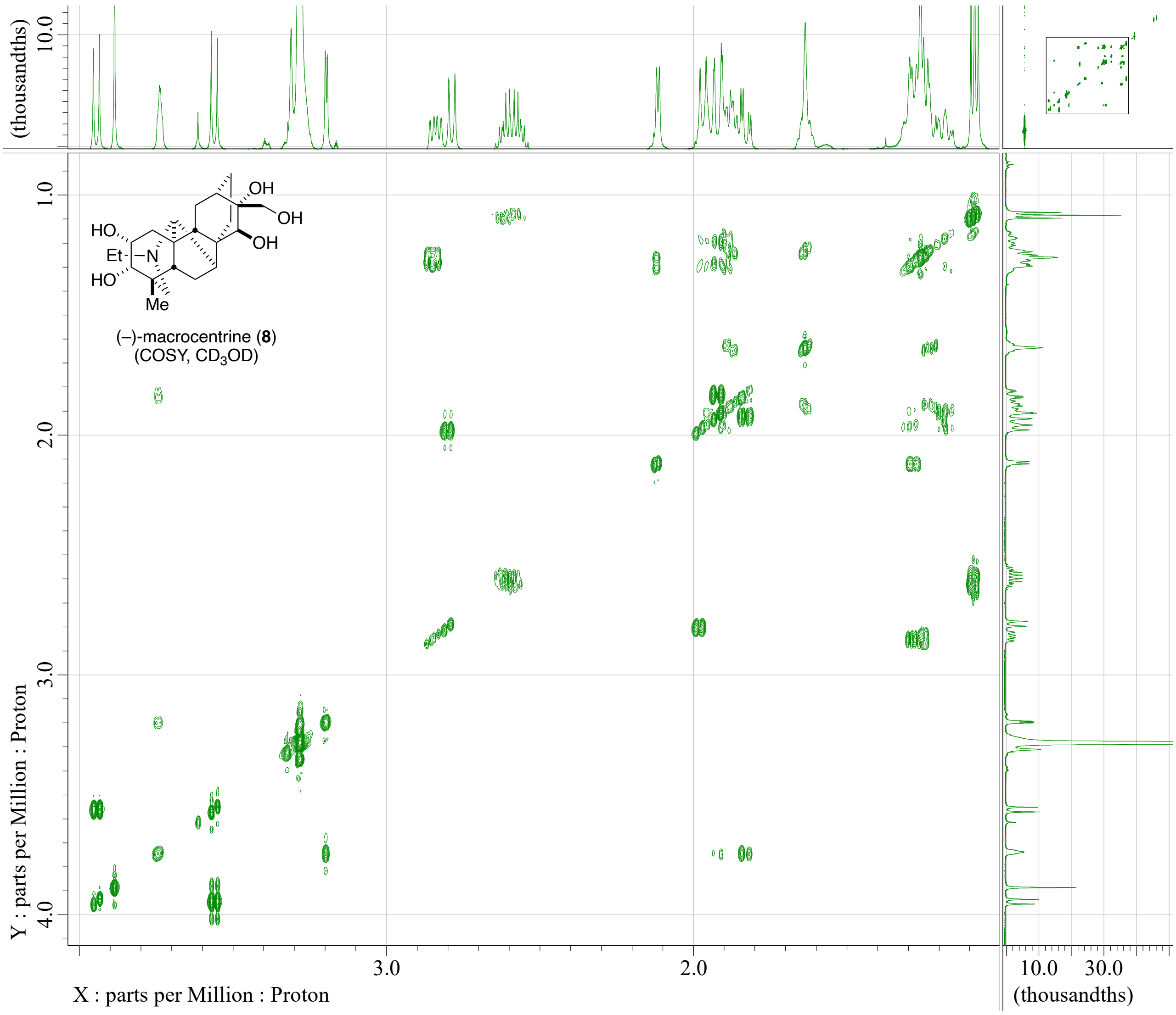

(thousandths)
10.0
HO
Et
N
HO
Me
OH
OH
OH
(–)-macrocentrine (8)
(COSY, CD3OD)
1.0
2.0
3.0
4.0
Y : parts per Million : Proton
3.0
2.0
X : parts per Million : Proton
10.0
30.0
(thousandths)

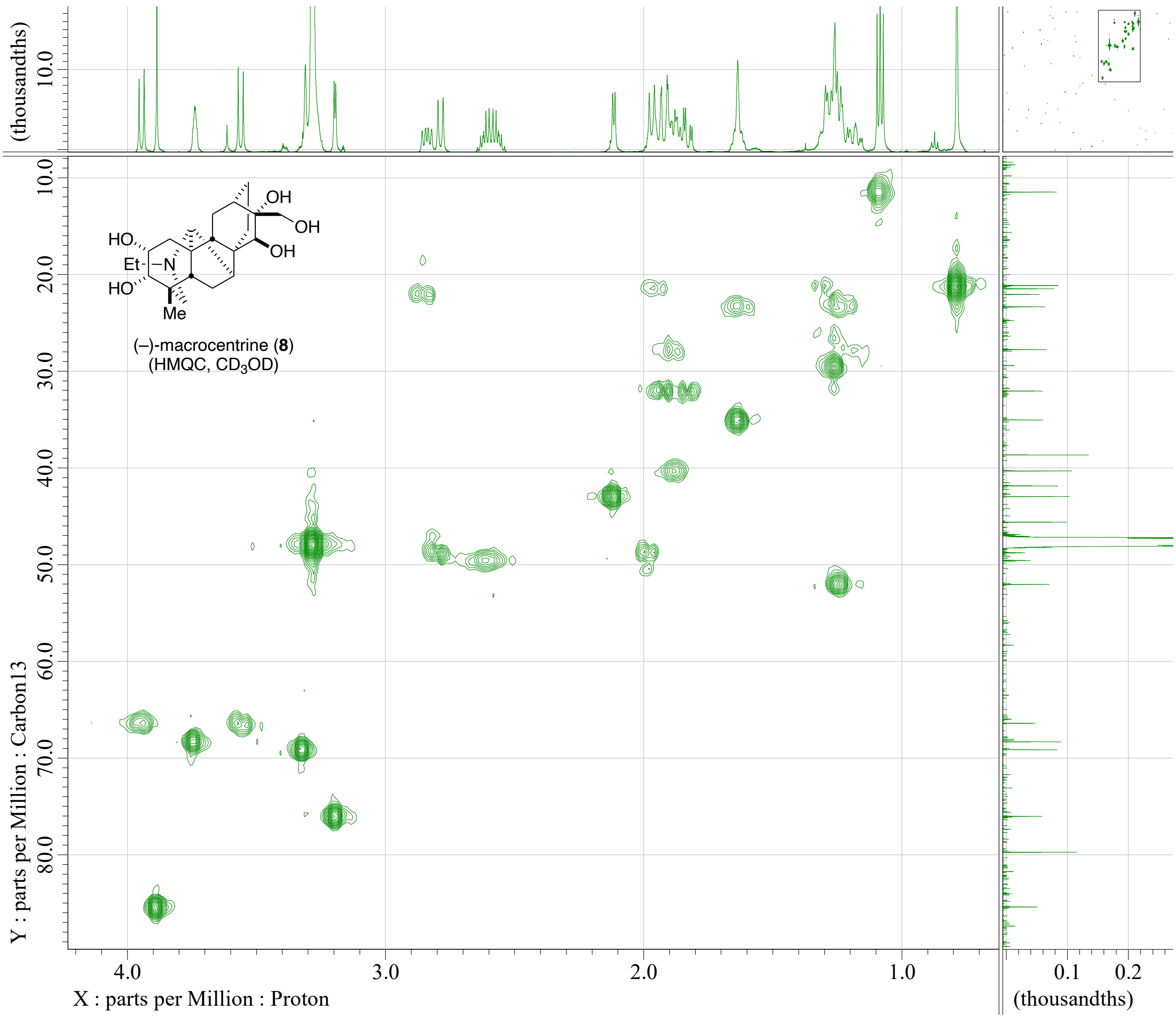

(thousandths)
10.0
HO
Et
N
HO
Me
OH
OH
OH
(–)-macrocentrine (8)
(HMQC, CD3OD)
Y : parts per Million : Carbon13
10.0
20.0
30.0
40.0
50.0
60.0
70.0
80.0
4.0
3.0
2.0
1.0
X : parts per Million : Proton
0.1
0.2
(thousandths)

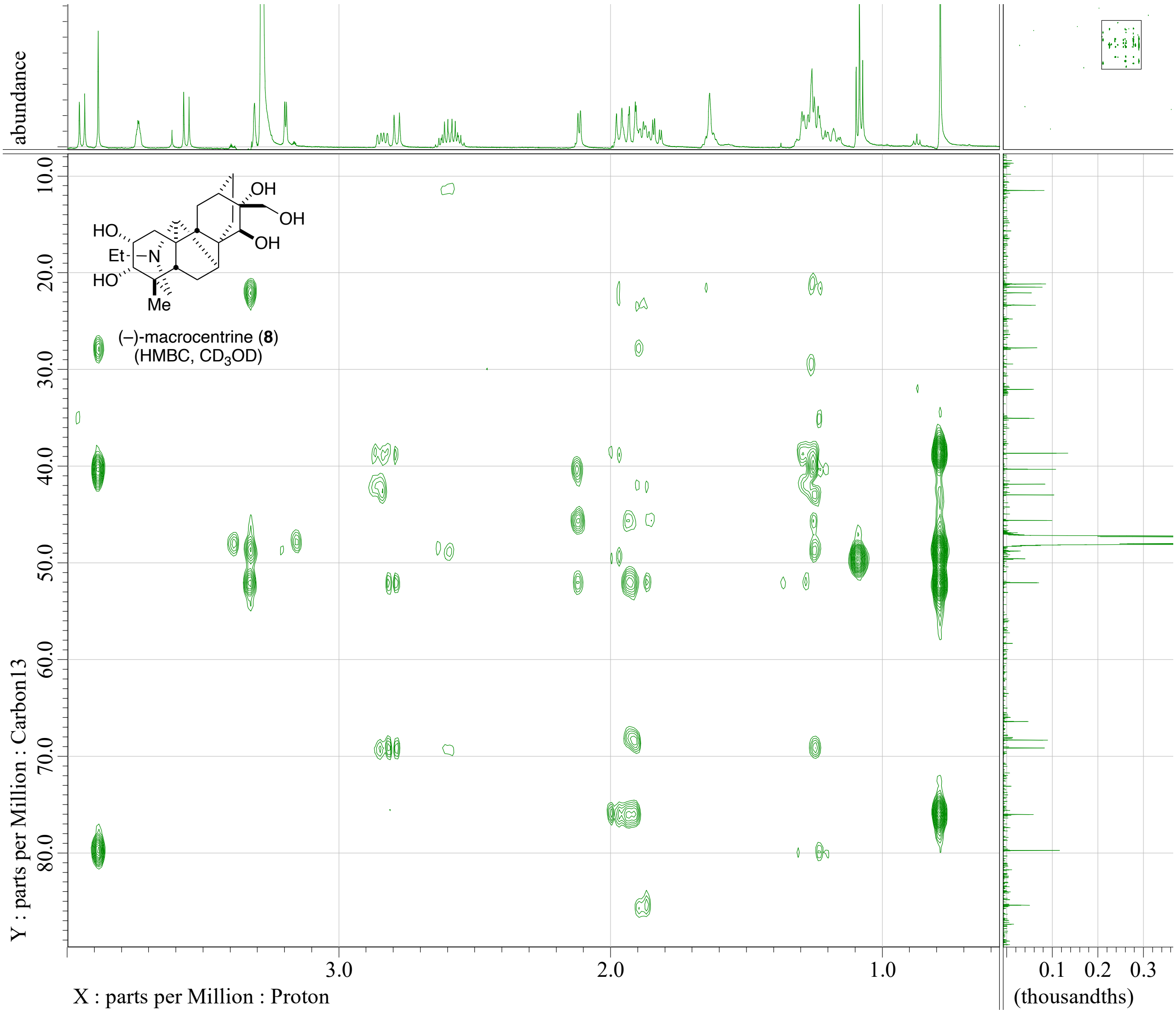
abundance
HO
Et
N
HO
Me
OH
OH
OH
(–)-macrocentrine (8)
(HMBC, CD3OD)
Y : parts per Million : Carbon13
10.0
20.0
30.0
40.0
50.0
60.0
70.0
80.0
3.0
2.0
1.0
X : parts per Million : Proton
0.1 0.2 0.3
(thousandths)

^{1}H-NMR (400 MHz, $CDCl_3$)

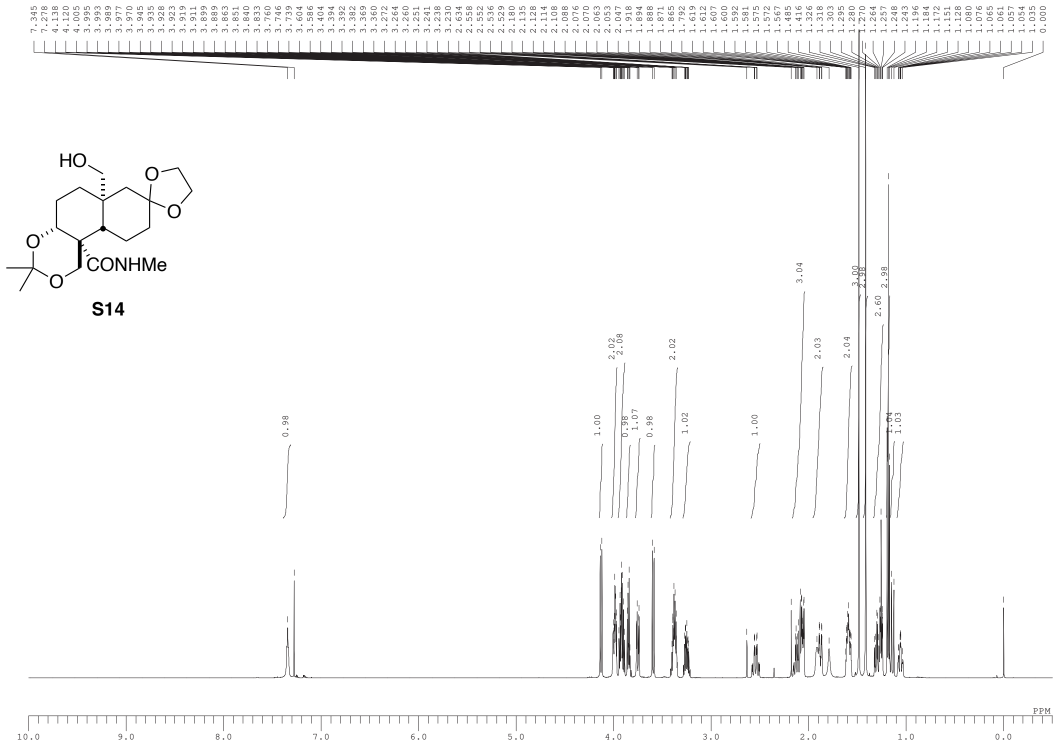

^{13}C-NMR (100 MHz, $CDCl_3$)

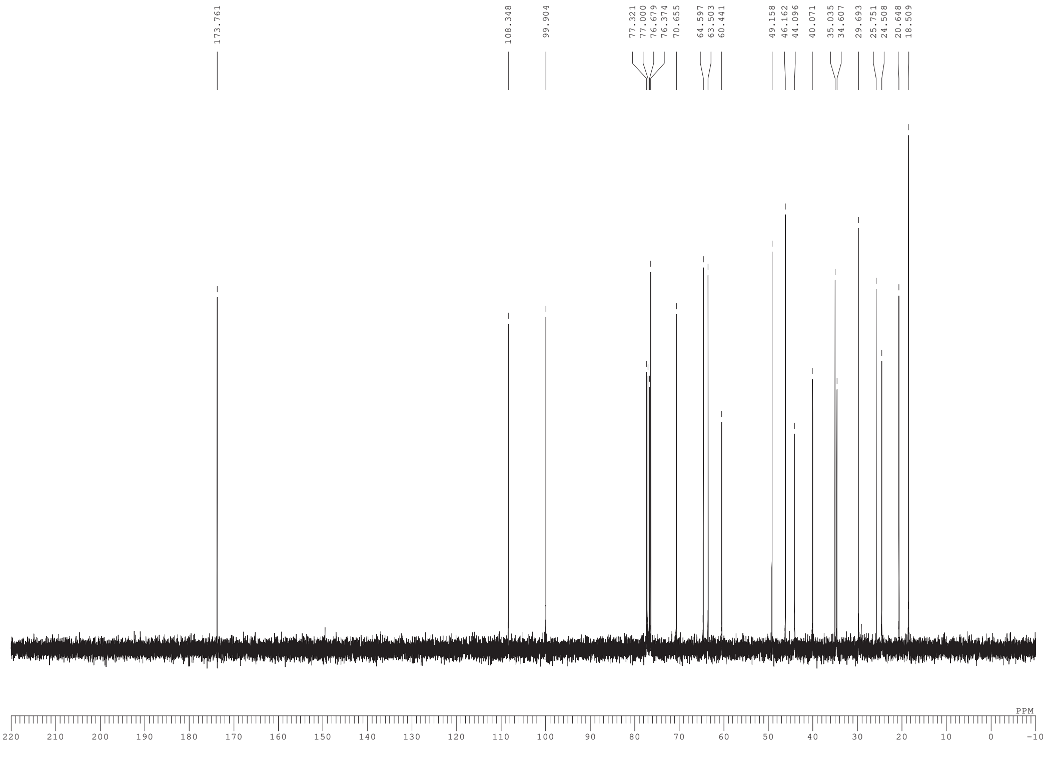

^{1}H-NMR (400 MHz, CD_3OD)

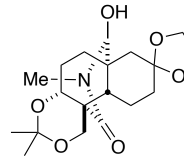

S15

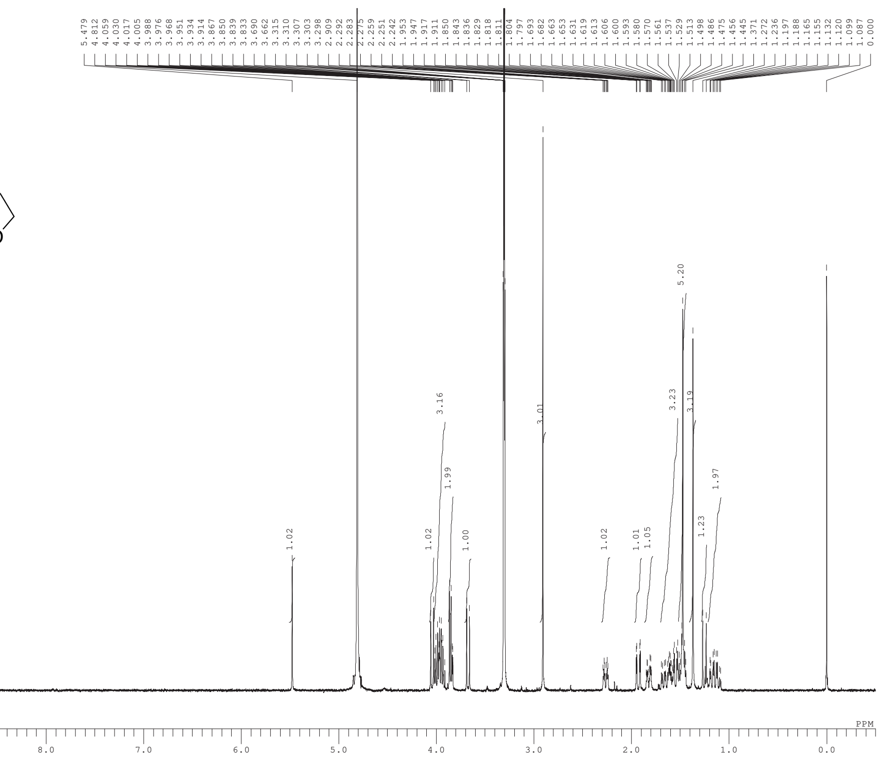

^{13}C-NMR (150 MHz, CD_3OD)

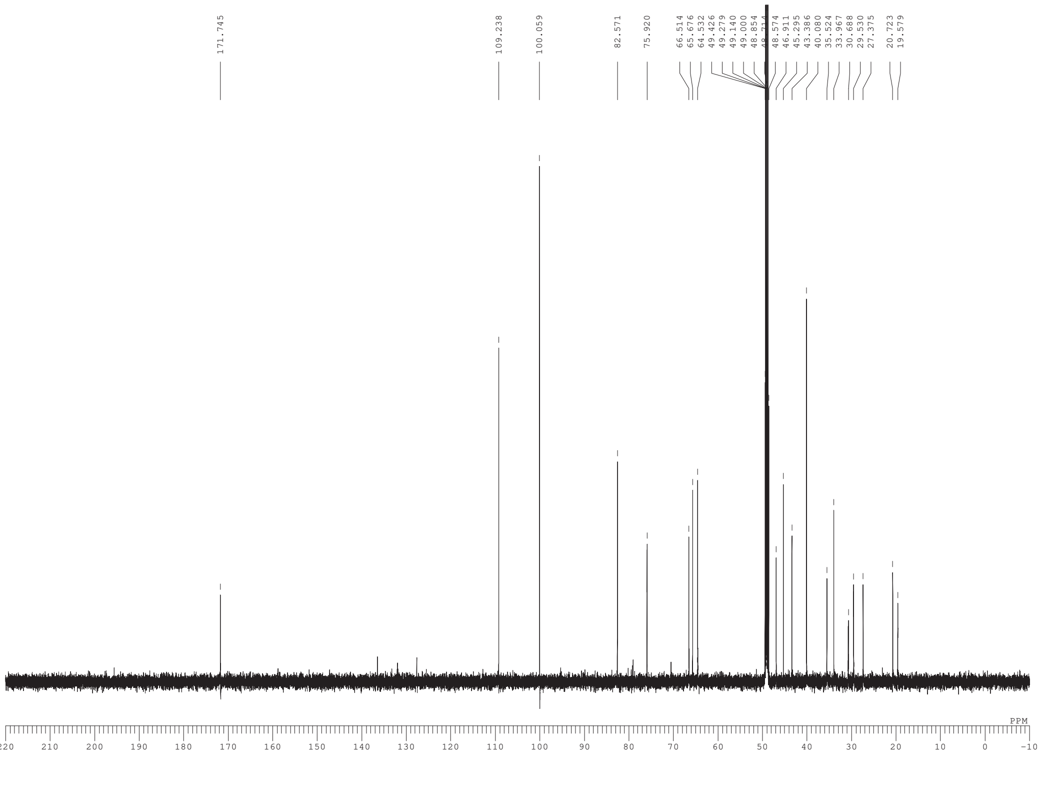

^{1}H-NMR (400 MHz, $CDCl_3$)

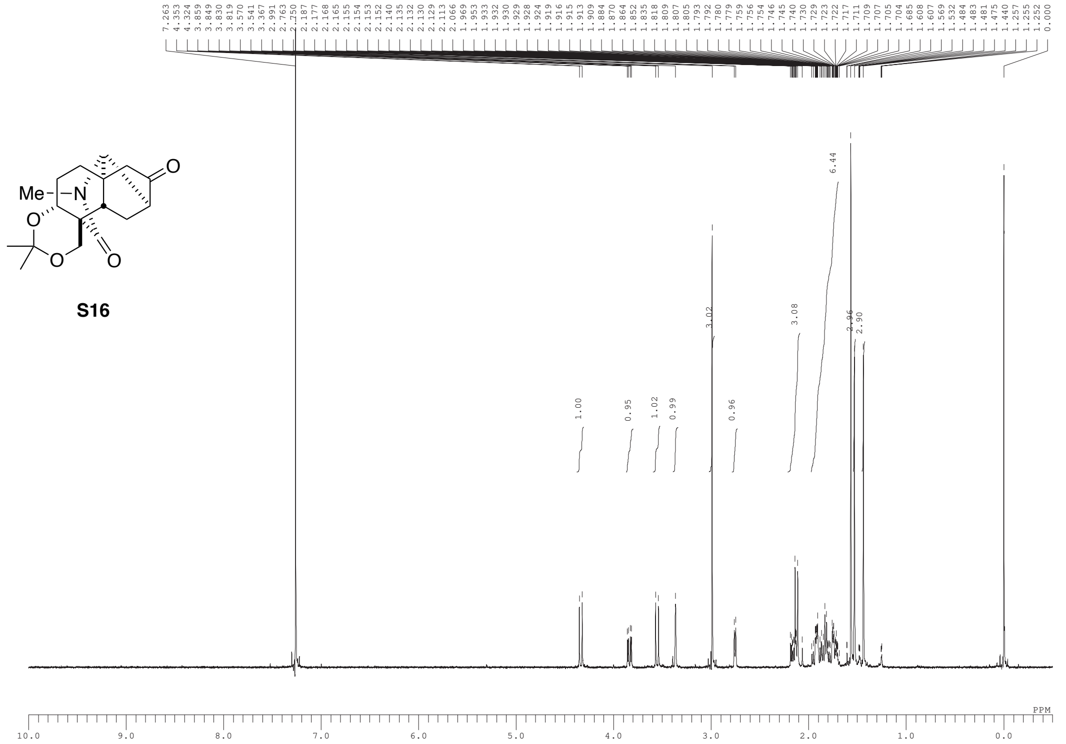

^{13}C-NMR (100 MHz, $CDCl_3$)

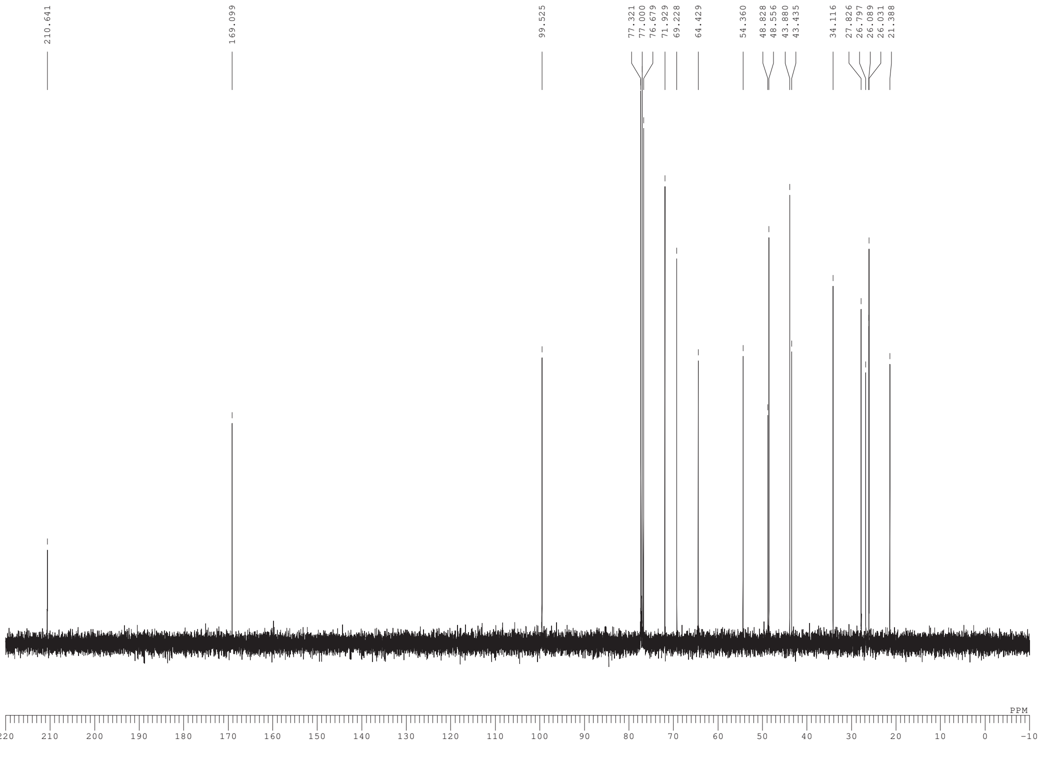

^{1}H-NMR (400 MHz, $CDCl_3$)

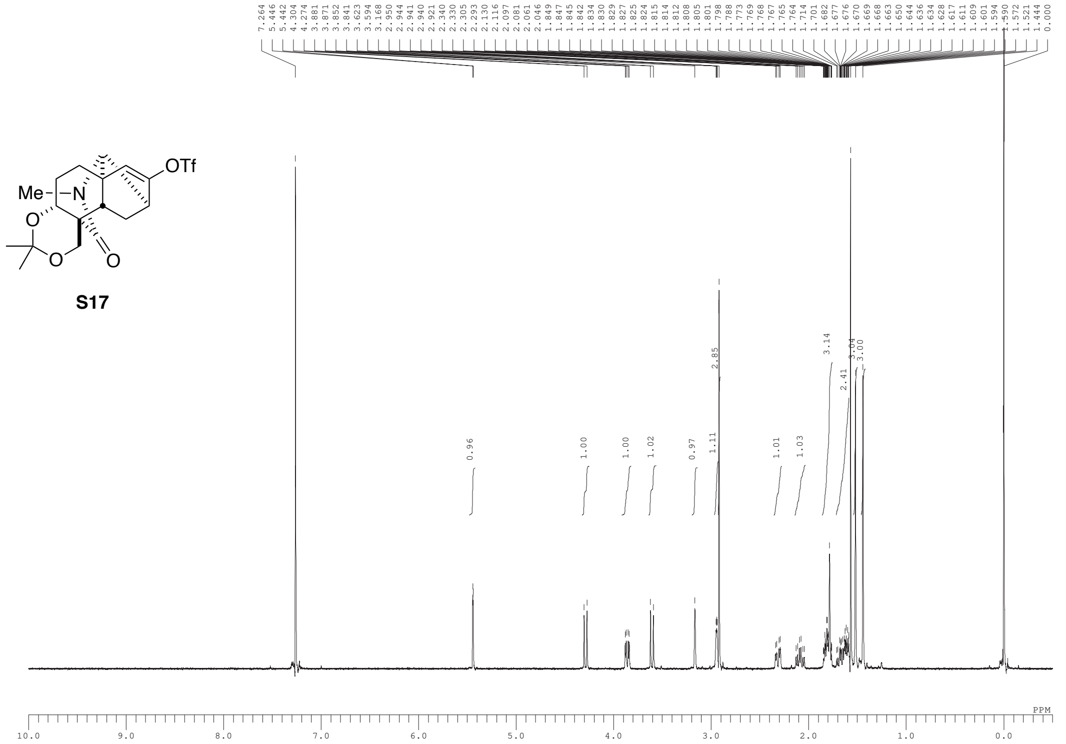

^{13}C-NMR (150 MHz, $CDCl_3$)

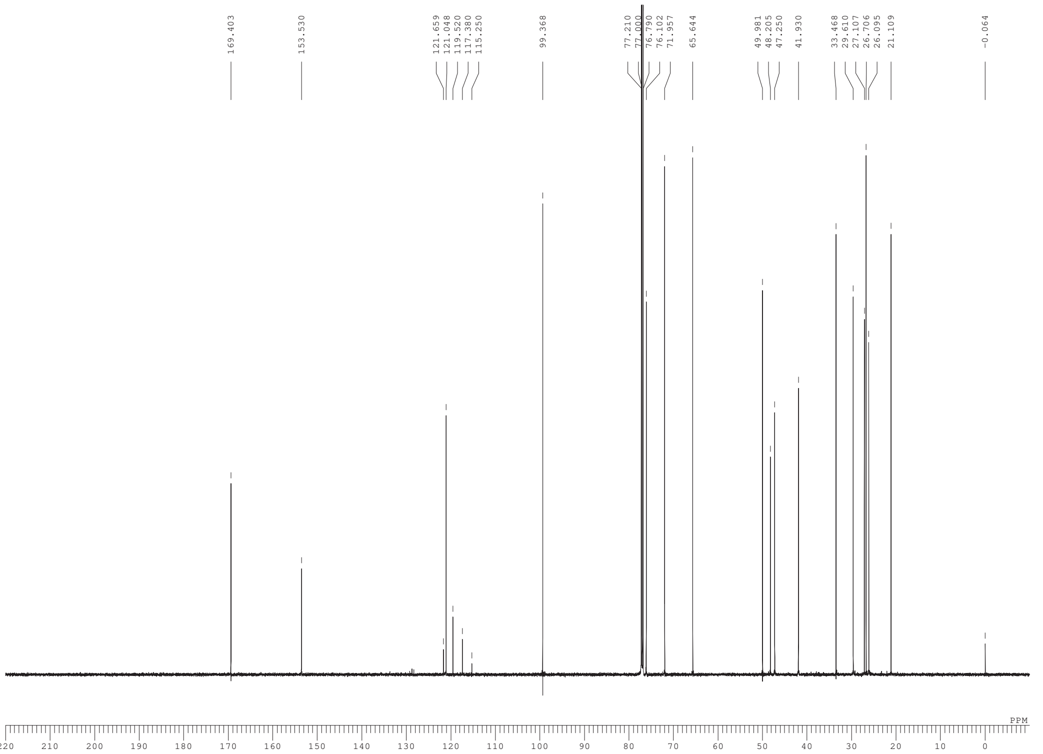

^{1}H-NMR (400 MHz, $CDCl_3$)

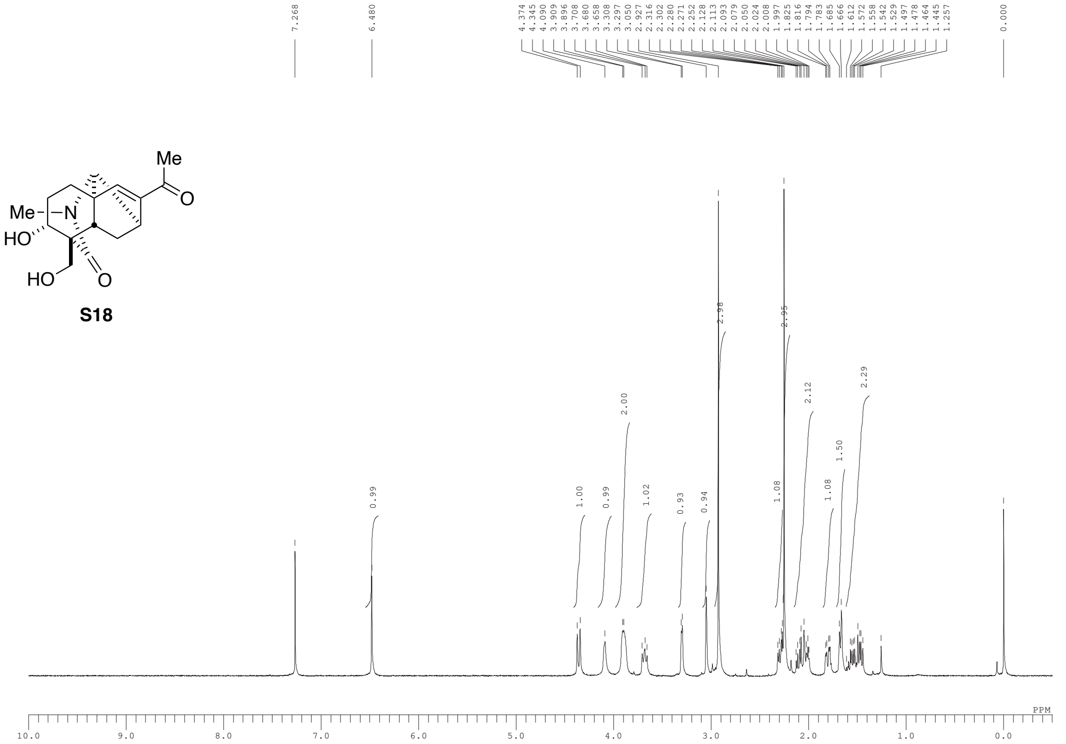

^{13}C-NMR (100 MHz, $CDCl_3$)

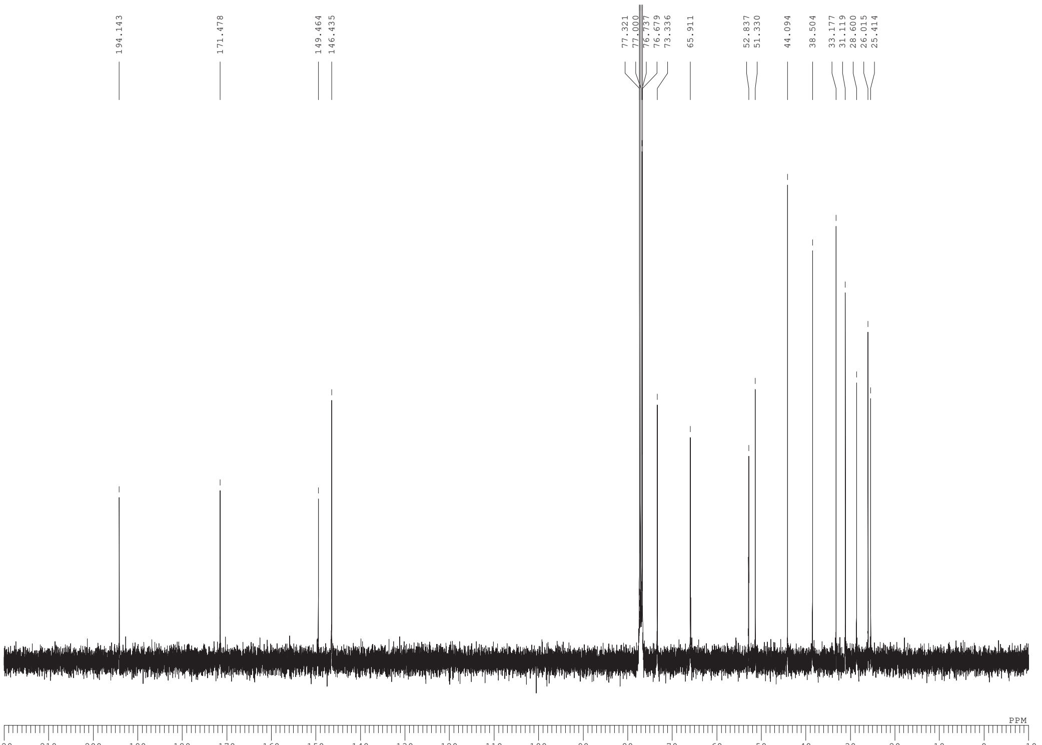

^{1}H-NMR (600 MHz, $CDCl_3$)

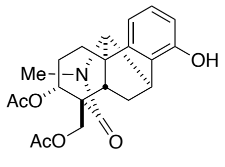

S21

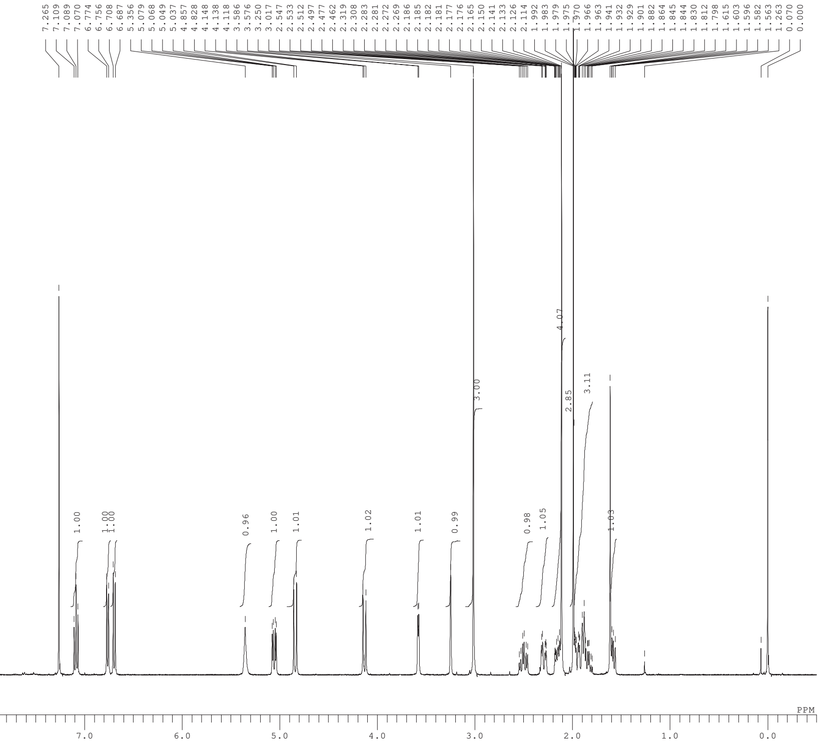

^{13}C-NMR (150 MHz, $CDCl_3$)

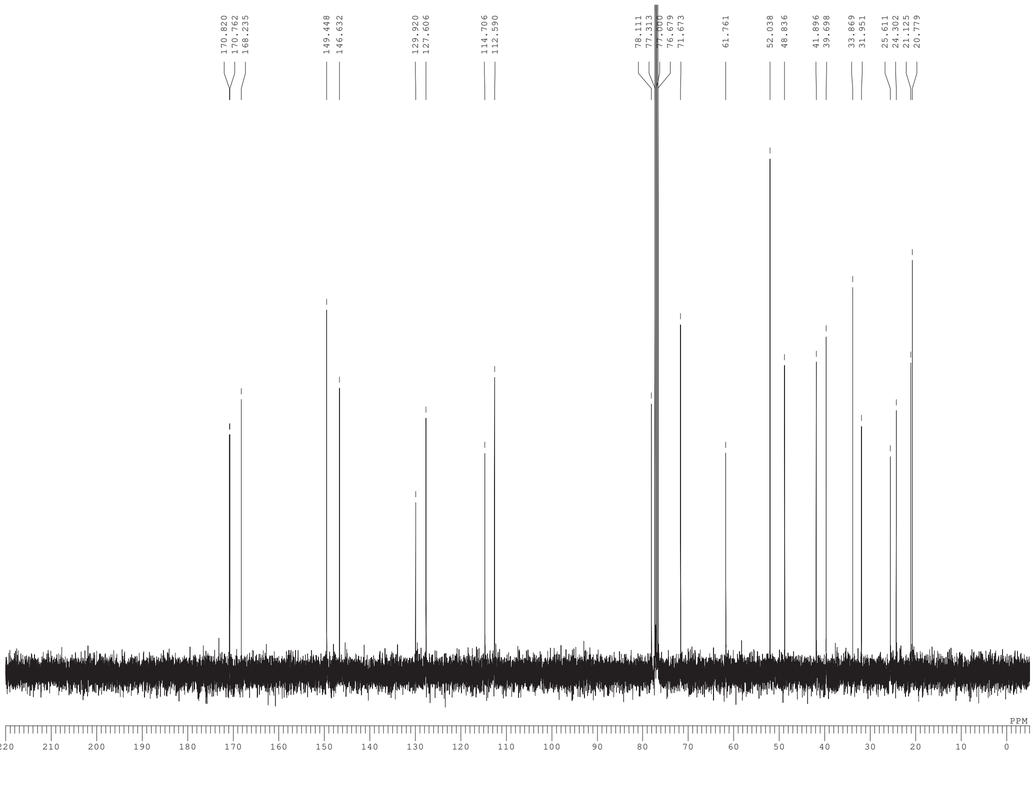

^{1}H-NMR (400 MHz, $CDCl_3$)

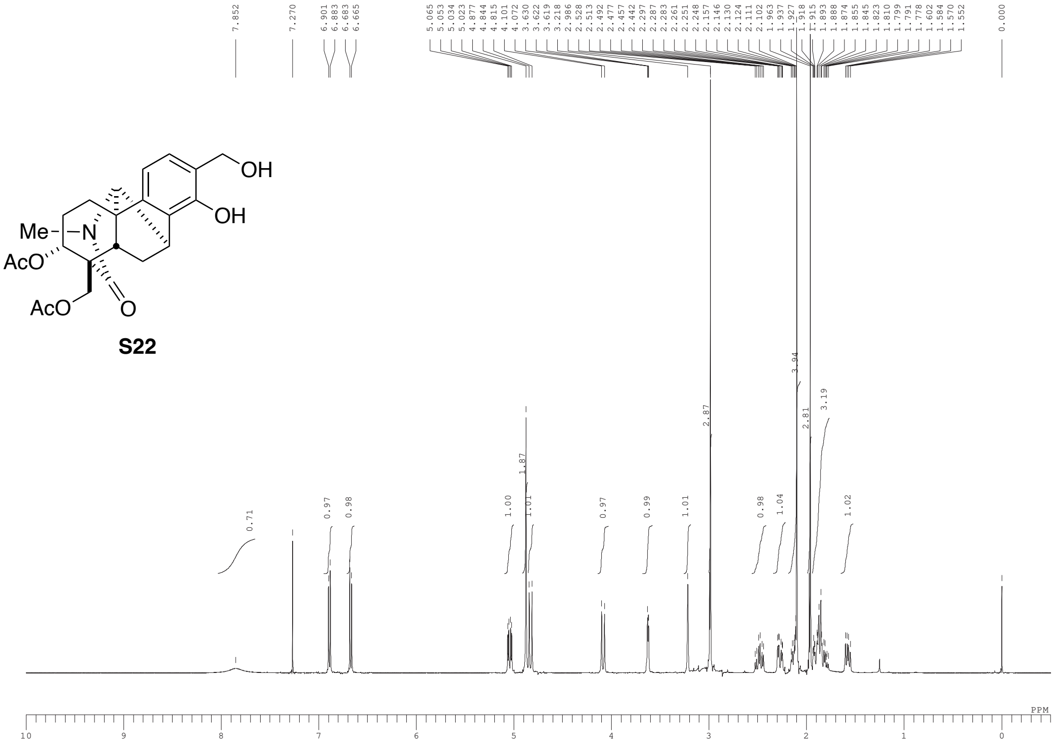

^{13}C-NMR (100 MHz, $CDCl_3$)

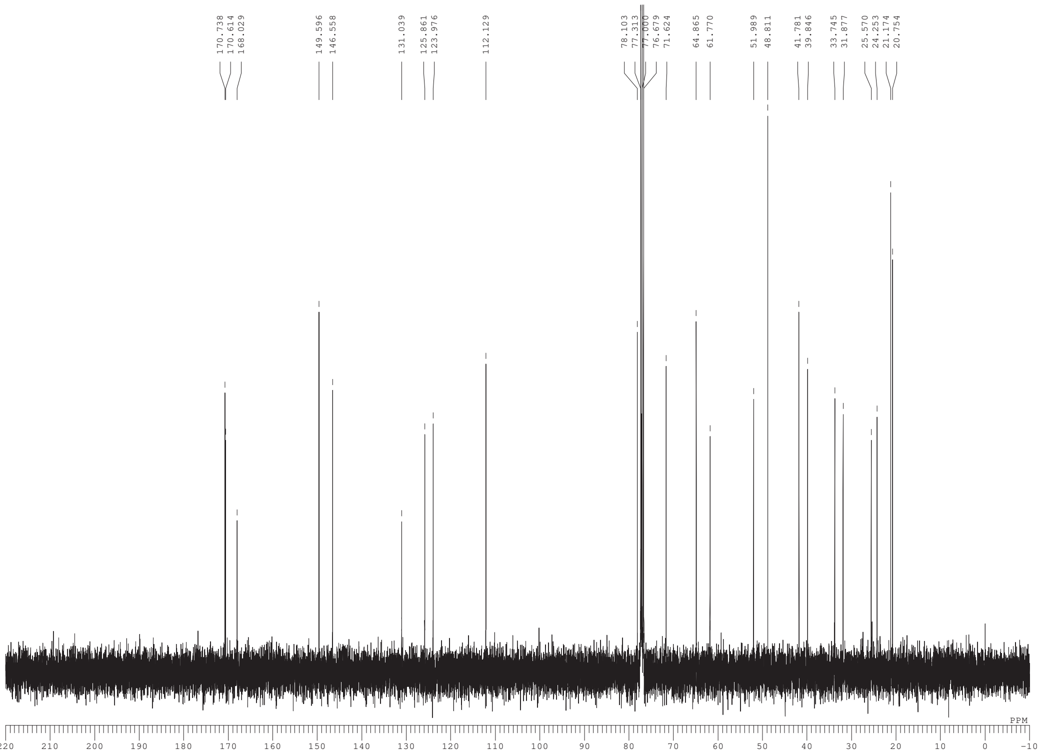

^{1}H-NMR (400 MHz, $CDCl_3$)

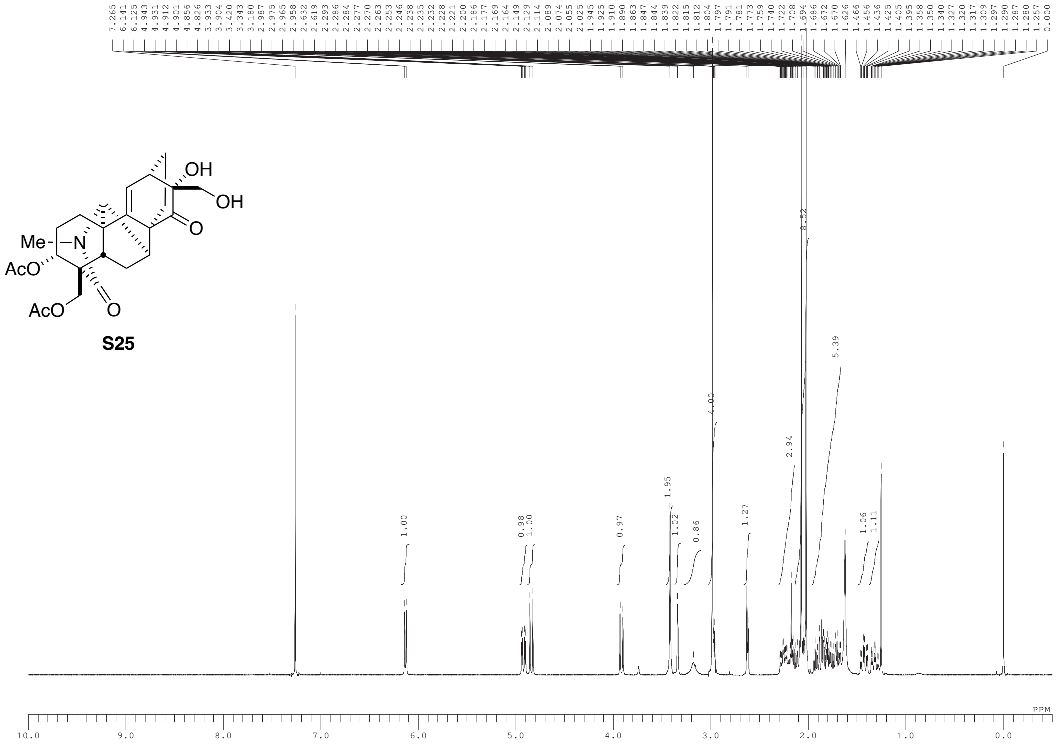

^{13}C-NMR (150 MHz, $CDCl_3$)

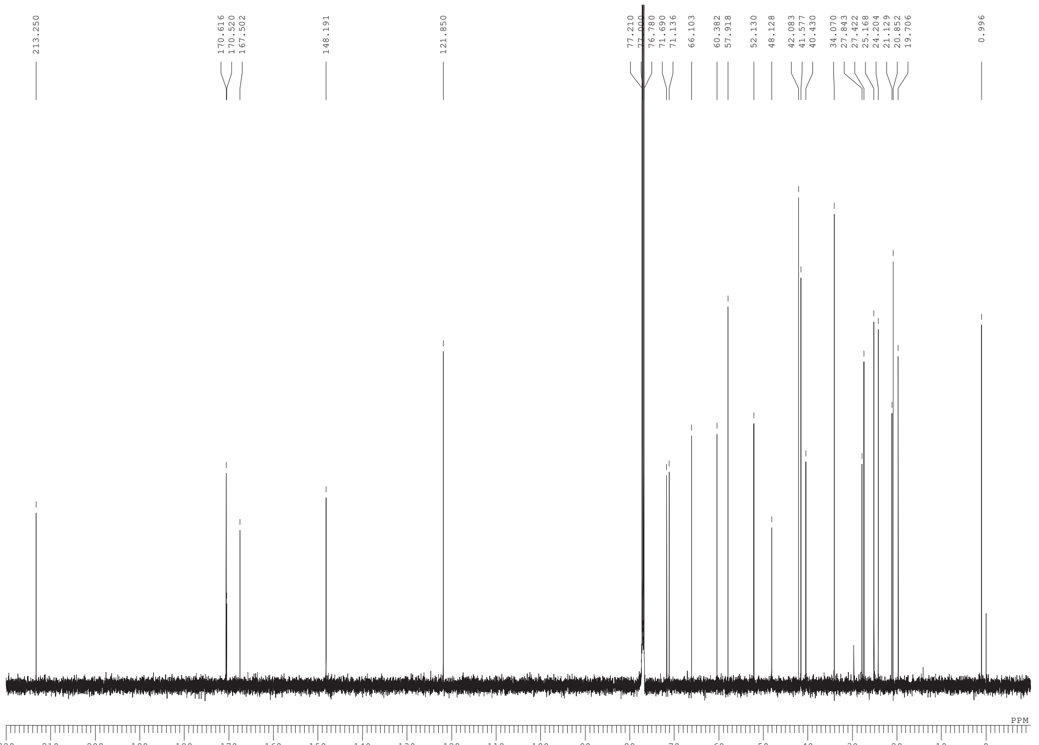

^{1}H-NMR (600 MHz, CD_3OD)

S26

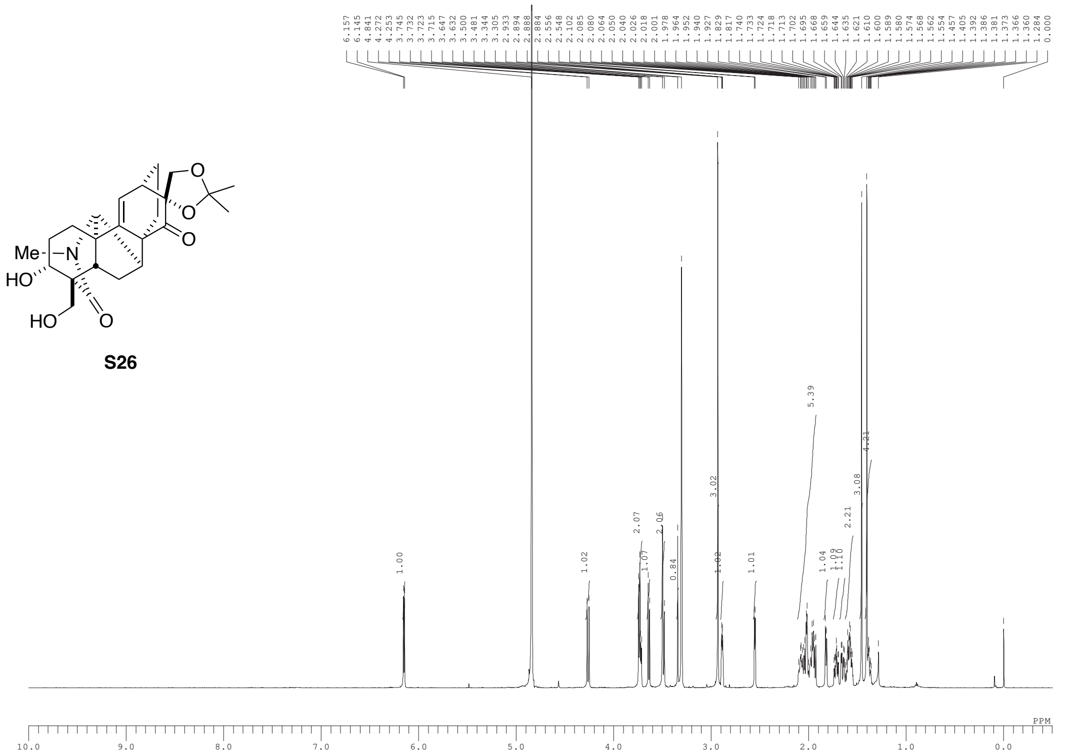

^{13}C-NMR (150 MHz, CD_3OD)

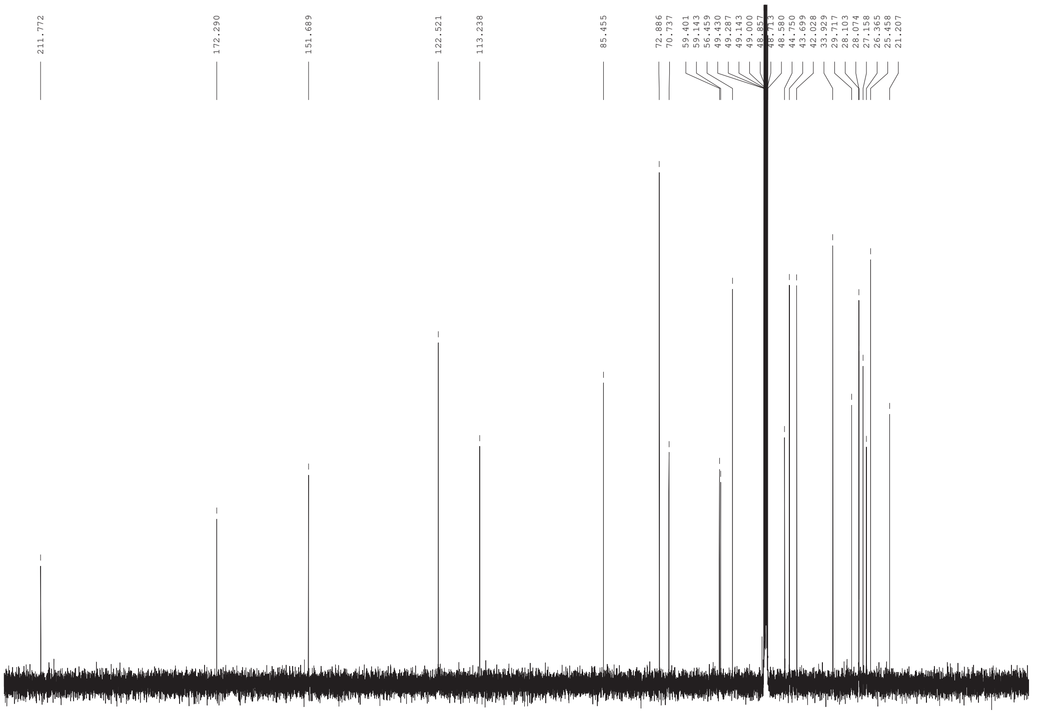

^{1}H-NMR (600 MHz, $CDCl_3$)

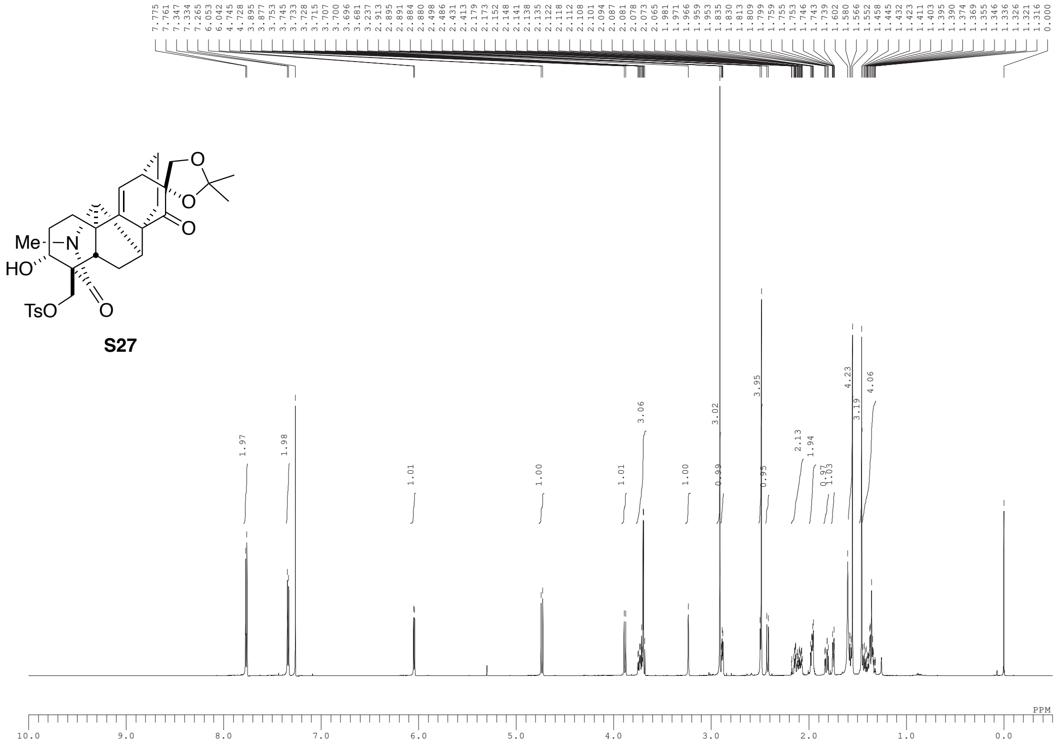

^{13}C-NMR (150 MHz, $CDCl_3$)

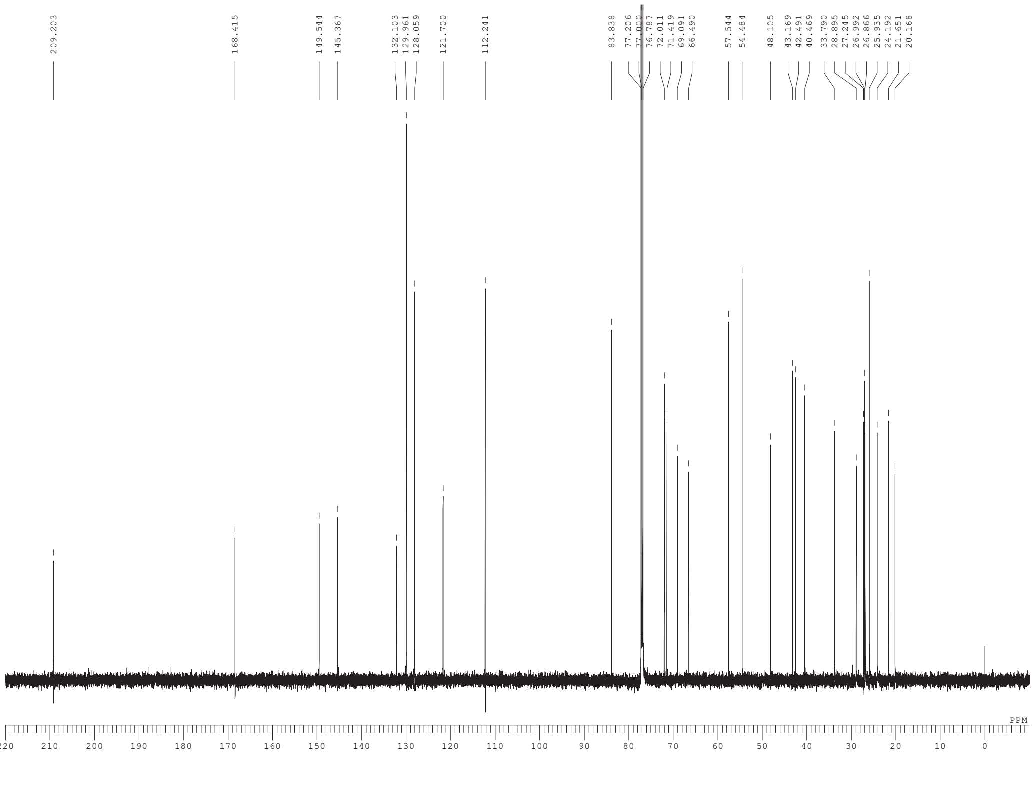

^{1}H-NMR (600 MHz, $CDCl_3$)

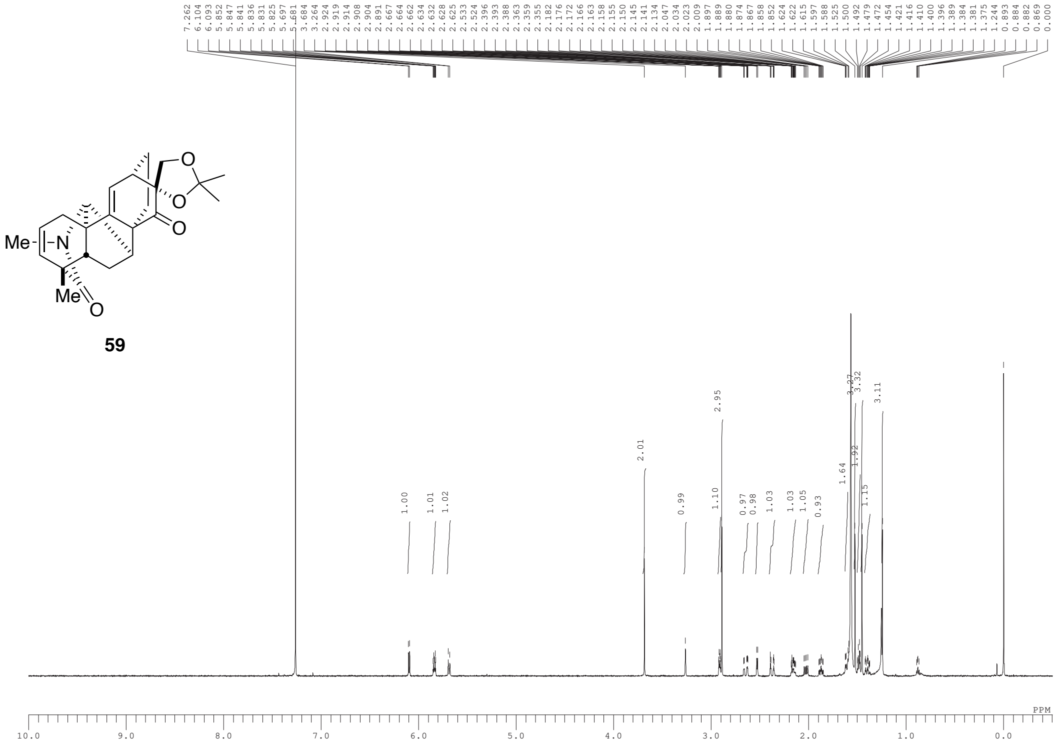

^{13}C-NMR (150 MHz, $CDCl_3$)

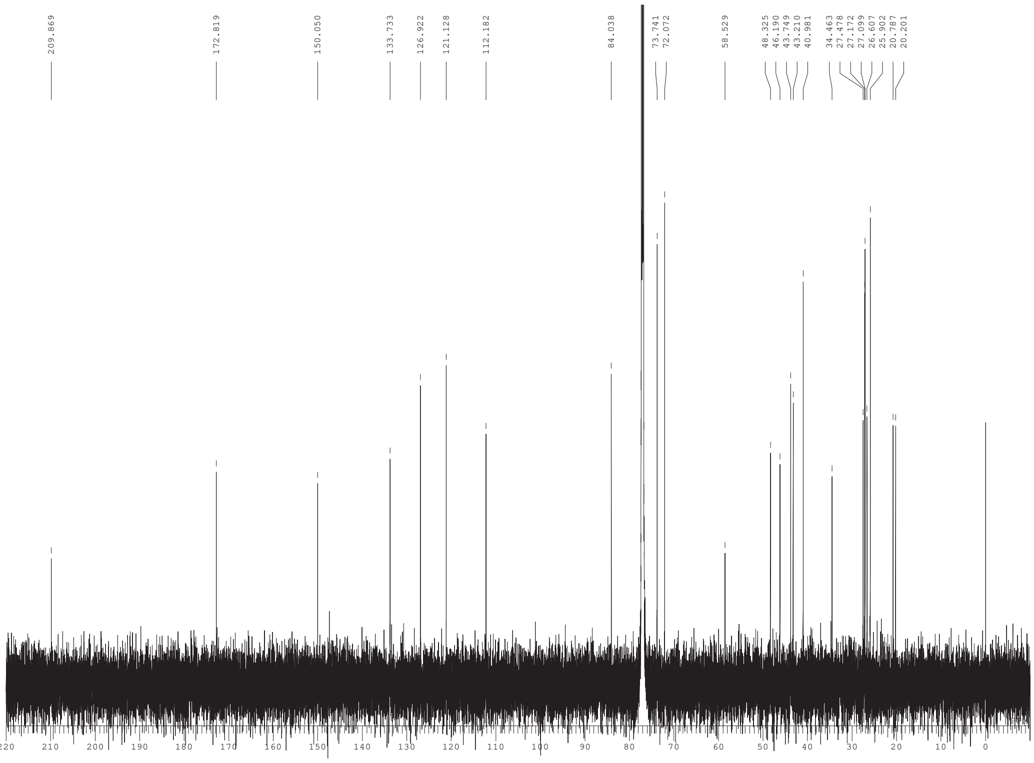

^{1}H-NMR (600 MHz, $CDCl_3$)

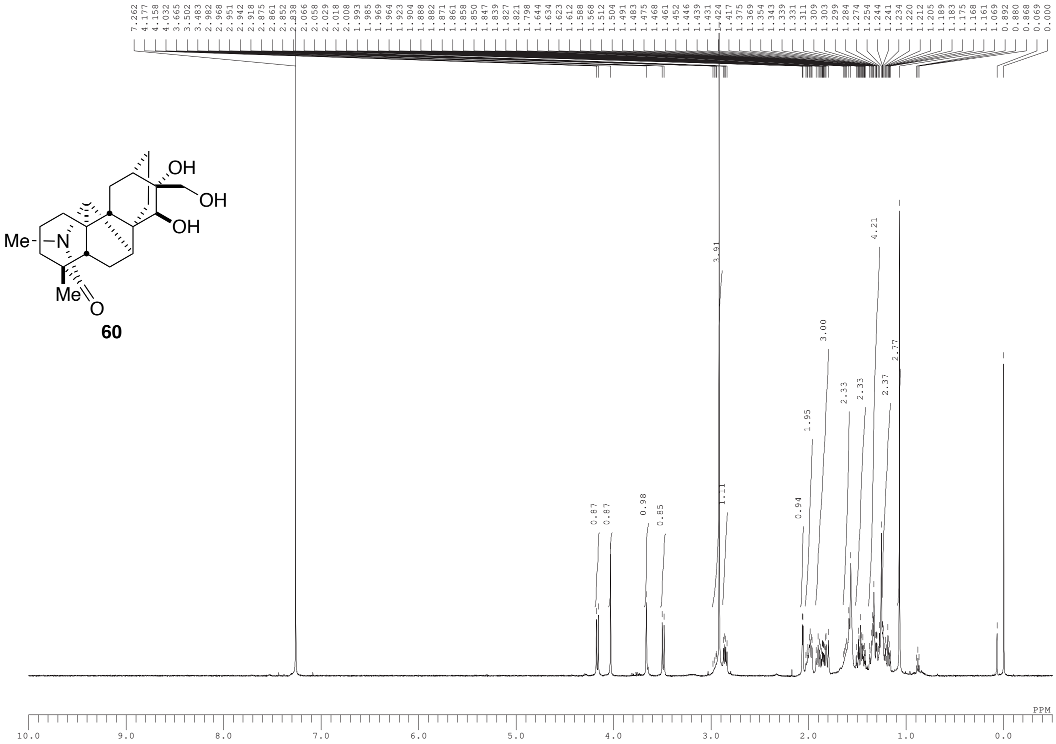

60

^{13}C-NMR (150 MHz, $CDCl_3$)

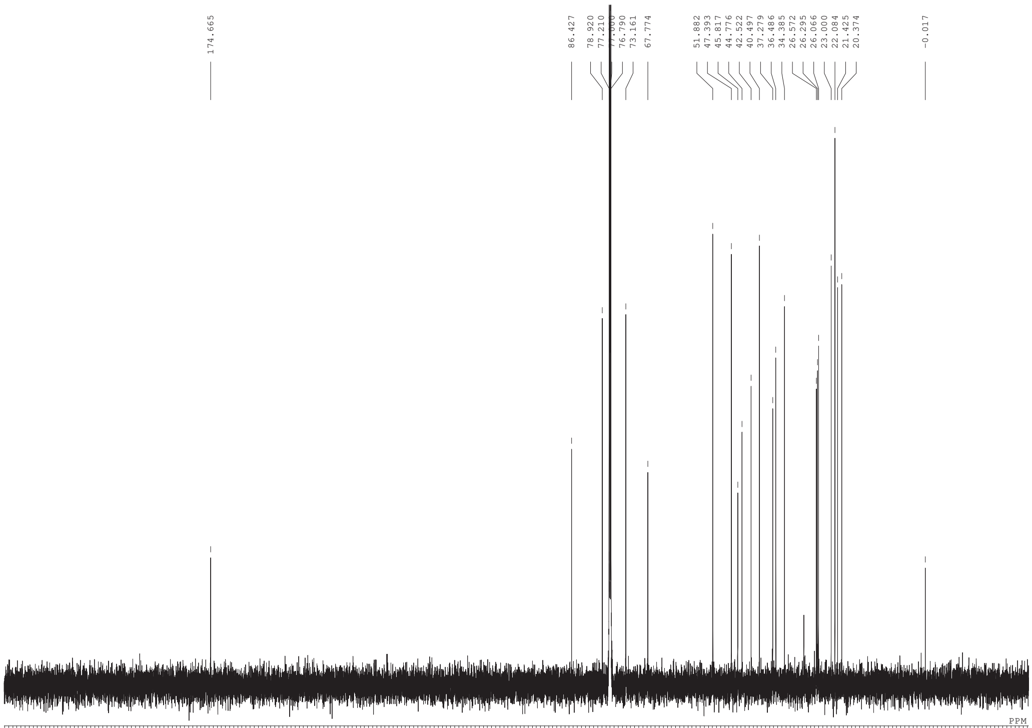

^{1}H-NMR (600 MHz, $CDCl_3$)

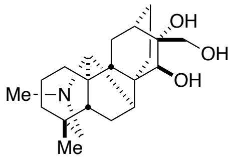

(–)-dictizine (**9**)

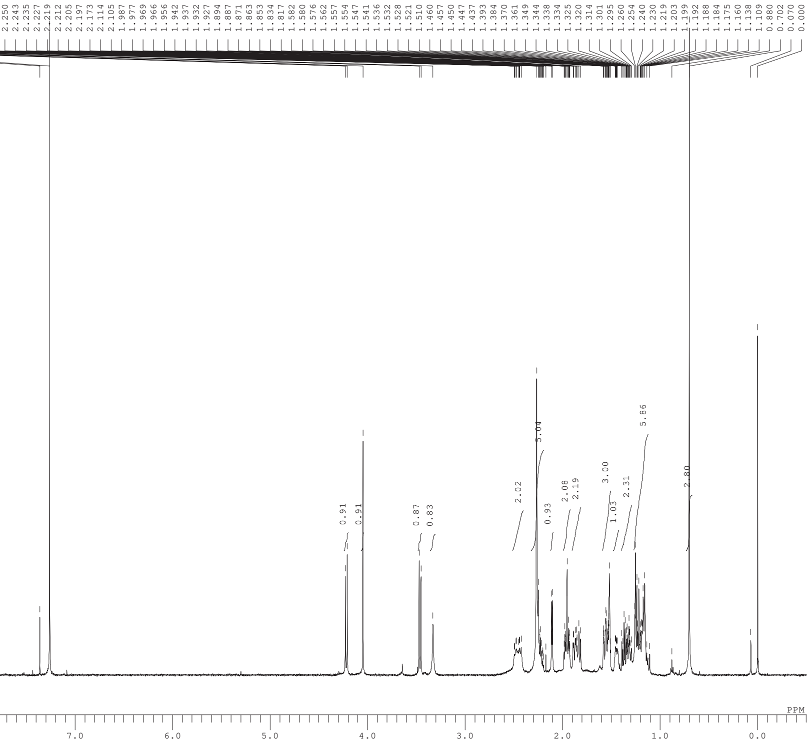

^{13}C-NMR (150 MHz, $CDCl_3$)

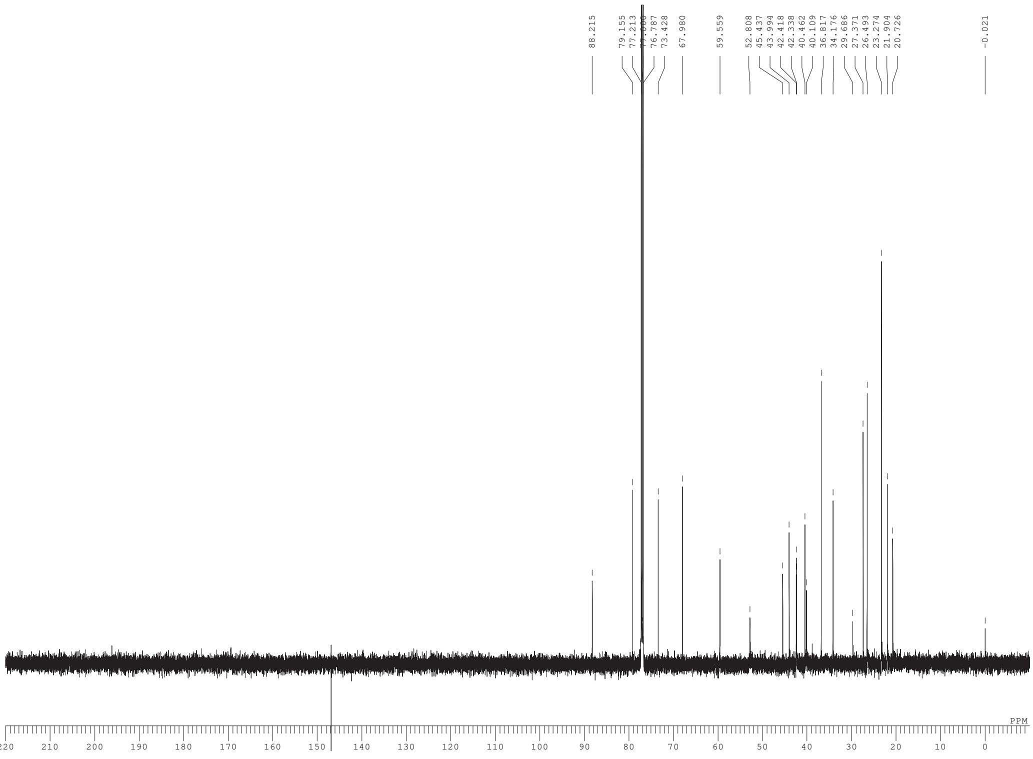

^{1}H-NMR (600 MHz, $CDCl_3$)

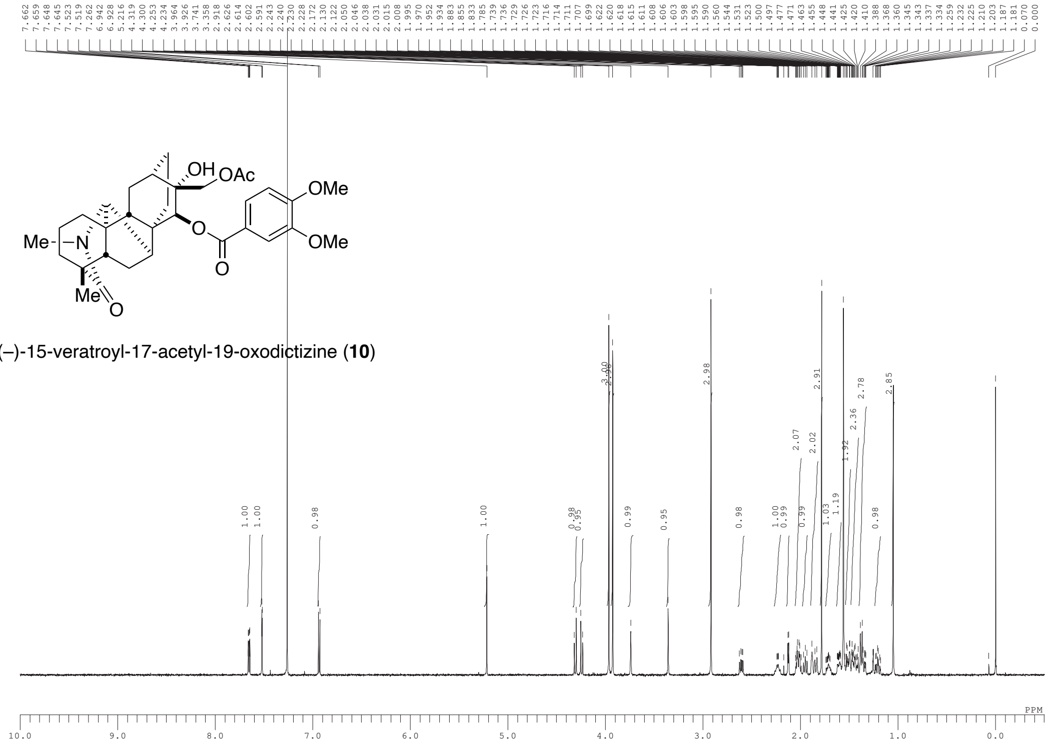

^{13}C-NMR (150 MHz, $CDCl_3$)

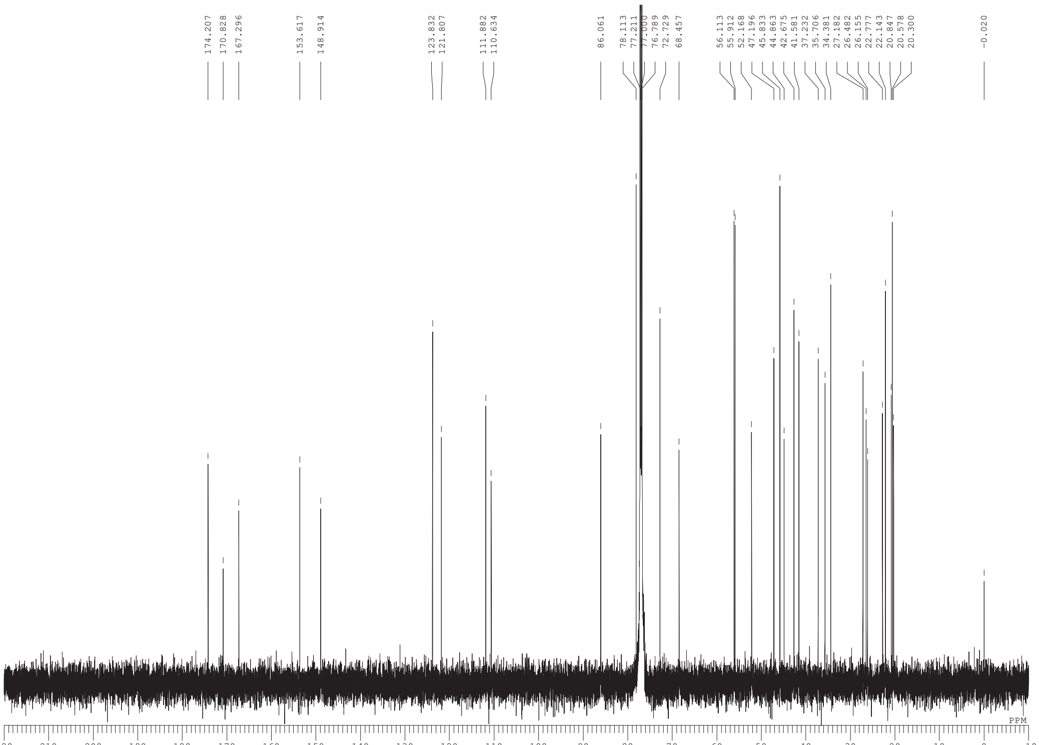

Supplement: Supplementary file 1 — Supporting Information [file ANIE-65-e21481-s001.pdf]
